# Damping amyloid‐associated conformational fluctuations in a protein by an engineered diselenide bridge

**DOI:** 10.1002/pro.70697

**Published:** 2026-07-21

**Authors:** Yanwu Yang, Balamurugan Dhayalan, Andreas Ehnbom, Orit Weil‐Ktorza, Norman Metanis, Michael A. Weiss

**Affiliations:** ^1^ Department of Biochemistry, Molecular Biology & Pharmacology Indiana University School of Medicine Indianapolis Indiana USA; ^2^ Institute of Chemistry The Hebrew University of Jerusalem Jerusalem Israel

**Keywords:** diabetes mellitus, nonstandard protein engineering, protein dynamics, protein stability, unnatural mutagenesis

## Abstract

Polypeptide cross‐β assembly, characteristic of diverse proteotoxic diseases, defines a general thermodynamic ground state and limits the shelf lives of peptide‐ and protein therapeutics. A model is provided by insulin. Although the hormone contains a predominance of α‐helix, its fibrils exhibit cross‐β reorganization. In the real world, aggregation‐coupled fibrillation of insulin underlies its degradation above room temperature, impairing activity and imposing a complex global “cold chain” of transport and storage. Here, we describe biophysical protection of an insulin analog at an elevated temperature by an engineered diselenide bridge. Our studies focused on insulin *glargine*, the active ingredient of long‐acting formulations in broad clinical use. Insoluble in a subcutaneous depot due to its shifted isoelectric point, the analog dissolves at pH 4.0 and so, unlike neutral formulations of the wild‐type hormone, is unprotected by zinc‐mediated hexamer assembly. Whereas at 37°C the fibrillation lag time of insulin *glargine* is accelerated by fourfold relative to WT insulin, such instability is circumvented by pairwise substitution of Cys^A6^ and Cys^A11^ by selenocysteine. Protection from fibrillation correlates with augmented resistance to pepsin cleavage, guanidine denaturation, and thermal unfolding. Although NMR structures of insulin *glargine* and its diselenide analog are similar, damping of conformational fluctuations is evidenced by patterns of ^1^H‐NMR chemical shifts, helix‐associated NOEs, amide‐resonance line widths, and ^1^H‐^2^H amide‐proton exchange. Such damping is discussed in relation to molecular dynamics simulations. Demonstrating a likely mechanistic relationship between fibrillation and native‐state conformational fluctuations, our findings highlight the translational promise of “dynamic engineering” via nonstandard mutagenesis.

## 1 INTRODUCTION

Toxic amyloid deposits and their oligomeric precursors (Dobson 2003) are characteristic of diverse diseases, including Alzheimer's disease and prion‐related encephalopathy (Bellotti et al. 1999; Carrell and Gooptu 1998). Such deposits exhibit cross‐β assembly (Jimenez et al. 2002), the general thermodynamic ground state of polypeptides as a class of heteropolymers (Figure S1, Supporting Information) (Chiti and Dobson 2006). Insight into mechanisms of amyloid formation has been obtained through studies of monogenic proteotoxic syndromes, including unstable variants of transthyretin (Jiang et al. 2001; Peterson et al. 1998) and lysozyme (Booth et al. 1997). The propensity of peptides and proteins to form amyloid above room temperature (also designated *fibrillation*) poses challenges in therapeutic protein engineering and pharmacology (Ke et al. 2020). Aggregation‐coupled misfolding leads to loss of biological activity (Stefani and Dobson 2003), limiting shelf life and complicating shipment and storage (Krause and Sahin 2019). On injection, amyloid and its lower‐molecular‐mass precursors can be pro‐inflammatory (Lewis et al. 2023; Nakamura et al. 2018) and provoke development of neutralizing antibodies (Van Haeften 1989). These problems have stimulated strategies to stabilize protein formulations, such as through protective ligand binding (Rahban et al. 2023) or addition of hydrophilic solubility tags (Ebrahimi and Samanta 2023). These technologies are especially important in regions of the developing world where electricity and refrigeration are unavailable (Barnes and Floor 1996). Because stochastic formation of amyloidogenic seeds (Figure S2) (Kelly 2000) hinges on conformational fluctuations in a protein's native state (Chiti and Dobson 2009), we sought to protect a therapeutic protein from fibrillation through damping of such fluctuations. Our approach (designated *dynamic protein engineering*) exploits unnatural mutagenesis (England 2004) to mitigate cryptic packing defects in an otherwise conserved hydrophobic core.

**FIGURE 1:**
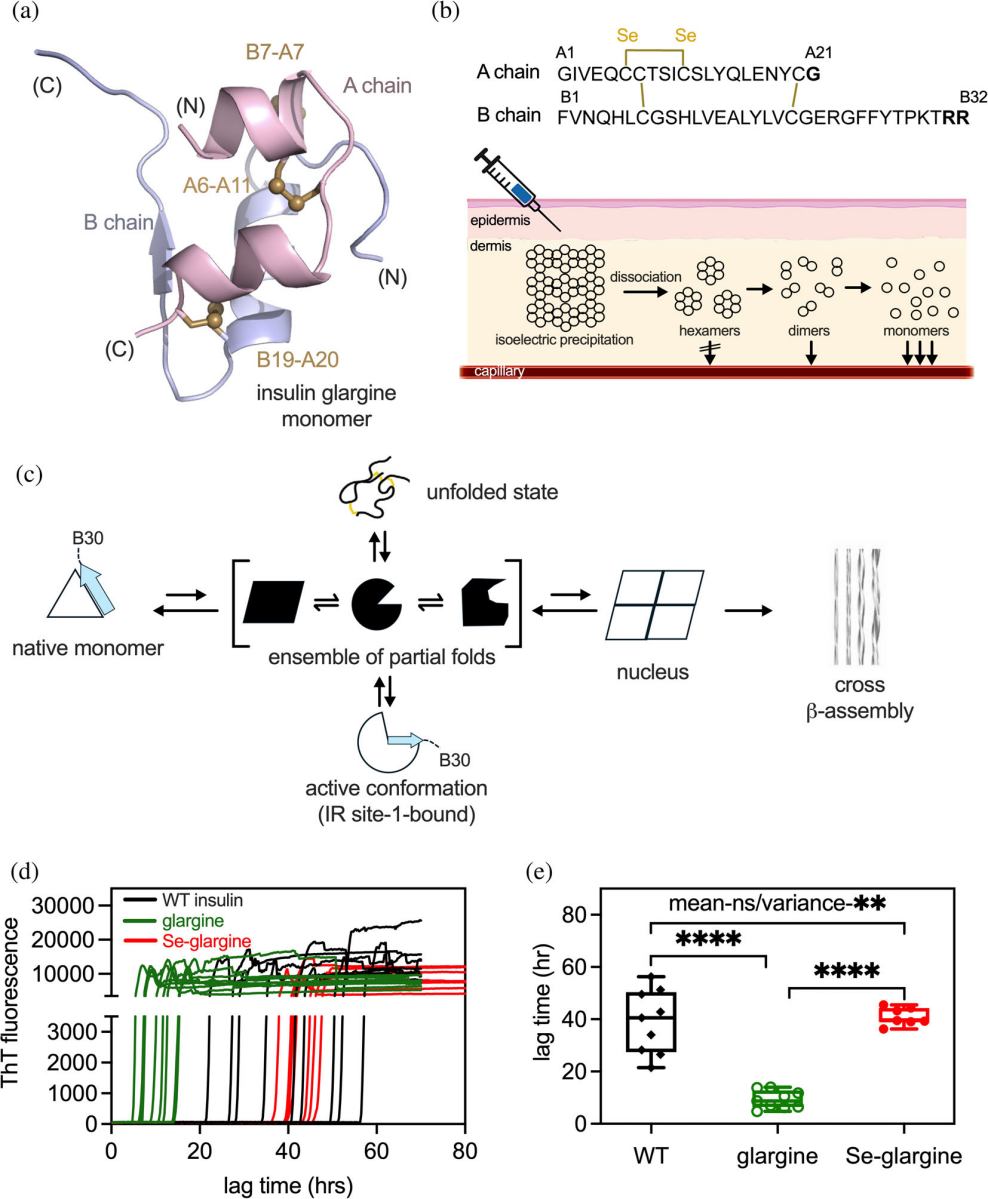
Insulin *glargine* and comparative fibrillation assays. (a) Ribbon representation of the X‐ray structure of glargine (PDB code 4IYD). The A chain is highlighted in light pink, B chain in light blue and disulfide bridge in gold. Sulfur atoms are represented as *spheres*. (b) Sequence of insulin glargine (top panel). Initially formulated at an acidic pH, insulin glargine forms microcrystalline aggregates upon subcutaneous injection, which slows its dissociation into glargine monomers (bottom). (c) Mechanism of fibrillation. The insulin monomer (left) may first undergo partial unfolding (center, partial folds is represented by black shapes; see Figure S5 for various conformational states obtained from molecular dynamics simulations), followed by complete unfolding‐resembling its receptor‐bound conformation‐which facilitates the formation of a nucleus or a cross β‐structure (far right). This panel was adapted from Nielsen et al. (2001). The image of insulin protofilament (far right) is reproduced from Jimenez et al. (2002). (d) Plot showing ThT fluorescence of insulin fibrils. Twofold increase in fluorescence intensity from the baseline provided the definition of lag times. (e) Box‐and‐whisker plots showing the fibrillation lag times of insulin analogs at 37°C (ns‐not significant, **p < 0.01, ****p < 0.0001).

A model is provided by insulin (Figure 1a), a prototypical amyloidogenic domain (Brange et al. 1997; Jimenez et al. 2002; Manno et al. 2006; Manno et al. 2007; Podestà et al. 2006). Fibrillation constrains the formulation and shelf lives of insulin analogs designed to optimize pharmacokinetic properties (Figure S3) (Hirsch et al. 2020; see sect. S1.1 of Data S1 for complementary insulin formulation strategies); an example is insulin *glargine* (Figure 1b), the active component of long‐acting clinical formulations (Bolli and Owens 2000). Whereas three‐dimensional structures of insulin fibrils have attracted broad interest (Suladze et al. 2024; Wang et al. 2023; Yang et al. 2010), molecular mechanisms of fibrillation (including structures of non‐native aggregates; Hua et al. 2006; Huang et al. 2006) are less‐well understood (Brange et al. 1997; DuBay et al. 2004; Hua and Weiss 2004; Ivanova et al. 2009; Lenton et al. 2025; Nielsen et al. 2001). An overview is provided in Figure 1c: its complexity is underscored by evidence that insulin protofilaments can adopt distinct modes of higher‐order assembly, leading to conformational “strains” (Dzwolak et al. 2004; Lenton et al. 2025; Surmacz‐Chwedoruk et al. 2012) analogous to those encountered in prion‐related encephalopathies (Prusiner 1991). For translational purposes, however, such complexity may be circumvented through focus on the structure of the native insulin monomer (Hua et al. 1991; Hua et al. 1996; Olsen et al. 1996): its fibrillation can be blocked by insertion of peptide cross‐links compatible with biological activity (Menting et al. 2013; Menting et al. 2014; Scapin et al. 2018; Weis et al. 2018), but incompatible with cross‐β assembly (Wang et al. 2023). Such cross‐links constrain both the native state and ensemble of partial folds (central panel in Figure 1c). Examples are provided by the shortened C domain of single‐chain insulin analogs (SCIs; Hua et al. 2008; Phillips et al. 2012; Glidden et al. 2018) and by insertion of an additional disulfide bridge between A‐ and B chains (Brunel et al. 2019; Vinther et al. 2013; Vinther et al. 2015). These modifications lie on the protein surface (Figure S4).

As illustrated in Figure 1c, key initial steps in the conformational pathway of insulin fibrillation are (a) dissociation of protective hexamers and dimers to liberate the isolated monomer (Brange et al. 1997) and (b) conformational fluctuations in the monomer (Ahmad et al. 2005; Hua and Weiss 2004; Surmacz‐Chwedoruk et al. 2012) that enable its non‐native aggregation as an amyloidogenic seed (see sect. S1.2 of Data S1 for low‐abundance amyloidogenic precursor species; Hua and Weiss 2004; Ahmad et al. 2005; Surmacz‐Chwedoruk et al. 2012). Subglobal fluctuations are of particular interest because (c) extended molecular‐dynamics (MD) simulations of the insulin monomer suggest a complex equilibria among partial folds (central panel of Figure 1c and Figure S5; Busto‐Moner et al. 2021) and (d) relative susceptibilities of insulin analogs to fibrillation, as measured by mean lag times prior to exponential growth (Ahmad et al. 2005), do not generally correlate with thermodynamic stabilities (Δ*G*_u_ values in Table S1, as probed by chemical denaturation studies; Ahmad et al. 2003; Hua and Weiss 2004). This line of reasoning raises the question: how might amyloid‐associated conformational fluctuations be damped? Past efforts to address this question have focused on *surface* modifications, such as binding of stabilizing ions or ligands (sect. S1.2 of Data S1) (Brange and Langkjœr 1993; Lenton et al. 2025), optimization of solvent‐exposed α‐helical N‐/C‐cap residues (Glendorf et al. 2012; Weiss et al. 2001) or (as above) an engineered solvent‐exposed disulfide bridge (Vinther et al. 2013). Although of biophysical interest, such surface modifications can alter signaling (Gallagher et al. 2013; Glidden et al. 2018; Svendsen et al. 2014) or immunogenicity (Ottesen et al. 1994; Schroer et al. 1983), discouraging clinical translation (Hansen et al. 2011).

Might instead the *hydrophobic core* of insulin be optimized to damp conformational fluctuations? At first glance this alternative may appear unpromising, as insulin's core is unusually well conserved relative to other vertebrate protein families (Baker et al. 1988; Conlon 2001). Indeed, core packing is constrained by internal disulfide bridges A6‐A11 and B19‐A20 (gold spheres in Figure 1a and Figure S6). Further, core residues can play a dual role in receptor binding due in some cases to partial exposure at edges of the receptor‐binding surfaces and in other cases to their exposure following conformational changes on receptor binding (Hua et al. 1991; Menting et al. 2013; Menting et al. 2014; Scapin et al. 2018; Weis et al. 2018). Potentially stabilizing substitutions (such as at sites Leu^B6^, Val^B12^, Leu^B15^, Ile^A2^, and Val^A3^; Figure S6) may thus impair bioactivity. Core substitutions may also perturb nascent protein folding (hence limiting scalable manufacturing) or protective native self‐assembly (limiting formulation stability; Dhayalan et al. 2021). Given these barriers, insulin analogs containing core substitutions have not been introduced into clinical practice.

The present study has focused on the biophysical properties of insulin glargine (i.e., with Cys^A6^ and Cys^A11^; henceforth designated simply “glargine”), an analog in broad clinical use (Figure 1a) (Home and Ashwell 2001). Containing two additional positive charges due to a C‐terminal di‐Arg B‐chain extension (upper panel of Figure 1b), the analog is soluble in an acidic formulation, but undergoes isoelectric precipitation in a subcutaneous depot, thereby providing protracted action (lower panel of Figure 1b and sect. S1.2 of Data S1). Whereas insulin glargine is more susceptible to fibrillation than is wild‐type (WT) insulin, we report that this defect is mitigated in a diselenide analog of glargine (i.e., with Sec^A6^ and Sec^A11^, designated “Se‐glargine”). Protection from fibrillation is associated with damping of conformational fluctuations as probed by a diverse set of assays: susceptibility to protease digestion, temperature‐dependent CD spectra, ^1^H‐NMR secondary shifts, ^1^H‐NMR nuclear Overhauser effects (NOEs), and subglobal ^1^H‐^2^H amide‐proton exchange in D_2_O (for hydrogen exchange formalism, see sect. S2.1 of Data S2). Although the present high‐resolution solution structure of Se‐glargine is essentially identical to that of insulin glargine (its “parent” analog), damping of conformational fluctuations is associated with a prolonged dimer lifetime at high protein concentrations. Our findings are discussed in relation to MD simulations of the A6‐A11 diselenide bridge, which suggest a nonlocal coupling between bridge rigidity, adjoining core packing efficiency and transmitted motions. Together, our results demonstrate that subtle substitution of an internal disulfide bridge in a globular protein by a diselenide bridge damps amyloid‐associated conformational fluctuations without change in overall structure or biological activity. Such protective damping stands in contrast to the modest differences between sulfur and selenium in radius, bond length and valence properties (Figure S7) (Arnér 2010; Mousa et al. 2017; see also sect. S1.3 of Data S1). Nonstandard optimization of core packing—illuminating otherwise cryptic relationships between protein dynamics and amyloidogenesis—promises to enhance real‐world access to an essential therapeutic protein.

## 2 RESULTS

### 2.1 Diselenide bridge augments conformational stability with native activity

Se‐glargine retained nativelike activity as shown by Western‐blot detection of hormone‐stimulated insulin‐receptor phosphorylation and downstream Akt phosphorylation (Figure S8). Susceptibility to fibrillation was evaluated in a 96‐well plate assay (El Hage et al. 2025) wherein protein solutions were continuously agitated at 37°C in the presence of an aggregation‐sensitive fluorescent probe (thioflavin T; ThT). Lag times were defined by fluorescence doubling relative to initial values (Figure 1d). Under assay conditions (10 mM Tris–HCl [pH 4.0] and 140 mM NaCl), glargine and Se‐glargine exhibited respective lag times of 9(±3) hours (h) and 41(±3) h; the latter prolongation was significant (*p* < 0.0001; Figure 1e and Table 1). By contrast, the mean lag times of Se‐glargine and WT insulin (39(±12) h) were indistinguishable (*p* > 0.5). Respective variances were nonetheless distinct (*p* < 0.01); the smaller variance of Se‐glargine may have real‐world implications (see section 3) (Brange et al. 1997). These findings demonstrate that a disulfide‐to‐diselenide substitution can protect a protein analog from accelerated degradation.

**TABLE 1:** Structural and stability parameters of insulin analogs.

Analog	Fibrillation lag time^a^ hours ± SD (*N*)	Pepsin degradation half‐lives^b^ min ± SD	222/208 nm ratio	*dθ*/*dT* ^c^	Far UV‐CD analysis^d^				Δ*G*_u_ ^e^ (kcal/mol)
					α‐helix (%)	β‐sheet (%)	Disordered (%)	Turn (%)	
Human insulin	39 ± 12 (9)	–	0.86	–	55 ± 4	13 ± 3	13 ± 2	19 ± 4	3.8 ± 0.1
Glargine	9 ± 3 (9)	36.0 ± 3.8	0.79	0.036	52 ± 4	16 ± 4	13 ± 3	19 ± 6	2.8 ± 0.1
Se‐glargine	41 ± 3 (7)	61.6 ± 0.3	0.89	0.019	55 ± 8	12 ± 2	11 ± 6	21 ± 1	3.8 ± 0.1

^a^ Lag time refers to the duration required for a two‐fold increase from baseline in ThT fluorescence. The number of replicates is given by (*N*).

^b^ Half‐lives were the average of two independent experiments.

^c^ These data were derived from analysis of the 222 nm band across wavelength experiments conducted between 5°C and 40°C.

^d^ Protein CD analysis (% helix, % sheet, % disordered, and % turn) was done using the CDPro software package (https://sites.google.com/view/sreerama). The reference dataset used was SP43 (soluble protein 43). The numbers are average values from three different programs (SELCON3, CONTINLL, and CDSSTR).

^e^ Data are from two‐state modeling of CD monitored guanidine titrations performed at 25°C and pH 4 (Sosnick et al. 2000; Weil‐Ktorza et al. 2024).

**FIGURE 2:**
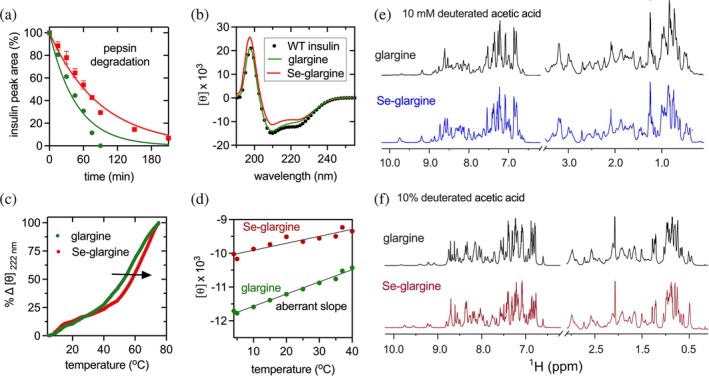
Comparative biochemical and biophysical studies of insulin analogs. (a) Resistance to protease cleavage: HPLC assays probing stabilities of insulin glargine and Se‐glargine to pepsin. (b) Far‐UV CD spectra of insulin analogs. (c) Thermal stability assay monitored by circular dichroism at helix sensitive wavelength (222 nm) shows enhanced stability of Se‐glargine. (d) Temperature‐wavelength scan inferred slopes of glargine and Se‐glargine from 4 to 40°C. (e) 1D ^1^H‐NMR spectra of glargine insulin (top, black) and Se‐glargine (bottom, blue) acquired in 10 mM deuterated acetic acid in H_2_O (10% D_2_O) at pH 3.0 (direct meter reading). (f) 1D ^1^H‐NMR spectra of glargine insulin (top, black) and Se‐glargine (bottom, maroon) acquired in 10% deuterated acetic acid in H_2_O (10% D_2_O) at pH 2.1 (direct meter reading). Spectral line narrowing was observed in the methyl region (upfield) and amide/aromatic regions (downfield) for Se‐glargine. Data were acquired at a ^1^H frequency of 700 MHz at 25°C.

Pepsin digestion at pH 2 was employed to probe stability differences between glargine and Se‐glargine (Figure 2a). Respective rates of disappearance of the principal elution peak (in high‐performance liquid chromatography (HPLC); Figures S9 and S10) implied distinct half‐lives (61.6(±0.3) min versus 36(± 4) min) (Table 1; *p* < 0.05). Se‐glargine's twofold slower rate of digestion (relative to its parent) suggests damping of local conformational fluctuations required for the substrate to access the enzyme's active site. Conformational stability was further probed by far‐ultraviolet (UV) CD to assess secondary structure: spectra from 190 to 255 nm revealed qualitative differences (Figure 2b). Whereas helix‐associated ellipticities at 222 nm were similar, Se‐glargine exhibited an attenuated 208‐nm signal relative to glargine, thereby increasing the 222/208‐nm ratio (0.89 vs. 0.79; Table 1); the 222/208‐nm ratio of WT insulin is similar to that of Se‐glargine (0.86 vs. 0.89). Deconvolution of CD spectra nonetheless indicated similar α‐helical contents (Tables 1 and S2) (Sreerama and Woody 2000); the inferred helix content of glargine was slightly lower than that of the other proteins. Differences in stability were further probed based on temperature‐dependent attenuation of ellipticity at 222 nm (Figure 2c): Se‐glargine more effectively resisted thermal unfolding (arrow). To gain further insight, we acquired CD spectra between 4 and 40°C (Figure S11a–c). As shown in Figure 2d, the 222‐nm signal from glargine exhibited a steeper temperature dependence than did Se‐glargine (corresponding to cumulative attenuation between 4 and 40°C (Δ[*θ*]) of 1.28 versus 0.68 deg·cm^2^·dmol^−1^; Figure S11d). Such attenuation presumably reflects progressive segmental disorder among one or more helical segments.

To obtain residue‐specific probes and hence distinguish between the domain's three α‐helices, we undertook an ^1^H‐NMR study. Spectra of glargine and Se‐glargine were first obtained at pH 3.0 in 10 mM deuterio‐acetic acid (dAA) in H_2_O (10% D_2_O). Protein concentrations were 2.4 times higher than those employed in CD. One‐dimensional (1D) spectra of glargine (top, black) and Se‐glargine (bottom, blue) are shown in Figure 2e; overall dispersion is similar as illustrated by the downfield amide‐aromatic and upfield aliphatic regions (left and right panels, respectively). The spectra differ in detail. As observed in past ^1^H‐NMR studies of insulin and insulin analogs (Weiss et al. 1989), amide resonances in each case exhibited anomalous conformational broadening as illustrated by resolved downfield resonances (assigned to residues Cys^A11^ or Sec^A11^, Leu^B6^, Gly^B8^, and Ser^B9^ below). Such broadening has been ascribed to (i) intermediate exchange between monomers and dimers (Cheshoversusky et al. 1983) and (ii) millisecond motions in the monomer (Weiss et al. 1989). To circumvent this technical barrier, ^1^H‐NMR spectra were also acquired in 10% dAA in H_2_O (10% D_2_O) at pH 2.1. As shown in Figure 2f, introduction of an organic co‐solvent resulted in overall resonance narrowing in accordance with past studies (Kline and Justice Jr 1990; Weiss et al. 1989); an organic co‐solvent both weakens dimerization and enhances conformational averaging of chemical shifts.

### 2.2 Diselenide bridge enhances ^1^H‐NMR spectral quality

**FIGURE 3:**
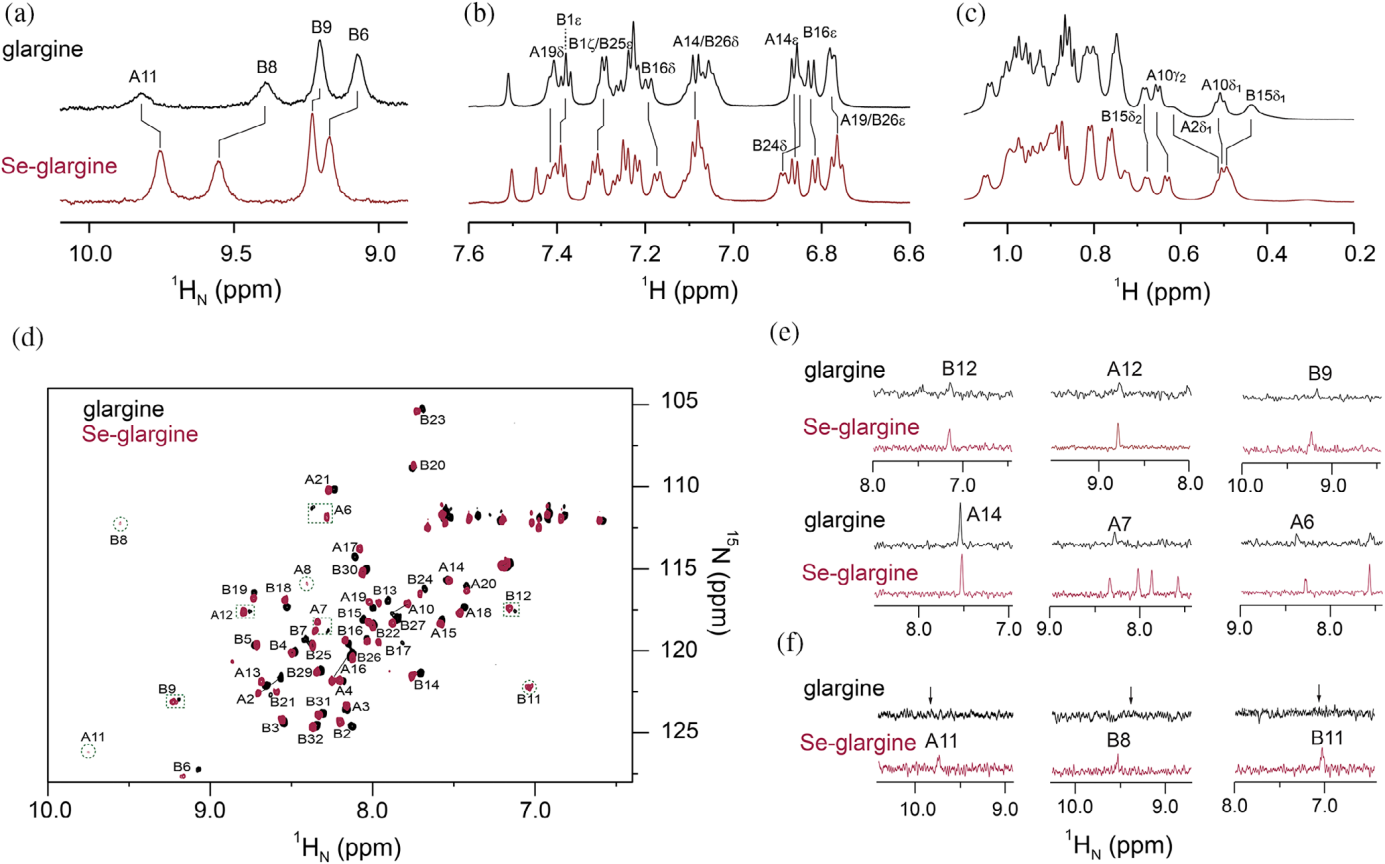
Internal diselenide bridge mitigates conformational broadening of ^1^H‐NMR resonances. (a) The most downfield region of the 1D ^1^H‐NMR spectra of glargine insulin (top, black) and Se‐glargine (bottom, maroon) acquired in 10% deuterated acetic acid in H_2_O (10% D_2_O) at pH 2.1 (direct meter reading). The well‐resolved amide resonances are indicated on the top: Cys^A11^/Sec^A11^, Gly^B8^, Ser^B9^, and Leu^B6^. Subsequent panels show aromatic (b) and methyl region (c) in the 1D ^1^H‐NMR spectra. The assigned aromatic signals and well‐resolved methyl resonances are labeled. Spectral line narrowing was observed in the methyl and amide/aromatic regions in the Se‐glargine spectra. (d) Overlay of the natural abundance ^1^H‐^15^N HSQC spectra. Main‐chain resonance assignments are indicated. Dashed circles indicate backbone cross peaks that were not observed in glargine. Those cross peaks were only visualized in the lower contour level in Se‐glargine. Dashed boxes revealed cross peaks that were much stronger in Se‐glargine. (e) Spectral trace of the dashed‐box cross peaks in 2D ^1^H‐^15^N HSQC spectra. Intensities of traced peaks were normalized based on the intensity of Tyr^A14^ residue. (f) Spectral trace of the dashed‐circle cross peaks in 2D ^1^H‐^15^N HSQC spectra. The trace spectra of glargine were derived based on the ^15^N chemical shift of Se‐glargine due to missing cross peaks of glargine. Arrows indicate amide proton chemical shift of corresponding residues. The spectral intensity was increased three times in comparison to that in panel (e). Selenium replacements at A6 and A11 attenuate conformational exchange of the A6–A11 bridge, which in turn limits excursions of the A1–A8 and B6–B12 segments.

Comparison of corresponding 1D ^1^H‐NMR spectra of glargine and Se‐glargine in 10 mM dAA (black vs. blue in Figure 2e) and in 10% dAA (black vs. maroon in Figure 2f) suggests that, in either solvent, the degree of resonance broadening is more marked in glargine. This impression is corroborated by expansion of spectral regions in 10% dAA containing resolved resonances: downfield amide resonances in H_2_O (Figure 3a), aromatic resonances in D_2_O (Figure 3b), and aliphatic resonances in D_2_O (Figure 3c). As indicated in the panels by vertical lines, diselenide‐associated sharpening is accompanied in each region by changes in chemical shifts. Although similar qualitative trends occurred in 10 mM dAA, the co‐solvent enabled complete resonance assignment through 2D homonuclear ^1^H‐NMR spectra (Figure S12; Table S3); ambiguities were resolved through natural‐abundance ^1^H‐^15^N and ^1^H‐^13^C heteronuclear single‐quantum coherence (HSQC) spectra (Figures 3d and S13, respectively).

Of particular interest are the line widths and chemical shifts of resolved downfield amide resonances (assigned to Cys^A11^ or Sec^A11^, Gly^B8^, Ser^B9^, and Leu^B6^; Figure 3a), sites that are fortuitously near the paired sites of Cys → Sec substitution (Figure S14a). These resonances in the spectrum of Se‐glargine (bottom of Figure 3a) were much narrower than that of glargine (Cys^A11^, Gly^B8^, Ser^B9^, and Leu^B6^; top of Figure 3a). Analysis of aromatic chemical shifts indicates that such differences are widely distributed in the protein (Tyr^B16^, Phe^B24^, Tyr^B26^, and Tyr^A19^; Figure 3b). A combination of local and nonlocal perturbations was also observed in the upfield aliphatic region as indicated by the upfield methyl resonances of Ile^A2^, Ile^A10^, and Leu^B15^ (Figures 3c and S14b). An overview of chemical‐shift changes is provided by an overlay of natural‐abundance ^1^H‐^15^N HSQC spectra (Figure 3d). Under these conditions, a subset of fingerprint ^1^H‐^15^N cross peaks in the spectra of both Se‐glargine and its parent are attenuated by resonance broadening, but to different extents. Shown in dashed circles in Figure 3d are ^1^H‐^15^N cross peaks observed only in Se‐glargine, and in dashed boxes cross peaks stronger in Se‐glargine than glargine; representative ω_2_ traces are shown in Figure 3e,f. There were by contrast no ^1^H‐^15^N cross peaks observed only in the spectrum of glargine. Together, these observations provide evidence that the diselenide substitution damps millisecond motions responsible for the more extensive conformational broadening in the parent spectrum. These comparisons are further detailed in Figures 3d‐f traces taken through representative cross peaks. A control was provided by Tyr^A14^ on the protein surface, which exhibits motional narrowing (Weiss et al. 1989). The A14 ^1^H‐^15^N cross peak has essentially the same line width and chemical shift in glargine and Se‐glargine.

**FIGURE 4:**
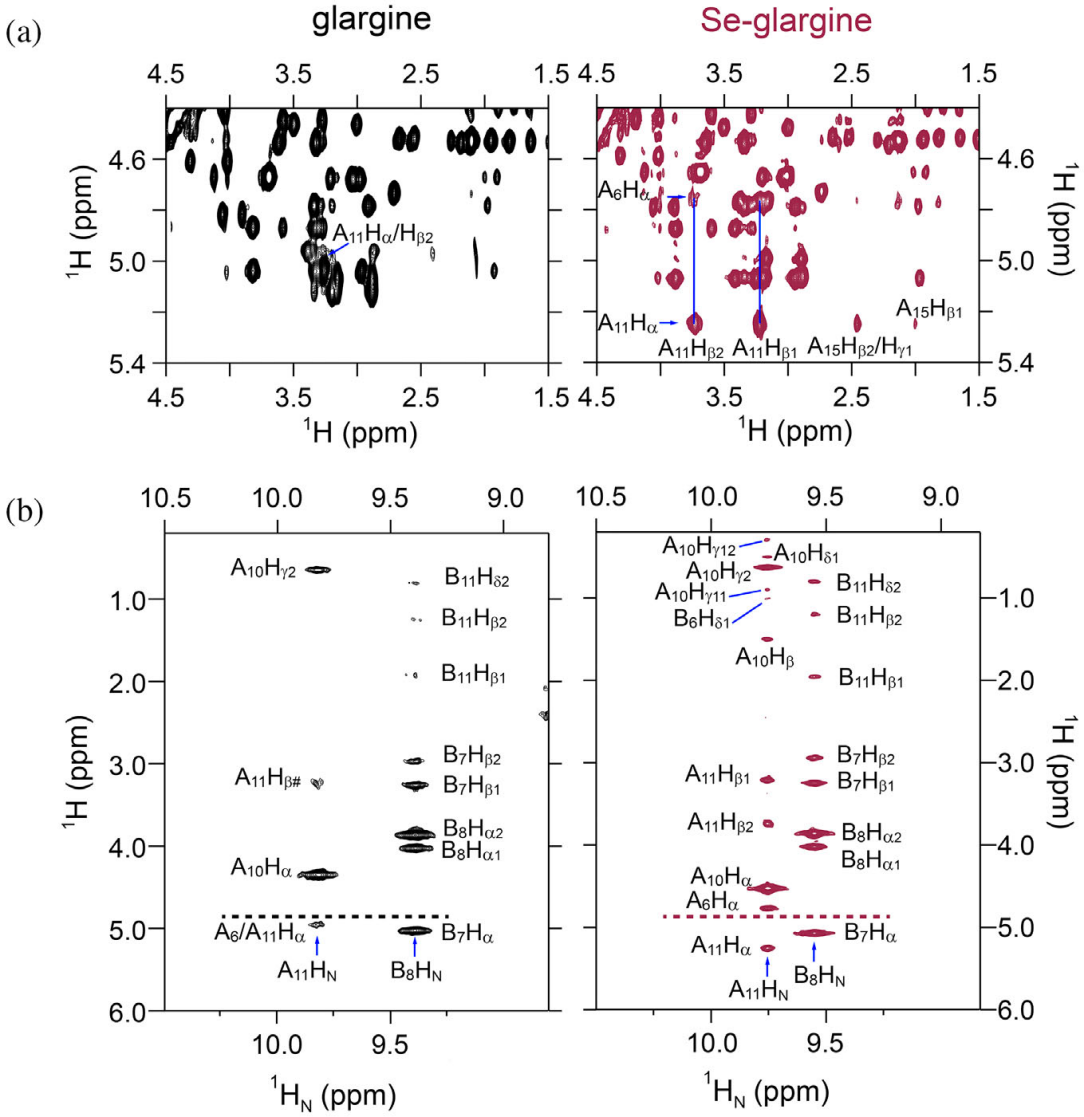
Diselenide bridge is associated with enhanced main‐chain NOE density. (a, b) NOESY spectra of glargine (left, black) and Se‐glargine (right, maroon): (a) spectral region showing Cys^A11^/Sec^A11^ H_α_‐related NOEs. Due to exchange line broadening and chemical shift degeneracy, only Cys^A11^ H_α_/H_β2_ NOE cross peak was clearly observed in the NOESY spectrum of glargine. Conversely, four Sec^A11^ H_α_‐related NOEs (Sec^A11^H_α_/H_β1,2_ and Sec^A11^H_α_/Gln^A15^H_β1,2_) and two Sec^A6^ H_α_‐related (Sec^A6^H_α_/A11H_β1_ and Sec^A6^H_α_/A11H_β2_) were observed in the NOESY spectrum of Se‐glargine. Additional NOEs were also observed for Sec^A11^ H_β_‐protons. (b) Spectral region showing Cys^A11^/Sec^A11^ H_N_‐related NOEs. Only Cys^A11^ H_β2_ NOE cross peaks were clearly visualized in the spectrum of glargine (left panel, black), the Cys^A11^ H_β1_‐related NOE cross peaks were not visualized due to exchange line broadening. Similarly, more Sec^A11^ H_N_‐related NOEs were observed in the NOESY spectrum of Se‐glargine. Eleven Sec^A11^ H_N_‐related NOEs (right panel, maroon) were observed, whereas only four Cys^A11^ H_N_‐related NOEs (left panel, black) were obtained. The cross‐peak line width and intensity was also improved in the NOESY spectrum of Se‐glargine. Dashed lines indicate the position of water peaks. The NOESY mixing time in each case was 150 ms.

The A6‐A11 diselenide substitution was also associated with an increased density of inter‐residue nuclear Overhauser effects (NOEs) relative to glargine (Figures 4 and S15a,b). There are 62 NOEs observed in the NOESY spectrum of Se‐glargine, but not in that of glargine (Table S4). Of these, 45 relate to either (a) the A2‐A8 helical segment (16 NOEs), (b) the A10‐A12 segment (13 NOEs), (c) residues in contact with Sec^A11^ (residues A15, A16, B6, and B11; 10 NOEs), or (d) residues in the next shell of side chains (B12 and B15; 6 NOEs). By contrast, only 10 NOEs were observed in the NOESY spectrum of glargine, but not in that of Se‐glargine (Table S4). Such differences, in either direction, are unlikely to represent marked structural changes in side‐chain relationships. Instead, the observability or prominence of corresponding NOEs in the two spectra was generally influenced by degree of resonance broadening and resonance overlap. This technical distinction is illustrated in (i) Figure 4a with respect to the H_α_ and H_β_ resonances of Sec^A11^ versus Cys^A11^ and (ii) Figure 4b with respect to the amide resonances of Sec^A11^/Cys^A11^ and Gly^B8^ (Figure S15c).

### 2.3 Se‐glargine exhibits nativelike structure with distributed chemical‐shift changes

The solution structure of Se‐glargine was determined in 10% dAA (pH 2.1) based on hybrid distance‐geometry/dynamic simulated annealing (Nilges et al. 1988; Omichinski et al. 1990; Schwieters et al. 2003; Schwieters et al. 2006); a control structure of its parent was likewise determined. Overall NOE patterns were in each case similar (Table S4) to those observed at neutral pH in ^1^H‐NMR studies of an engineered insulin monomer (Hua et al. 1996) and related monomeric analogs (Rege et al. 2020). Respective structures were defined by 1284 distance restraints (Se‐glargine; 24 restraints per residue) or 1237 distance restraints (glargine; 23 restraints per residue). For Se‐glargine, the root‐mean‐square difference (RMSD) among main‐chain atoms is 0.35(±0.11) Å and among all heavy atoms is 0.77(±0.11) Å. For glargine, respective main‐chain and all‐atom RMSD values are 0.34(±0.13) Å and 0.65(±0.15) Å. Statistics are given in Tables S5 and S6.

**FIGURE 5:**
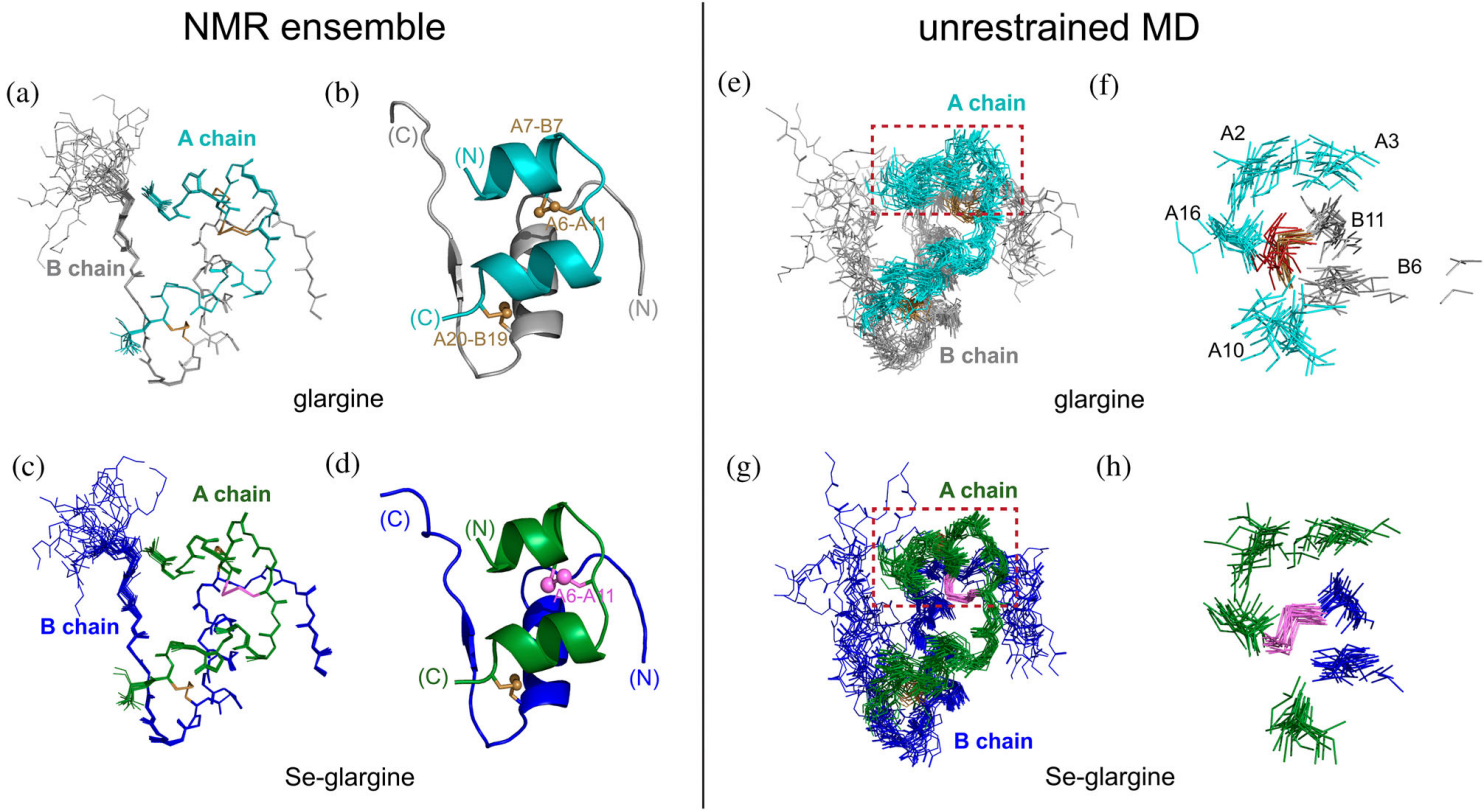
Structural comparison of glargine and Se‐glargine. Left panels: superposition of 20 NMR‐derived structures and corresponding ribbon models. (a) *Glargine*. The A chain is shown in cyan, the B chain in gray, and disulfide bridges in gold. Structures were aligned according to the main‐chain atoms of residues A1–A21 and B3–B28. The backbone RMSD is 0.34 ± 0.13 Å, and the all‐heavy‐atom RMSD is 0.65 ± 0.15 Å. (b) Ribbon structure of glargine. (c) *Se‐glargine*. The A chain is shown in green, the B chain in blue, disulfide bridges in gold, and the A6‐A11 diselenide bridge in violet. The ensemble was aligned as above. The backbone RMSD is 0.35 ± 0.11 Å, and the all‐heavy‐atom RMSD is 0.77 ± 0.11 Å. (d) Ribbon representation of Se‐glargine. The conserved core‐packing schemes of stereo representation and representative ball‐and‐stick model are shown in Figure S16. Right panels: superposition of unrestrained MD‐derived structures and local environment surrounding the A6–A11 bridge. (e) Superposition of 20 glargine structures obtained from unrestrained MD simulations across five replicates (*r*_1_–*r*_5_, one structure per 100 ns). Structures were aligned using the main‐chain atoms of residues A2–A20 and B10–B19 (core α‐helix region). The red dashed box highlights the comparatively less‐ordered A1–A8 α‐helix and disulfide bridge region relative to Se‐glargine. The backbone RMSD is 1.37 ± 0.57 Å, and the all‐heavy‐atom RMSD is 2.18 ± 0.59 Å. Color scheme matches panel (a). (f) Expanded view of side‐chain packing around the A6–A11 disulfide bridge. The disulfide bridge conformations resembling those of Se‐glargine are shown in gold; all others are shown in red. The backbone RMSD of those residues is 1.57 ± 0.73 Å, and the all‐heavy‐atom RMSD is 2.10 ± 0.82 Å. (g) Superposition of 20 Se‐glargine MD structures aligned according to the main‐chain atoms of residues A2–A20 and B10–B19. The backbone RMSD is 1.00 ± 0.32 Å, and the all‐heavy‐atom RMSD is 1.76 ± 0.41 Å. Color scheme matches panel (c). (h) Expanded view of side‐chain packing surrounding the A6–A11 diselenide bridge. The backbone RMSD of those residues is 0.90 ± 0.33 Å, and the all‐heavy‐atom RMSD is 1.45 ± 0.42 Å.

Solution structures are shown in Figure 5a–d; structural comparisons are further provided in Figures S16 and S17. Analyses of cross violations (i.e., consistency between the distance restraints for Se‐glargine and the structure of glargine, and vice versa) are given in Table S7. As expected, Se‐glargine (Figure 5c,d) contains a well‐ordered α‐helical domain that is similar to corresponding portions of crystallographic T‐state protomers of porcine insulin (Baker et al. 1988) and glargine (Protein Data Bank entry 5VIZ). Non‐helical B‐chain residues B24–B26 form a well‐defined β‐strand as in the solution structure of WT insulin at neutral pH (Rege et al. 2020). Whereas the N‐terminal arm of the B chain (residues B1–B4) is also well defined, its C‐terminal residues (B27–B32) are less well defined, presumably due to intrinsic disorder associated with motional narrowing and absence of long‐range NOEs. Only subtle differences are observed between the corresponding core‐packing schemes of Se‐glargine and its parent.

**FIGURE 6:**
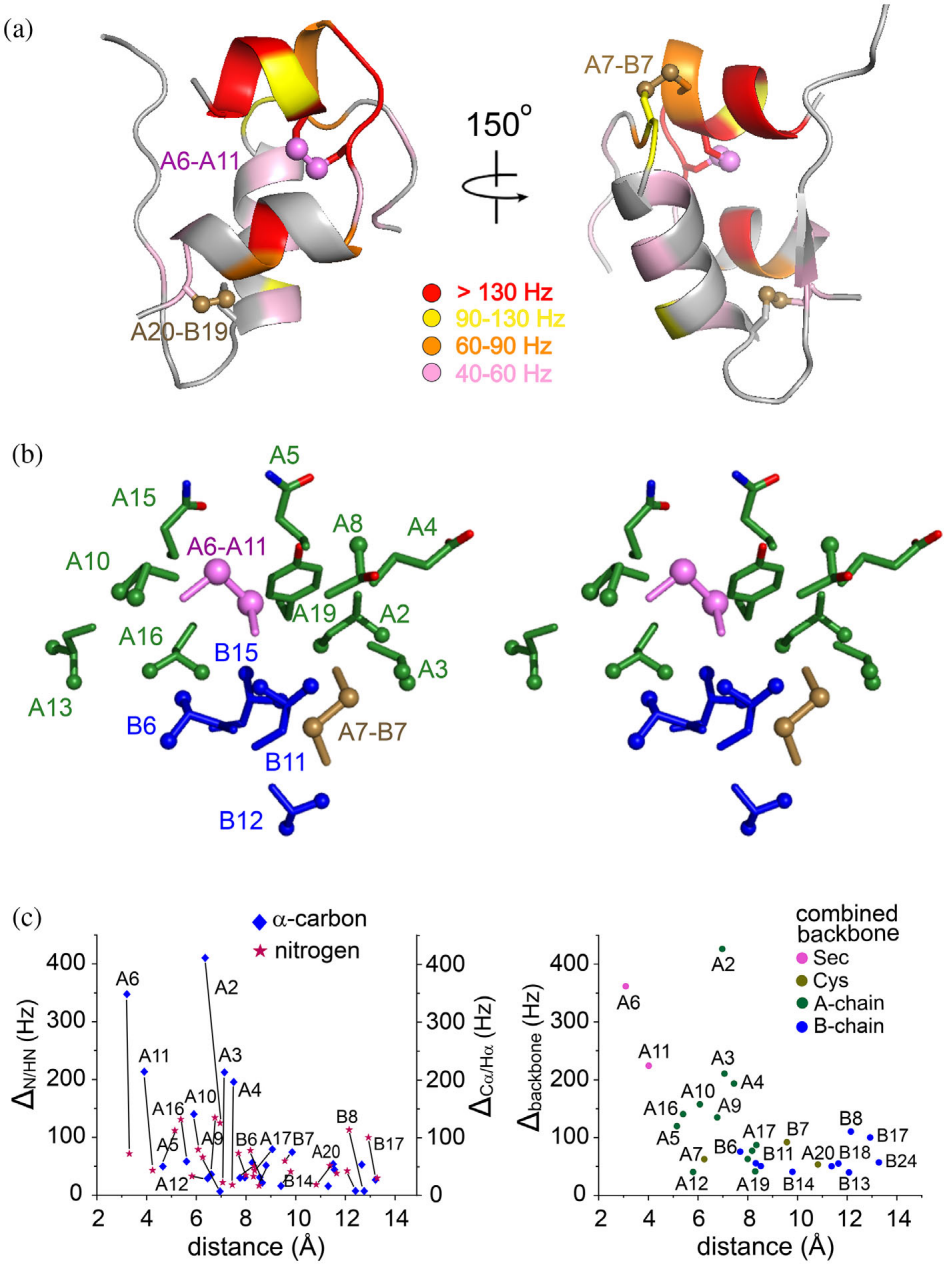
Chemical‐shift differences between glargine and Se‐glargine. (a) Color‐coded ribbon representation of Se‐glargine showing combined backbone chemical‐shift changes induced by the A6–A11 diselenide bond. Chemical shifts are given in Table S3. Color coding for Δ_backbone_; red: >130 Hz, yellow: 90–130 Hz, orange: 60–90 Hz, light pink: 40–60 Hz, Gray: <40. (b) Stick model of residues near the A6–A11 diselenide bond in Se‐glargine exhibiting side‐chain chemical shift perturbation >40 Hz (Table S8). A‐chain residues are shown in green and B‐chain residues in blue. Sulfur atoms and methyl groups are shown as spheres. (c) Chemical shift perturbation in relation to atomic distance from A6–A11 diselenide bridge. Left: Combined ^15^N/^1^H_N_ or ^13^C_α_/^1^H_α_ perturbations plotted against average distance from A6/A11 selenium atoms to backbone nitrogen or α‐carbon. Right: Combined backbone perturbations plotted against average distance from A6/A11 selenium atoms to backbone nitrogen and α‐carbon.

Despite the similarity of their solution structures, Se‐glargine exhibits widespread changes in ^1^H, ^13^C, and ^15^N chemical shifts. Shown in Figure 6a is a ribbon representation of main‐chain chemical‐shift differences, color‐coded by extent of perturbation. The largest changes in main‐chain chemical shifts occur in the A‐chain N‐terminal α‐helix (residues A2–A8) and in residues adjoining Sec^A11^ in sequence (A9–A11) or structure (core residues Leu^A16^ and Leu^B11^). Less marked chemical‐shift perturbations occur throughout the well‐ordered portions of the structure. Mapped in Figure 6b (and summarized in Table S8) are corresponding side‐chain chemical‐shift perturbations >40 Hz. Subtle reorganization of the hydrophobic core adjoining the A6–A11 diselenide bridge (Figures S14b and S18a) and of neighboring inter‐chain hydrogen bonds (Figure S14a) presumably underlies transmission of chemical‐shift perturbations to distant sites (see sections 3 and 3.3, and sect. S1.4 of Data S1). The relationship between chemical‐shift perturbations and distance from the A6–A11 diselenide bridge is shown in Figure 6c and elaborated in Figure S18b.

### 2.4 Diselenide bridge damps monomer–dimer exchange

In 10 mM dAA (pH 3.0) and 25°C Se‐glargine and its parent are predominantly dimeric in the protein concentration range 60–200 μM, but in slow exchange with a monomeric subpopulation. Such exchange is in accordance with past studies of WT insulin under similar conditions (Hua and Weiss 1990). Respective lifetimes of the dimer provide an intrinsic probe for the dynamics of the dimer interface (Figure S19). Since this interface (comprising the central B‐chain α‐helix and C‐terminal β‐strand as in T_2_ and T_6_ crystal structures; Blundell et al. 1972; Gursky et al. 1992) does not directly involve the A6–A11 bridge (Baker et al. 1988), potential changes to the kinetics of monomer–dimer exchange reflect transmitted effects of the diselenide substitution on the structure or dynamics of the interface. No exchange cross peaks were observed in 10% dAA.

**FIGURE 7:**
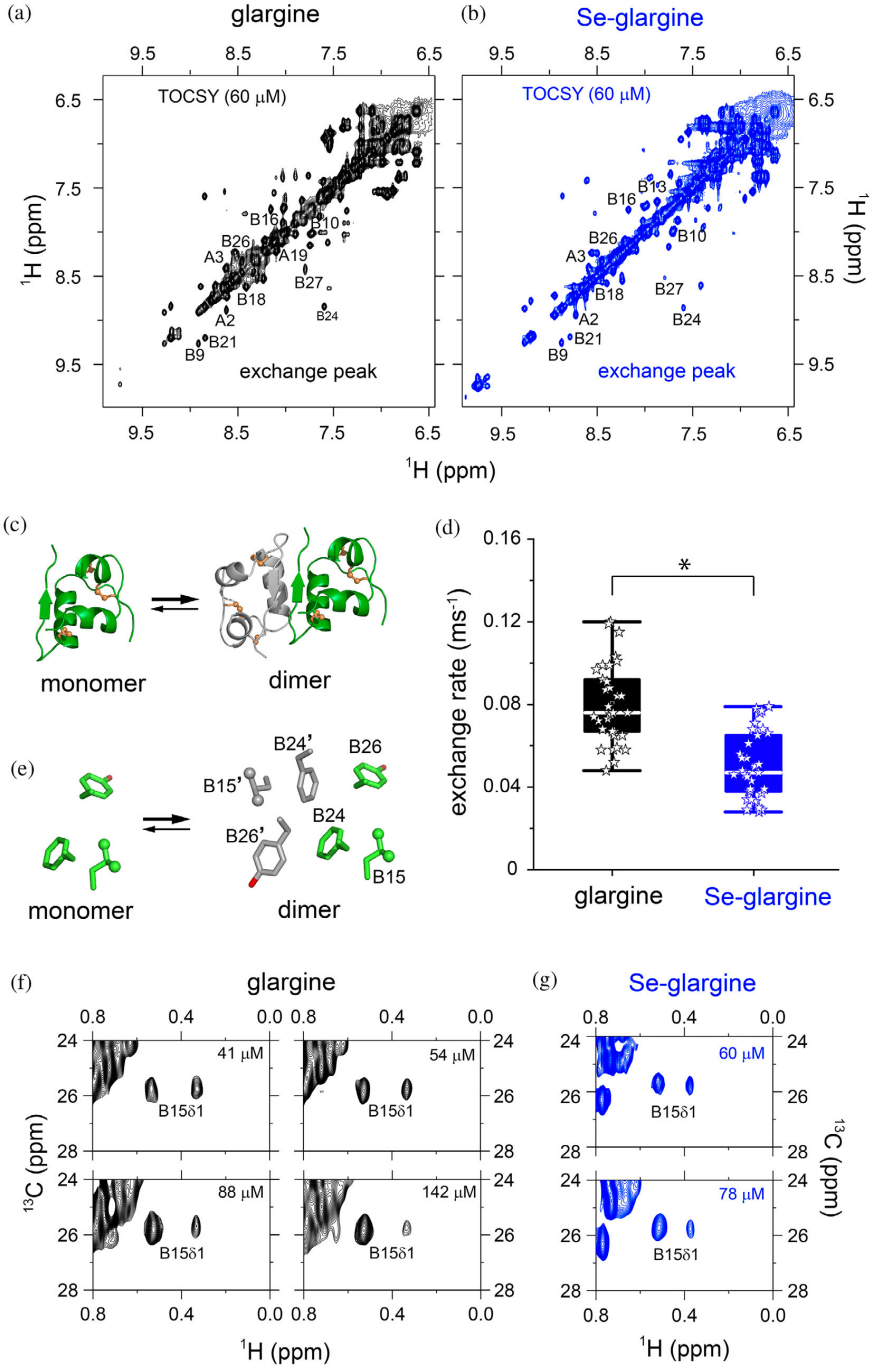
Comparative monomer–dimer equilibrium. (a, b) Respective NMR evidence of monomer–dimer equilibrium of glargine (a) and Se‐glargine (b) at low protein concentration (<150 μM). TOCSY spectra of glargine (black) and Se‐glargine (blue) were acquired in 10 mM deuterated acetic acid (pH 3.0, direct meter reading) at 25°C. The protein concentration was ~60 μM. Dimer‐related exchange cross‐peaks among amide resonances were observed as indicated. (c) Ribbon model of insulin monomer–dimer exchange (PDB code: 4INS). One protomer in the dimer is highlighted in gray and another one in green. (d) Monomer–dimer exchange rate for glargine (black) and Se‐glargine (blue) in 10 mM deuterated acetic acid (pH 3.0, direct meter reading) at 25°C. Statistical significance at the level *p* < 0.01 (Wilcoxon test) for monomer–dimer exchange measured by NMR exchange spectroscopy (*). Exchange rates are given in Table S9. (e) The environment of methyl groups of Leu^B15^ residue in the monomer and dimer of insulin. The color code is the same as panel (c). (f) Expanded regions of ^1^H, ^13^C‐HSQC spectra of glargine in methyl region acquired at concentrations of 142, 88, 54, and 41 μM. The Leu^B15^ δ_1_‐CH_3_ resonance varied in chemical shift due to differences in aromatic ring‐current effects in the monomer and in the dimer. The doublet of Leu^B15^ δ1‐CH_3_ provided direct evidence of monomer–dimer equilibrium and demonstrated concentration dependence of the monomer–dimer ratio at low protein concentration. (g) Expanded regions of ^1^H, ^13^C‐HSQC spectra of Se‐glargine in methyl region. Spectra were acquired at a ^1^H frequency of 700 MHz in 10 mM deuterated acetic acid (pH 3.0, direct meter reading) at 25°C. The corresponding monomer–dimer ratios and dimerization constants are given in Table S10.

Evidence for dimerization (slow on the time scale of NMR chemical shifts) was provided by corresponding exchange cross peaks in TOCSY and NOESY spectra. Such exchange is particularly clear in the amide region of the spectra in H_2_O (Figures 7 and S20a,b); its pattern is similar in the two analogs. Whereas NOEs between amide resonances typically occur within α‐helices, in the absence of slow exchange no TOCSY cross peaks would be observed (due to negligible *J*_NN_ coupling constants). Residue‐specific assignment of these exchange cross peaks, established by combined use of homonuclear 2D spectra and natural‐abundance ^1^H‐^13^C HSQC spectra, is shown in Figure 7a,b (see also Figures S20 and S21); an example is provided by the discrete monomer‐ and dimer‐specific amide resonances of Glu^B21^ (adjoining but not within the dimer interface; Figure S19) as highlighted in Figure S20c. In the solution structures of glargine and Se‐glargine, respective A6–A11 bridges buttress core side chains Leu^B6^, Leu^B11^, Ile^A2^, and Leu^A16^, which in turn underlie the side chains of Leu^B15^, Phe^B24^, and Tyr^B26^ at the dimer interface (Figure S22). These structural relationships presumably provide a pathway for transmission of conformational adjustments and associated changes in protein dynamics.

Quantitative analyses of monomer–dimer exchange (Figure 7c) provided insight into transmitted effects of the diselenide bridge. Two approaches were undertaken: (a) first, to estimate respective dimer lifetimes, exchange peaks were integrated as a function of mixing time (Figures 7d and S23). Exchange rates are given in Table S9. Corresponding lifetimes are 12.3(±2.6) ms (glargine) and 17.5(±3.8) ms (Se‐glargine); this difference achieved statistical significance (*p* < 0.0001; Wilcoxon test). (b) Next, to estimate respective dissociation constants, we exploited the distinct upfield ^1^H‐NMR chemical shifts of Leu^B15^ methyl groups (Figure 7e). These shifts reflect aromatic ring currents both within the monomer and at the dimer interface (Jacoby et al. 1996). An expanded region of the natural‐abundance ^1^H‐^13^C HSQC spectrum containing ^1^H‐^13^C methyl cross peaks of Leu^B15^ is shown in Figure 7f (black, glargine) and Figure 7g (blue, Se‐glargine). In each case the pair of Leu^B15^ δ_1_‐CH_3_ cross peaks reflects the monomer–dimer equilibrium: their protein concentration‐dependent ratio provided estimates of respective equilibrium constants (Table S10): 16.5(±4.4) μM (glargine) and 14.1(±1.2) μM (Se‐glargine). These estimates are not statistically distinguishable. A slight strengthening of the Se‐glargine dimer is possible but insufficient to account for its longer lifetime. Together, these findings provide evidence that the diselenide bridge hinders dimer dissociation as a transmitted dynamic effect.

### 2.5 Diselenide bridge accentuates α‐helical chemical shifts and damps ^1^H‐ ^2^H exchange

The residue whose ^1^H‐^13^C‐^15^N chemical shifts are most altered by the diselenide substitution is Ile^A2^ (Figures 6 and S22), an invariant aliphatic residue that pins the A1–A8 α‐helix to the hydrophobic core (Figure S24a). Main‐chain and side‐chain chemical shifts of Ile^A2^ (as assigned in 10% dAA) are given in Table S11 together with corresponding secondary shifts (calculated relative to standard random‐coil values; Wishart et al. 1995; Wang and Jardetzky 2002). A subtle trend is observed wherein secondary shifts are larger in magnitude in Se‐glargine than in its parent (Figure S24b,c). The pattern of main‐chain chemical shifts suggests that the diselenide bridge stabilizes a local α‐helical conformation (Table S11), whereas the pattern of methyl chemical shifts suggests enhanced aromatic ring‐current shifts (Jacoby et al. 1996). Since the diselenide substitution does not significantly alter the *mean positions* of neighboring aromatic rings (principally Phe^B24^, Tyr^B26^, and Tyr^A19^), it is possible that in glargine the dynamics of core packing leads to more effective averaging of ring currents due to fluctuations in the distance and orientation of aromatic rings relative to the A2 side chain.

The above features of Ile^A2^ secondary shifts extend to other sites in Se‐glargine (Table S12). Examples are provided by residues Gln^A5^, Leu^A16^, and Leu^B15^, respective probes within each of insulin's three α‐helices. In each case the diselenide bridge accentuates the α‐helical character of signature ^13^C_α_, ^1^H_α_, and ^1^H_N_ chemical shifts. Whereas the Ile^A2^ spin system was not well characterized in 10 mM dAA, fortuitously the spin systems of Gln^A5^, Leu^A16^, and Leu^B15^ could be assigned under these conditions: these spin systems exhibit a similar α‐helical trend (Tables S13 and S14 and Figure S24b,c). Since the solution structures of Se‐glargine and its parent contain identical α‐helical endpoints, we envision that the diselenide bridge damps conformational fluctuations within helical segments that otherwise attenuate canonical α‐helical chemical shifts. This interpretation is in accordance with the above CD findings (Table 1).

**FIGURE 8:**
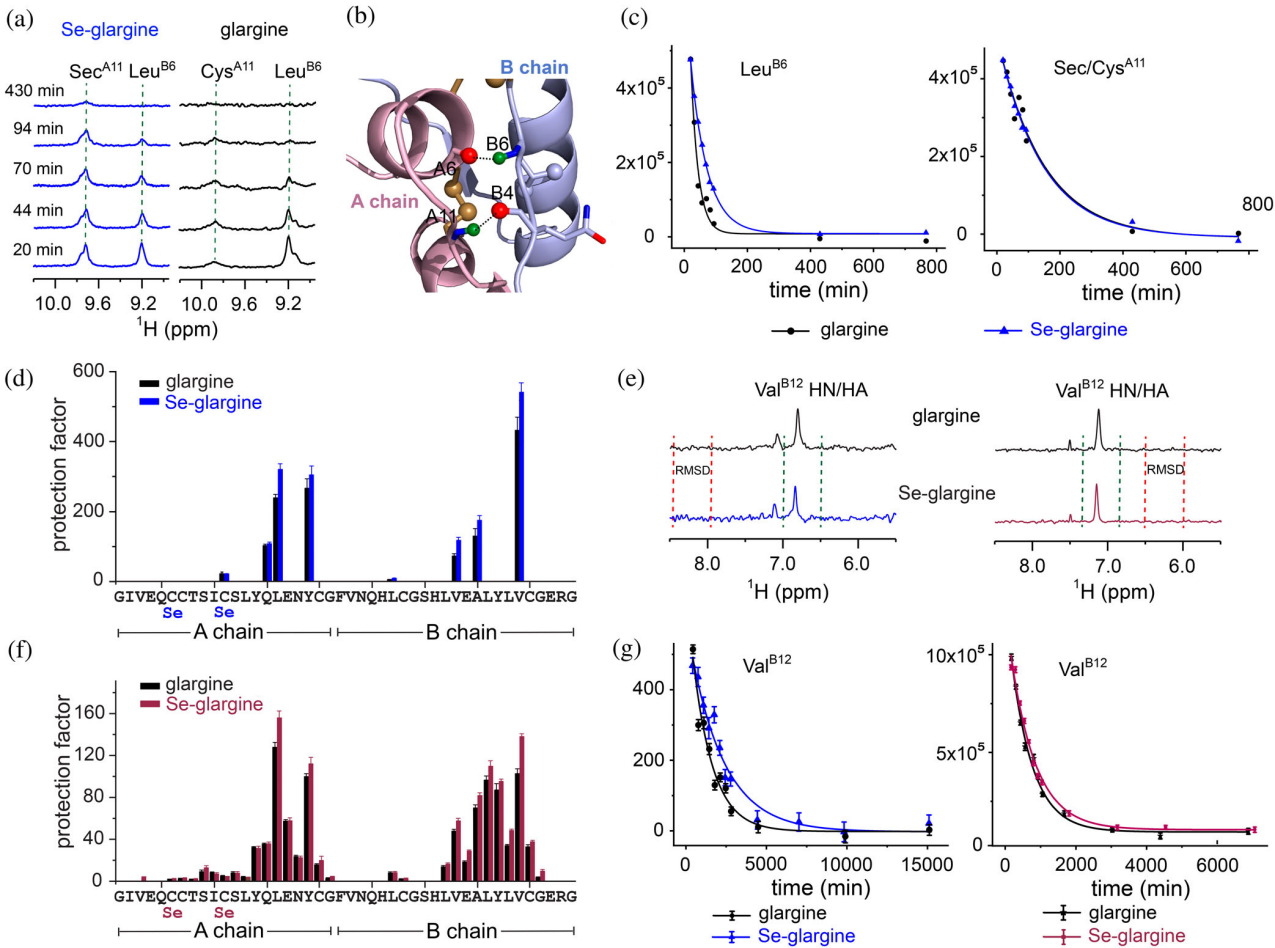
Hydrogen‐deuterium amide‐proton exchange studies. (a) Successive 1D ^1^H NMR spectra of glargine (right panel, black) and A6–A11 diselenide analog (left panel, blue) in the far downfield region at the stated time points after dissolving the protein in 100% D_2_O in 10 mM deuterated acetic acid (pD 3.0, direct meter reading) at 25°C. (b) Structure representation of A6–A11 bridge‐related hydrogen bond (PDB code 4INS). The A chain is in light pink, B chain in light blue and A6–A11 disulfide bridge in gold. The amide protons of A11 and B6 are shown as green spheres, the oxygen atoms of B4 and A6 as red spheres. Dashed lines indicate hydrogen bonds between A11‐HN and B4‐CO and between B6‐HN and A6‐CO. (c) The 1D data fitting curves of amide‐proton ^1^H‐^2^H exchange for Leu^B6^ (left panel) and Sec/Cys^A11^ (right panel) residues in 10 mM deuterio‐acetic acid at pH 3.0 at 25°C. (d) Residue‐specific protection factors (PFs) in glargine insulin (left bars, black) and A6–A11 Se‐glargine (right bars, blue) based on amide‐proton ^1^H‐^2^H exchange monitored by 1D proton and 2D‐TOCSY spectra in 10 mM deuterated acetic acid at pH 3.0 and at 25°C. (e) The spectral trace of 2D‐TOCSY after adding fresh D_2_O for ^1^H‐^2^H exchange for Val^B12^ H_N_/H_α_ cross‐peak in 10 mM deuterated acetic acid at pH 3.0 (left panel) and in 10% deuterated acetic acid at pH 2.1 (right panel). The spectra of glargine are displayed in the top panel and that of Se‐glargine in bottom panel. The spectral RMSD noise level was measured between red dashed lines. (f) Protection factors (PFs) in glargine (left bars, black) and A6–A11 Se‐glargine (right bars, maroon) monitored by 1D proton and 2D‐TOCSY spectra in 10% deuterated acetic acid at pH 2.1 at 25°C. (g) The data fitting curves of amide‐proton ^1^H‐^2^H exchange for Val^B12^ residue in 10 mM deuterio‐acetic acid at pH 3.0 (left panel) and in 10% deuterated acetic acid at pH 2.1 (right panel) at 25°C.

Effects of the diselenide substitution on conformational fluctuations were independently probed by ^1^H‐^2^H amide‐proton exchange in D_2_O (Hua et al. 2011). Whereas conformational averaging of chemical shifts occurs on the submillisecond time scale, amide proton exchange under acidic conditions occurs on a time scale of minutes to hours (Englander et al. 1996; Wuthrich and Wagner 1979). Our studies focused on subglobal exchange, i.e., fluctuations within the native‐state ensemble not dependent on global unfolding. ^1^H‐^2^H exchange kinetics in D_2_O were first monitored in 10 mM dAA (pH 3.0) via serial 1D ^1^H‐NMR spectra and 2D TOCSY. In Figure 8a are shown successive 1D spectra of glargine (right, black) and Se‐glargine (left, blue) in the far downfield region at the indicated time points. Despite the broad line widths of the resolved amide resonances (and their small shoulders), selected ^1^H‐^2^H exchange rates and associated protection factors (PFs) could be extracted (Table S15). Of particular interest are exchange rates at B6 and A11, as these positions provide direct probes of respective bridge‐related hydrogen bonds Leu^B6^‐H_N_^…^O=C‐Sec^A6^ and Sec^A11^‐H_N_^…^O=C‐Gln^B4^ (Figure 8b). The diselenide substitution retards exchange at B6 (Figure 8c, left) whereas exchange at A11 is unaffected (Figure S25). The B6 PF is twice as large in Se‐glargine as in its parent (6.5(±0.2) vs. 2.9(±0.4), respectively); the A11 PFs are indistinguishable (18.9(±0.2) vs. 19.5(±4.5)). Further analysis of ^1^H‐^2^H exchange was not feasible in 10 mM dAA due to the monomer–dimer equilibrium, chemical‐shift degeneracy, and spectral overlap.

To provide a complete overview of ^1^H‐^2^H exchange, the NMR studies were repeated in 10% dAA. Although the co‐solvent weakens hydrophobic interactions (both at the dimer interface and within the core of the monomer), the tractability of the respective ^1^H‐NMR spectra enabled essentially complete analysis at sites of main‐chain hydrogen bonds (Figure S25). Exchange rates and PFs are given in Table S16 (the latter assume that the co‐solvent does not influence random‐coil exchange rates). A trend toward larger PFs in Se‐glargine was observed, both within the A2–A8 α‐helix and in the B chain (Figures 8f and S26, far right). Little or no change was observed at B6, indicating that the co‐solvent confounds the above diselenide‐specific stabilization of the Leu^B6^‐H_N_^…^O=C‐Sec^A6^ hydrogen bond. Although an organic co‐solvent would generally weaken core packing and so attenuate dynamic differences between analogs, an overall trend toward diselenide‐enhanced protection was nonetheless maintained. As expected, exceptions to this trend were observed at sites of rapid local exchange at the protein surface (as illustrated by Ser^A9^ and Gln^A15^; Figures S26–S28).

### 2.6 Se‐glargine exhibits only a marginal increase in thermodynamic stability

Thermodynamic stabilities of Se‐glargine and its parent were evaluated through measurement of global ^1^H‐^2^H exchange in 10 mM dAA (pH 3.0) and 25°C (Hua et al. 2011). Protein concentrations were made 60 μM to approach those employed in the above CD studies (25 μM). Because only long‐lived amide resonances are pertinent, successive ^1^H‐^1^H‐TOCSY spectra were acquired at the indicated time points after dissolution in 100% D_2_O. Under these conditions three sites of subglobal exchange (residues Gln^A15^, Val^B12^, and Ala^B14^) and three sites of presumed global exchange (Leu^A16^, Tyr^A19^, and Val^B18^) were observable (Figure S27); PFs are given in Figure 8d and Table S15. The largest PF in each analog occurs at position Val^B18^: 537(±25) (Se‐glargine) and 427(±39) (glargine); these values correspond to respective free energies of unfolding (Δ*G*_u_) of 3.72(±0.03) and 3.58(±0.05) kcal/mol, in turn implying only a small change in stabilization free energy (ΔΔ*G*_u_) of 0.14(±0.07) kcal/mol. Val^B12^, part of the first turn of the central B‐chain α‐helix, provides a representative site of subglobal exchange; its H_N_‐H_α_ TOCSY cross peak is well‐resolved under both solvent conditions (ω_2_ traces in Figure 8e).

Similar but not identical trends among global and subglobal sites of ^1^H/^2^H exchange were observed in the presence of the co‐solvent (Figures 8f and S28 and Table S16). Although absolute stabilities depend in part on which site is considered global and on the accuracy of standard random‐coil exchange rates in the context of the insulin sequence, the estimate of ΔΔ*G*_u_ is robust to such factors. These difference estimates are in each case markedly less than what was inferred from chemical denaturation studies (ΔΔ*G*_u_ 1.0(±0.2) kcal/mol as described by Weil‐Ktorza et al. 2024; see also Figure S8), a discrepancy presumably due to limitations of two‐state modeling (see section 3). The modesty of the inferred changes in thermodynamic stability stand in contrast to the pronounced differences between Se‐glargine and glargine with respect to fibrillation lag times and probes of conformational fluctuations. Addition of the co‐solvent also attenuates differences in rates of subglobal exchange as illustrated by Val^B12^ (Figure 8g).

### 2.7 Unrestrained MD trajectories support reduced conformational excursions in Se‐glargine

The solution structures of Se‐glargine and its parent (calculated based on NMR restraints) were extended by unrestrained MD simulations (1 μs × 10 replicas). In each case ensembles obtained from snapshots extracted from the MD trajectories maintain a nativelike insulin fold (i.e., without segmental unfolding or core reorganization; Figure 5e–h). In the simulations Se‐glargine nonetheless sampled a narrower range of conformations (relative to the parent WT glargine) near the A6–A11 bridge, including within the A1–A8 α‐helix. This conformational restriction is not associated with global structural rearrangement (Figure 5e–h). To relate the simulations to experiment, selected interproton distances (taken from Table S4) were converted to NOE‐related effective distances, *r*_weight_ = ⟨*r* ^−6^⟩^−1/6^, averaged across the extended replica set (Figure S29) to preferentially weight shorter distances. Whereas Se‐glargine and its parent generally exhibited similar interproton distance distributions, several contacts in Se‐glargine were modestly shifted toward shorter effective distances, consistent with attenuated local conformational excursions. The richer set of NOEs observed in Se‐glargine and the more extensive line broadening observed in the parent analog are thus in qualitative agreement with respective MD‐derived estimates of conformational fluctuations. The MD simulations are further described in the Supporting Information based on trajectories extended to 1 μs (sect. S1.4 of Data S1). Because effects of other NMR properties (such as spin diffusion, relaxation, and local correlation‐times) were not modeled, consistency between the simulations and the above NMR features is only qualitative. We have likewise not simulated other NMR observables, such as chemical‐shift broadening and ^1^H‐^2^H amide‐proton exchange.

## 3 DISCUSSION

The present study has focused on the dynamics of a globular protein and its modulation by a core diselenide bridge. As both model system and therapeutic protein, insulin has been well characterized by X‐ray crystallography (Adams et al. 1969; Baker et al. 1988; Derewenda et al. 1989), NMR spectroscopy (Chang et al. 1997; Hua et al. 1996; Hua and Weiss 1991; Jacoby et al. 1996; Keller et al. 2001; Knegtel et al. 1991; Olsen et al. 1996), and, in recent years, single‐particle cryo‐EM image reconstruction of hormone‐receptor complexes (Choi and Bai 2023; Scapin et al. 2018; Weis et al. 2018). A recurring theme has been *conformational change*, from the hexamer‐specific T → R transition (exploited to stabilize neutral‐pH pharmaceutical formulations; see sect. S1.1 of Data S1 and Figure S30) (Weiss 2009) to reorganization of the insulin monomer on receptor binding as a structural trigger for signal propagation (Menting et al. 2014). The variability of insulin's core packing efficiency—lower near cystine A6–A11 than near cystine B19–A20 (Baker et al. 1988)—exemplifies a general feature of globular proteins (Bhardwaj and Gerstein 2009; Richards 1979).

In previous studies we have shown that the A6–A11 diselenide bridge augments the resistance of insulin to reduction at neutral pH by dithiothreitol (Weil‐Ktorza et al. 2019), a general redox feature of selenocystine relative to cystine in peptides (Metanis and Hilvert 2012). These studies were conducted in the absence of the di‐Arg B‐chain extension in Se‐glargine, which otherwise renders the analog insoluble in pH range 7–8 as ordinarily employed in studies of insulin reduction. The acidic pH of insulin glargine in pharmaceutical formulations (pH 4) intrinsically stabilizes the monomeric protein with respect to reduction, but does not protect the protein from fibrillation (Figure 1c). Insulin retains its three native disulfide bridges on formation of fibrils (Kurouski et al. 2012), and so presumably Se‐glargine retains the A6–A11 diselenide bridge in corresponding fibrils. In a cryo‐EM‐based model of an insulin fibril (Wang et al. 2023) the structural environment of cystine A6–A11 readily accommodates a diselenide bridge with minimal local adjustments (sect. S1.5 of Data S1 and Figures S31 and S32). Our studies thus focused on native‐state dynamics rather than on the cross‐β fibril structure. Because the engineered diselenide bridge augments resistance to fibrillation (Figure 1d,e), its biophysical properties may be of translational interest.

### 3.1 Dynamics of an insulin monomer

Unlike well‐ordered zinc insulin hexamers, the ^1^H‐NMR spectrum of an isolated insulin monomer is remarkable for conformational broadening of a subset of amide resonances, implying that millisecond motions in the protein lead to incomplete averaging of chemical shifts (Hua et al. 1996; Hua et al. 2011; Weiss et al. 1989). Such resonance broadening is by contrast not observed in the ^1^H‐NMR spectrum of the R_6_ insulin hexamer (Jacoby et al. 1996), a rigid assembly stabilized by zinc‐ion coordination and phenolic ligands. Indeed, relative to the insulin monomer (Hua et al. 2011), the R_6_ hexamer exhibits (like the Se‐glargine monomer) enhanced ^1^H‐^2^H protection factors and higher inter‐residue NOE density (Jacoby et al. 1996) in association with prolonged fibrillation lag times (Brange et al. 1997). These R_6_ specific biophysical features are commonly exploited in many pharmaceutical formulations to extend shelf life (Brange and Langkjœr 1993). Hence, we sought by analogy to investigate whether enhancing the packing efficiency of the insulin *monomer's* hydrophobic core might likewise damp conformational fluctuations and retard fibrillation. Because conventional amino‐acid substitutions represent too marked a change in side‐chain volume or shape, our studies focused on subtle effects of substituting an internal disulfide bridge by a diselenide bridge (Figure S7) (Weil‐Ktorza et al. 2019; Weil‐Ktorza et al. 2024). We hypothesized that the slightly larger atomic radius of selenium (relative to sulfur) and slightly longer bond lengths in ‐H_2_C‐Se‐Se‐CH_2_‐ (bold and green; relative to ‐H_2_C‐S‐S‐CH_2_‐) could mitigate adjoining micro‐packing defects, in turn attenuating local and nonlocal motions.

Flexibility of the insulin B chain is required for its biological activity. Indeed, partial unfolding of the C‐terminal B‐chain segment (residues B24–B28) enables the hormone to engage the primary hormone‐binding site at the apex of the IR ectodomain (designated *Site 1*) as visualized by X‐ray crystallography (Menting et al. 2014) and cryo‐EM (Choi and Bai 2023; Scapin et al. 2018; Weis et al. 2018). Stabilization of this segment by chemical cross‐linking to the A chain (Nakagawa and Tager 1989) or by introduction of short peptide tethers between B‐ and A‐chains (<4 residues) markedly impairs activity (Derewenda et al. 1991; Hua et al. 1998; Huang et al. 2001; Kobayashi et al. 1989; Markussen et al. 1985). Because packing of the C‐terminal B‐chain segment against the α‐helical domain (via Phe^B24^, Tyr^B26^, and Pro^B28^) both impairs receptor binding and delays fibrillation, similar conformational fluctuations may be involved, at least in part, in both processes (Figure 1c). That the present A6–A11 diselenide bridge preserves biological activity (Figure S8) (Weil‐Ktorza et al. 2019; Weil‐Ktorza et al. 2024) implies that any enhanced efficiency of core packing does not hinder either (a) detachment of the B‐chain C‐terminal β‐strand on receptor binding or (b) docking of the A1–A8 α‐helix against the flat surface of the receptor's L1 β‐helix (Menting et al. 2014). The subtlety of a diselenide substitution stands in contrast to conventional small‐to‐large or large‐to‐small substitutions at conserved sites in insulin, which are broadly associated with marked impairment of bioactivity (Hu et al. 1993; Nakagawa and Tager 1992; Shoelson et al. 1983; Xu et al. 2002; Xu et al. 2004).

### 3.2 Thermodynamic stability versus conformational damping

Thermodynamic stabilities of globular proteins are typically inferred from chemical denaturation studies as interpreted by a two‐state model (Sosnick et al. 2000). Previous studies on Se‐insulin and Se‐glargine demonstrated that higher concentrations of denaturant (guanidine hydrochloride) were required for 50% unfolding (respective *C*_mid_ values 5.7(±0.1) and 6.0(±0.1)); respective free energies of unfolding (ΔΔ*G*_u_) were apparently augmented by 0.8(±0.2) and 1.0(±0.2) kcal/mol (Table S1) (Weil‐Ktorza et al. 2019; Weil‐Ktorza et al. 2024). In these assays extent of folding was probed by CD at helix‐sensitive wavelength 222 nm. Because (a) far‐UV CD spectra are sensitive to both helix content and segmental helical stability and (b) the conformational repertoire of the isolated monomer in microsecond MD simulations includes non‐helical excursions of the N‐terminal A‐chain segment (Figure S5) (Busto‐Moner et al. 2021), it seemed possible that the previous CD‐derived values of ΔΔ*G*_u_ overestimated the extent of diselenide‐associated thermodynamic stabilization. Non‐two‐state unfolding as a function of denaturant concentration, even if not resolvable by CD as a series of discrete transitions, could confound two‐state modeling, leading to inaccurate (but reproducible) estimates.

An independent estimate of a protein's thermodynamic stability may be obtained through analysis of global ^1^H‐^2^H amide‐proton exchange in D_2_O (Hua et al. 2011). This approach focuses on the largest PFs at sites at which ^1^H‐^2^H exchange requires *complete unfolding of the protein*. The few sites of global exchange in the insulin monomer reside in the central B‐chain α‐helix and C‐terminal A‐chain α‐helix adjoining cystine B19–A20 (residues Tyr^B16^, Val^B18^, Leu^A16^, and Tyr^A19^) (Hua et al. 2011). Like cystine A6–A11, the B19–A20 disulfide bridge is buried in the core, but unlike cystine A6–A11, its local structural environment is unaffected by the conformational excursions observed in microsecond MD simulations (Busto‐Moner et al. 2021). Analysis of these global PFs in Se‐glargine (relative to its parent; Figure 8d,f and Table S15) yielded a stability difference of only 0.14(±0.07) kcal/mole, suggesting that the larger CD‐based estimates were indeed influenced by the two‐state model. This confounding issue was presumably less pertinent in the original studies of substitutions in the central B‐chain α‐helix and C‐terminal A‐chain α‐helix (residues B10 and A13, respectively; Hua et al. 2011), for which independent estimates of ΔΔ*G*_u_ by CD and ^1^H‐^2^H exchange were concordant.

The present discordance between NMR‐ and CD‐based estimates of relative thermodynamic stabilities focused attention on the A1–A8 α‐helix. Constrained by the A6–A11 disulfide bridge, this segment is known to exhibit the following anomalies: (i) markedly attenuated PFs relative to the other two α‐helices (Hua et al. 2011), (ii) conformational broadening of amide ^1^H‐NMR resonances (Hua et al. 1996; Hua and Weiss 1991; Weiss et al. 1989), (iii) an overall change in orientation in the hexamer‐related TR transition (Chothia et al. 1983), (iv) local unfolding in an otherwise well‐ordered insulin variant (Ala^A2^‐insulin; Xu et al. 2002), and (v) non‐helical conformational excursions in the course of microsecond MD simulations (Busto‐Moner et al. 2021). The conformational lability of the native A1–A8 segment in an isolated monomer stands in contrast to its major role in receptor binding (Menting et al. 2013; Menting et al. 2014; Scapin et al. 2018; Weis et al. 2018). Steiner and colleagues have speculated that its segmental unfolding evolved to facilitate prohormone processing, as cleavage of the C–A junction of proinsulin requires an extended main‐chain conformation in the active site of furin‐related *proprotein convertase 2* (Lipkind and Steiner 1999).

In the present study we interrogated the dynamics of Se‐glargine (relative to its parent) through multiple biophysical probes, including (i) relative susceptibilities to cleavage by pepsin, (ii) far‐UV CD features, (iii) ^1^H‐NMR resonance line widths, (iv) secondary chemical shifts, (v) inter‐residue NOEs, and (vi) ^1^H‐^2^H amide‐proton exchange. Although solution structures of Se‐glargine and glargine are essentially identical, these studies in each case provided evidence that the diselenide bridge augments the segmental stability of the A1–A8 α‐helix and that such local dynamic changes propagate to the B chain. Remarkably, the degree of such dynamic stabilization—although modest in accordance with the structural similarity between cystine and selenocystine (Huber and Criddle 1967)—is nonetheless sufficient to mitigate the unfavorable susceptibility of insulin glargine to fibrillation (relative to WT insulin) above room temperature (Figure 1d,e). To our knowledge, Se‐glargine represents the first example of a core modification in an insulin analog that delays fibrillation.

### 3.3 MD simulations

**FIGURE 9:**
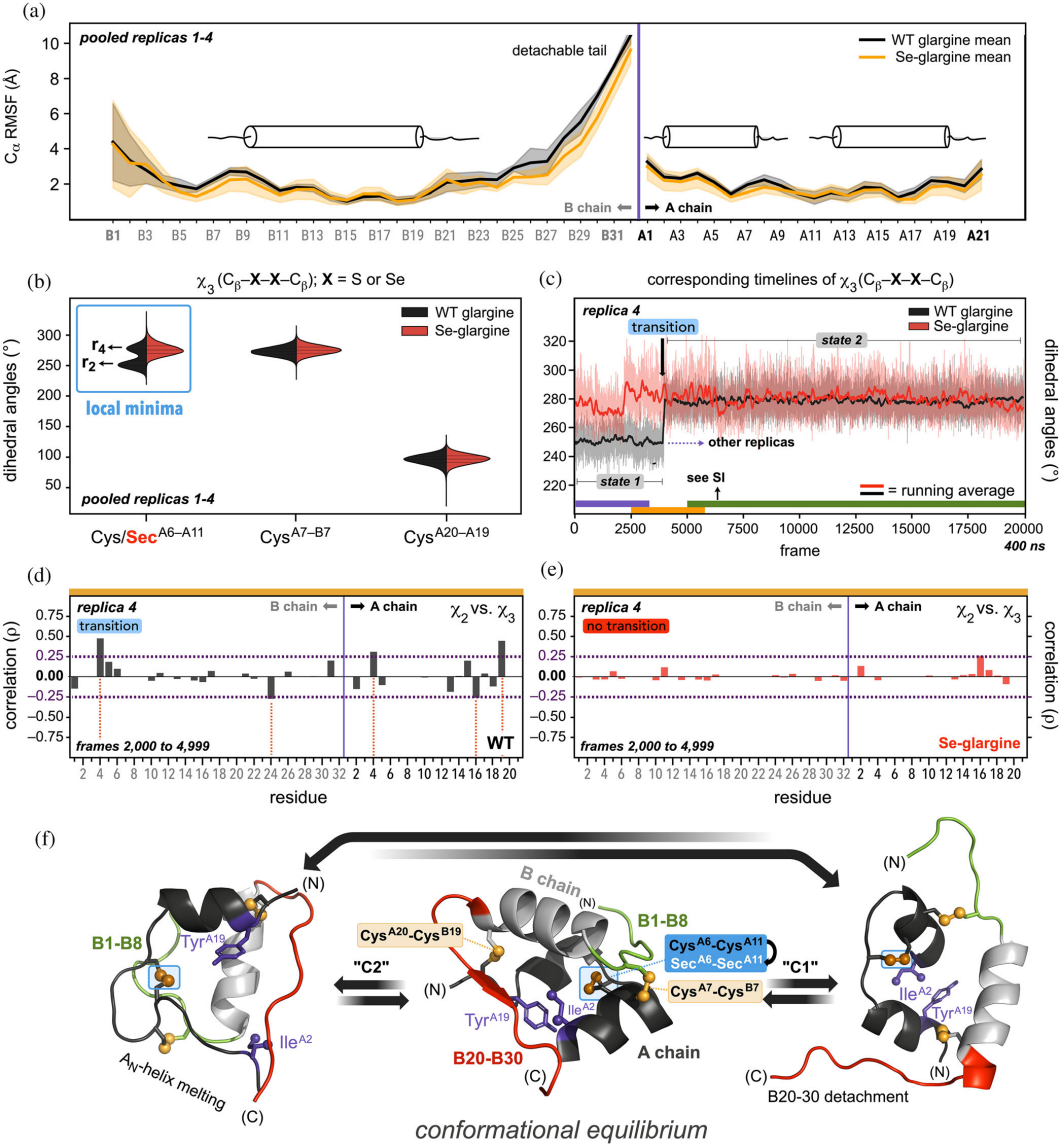
MD‐based conformational repertoire of WT glargine and Se‐glargine. The A6–A11 diselenide rigidifies local *χ*_3_ geometry. (a) Backbone C_α_ RMSF (mean ± SD over replicas 1–4) for B‐chain residues B1–B32 (left) and A‐chain residues A1–A21 (right), comparing WT (black/gray) and Se‐glargine (orange). Overall flexibility is similar, with modest damping around the A6–A11 region in Se‐glargine. (b) Violin plots of *χ*_3_ (C_β_–X–X–C_β_; X = S or Se) for the three bridges A6–A11, A7–B7, and A20–B19, pooled over initial replicas 1–4. Only A6–A11 is mutated to a diselenide bridge, and the *χ*_3_ distribution at this site collapses into a narrow local basin in Se‐glargine (blue box), whereas the native A7–B7 and A20–B19 disulfides remain unchanged. (c) Representative *χ*_3_ timelines (initial replica 4) for the A6–A11 bridges over 400 ns (20,000 frames), showing instantaneous values (shaded traces) and 100‐frame running averages (solid curves) for WT and Se‐glargine. Replica 4 undergoes a transition between two *χ*_3_ basins (“state 1” → “state 2”). Other replicas (e.g., replica 2; see Figure S36b) remain in a single “state 2” basin. Additional extended replicas (*r*_1_–*r*_10_) and simulation length (1 μs) are reported in Figure S33. (d, e) Computed Pearson correlation (*ρ*) between the *χ*_3_ dihedral and side‐chain *χ*_2_ dihedrals for each residue in both chains, used to assess whether structural changes propagate during the transition. Multiple window sizes were evaluated (additional analyses in Figures S36–S38); the transition window shown here corresponds to frames 2500–5499 of replica 4. WT and Se‐glargine are shown separately, with vertical dashed orange markers highlighting residues with moderate correlations in WT that are markedly reduced in Se‐glargine. (f) Structural cartoon summarizing equilibrium between a compact “**C1**” state and an alternative “**C2**” conformation (Busto‐Moner et al. 2021): the A chain (black), B1–B8 segment (green), and B20–B30 segment (red) are shown together with the A6–A11 S–S/Se–Se bridge (orange). These ribbon‐models illustrate coupled motions involving A‐chain local structure (“A‐helix melting”) and B20–B30 detachment. See Figure S5 for a broader structural overview of the human insulin conformational ensemble reported by Busto‐Moner et al. (2021).

The MD simulations suggest that even as the diselenide bridge does not alter the overall insulin fold, it dampens local conformational excursions. Relative to the parent glargine, Se‐glargine shows slightly reduced root‐mean‐square fluctuations (RMSF) in regions implicated in early partial unfolding, suggesting more efficient local packing rather than a global structural rearrangement (Figures 5, 6, and 9a). Local conformational restriction is most evident in the *χ*_3_ distributions of the three bridges. Whereas the native A7–B7 and A20–B19 disulfides behave similarly in the two analogs, the A6–A11 Se–Se bond adopts a markedly narrower *χ*_3_ distribution in Se‐glargine than does the corresponding A6–A11 disulfide bridge in the parent analog (Figures 9b,c and S33). Across the extended MD replicas, WT glargine sampled two χ_3_ states, whereas Se‐glargine remained largely confined to one, consistent with suppression of conformational exchange on the simulated MD timescale. A plausible structural basis is that the larger Se–Se bridge packs more efficiently against neighboring core residues, disfavoring the alternative WT rotamer (Figures S33c, S34–S35). Pearson‐correlation analysis (see Taddese et al. 2020 and sect. S2.2 of Data S2 for *χ*_3_ definition, circular‐correlation methodology, trajectory‐window selection, and correlation‐strength thresholds) further suggests that local A6–A11 *χ*_3_ dynamics in glargine are coupled to broader conformational fluctuations, *at a distance*, in both chains. In marked contrast, analogous correlations in Se‐glargine are weak in the absence of *χ*_3_ exchange (Figures 9d,e and S36–S38). The *χ*_3_ exchange was observed in two of the four initial replicas (400 ns each) (Figures S39 and S40). Together, these simulations provided evidence that the diselenide bridge damps local conformational excursions in the native‐state ensemble on a time scale of 1 μs. We envision that in the future longer MD simulations (including trajectories initiated from the non‐native partial folds described by Busto‐Moner et al. 2021) may provide insight into possible conformations of amyloidogenic partial folds (Figures 9f and S5).

### 3.4 Clinical challenge of insulin fibrillation

The susceptibility of insulin to fibrillation was first noted >90 years ago in relation to its pharmaceutical manufacture, purification, and storage (Waugh 1941; Waugh 1944; Waugh 1946). Remarkably, decades prior to the modern understanding of amyloid (Chiti and Dobson 2009) and its mechanistic relationship to seeded cross‐β assembly (Fitzpatrick et al. 2013), empirical factors promoting or delaying insulin fibrillation were identified as real‐world steps to ensure the hormone's broad availability for patients with diabetes mellitus (Brange 1987). This history foreshadowed current concepts in protein science, including the seminal role of conformational fluctuations in fibrillation (Brange and Langkjœr 1993; Hua and Weiss 2004). Risk of insulin fibrillation in current pharmaceutical formulations is mitigated at the societal level by regulatory requirements for refrigeration during transport and storage and by product‐specific shelf lives with expiration dates (Grajower et al. 2003). Molecular strategies to avoid fibrillation have focused on native self‐assembly (including the use of zinc‐stabilized hexamers and micro‐crystallization; Brange 1987) and addition of stabilizing small molecules (such as phenolic preservatives bound to R_6_ zinc hexamers; Smith and Dodson 1992). Despite these advances, the baseline susceptibility of insulin to fibrillation above room temperature continues to impose a complex and costly cold chain in the developing world (Heinemann et al. 2021). Avoidance of fibrillation is of clinical importance because (a) such cross‐β assemblies lack hormonal activity and (b) protein aggregation at injection sites can provoke inflammatory responses (Lewis et al. 2023; Nakamura et al. 2018), including neutralizing anti‐insulin antibodies (Van Haeften 1989). Finally, fibrils can occlude insulin delivery devices (such as pens and pumps), obstructing subcutaneous injection (Klonoff et al. 2017).

The A6–A11 diselenide substitution in insulin glargine restores its mean fibrillation lag time, foreshortened by the analog's modifications, to that of WT insulin. What's more, lag‐time variability is reduced. The latter may have independent clinical significance, as the stochastic nature of protein fibrillation has implications for both shelf life and patient safety. Such lag times are, in general, sensitive to subtle variations in conditions, amplifying effects of stochastic seeding (Figure 1c). Indeed, unpredictability from vial to vial regarding onset of aggregation itself imposes regulatory challenges, as guidelines must balance the statistical risk of degradation versus the cost of discarding still‐active protein solutions (Weiss 2023). What defines an acceptable level of risk has engendered controversy (Pendsey et al. 2023).

### 3.5 Concluding remarks

Because the native state of a protein is only slightly more stable than amyloidogenic partial folds and the unfolded‐state ensemble, proteins are marginally stable (Dobson 2003). Traditional strategies of protein engineering seek to reduce the propensity for aggregation‐coupled denaturation either through protective self‐assembly (Zhu et al. 2021), design of more stable variants (Chennamsetty et al. 2009; Ebrahimi and Samanta 2023), introduction of stabilizing small molecules as protective ligands (Fagihi and Bhattacharjee 2022; Sen et al. 2022), or attachment of hydrophilic tags such as polyethylene glycol (Harris and Chess 2003; Lawrence et al. 2014). The present study demonstrates an alternative approach based on protein dynamics: a subtle modification of a protein's hydrophobic core—substitution of a disulfide bridge by a diselenide bridge—damps amyloid‐associated conformational fluctuations. Whereas the solution structure and thermodynamic stability of the variant protein are similar to its parent, local and nonlocal damping of conformational fluctuations was uncovered by a variety of biochemical and biophysical probes. A model was provided by insulin glargine, a long‐acting insulin analog in broad clinical use (Bolli and Owens 2000). Its dynamic stabilization promises to be of complementary basic and applied interest.

Substitution of an internal disulfide bridge in an insulin analog by a diselenide bridge highlights the importance of core packing efficiency in globular proteins. Local packing efficiencies, never perfect, typically vary from site to site in a core. Whereas conventional small‐to‐large substitutions (such as Val → Leu) represent a coarse modification on the atomic scale, often associated with transmitted conformational changes to avoid steric clash (Baase et al. 2010), a diselenide substitution defines a more subtle core modification, enhancing local packing efficiency without perturbation of an overlying functional surface. We anticipate that the present findings may stimulate comparative studies of disulfide‐ and diselenide‐stabilized proteins by high‐pressure NMR spectroscopy (Charlier et al. 2018; Kremer 2006). This residue‐specific approach may enable changes in relative cavity sizes to be probed (Lassalle et al. 2001; Maeno et al. 2015; Roche et al. 2013); further information may be obtained by pressure‐jump NMR experiments (Charlier et al. 2018; Roche et al. 2013). Application of these techniques to Se‐glargine and glargine promises to illuminate relationships between core packing efficiency, native‐state dynamics and amyloidogenesis. We anticipate that such relationships will pertain to diverse therapeutic proteins and industrial enzymes.

## 4 MATERIALS AND METHODS

### 4.1 Preparation and purification of insulin analogs

Human insulin (Humulin® R), insulin lispro (Humalog®), and glargine (Lantus®) were obtained from pharmaceutical vials after purification by reversed‐phase HPLC. Se‐glargine was prepared by chain combination of individual chains, B‐chain S‐sulfonate (obtained from sulfitolysis of glargine) and Se^A6^‐Se^A11^ modified A chain (prepared by solid phase peptide synthesis) as described (Weil‐Ktorza et al. 2024). Analytical reversed‐phase HPLC was performed on a Waters Alliance HPLC with 220 nm UV detection. Linear gradients of acetonitrile (with 0.1% TFA, eluent B) in water (with 0.1% TFA, eluent A) were used to elute bound proteins. Semipreparative HPLC was performed using a Waters 1525 Binary HPLC system. Chromatographic separations were carried out on a TARGA C8 5μm (250 × 10 mm) Higgins Analytical, Inc. column using 25–50% Solvent B in Solvent A over 35 min. (solvent A = 0.1% TFA in water, solvent B = 0.1% TFA in acetonitrile) at a flow rate of 4.0 mL/min with detection by UV absorption at 215 nm. Liquid chromatography‐coupled mass spectrometry (LC–MS) was performed using an LCQ Advantage Ion Trap Mass Spectrometer (MS) System coupled with Agilent 1100 Series HPLC system. Masses were confirmed by online electrospray mass spectrometry.

### 4.2 Protein fibrillation assays

Physical stability was assessed by propensity to form fibrils. Insulin or analogs were made 60 μM in 10 mM Tris–HCl, 140 mM NaCl (pH 4.0), and 16 μM Thioflavin T (ThT). Samples were plated in a Costar® plate (250 μL/well) and incubated with continuous shaking in a Biotek Synergy H1® plate reader at 37°C. ThT fluorescence at 480 nm was assessed (following excitation at 440 nm) at 15 min intervals. A twofold increase in fluorescence intensity from the baseline defined the lag time.

### 4.3 Circular dichroism

Samples were quantitated using Genesis 150 UV–Vis spectrophotometer (Thermo Scientific) using 1‐cm cuvette by measuring peptide absorbances at 280 nm. Respective extinction coefficients used were 6335 M^−1^·cm^−1^ (WT insulin and *glargine*) and 6210 M^−1^·cm^−1^ (Se‐glargine). Far‐UV spectra were obtained using a Jasco J‐1500 spectropolarimeter; spectra were obtained from 190 to 255 nm as described (Sreerama and Woody 2000). Insulin analogs were made 50 μM in 10 mM Tris–HCl and 50 mM KCl (pH 4).

### 4.4 Protein stability

Thermal stability was probed using CD spectroscopy by monitoring helix‐sensitive wavelength 222 nm from 4 to 88°C; the heating rate was 1°C per min. Initial estimates of thermodynamic stability were inferred (based on a two‐state model) using a CD spectrophotometer equipped with an automated syringe‐driven titration unit. Helix‐sensitive wavelength 222 nm was used as a probe of denaturation of protein (at 5 μM) by guanidine hydrochloride. Validity of inferred parameters depends on the applicability of two‐state modeling (Sosnick et al. 2000). Protein stability was further assessed by relative susceptibility to pepsin digestion. To this end, 0.1 mg of insulin was dissolved in 100 μL 10 mM HCl (at a protein concentration of 1 mg/mL as measured by UV), at pH ~2. A stock solution of pepsin enzyme was prepared by dissolving 4.7 mg of the protease in 4.7 mL of 10 mM HCl (enzyme concentration 1 mg/mL), then 200 μL of this stock solution were added to 800 μL, 10 mM HCl to obtain a final enzyme concentration of 0.2 mg/mL. The pepsin solutions were then placed on ice. In the pepsin degradation assay, 1 μL of 0.2 mg/mL pepsin was added to 100 μL of protein (1 mg/mL at pH ~2), and the reactions were incubated at 37°C. To monitor degradation, 7 μL aliquots of the reaction were taken and quenched with 15 μL of 10 mM NaOH and were placed on ice to stop the reaction. Aliquots were analyzed by analytical HPLC using an XSelect C4 column (3.5 μm, 130 Å, 4.6 × 150 mm); elution employed a gradient of 5% eluent B in eluent A for 1 min and then 10–60% B over 20 min.

### 4.5 NMR spectroscopy

Initial NMR spectra were acquired at a proton frequency of 700 MHz at pH 3.0 (direct meter reading) in 10 mM dAA at 25°C at protein concentrations <100 μM. 2D homonuclear‐ and heteronuclear‐NMR spectra were also acquired at pH 2.1 (direct meter reading) in 10% dAA at 25°C at protein concentrations of 300 μM. All NMR spectra were acquired using a BRUKER 700 MHz spectrometer equipped with quadruple [^1^H, ^19^F, ^13^C, ^15^N]‐resonance liquid‐helium‐cooled cryoprobe; data were processed with Topspin 4.0.5 (Bruker Biospin) and analyzed with Sparky software (Lee et al. 2015).

### 4.6 Chemical shift perturbation

Chemical‐shift assignments were accomplished by homonuclear 2D ^1^H‐^1^H NOESY, TOCSY, DQF‐COSY in D_2_O and H_2_O (10% D_2_O), and natural abundance ^1^H‐^15^N and ^1^H‐^13^C HSQC spectra. Chemical‐shift perturbations were defined by chemical‐shift differences between corresponding resonances in Se‐glargine and glargine. Combined ^15^N/^1^H_N_ or ^13^C_α_/^1^H_α_ chemical‐shift perturbations were calculated according to the equation $\Delta_{N/\mathrm{HN}}=\sqrt{\delta_{\mathrm{HN}}^{2}+\delta_{N}^{2}}$(or $\Delta_{\mathrm{C\alpha}/\mathrm{H\alpha}}=\sqrt{\delta_{\mathrm{H\alpha}}^{2}+\delta_{\mathrm{C\alpha}}^{2}}$), where δ_HN_ and δ_N_ are respective ^1^H_N_ and ^15^N chemical‐shift differences between Se‐glargine and glargine; δ_Hα_ and δ_Cα_ are respective ^1^H_α_ and ^13^C_α_ chemical shift differences between Se‐glargine and glargine. Combined backbone chemical shift perturbation was calculated according to the equation $\Delta_{\text{backbone}}=\sqrt{\Delta_{N/\mathrm{HN}}^{2}+\Delta_{\mathrm{C\alpha}/\mathrm{H\alpha}}^{2}},$ where Δ_Ν/ΗΝ_ and Δ_Cα/Hα_ are the combined ^15^N/^1^H_N_ and ^13^C_α_/^1^H_α_ chemical‐shift differences between Se‐glargine and its parent analog, respectively.

### 4.7 ^1^H‐ ^2^H amide proton exchange

^1^H‐^2^H exchange was monitored in 100% D_2_O in 10% dAA at pH 2.1 (direct meter reading) or in 10 mM dAA at pH 3.0 (direct meter reading) at 25°C. Respective samples of glargine or Se‐glargine (lyophilized from H_2_O in 10% or 10 mM acetic acid) were placed on ice followed by addition of chilled D_2_O in 10% or 10 mM dAA. A series of 1D proton and 2D TOCSY spectra were collected to measure exchange of amide protons as a function of resonance intensity; 1D ^1^H spectra were collected at 2 min intervals to assess very fast exchange, whereas 2D TOCSY spectra were acquired to assess amide protons with slower exchange rates. PFs were calculated from the ratio of measured exchange rate and intrinsic exchange rate (Glidden et al. 2018; Hua et al. 2011; Rege et al. 2020).

### 4.8 NMR structure determination

NMR structures were calculated based on the hybrid distance geometry‐dynamical simulated annealing method (Nilges et al. 1988; Omichinski et al. 1990) using *XPLOR‐NIH* (Schwieters et al. 2003; Schwieters et al. 2006) as described. Distance restraints were derived from through‐space correlation NOESY experiments acquired in H_2_O (90/10 volume ratio) and D_2_O. NOEs were classified as strong, medium, weak, and very weak categories corresponding to interproton distance‐restraint ranges of 1.8–2.7 Å (1.8–2.9 Å for NOEs involving NH protons), 1.8–3.3 Å (1.8–3.5 Å for NOEs involving NH protons), and 1.8–5.0 Å and 1.8–6.0 Å, respectively (Clore et al. 1986; Williamson et al. 1985). An additional 0.5 Å was added to methyl‐related distance restraints. Initial structures were calculated by employing only unambiguous NOEs; subsequent calculations were iteratively performed on further NOE analysis. Twenty best‐fit structures were chosen from 100 calculated structures. Structural ensembles were visualized using *PyMOL* (Schrodinger 2015) and *Molmol* software (Koradi et al. 1996). The present solution structure of native insulin glargine, as determined herein in 10% dAA, more closely resembles the crystal structure of glargine (RMSD 0.6 Å as calculated based on alignment of main‐chain atoms in the three helices) than does a previously reported NMR‐derived structure (PDB entry 6K59; Ratha et al. 2020) as determined in a higher concentration of the organic co‐solvent (20% dAA; RMSD 1.8 Å) as illustrated in Figure S41.

### 4.9 Molecular dynamics simulations

All‐atom MD simulations were performed on monomeric glargine and Se‐glargine to characterize dynamic consequences of A6–A11 diselenide substitution on a time scale of 1–1000 ns. Starting coordinates were taken from the NMR structure of glargine; the Se‐glargine model was obtained by mutating Cys^A6^ and Cys^A11^ to selenocysteine and replacing the A6–A11 S–S bond with a Se–Se bond of appropriate equilibrium length (2.35 Å). The chosen 2.35 Å bond distance was implemented by inspecting a crystallographic octamer structure containing A6–A11 diselenide bridges (PDB entry 6H3M; Weil‐Ktorza et al. 2019). Literature reports also supported this Se–Se bond distance for diselenides of different types (Herrmann 1997; Takaluoma et al. 2015). Further details on the workflow for implementing a diselenide bridge in CHARMM are provided in Figure S42 and in sect. S2.3 of Data S2. Each protein was placed in a periodic cubic box of explicit TIP3P water with at least 10 Å between any solute atom and the box edge; Na^+^ and Cl^−^ ions were added to achieve electroneutrality and an ionic strength of 0.15 M (representing physiological conditions). Simulations used the CHARMM36 protein force field (Best et al. 2012a; Best et al. 2012b) together with previously validated Lennard‐Jones parameters (*ε*, kcal·mol^−1^; *σ*, Å) for a methyl selenol model of selenocysteine (CH₃SeH), obtained by scaling sulfur to selenium as described in table 1 of Pedron et al. (2023). Simulation parameters are summarized in Tables S17–S22. Notably, although prior MD studies report Se–Se interactions (Fehér et al. 2016), they have effectively modeled these using S–S parameters; here, we provide the first explicit Se–Se implementation within CHARMM. After energy minimization and stepwise heating, systems were equilibrated and simulated in the NPT ensemble at 310.15 K and 1 atm using the BLaDE engine in CHARMM (build c48b1) (Brooks et al. 1983; Brooks et al. 2009) with particle–mesh Ewald electrostatics (Darden et al. 1993; Essmann et al. 1995), a 12‐Å real‐space cutoff, and SHAKE constraints on bonds to hydrogen (Ryckaert et al. 1977), enabling a 2‐fs integration‐time step. For each analog, the initial exploratory set involved four independent 400 ns production trajectories were generated from different randomized velocity seeds. An expanded sampling to validate the A6–A11 *χ*_3_ torsion dynamics was then performed by both expanding the replica sampling and simulation length (10 replicas per WT/Se–glargine for 1 μs, with a cumulative simulation time of 20 μs). Split violin and timeline plots illustrated in Figure S33 confirm suppressed A6–A11 *χ*_3_ state‐switching in Se‐glargine, whereas corresponding simulations of its parent analog exhibited intermittent transitions between two *χ*_3_ basins in multiple replicas. These MD trends support dynamic redistribution rather than a major structural change. Analysis of dihedral angles and backbone root‐mean‐square fluctuations was performed on all frames of these trajectories using standard CHARMM/MDAnalysis tools. Discussions and geometric analyses pertaining to select species in Figures 9f and S5 is provided in Table S23 and Figure S43.

Local packing around the A6–A11 bridge was evaluated by a container‐based MoloVol analysis (Maglic and Lavendomme 2022). An 11‐Å cubic container (yellow atoms in Figure S35) composed of dummy atoms was first positioned in the crystallographic insulin monomer scaffold (PDB entry 4INS), centered on the C_δ1_ atom of Leu^B11^, and the same container geometry was then transferred to Se‐glargine and its parent after superposition of their respective A chains onto the A chain of a WT insulin T‐state protomer (PDB entry 4INS), so that the same spatial region was sampled in each structure. Void volumes within this container were computed with MoloVol using a probe radius of 0.20 Å and a grid spacing of 0.075 Å; sulfur and selenium models were evaluated using the corresponding van der Waals radii (1.80 and 1.90 Å, respectively) and adjusted bond distances.

### 4.10 Molecular modeling of an insulin fibril

To explore whether the A6–A11 diselenide bridge could be accommodated in a fibril structure, 0.5 μs simulations were undertaken based on a cryo‐EM‐derived structure (PDB entry 8SBD; Wang et al. 2023). The simulations assumed pH 7 at a nominal temperature of 40°C. The system was simplified to encompass a five‐layer insulin fibril (protomer 1) with analysis confined to the middle three layers.

## AUTHOR CONTRIBUTIONS

**Yanwu Yang:** Methodology; writing – review and editing; data curation; investigation; visualization; software; validation. **Andreas Ehnbom:** Methodology; writing – review and editing; data curation; investigation; visualization; software; validation. **Orit Weil‐Ktorza:** Methodology; data curation; investigation; validation. **Norman Metanis:** Methodology; data curation; investigation; funding acquisition; validation. **Balamurugan Dhayalan:** Methodology; writing – review and editing; data curation; investigation; visualization; validation. **Michael A. Weiss:** Writing – review and editing; writing – original draft; funding acquisition; project administration; conceptualization; formal analysis; supervision; investigation; resources; validation.

## FUNDING INFORMATION

U.S. National Institutes of Health, Grant/Award number: NIH R01 DK04949; Israel Science Foundation, Grant/Award number: 1388/22; INCITE program of the Lilly Foundation at the Indiana University School of Medicine and the Distinguished Professor Fund of Indiana University.

## CONFLICT OF INTEREST STATEMENT

O.W.‐K., N.M., and M.A.W. are among the co‐inventors of a pending U.S. patent application, entitled “Stabilization of prandial or basal insulin analogs by an internal diselenide bridge” (US20220144915A1), jointly submitted by Hebrew University and Indiana University.

## Supporting information


**Figure S1.** A schematic energy landscape for protein folding and aggregation. Figure adapted from (Jahn and Radford, 2005). The surface shows the multitude of conformations ‘funneling’ towards the native state via intramolecular contact formation, or towards the formation of amyloid fibrils via intermolecular contacts.
**Figure S2**. Overview of ion‐dependent aggregation pathways. Reproduced from publication by Lenton et al. (2025). Sulfate promotes a colloidal pathway, forming folded aggregates that later convert to β‐rich amyloids and can undergo phase separation or crystallization (top). Perchlorate yields an intermediate pathway (middle), whereas chloride enforces a conformational pathway in which unfolding drives formation of elongated β‐sheet fibrils (bottom).
**Figure S3**. Pharmacokinetic profile of insulin injections: onset, peak activity, and duration of action (adapted from Petznick, 2011).
**Figure S4**. Prior strategies for improving insulin stability involved linking the B‐ and A‐chains with short peptide connectors and introducing an additional disulfide bond. (a) NMR structure of single chain insulin (PDB‐ID: 2LWZ). (b) X‐ray crystal structure of four‐disulfide insulin (PDB‐ID: 4EFX). Color coding: A chain (pink), B chain (blue), C domain (green), disulfides are shown as gold spheres with one third van der Waals radii.
**Figure S5**. Conformational “islands” **C0–C9** of monomeric human insulin as defined by Dinner and co‐workers (Busto‐Moner et al. 2021). The central structure shows the A chain (dark gray) and B chain (light gray) with the B‐chain N‐terminal segment B1–B8 (green), C‐terminal segment B20–B30 (red), and the A‐chain disulfide Cys^A6^–Cys^A11^ (burnt orange). Surrounding panels depict representative structures from each island (**C0–C9**) projected around the circle, colored with the same scheme. Labels and percentages indicate the corresponding island identity and fractional population in the conformational ensemble.
**Figure S6**. High conservation within the core. (a) Stereo view of insulin monomer with conserved residues highlighted. Color coding: A chain (pink), B chain (blue), methyl groups, and disulfides (gold) are shown as spheres with one third van der Waals radii. (b) Sequences of insulins from different species with bold residues being core residues that are >90% conserved.
**Figure S7**. Structural comparisons of S–S (WT) and Se–Se A6–A11 bridges in glargine. (a) Ball‐and‐stick models of the A6–A11 bridge showing the disulfide (S–S) and diselenide (Se–Se); the rightmost inset places both bridges on the same scale to emphasize that Se–Se is longer (and more polarizable) than S–S. (b) Geometric parameters used in the corresponding simulations are shown to scale. For more details on the underlying values used, refer to a Se–insulin analog (PDB‐ID: 6H3M; Weil‐Ktorza et al. 2019) with eight crystallographically independent protomers containing SecA6–SecA11 diselenide bridges. The observed Sec^A6^–Sec^A11^ distances clustered directly below ~2.4 Å and we therefore adopted an equilibrium Se–Se bond length of 2.35 Å for the A6–A11 diselenide in our CHARMM parametrization, which match a wide search in Conquest by Cambridge Structural Database of Se–Se bonds. The equilibrium distance in the two species is *r*_0(S–S)_ = 2.03 Å, and *r*_0(Se–Se)_ = 2.35 Å, respectively. Circle sizes represent the relative van der Waals radii of S and Se, and colored dots indicate the atom centers from which the bond distances are measured.
**Figure S8**. Biological activity of selenium‐substituted glargine (Se‐glargine). (a, b) Schematic of the in‐cell Western blot assay used to assess the activity of insulin analogs. Human HepG2 cells were plated in parallelized 96‐well formats and treated with increasing concentrations of individual insulin analogs (a), enabling fluorescent detection of hormone‐dependent insulin receptor (IR) autophosphorylation (b). Hormone‐induced IR autophosphorylation was quantified by optical readouts (upper panels; pseudo‐green signals). Signal intensity was normalized to DRAQ5 fluorescence at 700 nm (lower panels; pseudo‐red signals) to control for cell number. (c) Dose–response curves showing fold changes in IR autophosphorylation (pIR/IR; *y*‐axis) as a function of insulin analog concentration (50 pM–1 μM; *x*‐axis, log scale). Data represent mean ± SEM from three independent biological replicates. Data in this panel is reproduced from prior publication for convenience of the reader (Weil‐Ktorza et al. 2024). (d) Representative Western blots detecting insulin receptor phosphorylation and downstream Akt phosphorylation following stimulation with the indicated insulin analogs. Phosphorylated IR bands are marked by red arrows, and molecular weight markers are shown to the left of each gel.
**Figure S9**. HPLC monitoring of pepsin cleavage reaction using native glargine, i.e., with Cys^A6^ and Cys^A11^; henceforth designated simply “glargine.” The peak corresponding to the starting material at a retention time of 13.8 min gradually diminishes following pepsin treatment.
**Figure S10**. HPLC monitoring of pepsin cleavage reaction using Se‐glargine. The peak corresponding to the starting material at a retention time of 13.4 min gradually diminishes following pepsin treatment.
**Figure S11**. Analysis of stability differences between glargine and Se‐glargine. (a) Wavelength scans of glargine from 4–40°C. (b) Wavelength scans of Se‐glargine over the same range. Insets highlight the 222 nm band region. (c) Expanded regions from 220–225 nm is provided. (d) Plot of 222 nm band vs temperature reveals aberrant slope for glargine when compared to Se‐glargine.
**Figure S12**. Homonuclear 2D‐NMR spectra of glargine (*left panel, black*) and Se‐glargine (*right panel, maroon*): (*a*) NOESY spectra (mixing time 150 ms) showing NOEs from aromatic protons to methyl protons and (*b*) TOCSY spectra (mixing time 55 ms) showing aromatic resonance correlation. Spectra were acquired at a ^1^H frequency of 700 MHz in 10% deuterated acetic acid (pH 2.1, direct meter reading) at 25°C.
**Figure S13**. Natural abundance ^1^H‐^13^C HSQC spectra of glargine insulin (left panel, black) and A6–A11 Se‐glargine (right panel, maroon) at (a) methyl and (b) aromatic regions. Spectra were acquired at a ^1^H frequency of 700 MHz in 10% deuterated acetic acid (pH 2.1, direct meter reading) at 25°C.
**Figure S14**. (a) Structure representation of amide protons affected by A6‐A11 substitution (PDB‐ID: 4INS). The A chain is in *light pink*, B chain in *light blue* and disulfide bridges in *gold*. The amide protons of Cys^A11^, Leu^B6^, Gly^B8^ and Ser^B9^ are shown in *green spheres*, the carbonyl oxygen atoms of Gln^B4^, Ser^B9^, Cys^A6^ and Ser^B9^ side‐chain oxygen in *red spheres*. Dashed lines indicate Leu^B6^‐H_N_⋯O=C‐Cys^A6^, Cys^A11^‐H_N_⋯O=C‐Gln^B4^ and Ser^B9^‐H_N_^**…**^OH‐Ser^B9^ hydrogen bonds. (b) Structure representation of the transmission of chemical‐shift perturbations from A6‐A11 disulfide bridge to distant sites through hydrophobic interactions.
**Figure S15**. Homonuclear 2D NOESY spectra (mixing time 150 ms) of glargine (*black*) and Se‐glargine (*maroon*): (a) NOESY spectra showing A11 H_β_‐related NOEs of Se‐glargine. (b) NOESY spectra showing A11 H_β_‐related NOEs of glargine. Only A11 H_β2_ NOE cross peaks were clearly visualized in the spectrum of glargine. The A11 H_β1_‐related NOE cross peaks were not visualized due to exchange line broadening. Dashed circle indicated assuming NOE cross peak. (c) Structure representation of A11‐related NOEs that were only observed in the Se‐glargine.
**Figure S16**. Expanded and stereo view of the major hydrophobic environment in the ensemble (*left*) and in a representative stick model (*right*). The structure was aligned for residues 1–21 (A1–A21) and 24–49 (B3–B28). (a) Glargine insulin; the A chain is *cyan* and B chain *gray*. Methyl groups represented as *cyan or gray spheres*. (b) A6–A11 diselenide glargine insulin; the A chain is *green* and B chain *blue*. Methyl groups represented as *green or blue spheres*.
**Figure S17**. Structure comparison of Se‐glargine and glargine insulin. (a) Overlay of NMR structure of Se‐glargine and glargine; ribbon structure (left panel) and major hydrophobic core (right panel). The A chain is in *green* and B chain in *blue* in Se‐glargine*;* and the A chain is in *cyan* and B chain in *gray* in glargine; disulfide bridges are *gold and* diselenide bond *violet*. (b) Overlay of NMR structure of Se‐glargine and X‐ray structure of glargine insulin (PDB‐ID: 4IYD); ribbon structure (left panel) and major hydrophobic core (right panel). The color code of Se‐glargine is same as in panel *a*; the A chain is in *light pink* and B chain in *light blue* in the X‐ray model of glargine. Methyl groups represented as corresponding *spheres*.
**Figure S18**. (a) Stereo view of structure representation of the communication path from A6–A11 diselenide bridge to the amide proton of Val^B12^ in Se‐glargine. Methyl groups represented as *green* (A chain) or *blue spheres* (B chain), Val^B12^ amide proton in *white sphere*. (b) Side‐chain chemical shift perturbations related to the atomic distance from A6–A11 diselenide bridge. The distance is the average from A6/A11‐Se to side‐chain carbon (or nitrogen) atom. For ‐CH_2_‐ side‐chain groups, bigger chemical shift perturbation was selected. A‐chain residues are displayed in *green* and B‐chain residues in *blue*.
**Figure S19**. (a) Ribbon structural representation of insulin dimer (PDB‐ID: 4IYD) and that with important interfacial residues (b). Slow exchange amide protons between monomer and dimer were shown in *green* spheres that displayed exchange cross peak in the TOCSY and NOESY spectra. The A chain is in *light pink* and B chain in *gray* in one protomer*;* and the A chain is in *wheat* and B chain in *light purple* in another protomer; disulfide bridges are in *gold*. Methyl groups in the dimer interface represented as corresponding *spheres*.
**Figure S20**. NMR evidence of monomer‐dimer equilibrium of glargine and Se‐glargine in low protein concentration (~60 μM). (a) Spectral overlay of TOCSY (*back*) and NOESY (*orange*) spectra of glargine. (b) Spectral overlay of TOCSY (*blue*) and NOESY (*green*) spectra of Se‐glargine. Spectra were acquired at a ^1^H frequency of 700 MHz in 10 mM deuterated acetic acid (pH 3.0, direct meter reading) at 25°C. (c) 2D NOESY (*top, green*) and TOCSY spectra (*bottom, blue*) of A6–A11 Se‐glargine. Dashed rectangular box in *red* indicates exchange pattern between monomer and dimer amide protons (residue Glu^B21^) observed in TOCSY spectrum. Similar NOE patterns from the monomer and the dimer indicated by dashed lines in *black* provided further evidence of monomer‐dimer equilibrium of Se‐glargine in the protein concentration of 60 μM in 10‐mM deuterated acetic acid at pH 3 and at 25°C.
**Figure S21**. Homonuclear 2D‐NMR spectra of glargine (*left panel, black*) and Se‐glargine (*right panel, blue*): (a) NOESY spectra (mixing time 150 ms) showing NOEs from aromatic protons to methyl protons and (b) TOCSY spectra (mixing time 55 ms) showing aromatic resonance correlation. Spectra were acquired at a ^1^H frequency of 700 MHz in 10‐mM deuterated acetic acid (pH 3.0, direct meter reading) at 25°C.
**Figure S22**. Structure representation of A6–A11 bridges buttressed hydrophobic residues Leu^B6^, Leu^B11^, Ile^A2^ and Leu^A16^, in turn connected to the interfacial residues Leu^B15^, Phe^B24^ and Ty^rB26^ at the dimer interface. (a) Se‐glargine in the ensemble and (b) in a representative stick model. The A chain is *green* and B chain *blue*. Methyl groups represented as *green* or *blue*, diselenide bridge as *violet* spheres. (c) Glargine in the ensemble and (d) in a representative stick model. The A chain is *cyan* and B chain *gray*. Methyl groups represented as *cyan or gray*, A6–A11 disulfide bridge as *violet* spheres.
**Figure S23**. Monomer‐dimer exchange rate build‐up curve fitting for selected residues (*black*: glargine; *blue*: Se‐glargine). Exchange peaks between monomer and dimer amide protons were directly integrated in 2D exchange spectra with different mixing time. The protein concentration was 100 μM of glargine (*black*) and 84 μM of Se‐glargine (*blue*). The exchange rate of monomer‐dimer obtained by global fitting is 0.082(±0.012) ms^−1^ for glargine and 0.057(±0.013) ms^−1^ for Se‐glargine. Lifetimes are 12.3(±2.6) ms and 17.5(±3.8) ms, respectively. Data were acquired at a ^1^H frequency of 700 MHz in H_2_O (10% D_2_O) in 10‐mM deuterated acetic acid (pH 3.0, direct meter reading) at 25°C.
**Figure S24**. (a) Stereo view of structure representation of the invariant Ile^A2^ residue pinned the A1–A8 α‐helix to the hydrophobic core. The A chain is shown in green, B chain in blue, A6–A11 diselenide bridge in violet. Methyl groups represented as *green* (A chain) or *blue spheres* (B chain). (b, c) Secondary shift of alpha‐carbon and alpha‐protons of A‐chain residues (*panel b*) and B‐chain residues (*panel c*) for Se‐glargine (*blue*) and glargine (*black*) in 10‐mM deuterated acetic acid at pH 3.0 (direct meter reading) at 25°C. dashed boxes indicated residues that are sensitive to selenium replacement at A6 and A11 position. A6–A11 diselenide bond induced ^1^H_α_, ^1^H_N_ and ^13^C_α_ chemical shift of these residues changing toward alpha‐helix direction.
**Figure S25**. (a) Stereo view of structure representation of A6–A11 bridge‐related hydrogen bond (PDB‐ID: 4INS). The A chain is in *light pink*, B chain in *light blue* and A6–A11 disulfide bridge in *gold*. The amide protons of A11 and B6 are shown in *green spheres*, the oxygen atoms of B4 and A6 in *red spheres*. Local environment of Sec^A11^HN‐Gln^B4^CO H‐bond (b) and Leu^B6^HN‐Sec^A6^CO H‐bond (c). The color code is same as in panel (a) and dashed lines indicate hydrogen bonds. Successive 1D ^1^H NMR spectra of A6‐A11 diselenide mutant (*panel* d, *maroon*) and glargine (*panel* e, *black*) in far downfield region at the stated time points after dissolving the protein in 100% D_2_O in 10% deuterated acetic acid (pH 2.1, direct meter reading) at 25°C. The 1D data fitting curves of amide‐proton ^1^H‐^2^H exchange for Sec/Cys^A11^ residue (*panel* f) and Leu^B6^ residue (*panel* g) in 10% deuterio‐acetic acid at pH 2.1 at 25°C.
**Figure S26**. (a) The spectral trace of first 2D‐TOCSY after adding fresh D_2_O for ^1^H–^2^H exchange in 10% deuterated acetic acid at pH 2.1 at 25°C: Ser^A9^ H_α_/H_N_ cross‐peak of glargine (*left panel, black*) and Se‐glargine (*middle panel, maroon*). The *right panel* showed first 1D spectrum of Se‐glargine to display amide proton signal of Val^A3^. The RMSD noise level was measured in the spectral region between *blue* dashed lines. (B) The 2D data fitting curves of amide‐proton ^1^H–^2^H exchange for Ser^A9^ in glargine (*left panel, black*) and Se‐glargine (*middle panel, maroon*). The *right panel* was 1D data fitting of Val^A3^ in Se‐glargine.
**Figure S27**. Representative examples of exponential ^1^H–^2^H exchange at specific residues associated with subglobal‐ or global exchange kinetics as defined in the glargine insulin (*black*) and Se‐glargine (*blue*). Data were analyzed by 2D amide‐proton ^1^H–^2^H Exchange spectra in 10‐mM deuterated acetic acid (pH 3.0, direct meter reading) at 25°C.
**Figure S28**. Representative examples of exponential ^1^H–^2^H exchange 10% deuterated acetic acid at specific residues associated with subglobal‐ or global exchange kinetics as defined in the glargine insulin (*black*) and Se‐glargine (*maroon*). Analysis of global exchange corroborates with increase in Δ*G*_u_ due to selenium incorporation at Cys^A6^ and Cys^A11^ sites.
**Figure S29**. Complementary MD distance analysis for reported NOEs (Table S7). Split‐violin plots show pooled NOE‐relevant interproton distance distributions for WT glargine and Se‐glargine (A6–A11 diselenide) from 10 independent 1‐μs MD replicas per system. Distances are reported as NOE‐like effective distances, *r*_scaled_ = ⟨*r* ^− 6^⟩^−1/6^ thereby weighting shorter distances more strongly. WT is shown on the left half (gray) of each violin and Se‐glargine on the right (red). Methyl groups were treated by three‐proton averaging prior to distance evaluation. Panels are organized by the residue of the first proton in each pair and separated into A‐ and B‐chain parts. The plots provide NOE‐relevant distance *proxies* rather than full back‐calculated NOEs.
**Figure S30**. T‐R conformational transition. Structures: (a) T_6_ hexamer (PDB‐ID: 4INS), (b) T_3_R^f^_3_ hexamer (PDB‐ID: 1TRZ), (c) R_6_ hexamer (PDB‐ID: 1ZNJ). (d) Overlay of T‐state and R‐state monomer with respect to A12–A20 and B9–B19 helices show tilting and rotation of A1–A8 helix as previously shown by Chothia and colleagues (inset, adapted from Chothia et al. 1983). (e) Close view from the overlay with key residues and A6–A11 diselenide bridge highlighted. Color coding: A chain (pink), B chain (blue), methyl groups and disulfides (gold) are shown as spheres with one third van der Waals radii.
**Figure S31**. Structural accommodation and packing consequences of a modeled A6–A11 diselenide within an insulin fibril scaffold. (***a***) cryo‐EM insulin fibril model (PDB‐ID: 8SBD; Wang et al. 2023) showing two representative protomers and the three native bridges (A6–A11, A7–B7, A20–B19). The A‐ and B‐chains are shown as dark gray and white ribbons, respectively, and bridges are shown as spheres. A diselenide analog at A6–A11 was generated by PyMOL replacement and torsion (*χ*_3_) adjustment to increase the chalcogen–chalcogen distance from the native S–S value (~2.06 Å) to a Se–Se value (~2.35 Å), while leaving A7–B7 and A20–B19 unchanged. The coordinate axis indicates the viewing orientation, with the xz‐plane corresponding to the “top” view. **(*b–e, h–l*)** *Protomer 1* close‐ups of the A6–A11 region comparing S–S (*b,d,h*) versus Se–Se (*c,e,i*); neighboring side chains (purple; e.g., Gln^A5^ and Ser^A9^) indicate sufficient local free volume to accommodate the longer Se–Se bridge without obvious steric clashes in this ad hoc model. **(*f–g*)** Equivalent views for protomer 2 and the adjacent packing environment (purple; e.g., Glu^A4^ and Ser^A12^), again showing that the Se–Se model can be placed without apparent interprotomer interference. **(*j–m*)** Construction of a walled, rectangular cuboid “container” using dummy atoms (yellow spheres, *r*_vdW,wall_ = 0.8 Å) around the A6–A11 region and four anchor atoms (Gln5[CB], Ser12[OG], Ile10[CA], and Cys7[CA]) to define the cuboid corners; top views (*j,k*) and side views (*l,m*) are shown for S–S and Se–Se models, respectively. (***n–q***) MoloVol void analysis within the same container geometry (probe radius 0.20 Å; grid spacing 0.075 Å; identical settings except chalcogen vdW radii of 1.80 Å for S vs. 1.90 Å for Se; Bondi, 1964) reveals a small but reproducible reduction in probe‐excluded void space for the Se–Se model (***n,o***; with corresponding side views p,q), while preserving interlayer separation and showing no interlayer Se···Se contacts. Blue arrows and guidelines are shown to highlight subtle changes in void volume. (***r–u***) MD simulations of the 5‐layer fibril model containing the A6–A11 Se–Se substitution (only the three interior layers shown for clarity): overlays of the starting structure (gray = WT; yellow = Se–Se^A6–A11^) and the structure after 0.5 μs from the top (*r,s*) and side (*t,u*) views show an intact fibril architecture without layer detachment, consistent with only modest local packing adjustments near the A6–A11 site. It is important that only direct comparisons should be made between matched S–S vs. Se–Se panel pairs generated under the same view and settings (e.g., *b* vs. *c, d* vs. *e, f* vs. *g, h* vs. *i, j* vs. *k, l* vs. *m, n* vs. o, and *p* vs. *q*).
**Figure S32**. PyMOL mutagenesis‐based visualization of severe fibril‐disrupting substitutions in the insulin cryo‐EM fibril scaffold (PDB–ID: 8SBD; Wang *et al*., 2023), shown to contrast the much subtler S → Se^A6–A11^ substitution. Panels (*a*) and (*b*) show the two protomer contexts within the fibril model, with the three reported substitutions highlighted (labeled **1**–**3**). Numbered zoom panels (**1**–**6**) show local packing around each mutation site in the two protomers. In the inserts, neighboring residues are shown in green; the corresponding WT side chain is shown in yellow in nearby fibrils and magenta which had the substitution); and the modeled mutant rotamer with the lowest PyMOL clash score is shown in red in (*a*) and white in the insert panels. PyMOL clash indicators are displayed as red spheres/cylinders (severe clashes) and yellow cylinders (minor clashes). These substitutions represent substantially larger and/or charge‐altering perturbations (two include charge changes), and all three occur at internally packed fibril positions rather than solvent‐exposed sites, making local accommodation and reorganization more energetically costly. The visualized clashes are shown for a *single mutation* event in one local filament environment; in the full repeating fibril assembly, analogous steric and electrostatic penalties would be propagated across stacked layers, further reducing feasibility of maintaining the same packing arrangement. This behavior contrasts with the modeled A6–A11 diselenide substitution, which is a comparatively subtle geometric perturbation (primarily a longer Se–Se bond with modest steric expansion) and is therefore considered more compatible with fibril formation, consistent with experimental evidence indicating *increased lag time* rather than complete suppression of fibrillation.
**Figure S33**. A6–A11 χ_3_ torsion in WT glargine versus Se‐glargine from 1‐μs simulations with expanded replica sampling. The χ_3_ dihedral of the A6–A11 bridge, defined as C_β_(A6)–**X**(A6)–**X**(A11)–C_β_(A11) (**X** = S for glargine; **X** = Se for Se‐glargine), is plotted in degrees. Ten independent 1‐μs replicas were analyzed for each system (*r*_1_–*r*_10_). (a) Split violin plots show the χ_3_ distribution for each replica (WT, black; Se‐glargine, red). (b) Replica‐matched χ_3_ timelines show the time evolution of χ_3_ over 1 μs; purple arrows (**⇑**) mark WT transitions between the two major χ_3_ substates. (c) Representative structures from replica *r*_2_ define these substates: *s*_1_ (χ_3_ ≈ 247°), sampled only by WT, and *s*_2_ (χ_3_ ≈ 279‐290°), sampled by WT and Se‐glargine. Thus, the WT disulfide can adopt both *s*_1_ and *s*_2_, whereas the Se‐glargine diselenide is restricted to *s*_2_, because the *s*_1_ geometry is incompatible with the local packing environment and would clash with Leu^A16^.
**Figure S34**. Nearest‐neighbor contacts and van der Waals packing at the A6–A11 bridge in 4INS: WT Cys–Cys (S–S, top) versus Sec–Sec (Se–Se, bottom). (a) A ribbon overview with the A chain colored dark gray and the bridges shown as sticks (orange and dark orange for S vs. Se, respectively) with nearby residues shown in purple. (b) Heavy‐atom nearest neighbors to the bridge (contact cutoff in Pymol, 4.5 Å) shown as sticks and labeled in purple. (c) Space‐filling models of the local pocket with spheres depict atomic van der Waals radii (S in yellow, Se in orange and at scale, 10% increase). The Se–Se bridge preserves the overall geometry of A6–A11 but increases local steric occupancy, shifting hydrophobic packing among nearby side chains and reducing small voids adjacent to the bridge.
**Figure S35**. Container‐based analysis of local packing around the A6–A11 bridge using MoloVol. (a) Placement of an 11‐Å cubic container composed of dummy atoms, centered on the CD1 atom of Leu^B11^ in the crystallographic 4INS scaffold; the same container geometry is used for all subsequent calculations. Panels (b) and (c) show cross‐sections of the probe‐excluded void within this cube for the Cys^A6–A11^ disulfide (S–S; panel b) and Sec^A6–A11^ diselenide (Se–Se; panel c) variants of 4INS, with the absolute void volumes and their Se–S difference (≈5.9 Å^3^) indicated. (d) Equivalent container placement for WT glargine after superposition of its A chain onto the A chain of 4INS to ensure that the cube samples the same spatial region in the analog. Panels (e) and (f) show the corresponding analysis for WT glargine (A6–A11 disulfide; panel e) and Se‐glargine (A6–A11 diselenide; panel f), again reporting the cavity volumes within the cube and the S to Se difference (≈6.4 Å^3^).^[*a*]^ In all cases, void volumes were computed with MoloVol using identical probe radius (0.20 Å), grid spacing (0.075 Å) but with a different van der Waal (vdW) radii (1.80 *vs*. 1.90 for Se), so that the observed differences report changes in local packing around the A6–A11 bridge. Reference ^[*a*]^ (Maglic and Lavendomme, 2022).
**Figure S36**. Expanded analysis of the χ_3_ dynamics and χ₂ to χ₃ correlations for the A6–A11 bridge in WT glargine and Se‐glargine. (a) Timelines of χ_3_ (C_β_–X–X–C_β_; X = S or Se) for the A6–A11 bridge in replica 4 over 400 ns (20,000 frames). Shaded traces show instantaneous dihedral values, and solid curves represent 100‐frame running averages (WT glargine in gray, Se‐glargine in red). Replica 4 interconverts between two χ_3_ basins (“state 1” → “state 2”; colored bars below the axis). (b) Corresponding χ_3_ timelines for replica 2, which remains confined to a single basin (state 2) for both WT and Se‐glargine and thus serves as a comparison trajectory without a detectable transition. (c) Pearson correlation coefficients (*ρ*) between χ_2_ side‐chain dihedrals of each residue and χ_3_(A6–A11) for WT glargine, computed for the early portion of replica 4 dominated by state 1 (frames 0–2,999). (e) As in (c), but for the later portion dominated by state 2 (frames 5000–19,999). (d) χ_2_–χ_3_ correlation pattern for WT glargine in the transition window of replica 4 (frames 2500–5,499), highlighting residues with moderate positive correlations (vertical orange dashed lines) primarily in the B chain's detachable tail and near the A6–A11 region. (f) Corresponding χ_2_–χ_3_ correlations for Se‐glargine over the same transition window; correlations are generally attenuated relative to WT, consistent with damping of propagated side‐chain rearrangements by the A6–A11 diselenide staple. In panels (c–f) the B chain (B1–B32) and A chain (A1–A21) are separated by a vertical purple line, and horizontal dashed lines mark moderate Pearson's correlations of *ρ* = ±0.25. See Section S2.3 for additional information on Pearson R correlations.
**Figure S37**. Pearson correlations of A6–A11 χ_3_ with backbone and side‐chain dihedrals over the full trajectories. Pearson correlation coefficients (*ρ*) between the χ_3_ dihedral of the A6–A11 disulfide/diselenide bridge and χ_1_, χ_2_, φ, and ψ angles of each residue are shown for replica 4 of WT glargine (left, black bars) and Se‐glargine (right, red bars). Rows correspond to χ_1_ vs χ_3_, χ_2_ vs χ_3_, φ vs χ_3_, and ψ vs χ_3_, respectively. The *x*‐axis lists A‐ and B‐chain residues; the vertical purple line marks the A/B chain boundary. Vertical orange dashed lines highlight residues in the A‐chain helix and selected B‐chain positions that show a moderate or larger correlation. Horizontal magenta dashed lines indicate |*ρ*| = 0.25 as a visual guide for modest correlations. Correlations are computed over all 20,000 frames of the trajectories (“all frames”). See Section S2.3 for additional information on Pearson R correlations.
**Figure S38**. Pearson correlations of A6–A11 χ3 with backbone and side‐chain dihedrals during the χ_3_ “break” window. As in Figure S37, Pearson correlation coefficients (*ρ*) between the A6–A11 χ_3_ dihedral and χ_1_, χ_2_, φ, and ψ angles of each residue are shown for replica 4 of WT glargine (left, black bars) and Se‐glargine (right, orange bars). Here, correlations are calculated only for frames 2500–5499, the window encompassing the χ_3_ switching (“break”) event in WT (“frames 2500 to 5499”). Plot organization, residue labels, and reference lines are identical to the previous figure (purple A/B chain boundary, vertical orange dashed lines highlight residues in the A‐chain helix and selected B‐chain positions that show a moderate or larger correlation, magenta horizontal dashed lines at |ρ| = 0.25). Within this restricted window WT shows slightly accentuated local correlations near the bridge, whereas Se‐glargine—whose χ_3_ remains in a single rotamer—continues to exhibit generally weak χ_3_ coupling across the scaffold. See Section S2.3 for additional information on Pearson R correlations.
**Figure S39**. Time‐series of side‐chain torsions for the A6–A11 bridge comparing WT glargine (black) and Se‐glargine (red). The A7–B7 and A20–B19 disulfide bridges were also monitored. Panels show example dihedral angles (χ_3_) as labeled in the plots; units°. Four 400‐ns replicas per system are concatenated left‐to‐right; vertical black lines mark replica boundaries. Faint traces are raw frame values; bold curves are running mean averages over 100 frames.
**Figure S40**. Distributions of disulfide/diselenide torsion angles (χ_3_; C_β_–X–X–C_β_) from MD for glargine (WT, black) and Se‐glargine (Sec^A6^–Sec^A11^) (red). Each panel shows split‐violin plots per replica (*r*_1_–*r*_4_) for one bridge: Cys/Sec^A6–A11^ (X = S in WT, Se in mutant), Cys^A7^–Cys^B7^, and Cys^A20^–Cys^B19^ (X = S in both). Each violin summarizes all frames of the corresponding trajectory; *y*‐axis is dihedral angle (degrees). Selenium substitution at A6–A11 shifts the χ_3_ distribution relative to WT, while A7–B7 and A20–B19 remain similar across replicas.
**Figure S41**. Structural comparisons of native insulin glargine: (a, b) crystal structure (PDB‐ID: 4IYD) *versus* respective solution structures in 10% dAA (PDB‐ID: 9ZO6 herein (a)) or 20% dAA (PDB 6K59 (b)) as described (Ratha et al. 2020); and (c, d) alignment of the two solution structures. (a) Overlay of a representative solution structure in 10% dAA (present work) and a crystallographic protomer, as aligned in the region of A12–A19 and B9–B19. The RMSD is 0.6 Å. In the NMR‐derived structure the A chain is shown in cyan, and the B chain in gray. In the crystal structure the A chain is shown in light pink, and the B chain in light blue. In each case disulfide bridges are shown in gold (spheres). (b) Analogous overlay of a representative solution structure in 20% dAA and the crystal structure. The RMSD is 1.8 Å. In the prior NMR‐derived structure the A chain is shown in wheat, and the B chain in smudge. The crystal structure is colored as in panel (a). (c) Overlay of respective solution structures in 10% and 20% dAA. The structures are aligned according to the main‐chain atoms of the three helices (residues A2–A8, A13–A19, and B10–B19). In the present structure (10% dAA) the A chain is shown in cyan, and the B chain in gray. In the prior structure (20% dAA) the A chain is shown in wheat, and the B chain in light blue. The sulfur atoms in disulfide bridges are shown in gold (spheres). (d) Overlay of C_α_ traces of respective NMR‐derived structures: thick black line represents the structure determined in 20% dAA (model 1 in PDB‐ID: 6K59) and thin lines, the structure presently determined in 10% dAA C_α_ traces of 20 NMR structures. The color code is otherwise the same as in panel (c).
**Figure S42**. Workflow for implementing a Se–Se diselenide bridge in CHARMM (topology + parameters) (a) **Topology**. Starting from the standard CHARMM36 disulfide model (**1**), input starting structures; RESI: CYS; PATCH: DISU), we use the existing CHARMM36 CYS residue and DISU patch topology/connectivity as the reference template (**2**). We then introduce the selenium analogs needed to build a diselenide (**3**): (i) a selenocysteine residue template (RESI: SEC) in which the side‐chain chalcogen is renamed and retyped from S/SG to Se/SE (atom type SX1), and (ii) a diselenide patch (PATCH: DSEC) that mirrors DISU connectivity but forms an Se–Se bridge (atom type SX2). Separate CYS/SEC residue definitions are required because CHARMM constructs the bridge via a PATCH that removes the chalcogen H‐atom (HG1) and connects the two side chains. The used bonded terms are collected in Tables S17–S21 for straightforward porting into a CHARMM‐readable parameter file (e.g., *missing_params.prm* that can be easily read by CHARMM). (b) **Parameters**. Bonded terms for the selenium‐containing types (**4**; bonds/angles/dihedrals) are mapped from the corresponding CHARMM36 cysteine/disulfide terms because the topology is the same, and these are collected in Tables S17–S21 for direct porting into a CHARMM‐readable parameter file. Non‐bonded Lennard‐Jones parameters and selenium mass (**5**) are then assigned for the Se atom types SX1 (residue Se) and SX2 (bridge Se) (Table S22). For SEC, non‐bonded parameters are taken from a reference selenium compound (e.g., CH_3_SeH) and used directly for SX1 (**6**), whereas for the diselenide bridge the LJ size is intentionally enlarged for SX2 to reflect the bulkier bridge environment (**7**). Partial charges were adjusted minimally relative to the sulfur and reference selenol/selenocysteine templates to preserve the overall neutral character of CYS/DISU and SEC/DSEC. Non‐bonded sizes were generated using a two‐step scaling (**8**): first setting the residue selenium size via scaling relative to cysteine sulfur (*f*_1_, see panel **8a**) and then defining the bridge selenium by applying the same scaling again (*f*_2_, see panel **8b**), yielding 2.42 Å (see also Figure S7). This “extra” scaling is intended to reflect the bulkier and more polarizable bridge environment. The same inset (8) also provides the conceptual comparison used for PyMOL visualizations, illustrating the slightly larger van der Waals radius/LJ contact distance for Se vs. S and summarizing typical homolytic bond dissociation energy ranges (S–S ≈ 240–270 kJ·mol^−1^; Se–Se ≈ 170–200 kJ·mol^−1^; supporting the qualitative expectation that Se–Se is longer/weaker (“softer” or more polarizable) than S–S. Circle sizes illustrate the slightly larger van der Waals radius of selenium relative to sulfur (*r*_(S)_ ≈ 1.80 Å; *r*_(Se)_ ≈ 1.90 Å), with corresponding Lennard‐Jones contact distances (*R*_min_/2) of ≈ 2.00 Å for Cys sulfur and ≈ 2.20 Å for Sec selenium. Colored dots mark the centers from which these distances are measured. The LJ potentials used in simulations are illustrated for CYS vs. SEC and DISU vs. DSEC (**9**), and final parameters are validated by short MD tests and inspection of structural stability/motions (**10**). References: [*a*] (Pedron et al. 2023); [*b*] (Bondi, 1964); [*c*] (Kildahl, 1995; Sousa et al., 2019).
**Figure S43**. (a) Split violin plots of χ_2_ (C_α_–C_β_–C_γ_–C_δ_) side‐chain dihedral angles for leucine residues surrounding the A6–A11 bridge in WT glargine (black, left half of each violin) and Se‐glargine (red, right half), pooled over replicas 1–4. Distributions for Leu^A16^, Leu^B6^, Leu^B11^, and Leu^B15^ report whether rigidification of the A6–A11 diselenide propagates into neighboring hydrophobic side chains; only modest shifts and local narrowing are observed relative to WT. (b) Distributions of interatomic distances from MD simulations of WT glargine (Cys^A6^–Cys^A11^, black) and Se‐glargine (Sec^A6^–Sec^A11^, red). C_α_⋯C_α_ distance between Cys/Sec^A6^ and Leu^B6^. (c) C_α_⋯C_α_ distance between Gln^B4^ and Cys/Sec^A11^. Each pair of violins corresponds to an individual replica (*r*_1_–*r*_4_). Se‐glargine shows a modest compaction and narrowing of the B6–A6 distance distribution, but a broader and more heterogeneous B4–A11 distance distribution, indicating local stabilization near A6 and increased flexibility at the B4–A11 contact.
**Table S1**. Thermodynamic Stabilities (reproduced from prior publication for convenience of the reader from Weil‐Ktorza et al., 2024).
**Table S2**. Deconvolution of secondary structures from CD spectra.
**Table S3**. Backbone chemical shift perturbation induced by A6–A11 diselenide bridge in Se‐glargine. The ^1^H_N_/^1^H_α_ chemical shift difference bigger than 0.05 ppm and ^15^N/^13^C_α_ chemical shift difference bigger than 0.30 ppm are listed in red. NMR data were acquired in 10% deuterated acetic acid at pH 2.1 and 25°C.
**Table S4**. NOE lists that were only observed in Se‐glargine or in glargine (*red*) and that probably resulted in chemical shift degeneracy (*black*).
**Table S5**. Statistics of experimental data and structure calculations of glargine.
**Table S6**. Statistics of experimental data and structure calculations of Se‐glargine.
**Table S7**. NOE cross‐checking violations using the NOE distance restraints of Se‐glargine to check 20 NMR structures of glargine and that using the NOE distance restraints of glargine to check 20 NMR structures of Se‐glargine. The # indicated the chemical shift degeneracy. The number in the brackets was the structure number with NOE violation. The threshold for NOE violation was set up as 0.5 Å.
**Table S8**. Side‐chain chemical shift perturbation greater than 40 Hz induced by A6–A11 diselenide bond. NMR data were acquired in 10% deuterated acetic acid at pH 2.1 and 25°C.
**Table S9**. Monomer–dimer exchange rate of glargine and Se‐glargine measured by 2D exchange spectra in 10 mM deuterated acetic acid at pH 3.0 (direct meter reading) and 25°C.
**Table S10**. The protein monomer–dimer ratios and dimerization constants of Se‐glargine and glargine measured by ^1^H, ^13^C‐HSQC spectra in different protein concentration. Data were obtained at a 1H frequency of 700 MHz in 10 mM deuterated acetic acid (pH 3.0, direct meter reading) at 25°C.
**Table S11**. Main‐chain ^15^N, ^1^HN, ^13^C_α_, ^1^H_α_ chemical shifts, methyl ^13^C and ^1^H chemical shifts and corresponding secondary chemical shifts of Ile^A2^ in Se‐glargine and glargine in 10% deuterio‐acetic acid at pH 2.1 (direct meter reading) and at 25°C.
**Table S12**. Secondary ^13^C_α_ and ^1^H_α_ chemical shift of Se‐glargine and glargine in 10% deuterated acetic acid at pH 2.1 and 25°C.
**Table S13**. Backbone ^1^H_N_ and ^1^H_α_ chemical shift of Se‐glargine and glargine in 10 mM deuterated acetic acid at pH 3.0 (direct meter reading) and 25°C.
**Table S14**. ^13^C_α_, ^13^C_β_ and secondary ^13^C chemical shift of Se‐glargine and glargine in 10 mM deuterated acetic acid at pH 3.0 (direct meter reading) and 25°C.
**Table S15**. HD‐exchange results of glargine and Se‐glargine in 10 mM dAA at pH 3.0 and 25°C.
**Table S16**. HD‐exchange results of glargine and Se‐glargine in 10% dAA at pH 2.1 and 25°C.
**Table S17**. Supplementary table of adjusted and missing *residue topology* and partial charges relevant to the structural characterization of selenocysteine (RESI SEC).
**Table S18**. Supplementary table of adjusted and missing residue *topology* and partial charges relevant to the structural characterization of the diselenide bond patch (PRES DSEC).
**Table S19**. Supplementary table of adjusted and missing *bonding* parameters relevant to the structural characterization of selenocysteine residues and diselenide bonds.
**Table S20**. Supplementary table of adjusted and missing *angular* parameters relevant to the structural characterization of selenocysteine residues and diselenide bonds.
**Table S21**. Supplementary table of adjusted and missing *dihedral* parameters relevant to the structural characterization of selenocysteine residues and diselenide bonds.
**Table S22**. Supplementary table of adjusted and missing *nonbonded* and mass parameters relevant to the structural characterization of selenocysteine residues and diselenide bonds.
**Table S23**. Supplementary table of measured interatomic (C_α_⋯C_α_) distances in the conformers (frame #0001) of reported Aaron Dinner's islands named **C0–C9**. Classification is binned into “proximal” and “distant” with value‐ranges from 0 to 7.0 Å and >7.0, respectively.
**Data S1**. Supplemental Discussion.
**Data S2**. Supplemental Methods.

## Data Availability

The data that support the findings of this study are openly available in Protein Data Bank at https://www.rcsb.org.
