# Supplementary material for: Damping amyloid‐associated conformational fluctuations in a protein by an engineered diselenide bridge
Source: Protein Sci. 2026 Jul 21;35(8):e70697. doi: 10.1002/pro.70697 (PMC13386395; doi:10.1002/pro.70697)
Supplement: Supplementary file 1 — Figure S1. A schematic energy landscape for protein folding and aggregation. Figure adapted from (Jahn and Radford, 2005). The surface shows the multitude of conformations ‘funneling’ towards the native state via intramolecular contact formation, or towards the formation of amyloid fibrils via intermolecular contacts. Figure S2. Overview of ion‐dependent aggregation pathways. Reproduced from publication by Lenton et al. (2025). Sulfate promotes a colloidal pathway, forming folded aggregates that later convert to β‐rich amyloids and can undergo phase separation or crystallization (top). Perchlorate yields an intermediate pathway (middle), whereas chloride enforces a conformational pathway in which unfolding drives formation of elongated β‐sheet fibrils (bottom). Figure S3. Pharmacokinetic profile of insulin injections: onset, peak activity, and duration of action (adapted from Petznick, 2011). Figure S4. Prior strategies for improving insulin stability involved linking the B‐ and A‐chains with short peptide connectors and introducing an additional disulfide bond. (a) NMR structure of single chain insulin (PDB‐ID: 2LWZ). (b) X‐ray crystal structure of four‐disulfide insulin (PDB‐ID: 4EFX). Color coding: A chain (pink), B chain (blue), C domain (green), disulfides are shown as gold spheres with one third van der Waals radii. Figure S5. Conformational “islands” C0–C9 of monomeric human insulin as defined by Dinner and co‐workers (Busto‐Moner et al. 2021). The central structure shows the A chain (dark gray) and B chain (light gray) with the B‐chain N‐terminal segment B1–B8 (green), C‐terminal segment B20–B30 (red), and the A‐chain disulfide CysA6–CysA11 (burnt orange). Surrounding panels depict representative structures from each island (C0–C9) projected around the circle, colored with the same scheme. Labels and percentages indicate the corresponding island identity and fractional population in the conformational ensemble. Figure S6. High conservation within the core. ( [file PRO-35-e70697-s001.pdf]

Supplemental Information

for

Damping amyloid-associated conformational fluctuations in a protein by an engineered diselenide bridge

Yanwu Yang[1^], Balamurugan Dhayalan[1^], Andreas Ehnbom[1], Orit Weil-Ktorza[2], Norman Metanis[2], & Michael A. Weiss[1*]

Purpose of Supplement

The Supporting Information (SI) contains 43 Supplemental Figures (Figures S1-S43) and 23 Supplemental Tables (Tables S1-S23). Additional references are provided and an expanded Supplementary Discussion Sections (S1.1-S1.5) and Expanded Methods Sections (S2.1-2.3). The purpose of the SI is to provide:

(i) extended experimental characterization and controls that underlie the main conclusions, including analytical monitoring and stability measurements (*e.g.*, HPLC tracking of pepsin cleavage and temperature-dependent spectral scans) and expanded NMR analyses supporting structural interpretations (HSQC/NOESY/TOCSY, monomer-dimer exchange behavior, and residue-specific H/D-exchange kinetics);

(ii) mechanistic structural context around the engineered A6–A11 substitution (packing/contacts and local cavity/volume analyses); and

(iii) detailed molecular-dynamics (MD) post-processing and visualization across multiple replicas, including time-series and distribution-based analyses. In addition, the SI documents the full CHARMM implementation of the Se–Se diselenide bridge (topology + parameters) and provides tabulated bonded and nonbonded terms (including LJ and mass parameters) to enable direct reproducibility and porting.

Table of Contents

S1. Supplemental Discussion

S1.1. Dynamic probes of low-abundance species *3*
S1.2. Complementary strategies to stabilize pharmaceutical formulations of insulin *4*
S1.3. Redox properties of selenocysteine and Se-insulin chemistry *6*
S1.4. MD analysis reveals localized rigidification by the A6-A11 diselenide *8*
S1.5. Fibril-state structural analysis and simulations *10*

S2. Supplemental Methods

S2.1. EX2 Hydrogen Exchange Formalism 10
S2.2. Computational Details: Pearson R correlations 11
S2.3. Computational Details: Se–Se Diselenide Implementation in CHARMM 11

S3. Supplemental Figures

Figure S1 .... *4*
Figure S2 .... *6*
Figure S3 .... *12*
Figure S4 .... *12*
Figure S5 .... *13*
Figure S6 .... *14*
Figure S7 .... *15*
Figure S8 .... *16*
Figure S9-S10 .... *17*
Figure S11 .... *18*
Figure S12 .... *19*
Figure S13 .... *20*
Figure S14 .... *21*
Figure S15 .... *22*
Figure S16 .... *23*
Figure S17 .... *24*
Figure S18 .... *25*
Figure S19 .... *26*
Figure S20 .... *27*
Figure S21 .... *28*
Figure S22 .... *29*
Figure S23 .... *30*
Figure S24 .... *31*
Figure S25 .... *32*
Figure S26 .... *33*
Figure S27 .... *34*
Figure S28 .... *35*
Figure S29 .... *36*
Figure S30 .... *37*
Figure S31 .... *38-39*
Figure S32 .... *40*
Figure S33 .... *41*
Figure S34 .... *42*
Figure S35 .... *43*
Figure S36 .... *44*
Figure S37 .... *45*
Figure S38 .... *46*

Figure S39 47
Figure S40 48
Figure S41 49
Figure S42 50
Figure S43 51

S4. Supplemental Tables
Table S1 52
Table S2 53
Table S3 54
Table S4 55
Table S5 56
Table S6 57
Table S7 58
Table S8 59
Table S9 60
Table S10 61
Table S11 62
Table S12 63
Table S13 64
Table S14 65
Table S15 66
Table S16 67
Table S17 68
Table S18 69
Table S19 70
Table S20 71
Table S21 72
Table S22 73
Table S23 74

References 75

S1. Supplemental Discussion

S1.1. Dynamic probes of low-abundance species

In the present study the term "protein dynamics" pertains to multiple time scales, from subnanosecond- and nanosecond-scale atomic fluctuations within the native conformation (*e.g.*, as probed by NMR-derived order parameters (Palmer III, 1993) and *in silico* by molecular dynamics (MD) simulations; Karplus, 1984) to microsecond-scale motions involved in mechanisms of catalysis and from millisecond-scale conformational exchange among otherwise invisible "excited states" (Mulder et al., 2001) to even slower events, such as protein assembly and disassembly (Ben-Nissan and Sharon, 2011). Critical to the kinetics of protein fibrillation on a time scale of hours or days is its nucleation by low-abundance partial folds (Wetzel, 2006), reflecting segmental

motions at time scales slower than are typically probed by MD simulations with submicrosecond trajectories (see schematic below, Figure S1) highlighting various sub-populated species at the bottom of the wells). In the case of insulin, insight into such partial folds has been provided by longer replica-exchange MD simulations by Dinner and coworkers (Busto-Moner et al., 2021). These partial folds provide candidate excited states through which an amyloidogenic nucleus may be formed via aggregation of exposed nonpolar surfaces (Nielsen et al., 2001). To extend the lag time of an insulin analog, an engineered A6-A11 diselenide bridge might forestall the kinetic accessibility of one or more such partial folds, reducing their percent occupancy in the protein's conformational ensemble. We imagine that dynamic coupling occurs across multiple time scales relating (a) enhanced local packing efficiency around the A6-A11 bridge to (b) nanosecond-scale side-chain fluctuations in the core and in turn (c) rare larger-scale segmental unfolding events leading to Dinner excited states. Such damping of amyloid-associated conformational excursions is likely to affect segmental unfolding of the A1-A8 α-helix and detachment of the B24-B28 β-strand as illustrated in main-text Figure 1.

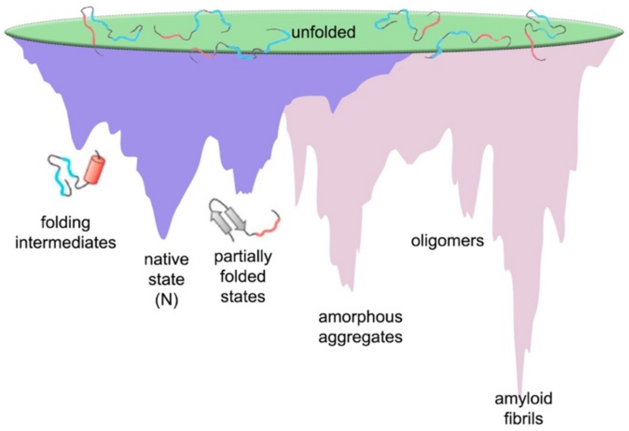

Figure S1. A schematic energy landscape for protein folding and aggregation. Figure adapted from (Jahn and Radford, 2005). The surface shows the multitude of conformations 'funneling' towards the native state via intramolecular contact formation, or towards the formation of amyloid fibrils via intermolecular contacts.

S1.2. Complementary strategies to stabilize pharmaceutical formulations of insulin

The susceptibility of insulin to fibrillation emerged in the immediate decades after its formulation as a major obstacle to both the hormone's manufacture from animal pancreata and its clinical formulation (Brange, 1987). Such practical obstacles led to pioneering studies of insulin amyloid between 1930 and 1960 (Waugh, 1941; Waugh, 1946; Waugh et al., 1953), eventually leading to the development of modern principles underlying the process of cross-β assembly (Jimenez et al., 2002) and the three-dimensional structure of an insulin fibril (Wang et al., 2023; Suladze et al., 2024). Instability of clinical formulations led to delays in the introduction of rapid-acting insulin analogs in the 1990s (Brange and Langkjaer, 1997) and continues to impose a global cold chain governing the transport, distribution and storage of insulin products (Heinemann et al., 2021). Here, we highlight

this translational motivation for the present study and describe two complementary approaches to forestall fibrillation under stressed conditions.

Reduced Zn^{2+} Binding at Low pH used in glargine formulations — Stabilization of insulin in neutral-pH formulations is mediated by zinc-dependent assembly, in which His^{B10} from each monomer coordinates Zn^{2+} to form a stable hexamer assembly (Dodson and Steiner, 1998). Under acidic conditions (pH 4) used for insulin glargine, this mode of assembly is disrupted, preventing efficient Zn^{2+} coordination and thereby preventing zinc-mediated hexamer formation. Therefore, glargine is more prone to fibrillation under formulation conditions. Introduction of an internal A6–A11 diselenide bridge in Se-glargine provides compensatory stabilization, offsetting the loss of zinc-mediated protection and reducing the propensity for fibril formation.

Relevance of TR transition to insulin pharmacology — Conformational changes such as the T→R transition have been exploited to stabilize neutral-pH pharmaceutical formulations (Figure S30a-c; for review, see Weiss, 2009). Whereas these conformational changes most prominently involve the B chain, the N-terminal A-chain α-helix is itself remarkable for segmental reorientation in the T→R transition (Figure S30d) (Chothia et al., 1983). Such reorientation is coupled to changes in the details of side-chain packing adjoining cystine A6-A11 (Figure S30e). The existence of alternative packing schemes reflects local micro-cavities in the hydrophobic core (Baker et al., 1988). The intrinsic dynamics of the T_6 insulin hexamer, including preexisting R-like conformations, foreshadows the long-range transmission of conformational change in the complete T→R transition (Choi et al., 1996).

Liquid-liquid phase separation — Recent studies by Lenton et al. (2025) focused on the effects of larger anions (relative to chloride), such as sulfate, on the fibril formation (Lenton et al., 2025). Binding of sulfate to the surface of insulin under acidic conditions stabilizes the native state and mitigates charge-charge repulsion between monomers or dimers (cf. Figure S2). Under these conditions, liquid-liquid phase separation becomes a major mechanism linking the protein-rich phase (a condensate or helix-rich aggregate) to the ultimate cross-beta assembly. In the present study, physical stability (propensity to form fibrils) of the insulin analogs was studied under dilute protein concentrations (60 μM) in 10 mM Tris-HCl (pH 4.0) and 140 mM NaCl. Under these conditions Lenton et al. (2025) categorize the mechanism of protein fibrillation as following the classical "conformational pathway", which does not involve liquid-liquid phase separation.

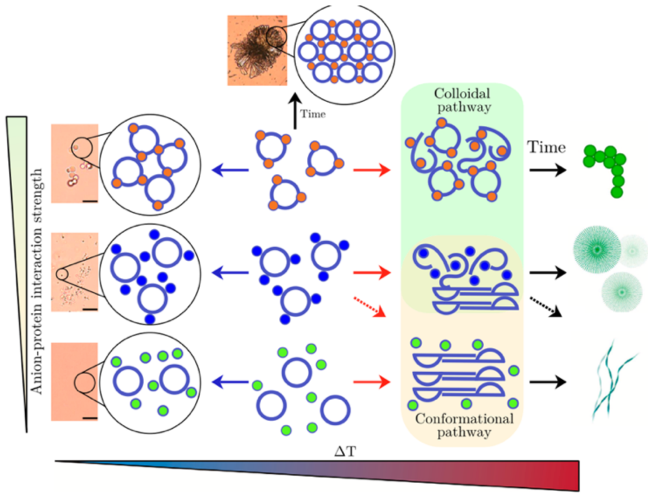

Figure S2. Overview of ion-dependent aggregation pathways. Reproduced from publication by Lenton et al. (2025). Sulfate promotes a colloidal pathway, forming folded aggregates that later convert to β-rich amyloids and can undergo phase separation or crystallization (top). Perchlorate yields an intermediate pathway (middle), whereas chloride enforces a conformational pathway in which unfolding drives formation of elongated β-sheet fibrils (bottom).

S1.3. Redox properties of selenocysteine and Se-insulin chemistry

Selenocysteine (Sec, U)[1], a near isostere of Cys and 21st-proteinogenic amino acid (Stadtman, 1996), has provided an elegant tool in peptide chemistry (Dawson, 2011) to enhance the efficiency of protein folding (Pegoraro et al., 1999; Metanis and Hilvert, 2012). Because the selenol group of Sec has a lower pK_a (near 5.2; Huber and Criddle, 1967) and lower reduction potential (E_0 = -388 mV) relative to the thiol group of Cys, pairwise Cys-to-Sec substitutions can enhance the rate and efficiency of oxidative folding (Armishaw et al., 2006; Gowd et al., 2010; Metanis and Hilvert, 2012; Dery et al., 2017). The present study exploits different aspects of selenium: its larger atomic size and longer bond lengths relative to sulfur. We hypothesized that such steric features (unrelated to redox chemistry) could mitigate cryptic packing defects in the hydrophobic core of a globular protein (Richards, 1974) and so in favorable cases enhance stability (Eriksson et al., 1992). For this purpose, insulin provides a favorable model of globular proteins because it (a) is amenable to total chemical synthesis, facilitating the introduction of non-standard amino-acid substitutions (Zaykov et al., 2016), and (b) has been extensively characterized in past studies by an unusually broad range of biochemical and biophysical methods (Baker et al., 1988; Jarosinski et al., 2021)—essential background for the present studies.

To circumvent the limitations of standard amino-acid substitutions, we previously undertook the chemical synthesis of an analog of human insulin in which an internal disulfide bridge (cystine A6-A11) was substituted by a diselenide bridge (Weil-Ktorza et al., 2019; Weil-Ktorza et al., 2024). The original study, motivated by the favorable redox properties of selenocysteine (Sec) with respect to its oxidative pairing (Hondal et al., 2013; Metanis and Hilvert, 2015; Mousa et al., 2017), demonstrated that the speed and efficiency of insulin chain

combination were markedly enhanced (Weil-Ktorza et al., 2019); nativelike structure and biological activity were retained. The original synthesis was thus motivated by the long-standing framework of Sec as a redox-active probe.

In the course of these studies, however, we unexpectedly observed that the A6-A11 diselenide bridge augmented the resistance of the analog (designated Se-insulin) to chemical denaturation by guanidine hydrochloride (Gu-HCl) and to reduction by dithiothreitol (DTT) at neutral pH (Weil-Ktorza et al., 2019). We recently extended these studies to an anomalous clinical analog refractory to classical chain combination (insulin *glargine*; Bolli and Owens, 2000; Weil-Ktorza et al., 2024). As highlighted in bold in the upper panel of Figure 1b in the main text, this analog contains an Arg-Arg extension of the B chain (thereby shifting its isoelectric point) and A-chain substitution $Asn^{A21} \rightarrow Gly$ (mitigating chemical degradation in an acidic formulation) (Bolli and Owens, 2000). As predicted, pairwise substitution of Cys^{A6} and Cys^{A11} by Sec (gold in upper panel of Figure 1b) partially rescued the yield of chain combination, presumably due to rapid and selective closure of the diselenide bridge early in the course of the reaction. The modified glargine (herein designated Se-glargine) retained native activity and (as in the context of human insulin) required higher concentrations of Gu-HCl to denature (as monitored by circular dichroism [CD]; C_{mid} 6 M versus 4.9 M, Table S1). Although insulin chain combination (pioneered for than 60 years ago; for review, see Katsoyannis, 1966) has been superseded by recombinant expression of single-chain insulin precursors as a scalable route to insulin manufacture (Thim et al., 1986; Chance and Frank, 1993; Kjeldsen et al., 2024), an intriguing problem is posed by the augmented resistance of Se-insulin and Se-glargine to chemical denaturation. Despite the prior CD-based studies of chemical denaturation (Weil-Ktorza et al., 2019; Weil-Ktorza et al., 2024), global thermodynamic stability (as probed by ^{1}H-^{2}H exchange) is not significantly enhanced; prior estimates of free energies of unfolding (ΔG_u) were based on a two-state model (Sosnick et al., 2000), which is unlikely to be valid given the insulin monomer's complex conformational equilibria of partial folds (Figure S5; Busto-Moner et al., 2021). The present reinvestigation of Se-glargine strongly suggests that interpretation of CD-detected guanidine titrations on the basis of a two-state model (Sosnick et al., 2000) provided an overestimate of its thermodynamic stability. We ascribe this overestimation to the presence of partial folds at intermediate concentrations of guanidine hydrochloride in broad accordance with the molecular dynamics simulations of Dinner and coworkers (Busto-Moner et al., 2021) (see Figure 9f in the main text and SI Figure S5).

S1.4. MD analysis reveals localized rigidification by the A6-A11 diselenide

Prior MD studies of insulin have identified both fast local flexibility and slower segmental conformational excursions, motivating the present simulations of glargine and Se-glargine (Mark et al., 1991; Busto-Moner et al., 2021). Our initial exploratory set comprised four independent 400-ns trajectories for each analog under comparable solvent and temperature conditions (Section 4.9), subsequently expanded to longer trajectories (1 μs each) and additional replicas (r_1–r_{10} per analog, cf. Figure S33) to improve χ_3 statistics. Ensemble representations from the extended replica set show that the two analogs preserve a similar overall fold, while local packing around A6-A11 appears slightly more ordered in Se-glargine (Figure 5e–h). This trend was already evident in the initial 400-ns trajectories and became clearer in the expanded ensembles.

Residue-resolved RMSF values, calculated from C_α coordinates, likewise indicate only modest differences across most of the insulin core, consistent with the close similarity of the NMR-derived solution structures and with the absence of a major global rearrangement (Figures 5 a-h and 9a). The central B-chain α-helix remains largely unaffected, whereas Se-glargine shows attenuated fluctuations in regions implicated in early partial unfolding, consistent with slightly tighter local packing near the A6-A11 bridge (Figure 1c; Nielsen et al., 2001; Ahmad et al., 2005).

A clearer site-specific effect is seen in the χ_3 torsion-angle (C_β–X–X–C_β; X = S or Se) distributions of the three intramolecular bridges (Figure 9b). Whereas the native A7–B7 and A20–B19 disulfides behave similarly in the two analogs, the A6-A11 Se–Se bond adopts a markedly narrower χ_3 distribution than the corresponding A6-A11 disulfide in glargine. Across both the initial and expanded replica sets, glargine samples two χ_3 states whereas Se-glargine remains largely confined to one (Figures 9b,c and S33). Time-resolved traces further show intermittent rotameric exchange in glargine but only small-amplitude fluctuations in Se-glargine, consistent with suppression of χ_3 exchange on the simulated timescale. One possible structural basis is steric crowding near Leu^{A16}, since a representative WT snapshot in the alternative χ_3 state brings the A6–A11 bridge into close contact with this side chain (Figure S33c).

These two WT conformational states are well precedented in the disulfide-bond literature (Armstrong et al., 2018; Fobe et al., 2019). After normalization by addition of +180°, the shared state s_2 exhibits a maximum at $\chi_3 \approx$ 279-290° and is sampled by both WT and Se-glargine, whereas the alternative state s_1 exhibits a maximum

near $\chi_3 \approx 247°$ and is observed only in WT. Together, these results suggest that, in this local structural context, the diselenide bridge acts as a more rigid "staple" on the present timescale without measurably perturbing the χ_3 behavior of the native A7-B7 and A20-B19 disulfides. This conclusion is specific to the present A6-A11 environment, however, and does not exclude the possibility that diselenide bridges at other, more solvent-exposed or conformationally open sites could access additional rotameric states.

Simple structural models further support a local packing explanation. Rigid-body selenium substitution at A6-A11 in a representative T-state crystallographic protomer (PDB entry 4INS), combined with MoloVol-based cavity analysis (Maglic and Lavendomme, 2022), suggests that the longer bond lengths and larger atomic radius of selenium reduce nearby microcavity volume and improve local packing near Ile^{A2}, Leu^{A16}, Leu^{B11}, and Tyr^{A19} (Figures S34 and S35). This interpretation is consistent with the richer A11-centered inter-residue NOE network observed experimentally for Se-glargine, including a more extensive set of A2-A8 α-helix-associated contacts. This same pattern is also evident in the extended replica-specific χ_3 distributions (Figure S33a,b). A plausible structural basis is shown by a representative WT snapshot from state s_1 (frame $f_{6,027}$; Figure S33c), in which the A6-A11 disulfide approaches Leu^{A16} closely. The corresponding diselenide would be less able to occupy this rotamer because the larger Se–Se bridge would increase steric crowding at this site.

Next, to understand whether local A6-A11 bridge dynamics are coupled to broader conformational fluctuations, Pearson correlation coefficients (ρ) (see Section S2.2 for further methods details) were calculated between the A6-A11 χ_3 dihedral and side-chain χ_2 dihedral angles throughout the protein. Analyses centered on the interval spanning the χ_3 transition observed in WT glargine showed moderate correlations both near and distal to the bridge (Figure 9d), whereas the corresponding interval in Se-glargine, which lacked a χ_3 transition, showed negligible correlations (Figure 9e). Similar trends were observed in additional views and across other dihedral classes (Figures S36-S38), replicas, and frame intervals. These results suggest that local A6-A11 bridge dynamics in WT glargine are coupled to more distributed conformational fluctuations in both chains and may contribute to early amyloidogenic fluctuations (Figure 1c).

These simulations nevertheless remain limited with respect to slower conformational excursions that may emerge only on longer timescales (including tens of microseconds or beyond). Across the initial 0.4-μs trajectories and the expanded 10×1-μs replica set, several χ_3 rotameric transitions were observed for the native

A6-A11 disulfide in WT glargine, whereas none were observed for the corresponding diselenide in Se-glargine (Figure S33), consistent with reduced χ_3 exchange on the present simulation timescale. In addition, the simulated distributions of interchain $C_a \cdots C_a$ distances (Figure S43) remain more restricted than those sampled in the non-native partial folds described by Busto-Moner et al., 2021 (Table S23). Future longer simulations, including trajectories initiated from such partial folds, will be needed to connect the present native-state dynamics more directly to early amyloidogenic intermediates (Figures 9f and S5).

S1.5. Fibril-state structural analysis and simulations

To assess whether the diselenide substitution is compatible with the fibril architecture, we performed a structural mutagenesis on the cryo-EM fibril structure using PyMOL (Figure S32) which resulted in *severe* steric clashes for three reported mutations (Thr^{A8}→Arg, Ile^{A10}→Arg, Asn^{A18}→Gln). These mutations are reported by cryo-EM to not form the same extended fibrils as wild type. We further computed the void space (Figure S31, panels *n*-*q*) using MoloVol for the WT- and diselenide fibril structures based on this cryo-EM structure. These showed a modest void space difference and the plausibility for a diselenide in the fibril structure. We further ran two short MD simulations (0.5 μs) of each of the two fibril structures. No β-sheet delamination was observed, and the fibril layers remained intact throughout the simulations (Figure S31, panels *r*-*u*). We therefore hypothesized that the prolonged lag time of Se-glargine (relative to glargine) reflects damping of conformational fluctuations in the susceptible monomer.

S2. Supplemental Methods

S2.1. EX2 Hydrogen Exchange Formalism

Under EX2 conditions the observed ^{1}H-^{2}H exchange rate constant (k_{obs}) is the product of a pH-dependent intrinsic rate constant (k_{int}) and the site-specific opening rate constant (k_{op}; Bai et al., 1993). The extent to which k_{obs} differs from k_{int} enables calculation of individual PFs (protection factors), defined as k_{int}/k_{obs} (Roder et al., 1985). Such PFs depend on local structural environment and so define a residue-specific probe. *Global* exchange refers to an overall equilibrium between native and unfolded structures; *subglobal* exchange pertains to cooperative segmental fluctuations. PFs may be interpreted in relation to protein stability (at sites of global exchange) or protein dynamics (at sites of subglobal or local exchange; Roder et al., 1985).

S2.2 Computational Details: Pearson R correlations

Correlation analysis was performed to quantify coupling between the A6–A11 χ_3 dihedral and residue-specific backbone (φ, ψ) and side-chain (χ_1, χ_2) dihedral angles. The χ_3 torsion was defined as C_β(A6)–**X**(A6)–**X**(A11)–Cβ(A11), where **X** = S (WT) or Se (Se-glargine). Dihedral time series for all residues in the PROA and PROB chains were extracted from MD trajectories using MDAnalysis. Backbone (φ, ψ) and side-chain (χ_1, χ_2) dihedral angles were computed using standard torsional definitions, and angular data were mapped to a 0-360° range prior to the analysis.

Correlations were computed using a formulation appropriate for periodic *angular* variables (eq. 1 below), thereby avoiding artifacts associated with linear correlation of dihedral angles (Jammalamadaka and SenGupta, 2001). For global analysis (Figure S37), correlations were calculated over *all* frames of the trajectory (i.e., 20,000 frames) without subsampling. To probe transient coupling associated with χ_3 state transitions, correlations were also evaluated over restricted time windows defined by frame indices (e.g., frames 2,500-5,499; Figure S38), as well as additional fixed and overlapping windows (e.g., 0-3,000, 2,000-5,000, 5,000-20,000; Figure S38). Correlation magnitudes were interpreted qualitatively as weak ($|\rho| < 0.25$), moderate ($|\rho| \geq 0.25$), or strong ($|\rho| \geq 0.5$).

$$\rho = \frac{\sum_i \sin(\theta_i - \bar{\theta}) \sin(\phi_i - \bar{\phi})}{\sqrt{\sum_i \sin^2(\theta_i - \bar{\theta}) \sum_i \sin^2(\phi_i - \bar{\phi})}} \quad \text{(eq 1)}$$

S2.3 Computational Details: Se–Se Diselenide Implementation in CHARMM

A Se–Se diselenide bond was implemented in CHARMM36 by extending the standard CYS/DISU topology. A selenocysteine residue (SEC) was defined by replacing sulfur (SG) with selenium (SE; atom type SX1), and a corresponding diselenide patch (DSEC) was introduced to form Se–Se linkages (SX2), directly analogous to DISU. Please refer to the schematic workflow of topology construction and parameter assignment in Figure S42, which illustrates how to implement the Se–Se diselenide bridge in CHARMM, including the list of additional parameter- and scaling details used. Bonded parameters were mapped from the corresponding CHARMM36 disulfide terms due to indistinguishable topology. Non-bonded parameters for selenium were assigned using reference selenol data (e.g., CH_3SeH) Pedron et al., 2023) with an increased Lennard–Jones radius applied to the bridge selenium (SX2) to reflect its larger and more polarizable environment. Lennard–Jones parameters were obtained via scaling relative to cysteine sulfur, yielding $R_{min}/2 \approx 2.42$ Å for the diselenide bridge. Partial charges were minimally adjusted to maintain overall neutrality. All parameters and topology identities are summarized in Tables S17-S22.

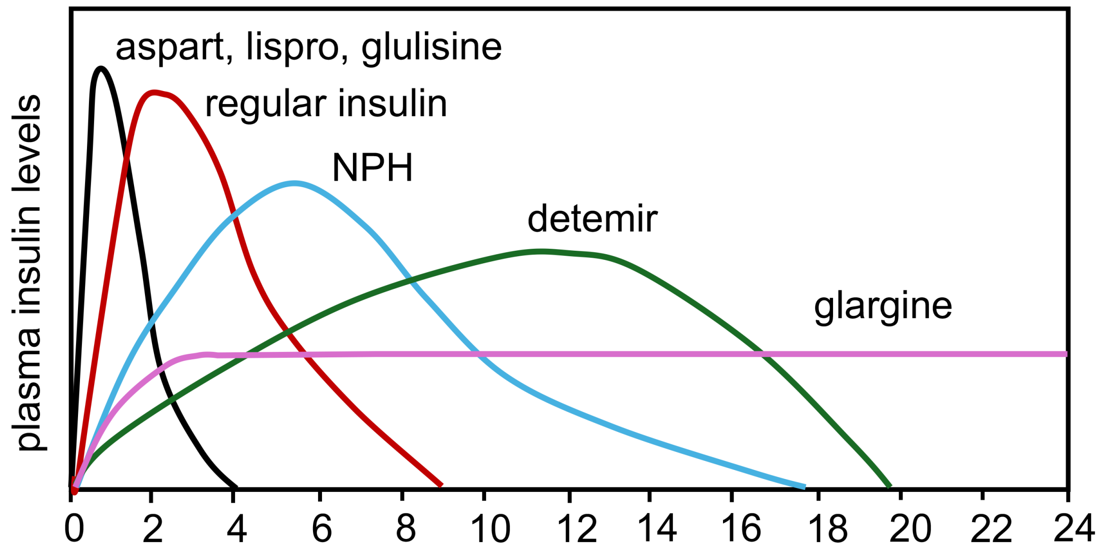

Figure S3. Pharmacokinetic profile of insulin injections: onset, peak activity, and duration of action (adapted from reference Petznick, 2011).

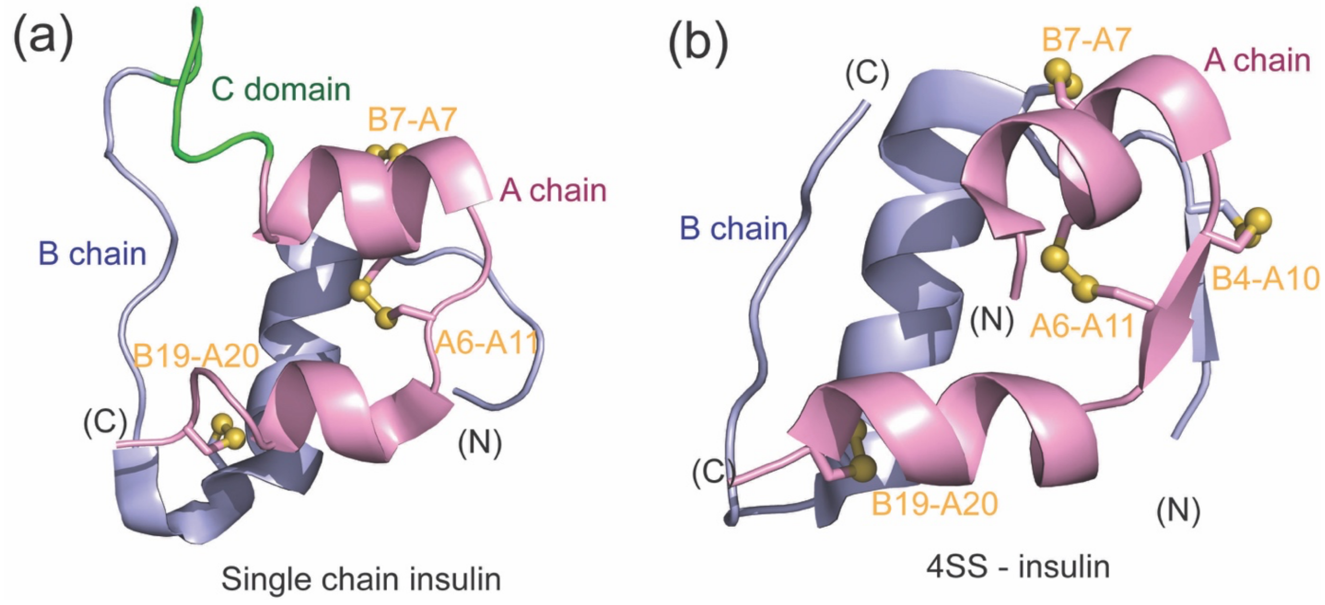

Figure S4. Prior strategies for improving insulin stability involved linking the B- and A chains with short peptide connectors and introducing an additional disulfide bond. (a) NMR structure of single chain insulin (PDB-ID: 2LWZ). (b) X-ray crystal structure of four-disulfide insulin (PDB-ID: 4EFX). Color coding: A chain (pink), B chain (blue), C domain (green), disulfides are shown as gold spheres with one third van der Waals radii.

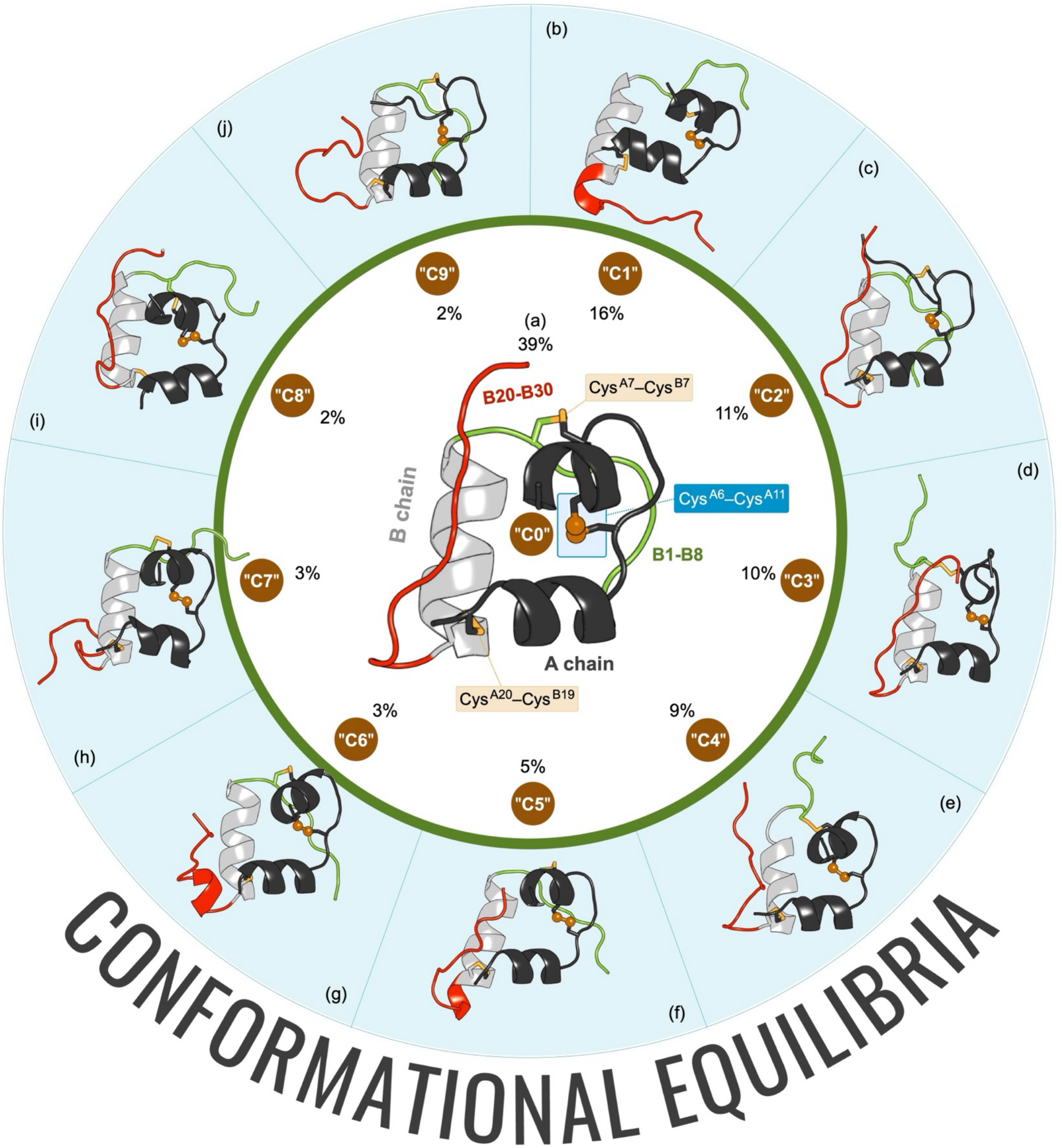

Figure S5. Conformational "islands" **C0–C9** of monomeric human insulin as defined by Dinner and co-workers (Busto-Moner et al., 2021). The central structure shows the A chain (dark grey) and B chain (light grey) with the B-chain N-terminal segment B1–B8 (green), C-terminal segment B20–B30 (red), and the A-chain disulfide CysA6–CysA11 (burnt orange). Surrounding panels depict representative structures from each island (**C0–C9**) projected around the circle, colored with the same scheme. Labels and percentages indicate the corresponding island identity and fractional population in the conformational ensemble.

(a)

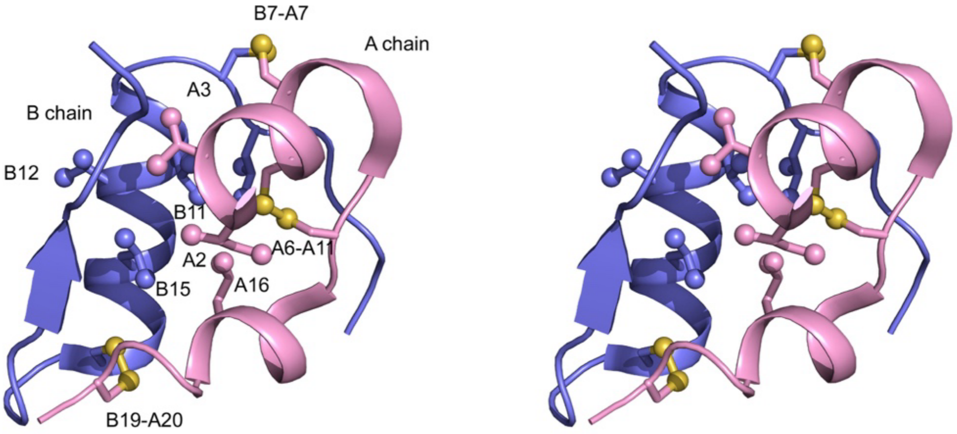

(b)

	B chain	A chain
mammals		
human	FVNQHLCGSHLVEALYLVCGERGFFYTPKT	GIVEQCCTSICSLYQLENYCN
dog	FVNQHLCGSHLVEALYLVCGERGFFYTPKA	GIVEQCCTSICSLYQLENYCN
cat	FVNQHLCGSHLVEALYLVCGERGFFYTPKA	GIVEQCCASVCSLYQLEHYCN
pig	FVNQHLCGSHLVEALYLVCGERGFFYTPKA	GIVEQCCTSICSLYQLENYCN
cattle	FVNQHLCGSHLVEALYLVCGERGFFYTPKA	GIVEQCCASVCSLYQLENYCN
sheep	FVNQHLCGSHLVEALYLVCGERGFFYTPKA	GIVEQCCAGVCSLYQLENYCN
beluga Whale	FVNQHLCGSHLVEALYLVCGERGFFYTPKA	GIVEQCCTSICSLYQLENYCN
rabbit	FVNQHLCGSHLVEALYLVCGERGFFYTPKS	GIVEQCCTSICSLYQLENYCN
gorilla	FVNQHLCGSHLVEALYLVCGERGFFYTPKT	GIVEQCCTSICSLYQLENYCN
chimpanzee	FVNQHLCGSHLVEALYLVCGERGFFYTPKT	GIVEQCCTSICSLYQLENYCN
bornean orangutan	FVNQHLCGSHLVEALYLVCGERGFFYTPKT	GIVEQCCTSICSLYQLENYCN
green monkey	FVNQHLCGSHLVEALYLVCGERGFFYTPKT	GIVEQCCTSICSLYQLENYCN
rodents		
rat	FVKQHLCGPHLVEALYLVCGERGFFYTPKS	GIVDQCCTSICSLYQLENYCN
octodon Degu	YSSQHLCGSNLVEALYMTCGRSGFYRPHD	GIVDQCCNNICTFNQLQNYCNVP
guinea pig	FVSRHLCGSNLVETLYSVCQDDGFFYIPKD	GIVDQCCTGTCTRHQLQSYCN
porcupine	FVNQHLCGSHLVEALYLVCGNDGFFYRPKA	GIVDQCCTGVCSLYQLQNYCN
chinese hamster	FVNQHLCGSHLVEALYLVCGERGFFYTPKS	GIVDQCCTSICSLYQLENYCN
reptiles and amphibians		
chameleon	LPNQHLCGSHLVEALYLVCGDRGFYYSPKT	GIVQQCCENTCSLYELENYCN
african clawed frog	LVNQHLCGSHLVEALYLVCGDRGFFYYPKV	GIVEQCCHSTCSLFQLESYCN
mainland tiger snake	APNQRLCGSHLVEALFLICGERGFYYSPRE	GIVEQCCENTCSLYELENYCN
central bearded dragon	IPNQHLCGSHLVEALYLVCGERGFYYSPKT	GIVQQCCENTCSLYELENYCN
alligator	AANQRLCGSHLVDALYLVCGERGFFYSPKG	GIVEQCCHNTCSLYQLENYCN
birds		
chicken	AANQHLCGSHLVEALYLVCGERGFFYSPKA	GIVEQCCHNTCSLYQLENYCN
turkey vulture	VANQHLCGSHLVEALYLVCGERGFFYSPKA	GIVEQCCHNTCSLYQLENYCN
common ostrich	AANQHLCGSHLVEALYLVCGERGFFYSPKA	GIVEQCCHNTCSLYQLENYCN
Rufous hummingbird	AVNQHLCGSHLVEALYLVCGERGFFYSPKA	GIVEQCCHNTCSLYQLENYCN
Wild turkey	AANQHLCGSHLVEALYLVCGERGFFYSPKA	GIVEQCCHNTCSLYQLENYCN
brown roatelo	AANQHLCGSHLVEALYLVCGERGFFYSPKA	GIVEQCCHNTCSLYQLENYCN
fish		
zebrafish	GTPQHLCGSHLVDALYLVCGPTGFFYNPKR	GIVEQCCHKPCSIFELQNYCN
common carp	GAPQHLCGSHLVDALYLVCGPTGFFYNP	GIVEQCCHKPCSIFELQNYCN
anglerfish	APAQHLCGSHLVDALYLVCGDRGFFYNP	GIVEQCCHRPCNIFDLQNYCN
barfin flounder	LPPQHLCGAHLVDALYLVCGERGFFYTP	GIVEQCCHKPCNIFDLQNYCN
Nile tilapia	GGPQHLCGSHLVDALYLVCGDRGFFYNPR	GIVEECCHKPCTIFDLQNYCN
butterflyfish	ASSQHLCGSHLVDALYMVCGEKGFFYQPKT	GIVEQCCHHPCNIFDLQNYCN
Chum salmon	AAAQHLCGSHLVDALYLVCGEKGFFYTP	GIVEQCCHKPCNIFDLQNYCN
ghost shark	VPTQRLCGSHLVDALYFVCGERGFFYSPKQ	GIVEQCCHNTCSLVNLEGYCN
spotted ratfish	VPTQRLCGSHLVDALYFVCGERGFFYSPKP	GIVEQCCHNTCSLANLEGYCN
lamprey	SALTGAGGTHLCGSHLVEALYVVCGDRGFFYTPSKT	GIVEQCCHRKCSIYDMENYCN
hagfish	RTTGHLCGKDLVNALYIACGVRGFFYDPTKM	GIVEQCCHKRCSIYDLENYCN

Figure S6. High conservation within the core. (a) Stereo view of insulin monomer with conserved residues highlighted. Color coding: A chain (pink), B chain (blue), methyl groups and disulfides (gold) are shown as spheres with one third van der Waals radii. (b) Sequences of insulins from different species with bold residues being core residues that are >90% conserved.

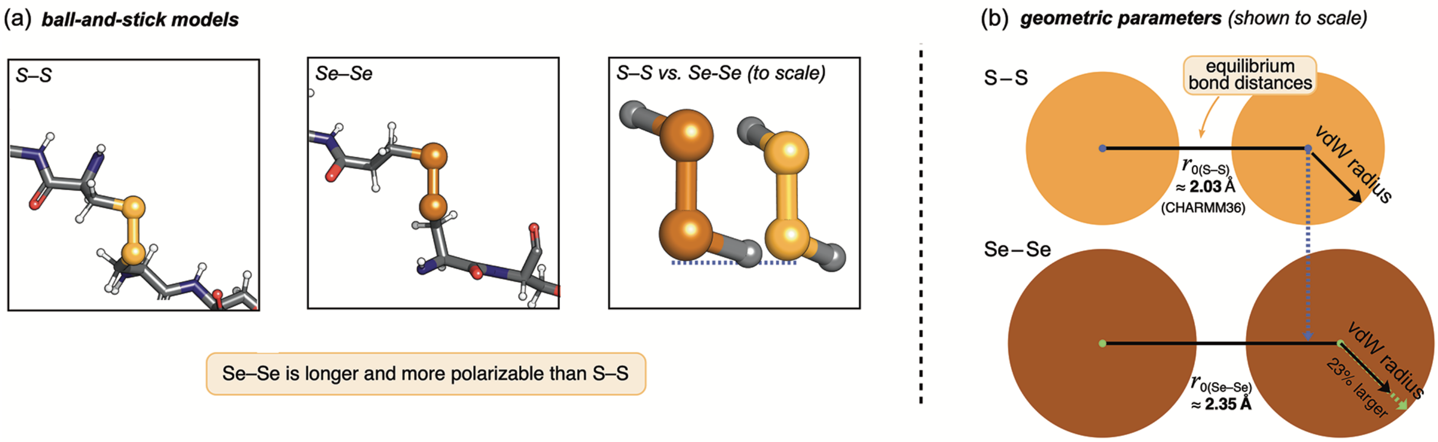

Figure S7. Structural comparisons of S–S (WT) and Se–Se A6–A11 bridges in glargine. (a) Ball-and-stick models of the A6–A11 bridge showing the disulfide (S–S) and diselenide (Se–Se); the rightmost inset places both bridges on the same scale to emphasize that Se–Se is longer (and more polarizable) than S–S. (b) Geometric parameters used in the corresponding simulations are shown to scale. For more details on the underlying values used, refer to a Se–insulin analog (PDB-ID: 6H3M; Weil-Ktorza et al., 2019) with eight crystallographically independent protomers containing SecA6–SecA11 diselenide bridges. The observed Sec^{A6}–Sec^{A11} distances clustered directly below ~2.4 Å and we therefore adopted an equilibrium Se–Se bond length of 2.35 Å for the A6–A11 diselenide in our CHARMM parametrization, which matches a wide search in Conquest by Cambridge Structural Database of Se–Se bonds. The equilibrium distance in the two species is $r_{0(S–S)}$ = 2.03 Å, and $r_{0(Se–Se)}$ = 2.35 Å, respectively. Circle sizes represent the relative van der Waals radii of S and Se, and colored dots indicate the atom centers from which the bond distances are measured.

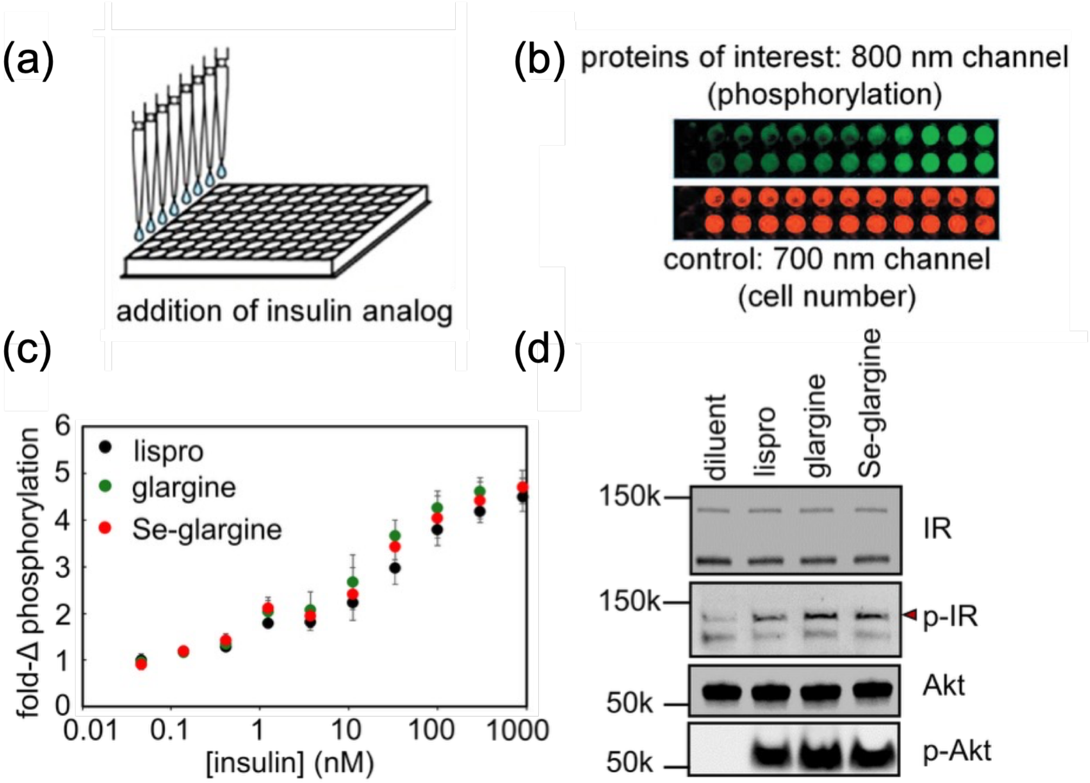

Figure S8. Biological activity of selenium-substituted glargine (Se-glargine). (a, b) Schematic of the in-cell Western blot assay used to assess the activity of insulin analogs. Human HepG2 cells were plated in parallelized 96-well formats and treated with increasing concentrations of individual insulin analogs (a), enabling fluorescent detection of hormone-dependent insulin receptor (IR) autophosphorylation (b). Hormone-induced IR autophosphorylation was quantified by optical readouts (upper panels; pseudo-green signals). Signal intensity was normalized to DRAQ5 fluorescence at 700 nm (lower panels; pseudo-red signals) to control for cell number. (c) Dose–response curves showing fold changes in IR autophosphorylation (pIR/IR; *y*-axis) as a function of insulin analog concentration (50 pM–1 μM; *x*-axis, log scale). Data represent mean ± SEM from three independent biological replicates. Data in this panel is reproduced from prior publication for convenience of the reader: (Weil-Ktorza et al., 2024) (d) Representative Western blots detecting insulin receptor phosphorylation and downstream Akt phosphorylation following stimulation with the indicated insulin analogs. Phosphorylated IR bands are marked by red arrows, and molecular weight markers are shown to the left of each gel.

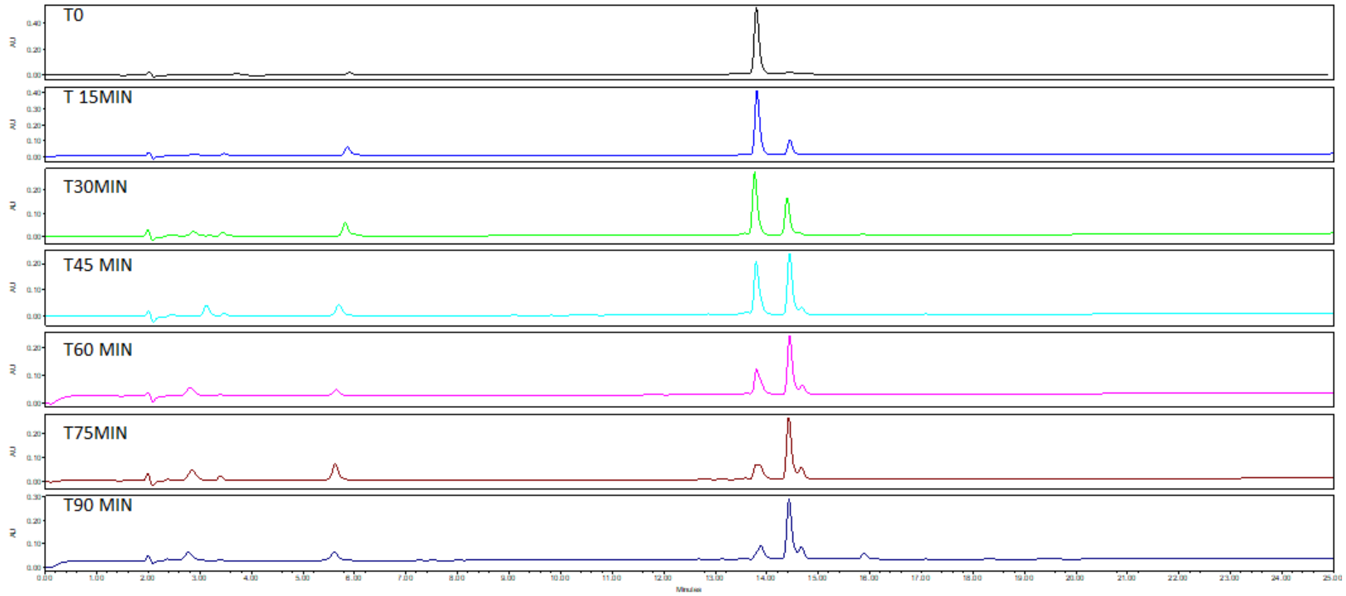

Figure S9. HPLC monitoring of pepsin cleavage reaction using native glargine i.e., with Cys^{A6} and Cys^{A11}; henceforth designated simply "glargine". The peak corresponding to the starting material at a retention time of 13.8 minutes gradually diminishes following pepsin treatment.

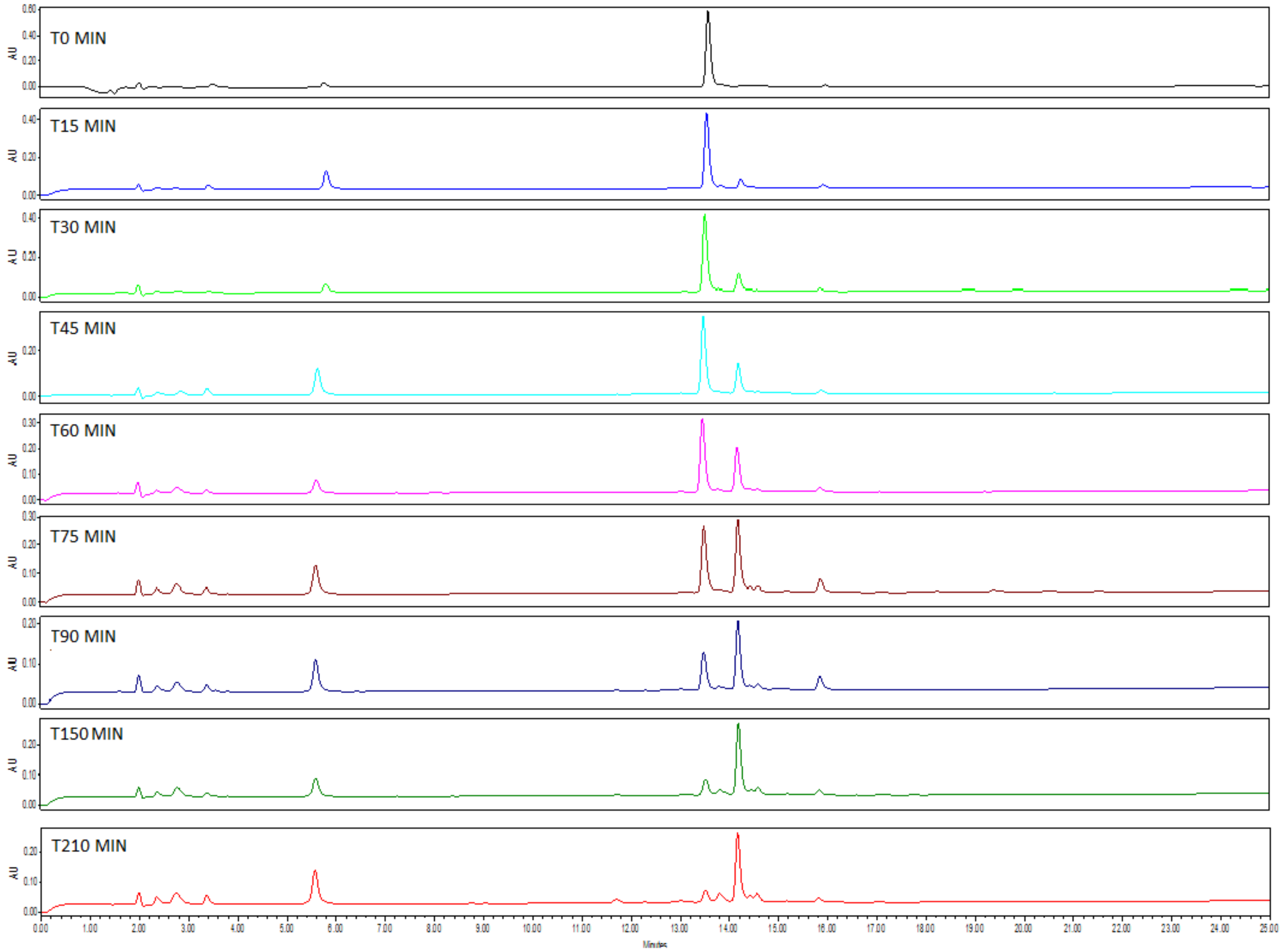

Figure S10. HPLC monitoring of pepsin cleavage reaction using Se-glargine. The peak corresponding to the starting material at a retention time of 13.4 minutes gradually diminishes following pepsin treatment.

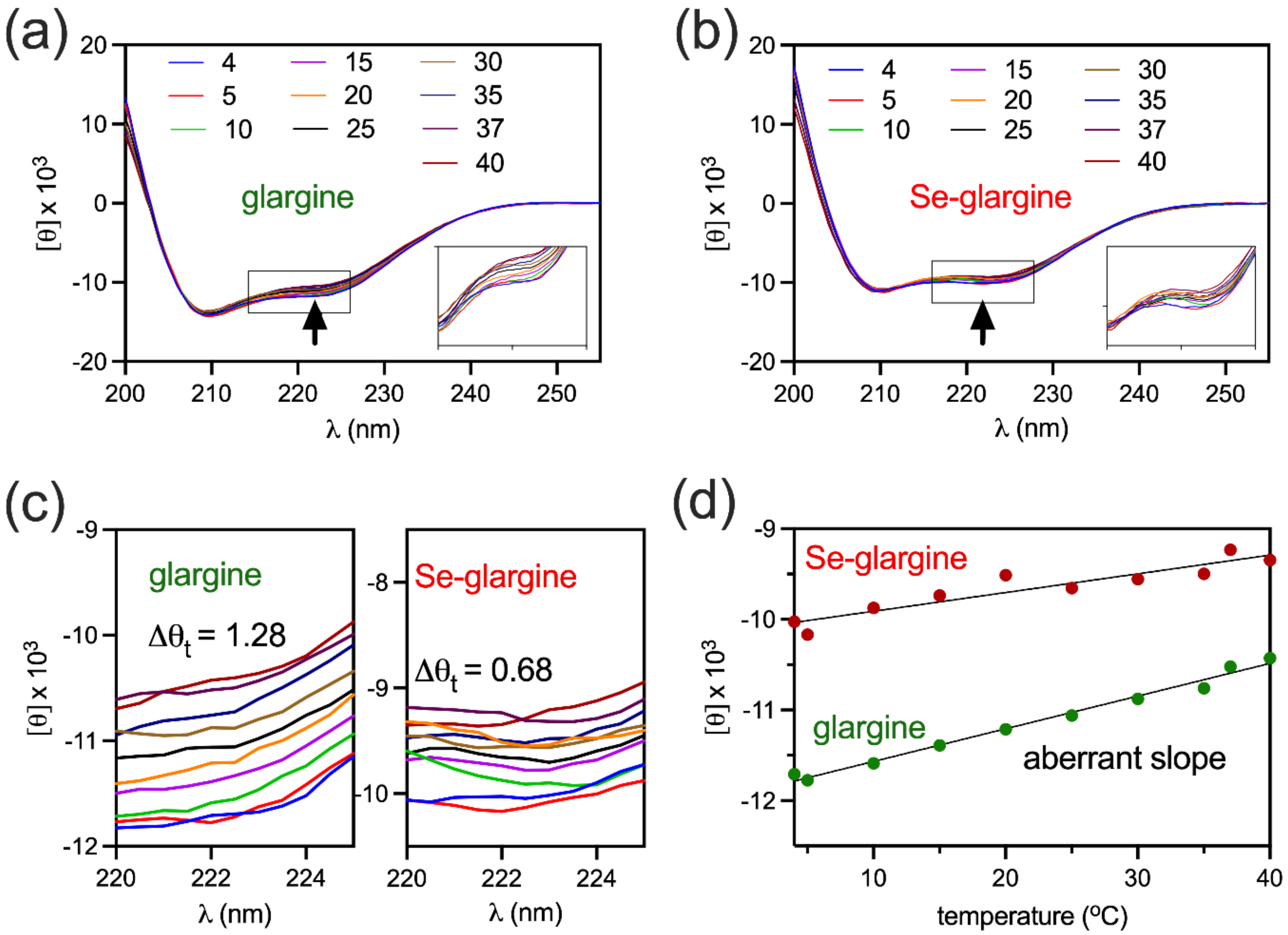

Figure S11. Analysis of stability differences between glargine and Se-glargine. (a) Wavelength scans of glargine from 4–40 °C. (b) Wavelength scans of Se-glargine over the same range. Insets highlight the 222 nm band region. (c) Expanded regions from 220-225 nm are provided. (d) Plot of 222 nm band vs temperature reveals aberrant slope for glargine when compared to Se-glargine.

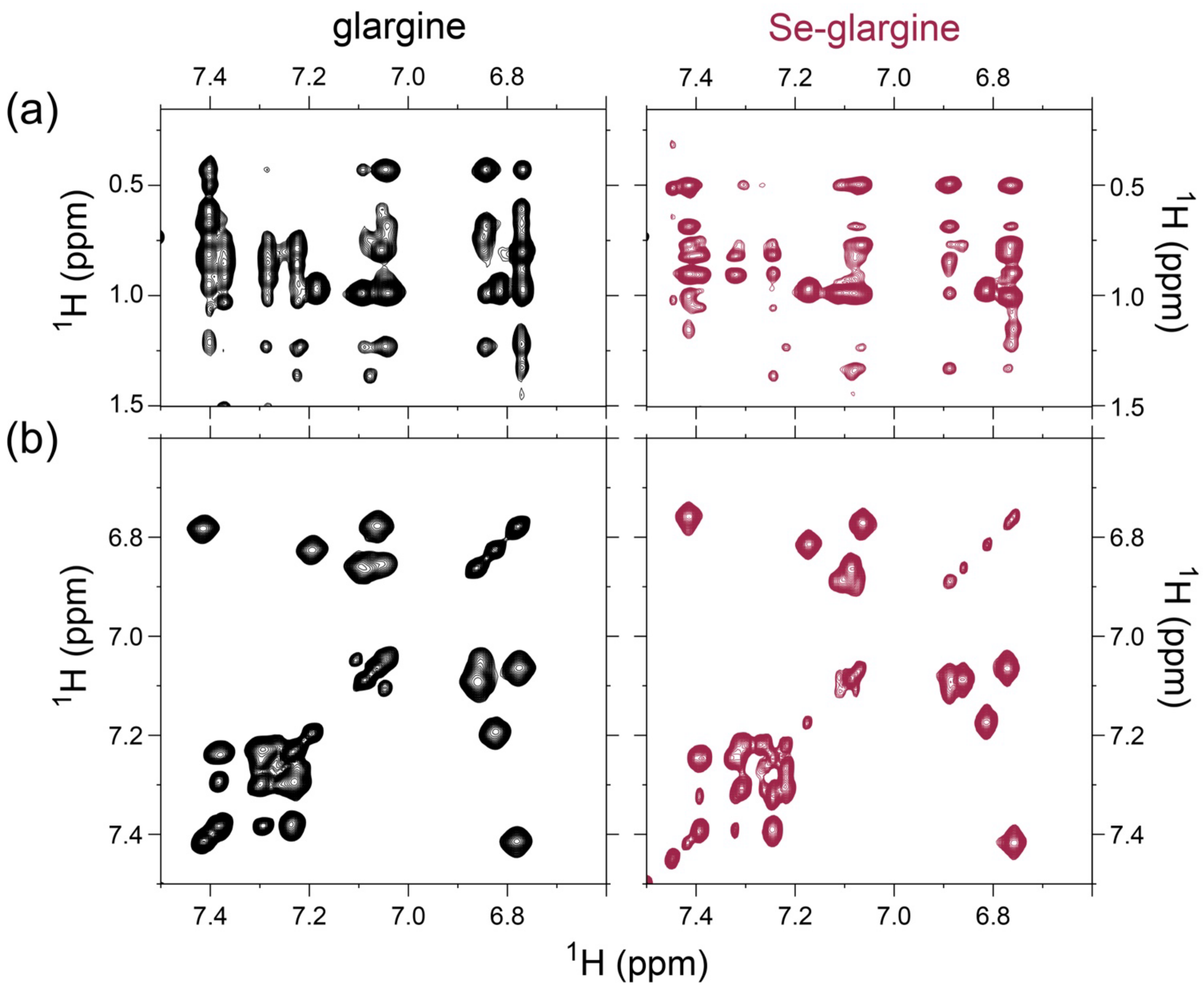

Figure S12. Homonuclear 2D-NMR spectra of glargine (*left panel, black*) and Se-glargine (*right panel, maroon*): (*a*) NOESY spectra (mixing time 150 ms) showing NOEs from aromatic protons to methyl protons and (*b*) TOCSY spectra (mixing time 55 ms) showing aromatic resonance correlation. Spectra were acquired at a ^{1}H frequency of 700 MHz in 10% deuterated acetic acid (pH 2.1, direct meter reading) at 25 °C.

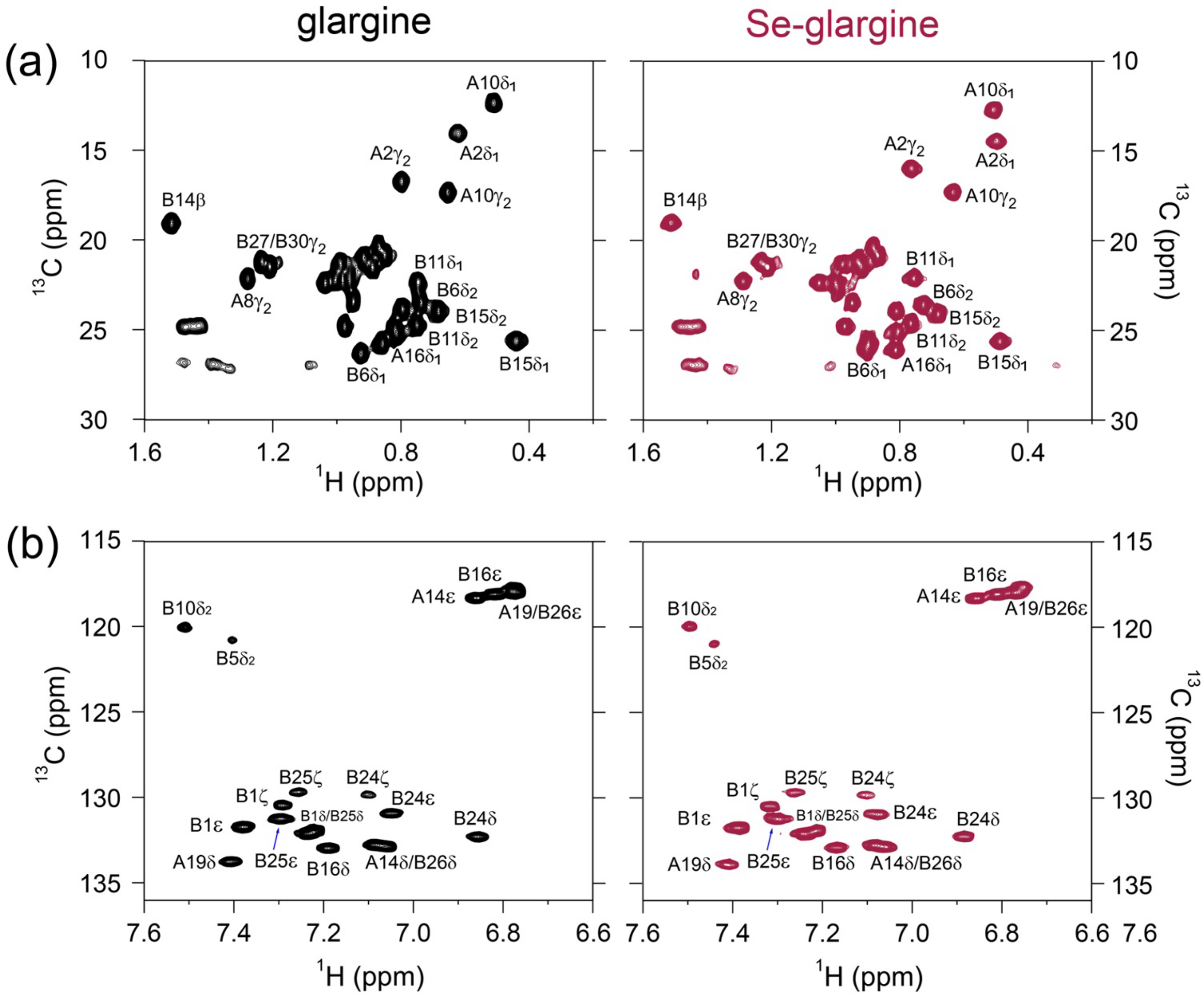

Figure S13. Natural abundance ^{1}H-^{13}C HSQC spectra of glargine insulin (left panel, black) and A6-A11 Se-glargine (right panel, maroon) at (a) methyl and (b) aromatic regions. Spectra were acquired at a ^{1}H frequency of 700 MHz in 10% deuterated acetic acid (pH 2.1, direct meter reading) at 25 °C.

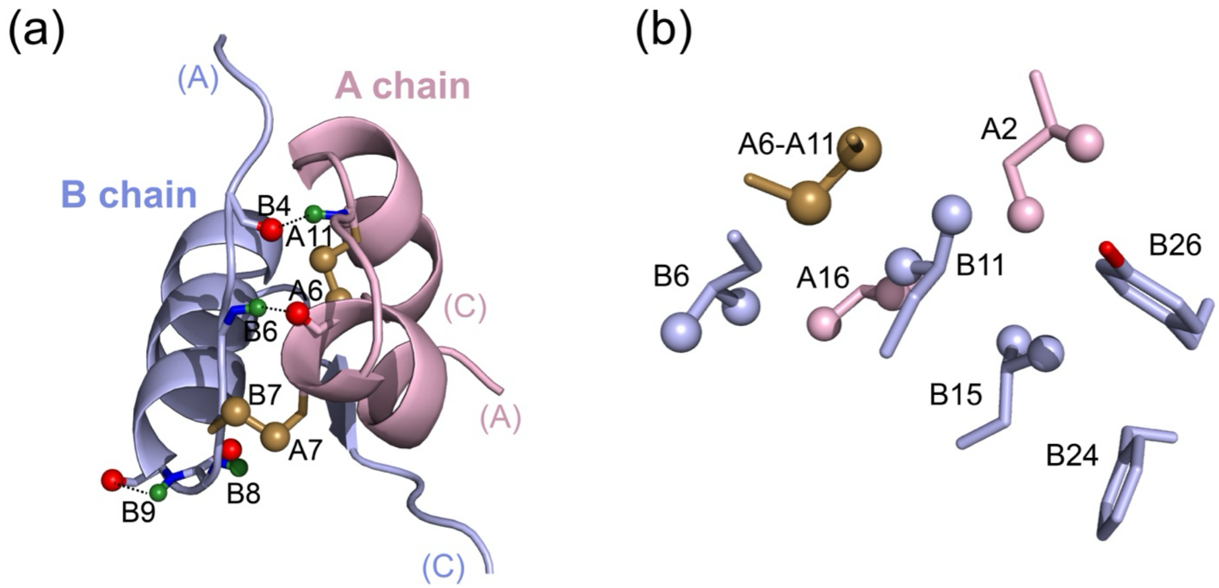

Figure S14. (a) Structure representation of amide protons affected by A6-A11 substitution (PDB-ID: 4INS). The A chain is in *light pink,* B chain in *light blue* and disulfide bridges in *gold.* The amide protons of Cys^{A11}, Leu^{B6}, Gly^{B8} and Ser^{B9} are shown in *green spheres*, the carbonyl oxygen atoms of Gln^{B4}, Ser^{B9}, Cys^{A6} and Ser^{B9} side-chain oxygen in *red spheres*. Dashed lines indicate Leu^{B6}-H_N···O=C-Cys^{A6}, Cys^{A11}-H_N···O=C-Gln^{B4} and Ser^{B9}-H_N···OH-Ser^{B9} hydrogen bonds. (b) Structure representation of the transmission of chemical-shift perturbations from A6-A11 disulfide bridge to distant sites through hydrophobic interactions.

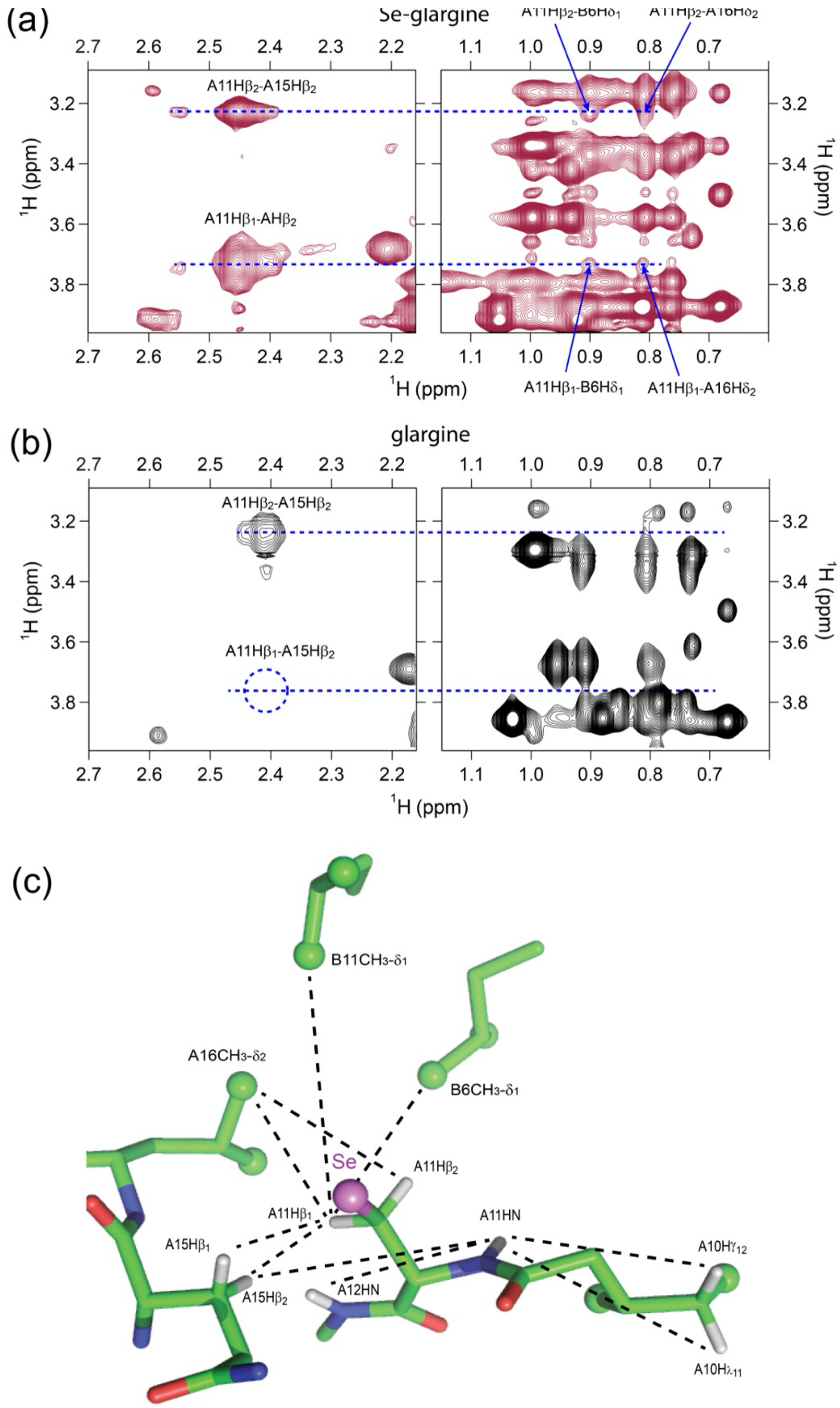

Figure S15. Homonuclear 2D NOESY spectra (mixing time 150 ms) of glargine (*black*) and Se-glargine (*maroon*): (a) NOESY spectra showing A11 H_{β}-related NOEs of Se-glargine. (b) NOESY spectra showing A11 H_{β}-related NOEs of glargine. Only A11 $H_{\beta 2}$ NOE cross peaks were clearly visualized in the spectrum of glargine. The A11 $H_{\beta 1}$-related NOE cross peaks were not visualized due to exchange line broadening. Dashed circle indicated assuming NOE cross peak. (c) Structure representation of A11-related NOEs that were only observed in the Se-glargine.

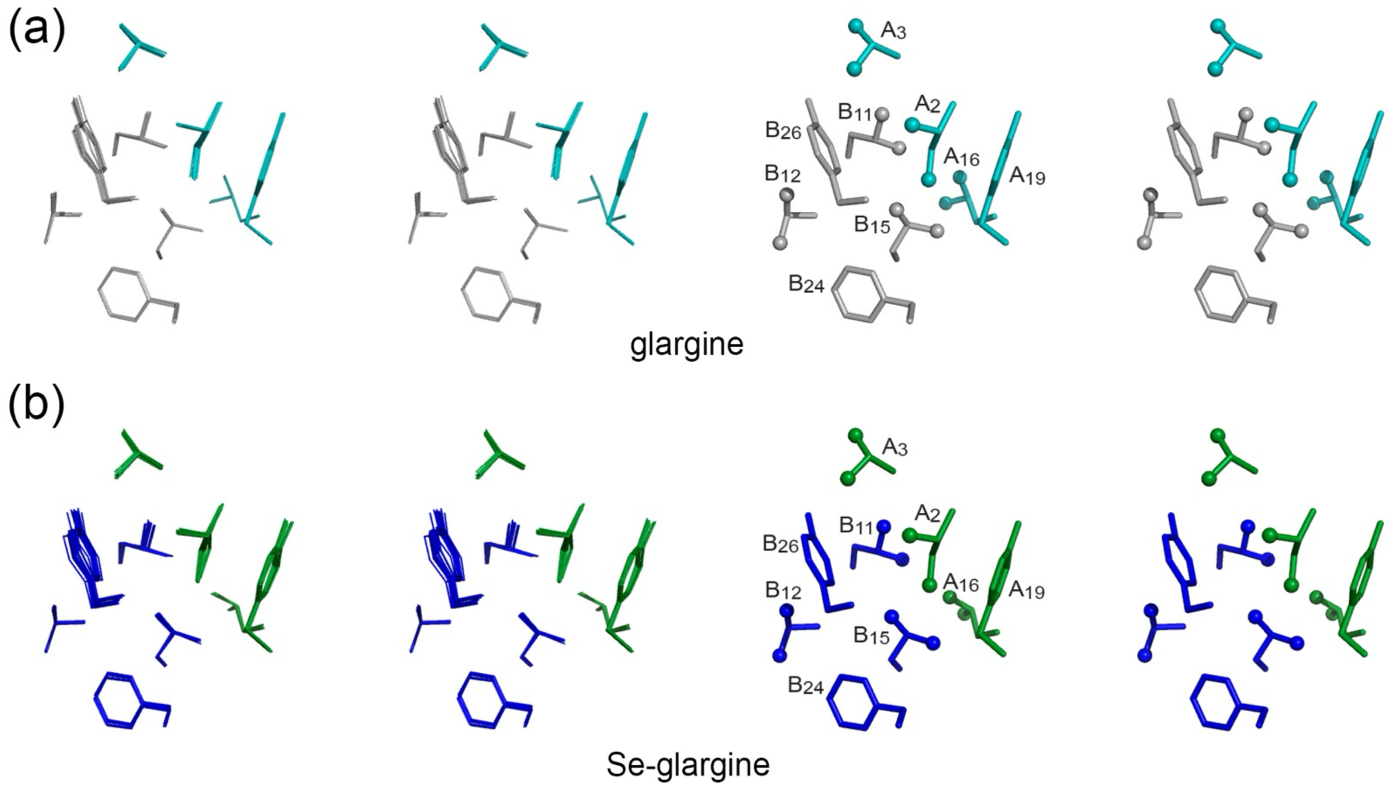

Figure S16. Expanded and stereo view of the major hydrophobic environment in the ensemble (*left*) and in a representative stick model (*right*). The structure was aligned for residues 1-21 (A1-A21) and 24-49 (B3-B28). (a) Glargine insulin; the A chain is *cyan* and B chain *gray.* Methyl groups represented as *cyan or gray spheres*. (b) A6-A11 diselenide glargine insulin; the A chain is *green* and B chain *blue.* Methyl groups represented as *green or blue spheres*.

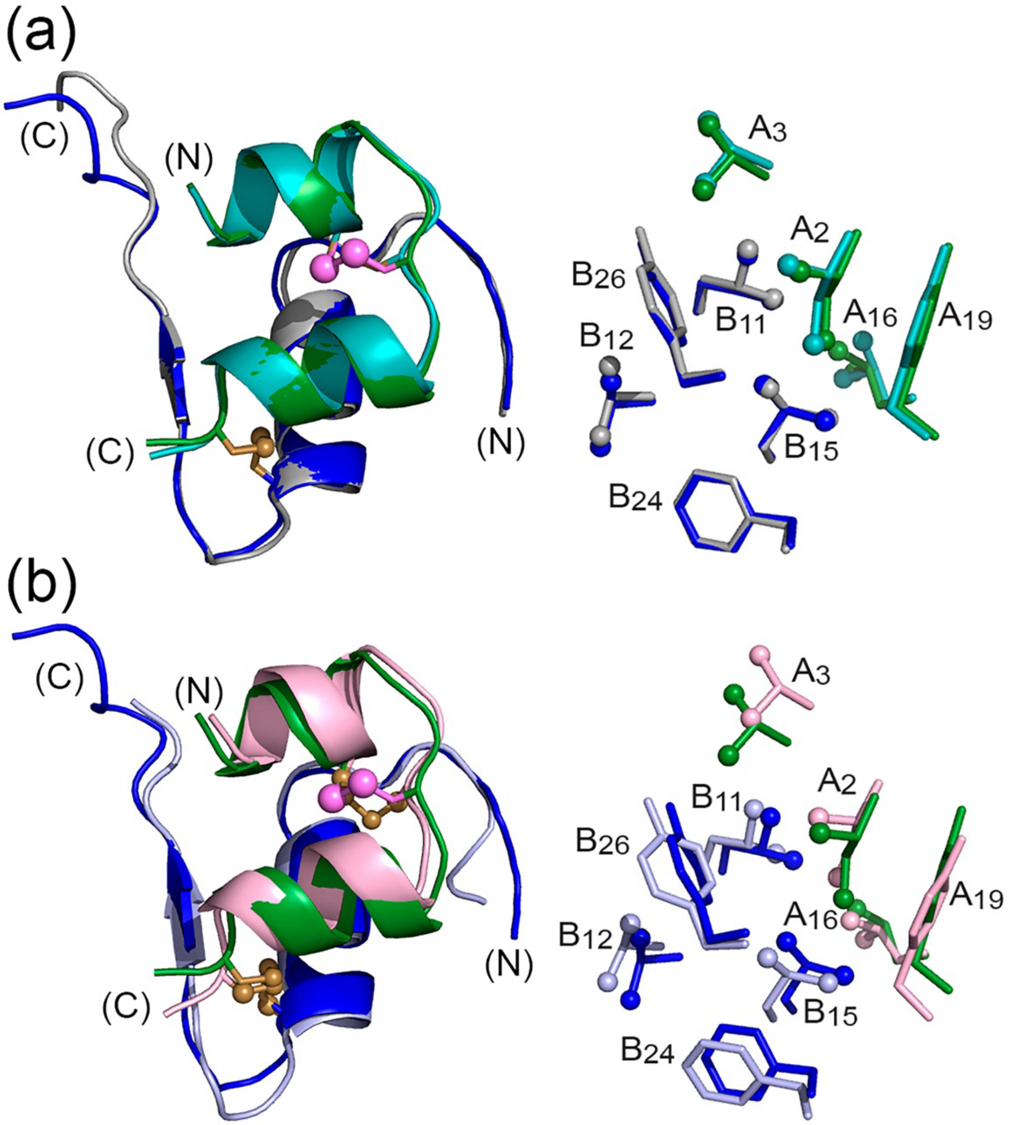

Figure S17. Structure comparison of Se-glargine and glargine insulin. (a) Overlay of NMR structure of Se-glargine and glargine; ribbon structure (left panel) and major hydrophobic core (right panel). The A chain is in *green* and B chain in *blue* in Se-glargine*;* and the A chain is in *cyan* and B chain in *gray* in glargine; disulfide bridges are *gold and* diselenide bond *violet*. (b) Overlay of NMR structure of Se-glargine and X-ray structure of glargine insulin (PDB-ID: 4IYD); ribbon structure (left panel) and major hydrophobic core (right panel). The color code of Se-glargine is same as in panel *a*; the A chain is in *light pink* and B chain in *light blue* in the X-ray model of glargine. Methyl groups represented as corresponding *spheres*.

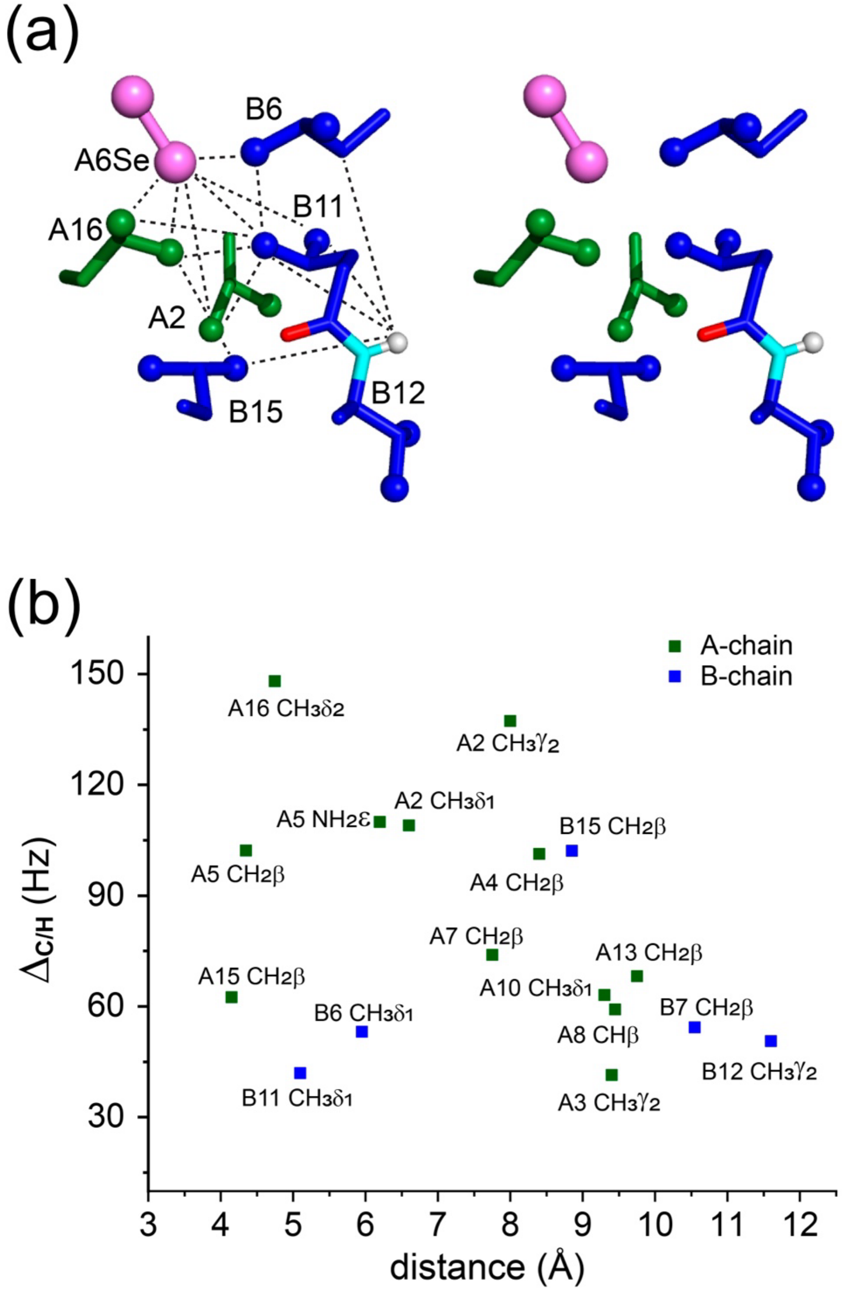

Figure S18. (a) Stereo view of structure representation of the communication path from A6-A11 diselenide bridge to the amide proton of Val^{B12} in Se-glargine. Methyl groups represented as *green* (A chain) or *blue spheres* (B chain), Val^{B12} amide proton in *white sphere.* (b) Side-chain chemical shift perturbations related to the atomic distance from A6-A11 diselenide bridge. The distance is the average from A6/A11-Se to side-chain carbon (or nitrogen) atom. For -CH_2- side-chain groups, bigger chemical shift perturbation was selected. A-chain residues are displayed in *green* and B-chain residues in *blue*.

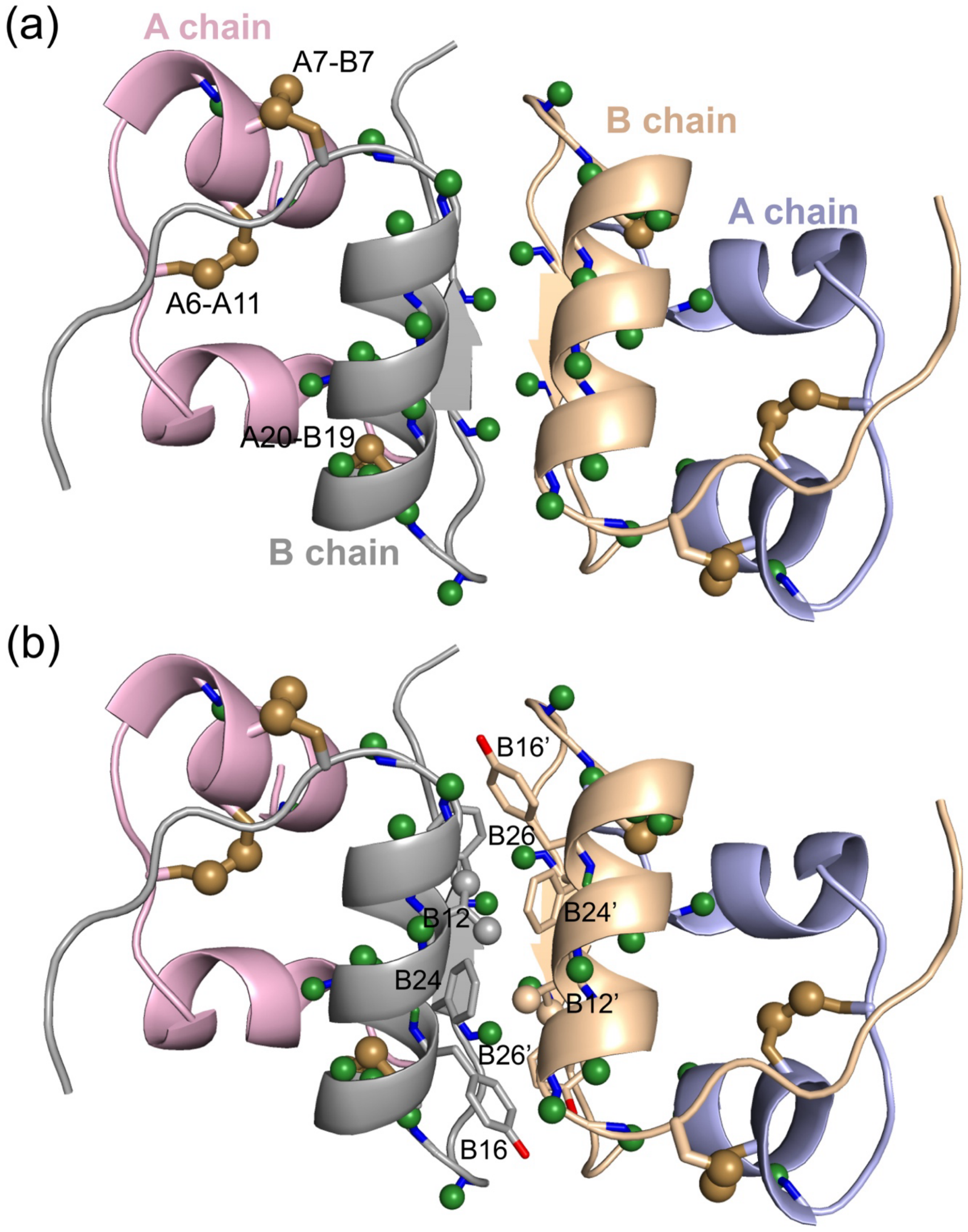

Figure S19. (a) Ribbon structural representation of insulin dimer (PDB-ID: 4IYD) and that with important interfacial residues (b). Slow exchange amide protons between monomer and dimer were shown in *green* spheres that displayed exchange cross peak in the TOCSY and NOESY spectra. The A chain is in *light pink* and B chain in *gray* in one protomer*;* and the A chain is in *wheat* and B chain in *light purple* in another protomer; disulfide bridges are in *gold*. Methyl groups in the dimer interface represented as corresponding *spheres*.

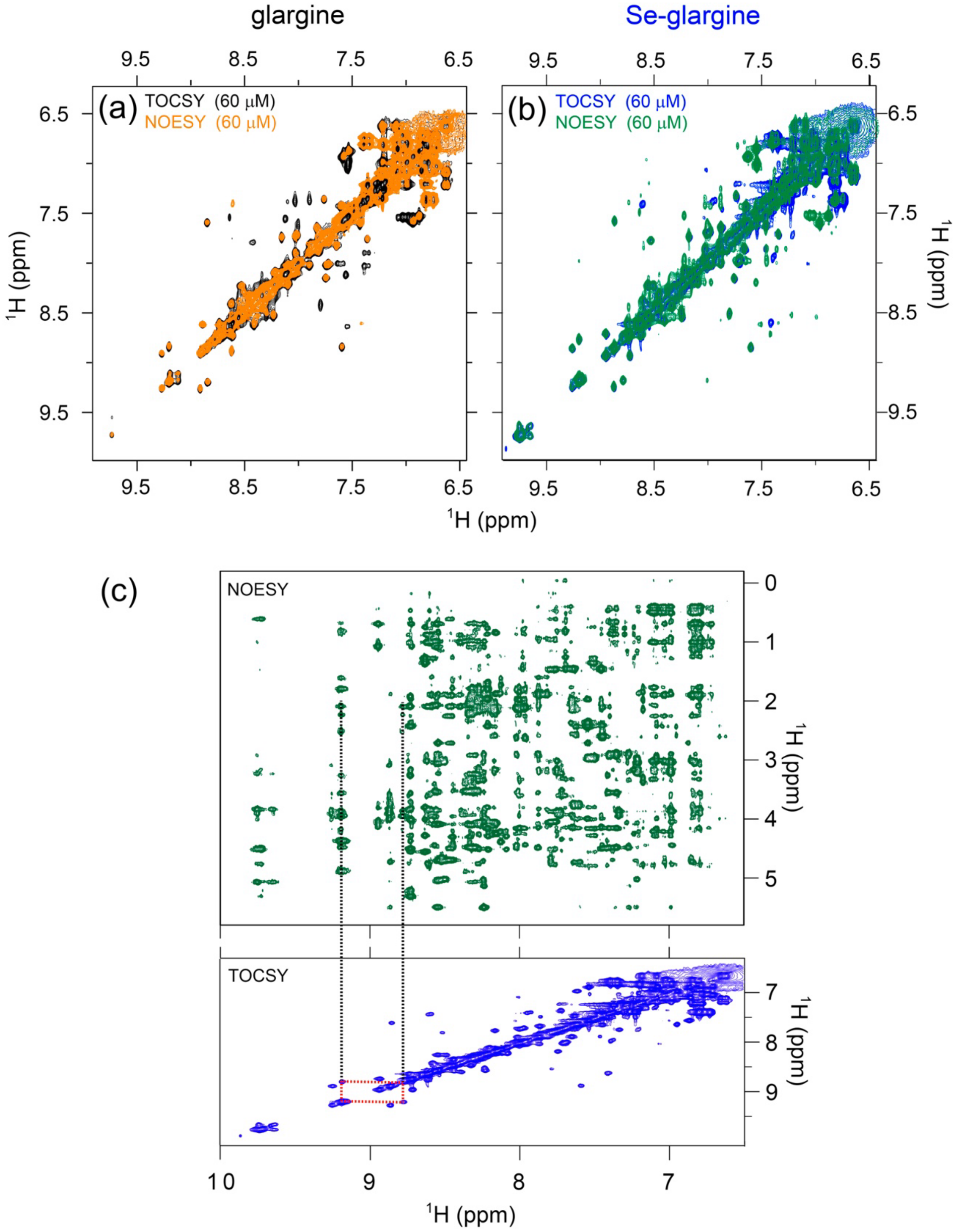

Figure S20. NMR evidence of monomer-dimer equilibrium of glargine and Se-glargine in low protein concentration (~60 μM). (a) Spectral overlay of TOCSY (*back*) and NOESY (*orange*) spectra of glargine. (b) Spectral overlay of TOCSY (*blue*) and NOESY (*green*) spectra of Se-glargine. Spectra were acquired at a ^{1}H frequency of 700 MHz in 10mM deuterated acetic acid (pH 3.0, direct meter reading) at 25 °C. (c) 2D NOESY (*top, green*) and TOCSY spectra (*bottom, blue*) of A6-A11 Se-glargine. Dashed rectangular box in *red* indicates exchange pattern between monomer and dimer amide protons (residue Glu^{B21}) observed in TOCSY spectrum. Similar NOE patterns from the monomer and the dimer indicated by dashed lines in *black* provided further evidence of monomer-dimer equilibrium of Se-glargine in the protein concentration of 60 μM in 10-mM deuterated acetic acid at pH 3 and at 25 °C.

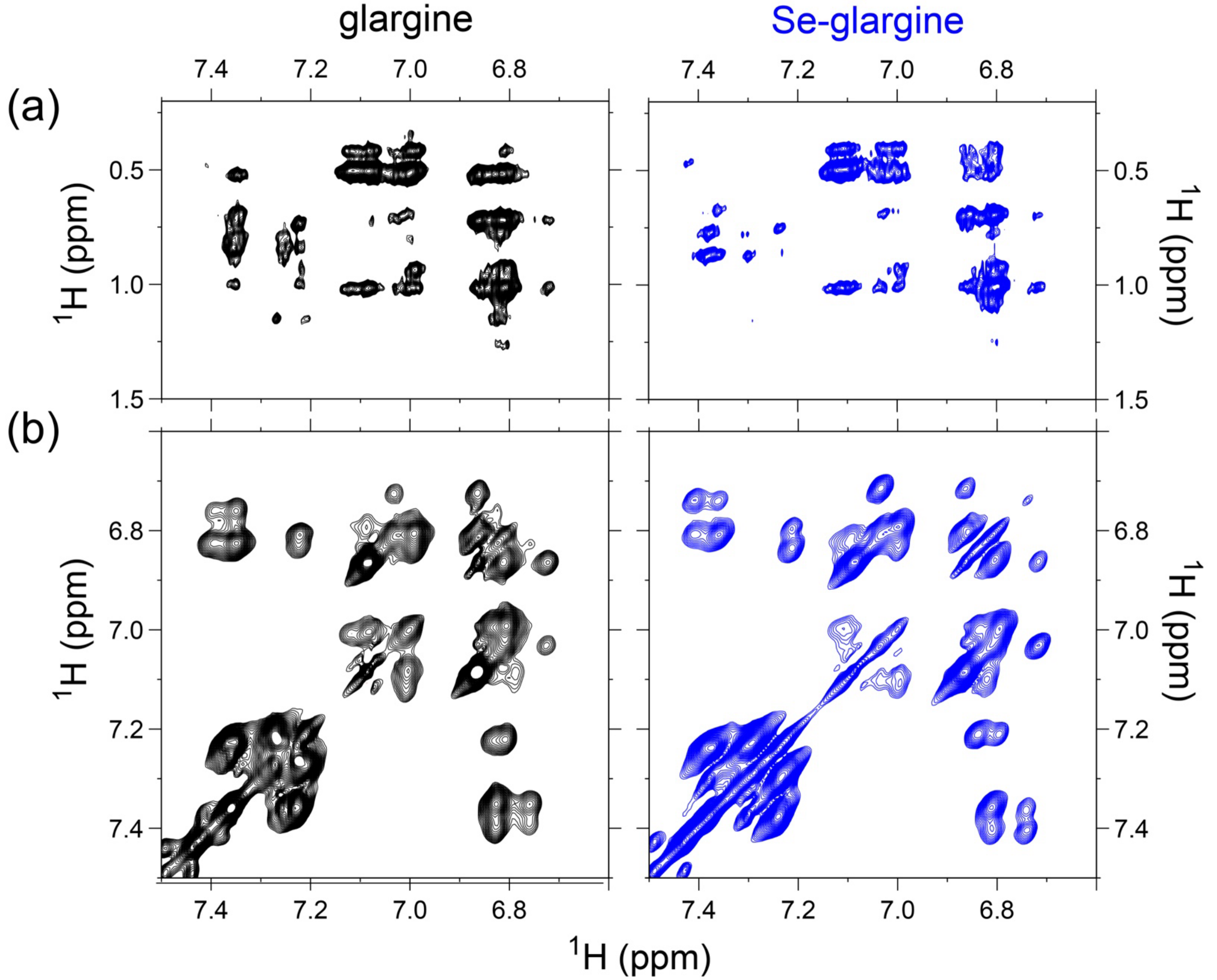

Figure S21. Homonuclear 2D-NMR spectra of glargine (*left panel, black*) and Se-glargine (*right panel, blue*): (a) NOESY spectra (mixing time 150 ms) showing NOEs from aromatic protons to methyl protons and (b) TOCSY spectra (mixing time 55 ms) showing aromatic resonance correlation. Spectra were acquired at a ^{1}H frequency of 700 MHz in 10-mM deuterated acetic acid (pH 3.0, direct meter reading) at 25 °C.

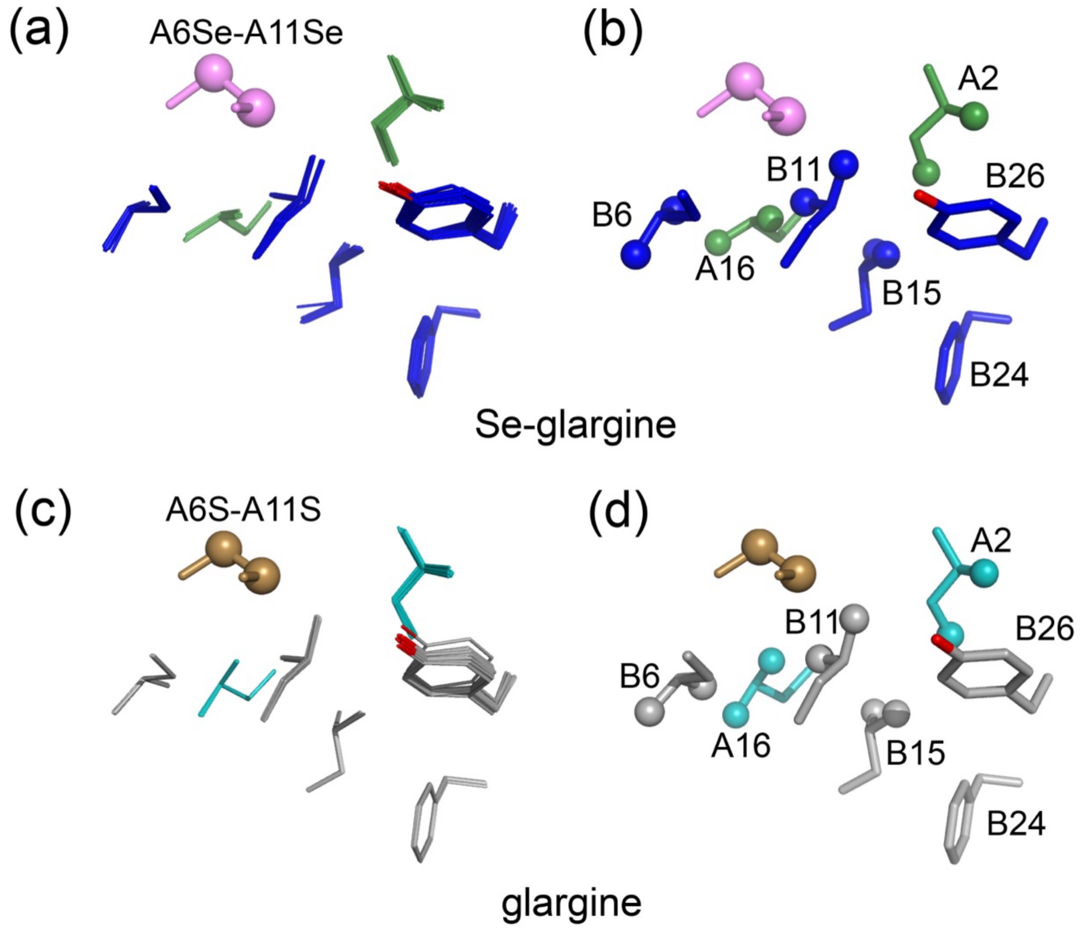

Figure S22. Structure representation of A6-A11 bridges buttressed hydrophobic residues Leu^{B6}, Leu^{B11}, Ile^{A2} and Leu^{A16}, in turn connected to the interfacial residues Leu^{B15}, Phe^{B24} and Ty^{rB26} at the dimer interface. (a) Se-glargine in the ensemble and (b) in a representative stick model. The A chain is *green* and B chain *blue.* Methyl groups represented as *green* or *blue,* diselenide bridge as *violet* spheres. (c) Glargine in the ensemble and (d) in a representative stick model. The A chain is *cyan* and B chain *gray.* Methyl groups represented as *cyan or gray,* A6-A11 disulfide bridge as *violet* spheres.

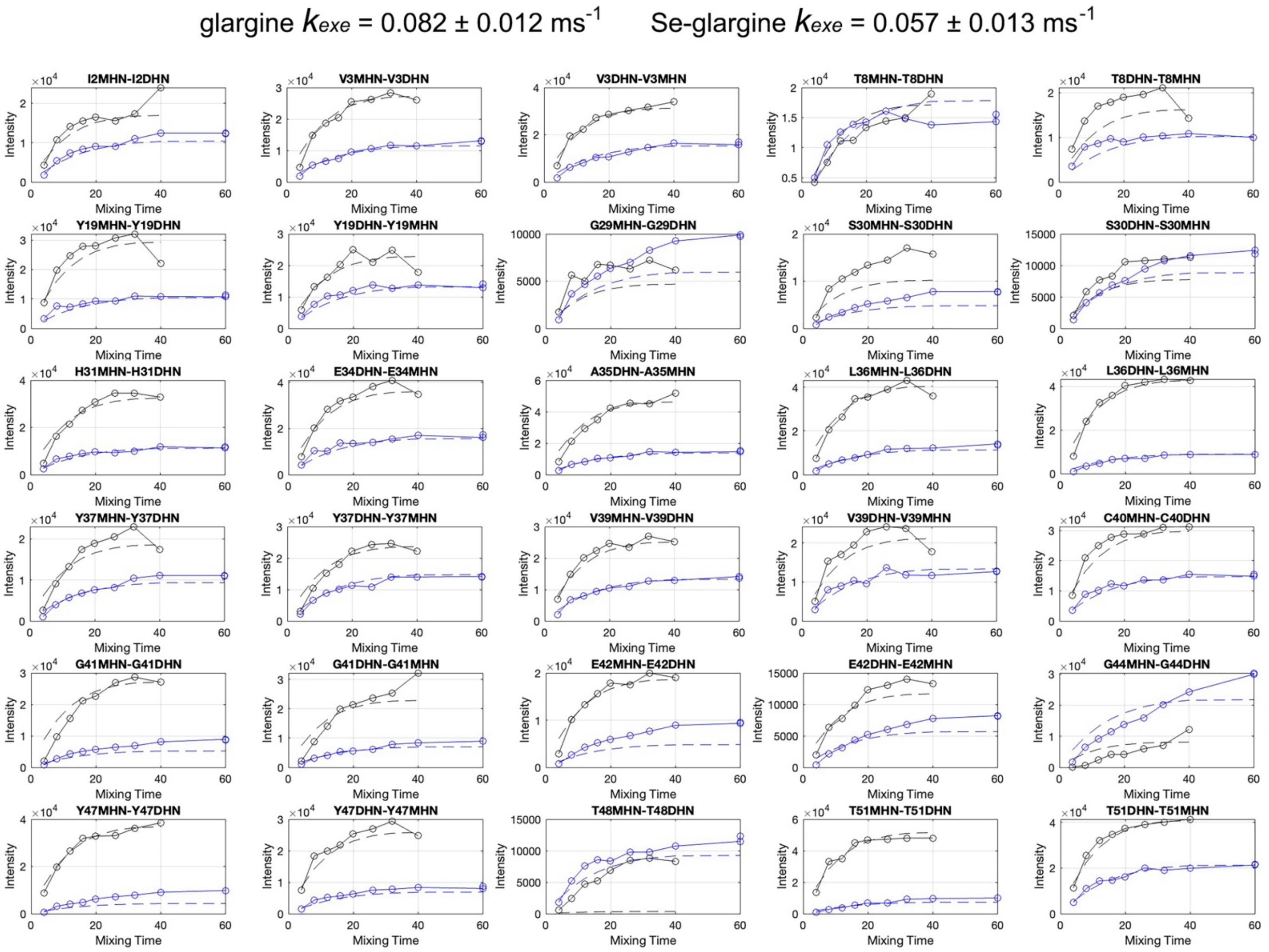

Figure S23. Monomer-dimer exchange rate build-up curve fitting for selected residues (*black*: glargine; *blue*: Se-glargine). Exchange peaks between monomer and dimer amide protons were directly integrated in 2D exchange spectra with different mixing time. The protein concentration was 100 μM of glargine (*black*) and 84 μM of Se-glargine (*blue*). The exchange rate of monomer-dimer obtained by global fitting is 0.082(±0.012) ms^{-1} for glargine and 0.057(±0.013) ms^{-1} for Se-glargine. Lifetimes are 12.3(±2.6) ms and 17.5(±3.8) ms, respectively. Data were acquired at a ^{1}H frequency of 700 MHz in H_2O (10% D_2O) in 10-mM deuterated acetic acid (pH 3.0, direct meter reading) at 25 °C.

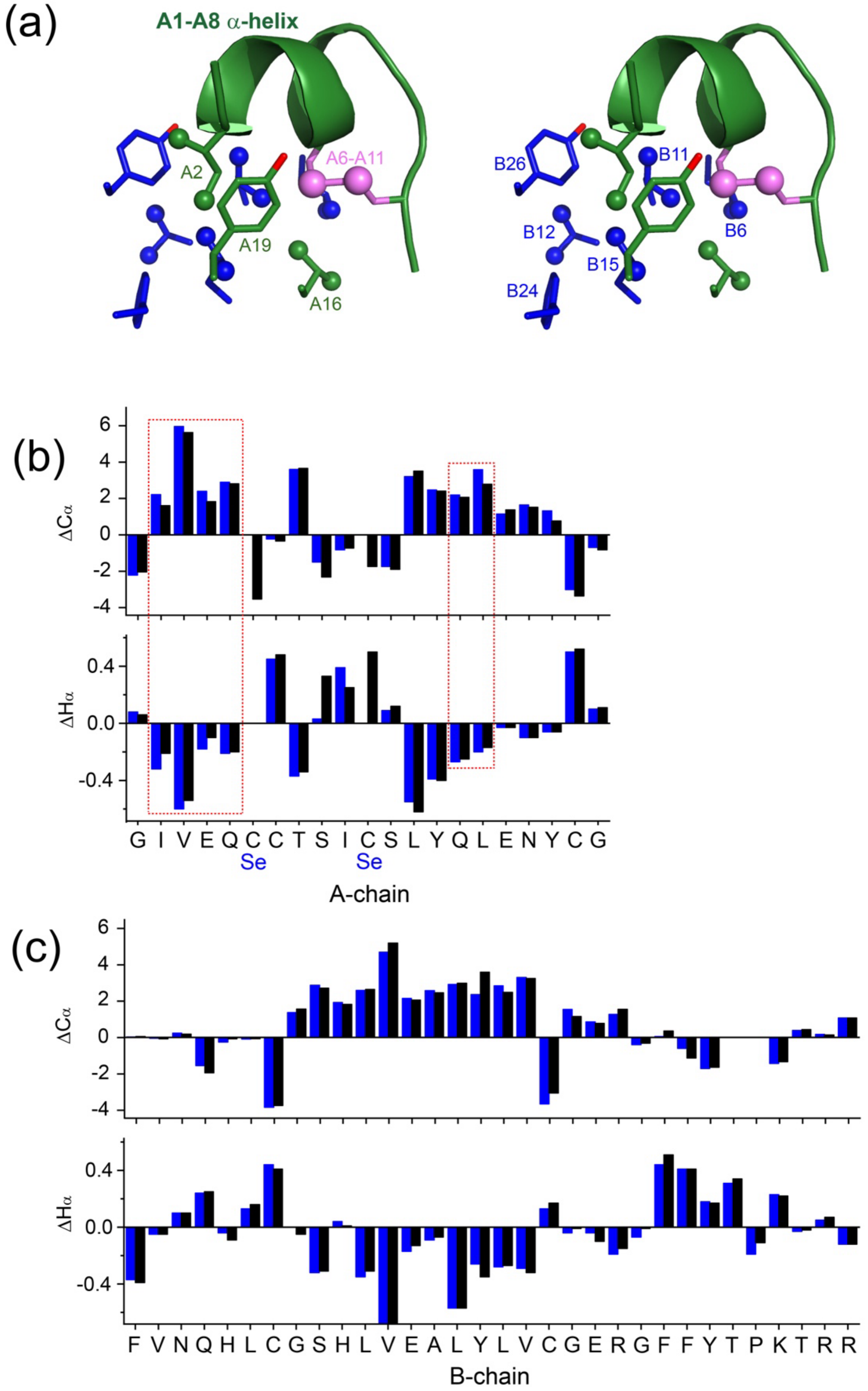

Figure S24. (a) Stereo view of structure representation of the invariant Ile^{A2} residue pinned the A1-A8 α-helix to the hydrophobic core. The A chain is shown in green, B chain in blue, A6-A11 diselenide bridge in violet. Methyl groups represented as *green* (A chain) or *blue spheres* (B chain). (b, c) Secondary shift of alpha-carbon and alpha-protons of A-chain residues (*panel b*) and B-chain residues (*panel c*) for Se-glargine (*blue*) and glargine (*black*) in 10-mM deuterated acetic acid at pH 3.0 (direct meter reading) at 25 °C. dashed boxes indicated residues that are sensitive to selenium replacement at A6 and A11 position. A6-A11 diselenide bond induced $^{1}H_{\alpha}$, $^{1}H_{N}$ and $^{13}C_{\alpha}$ chemical shift of these residues changing toward alpha-helix direction.

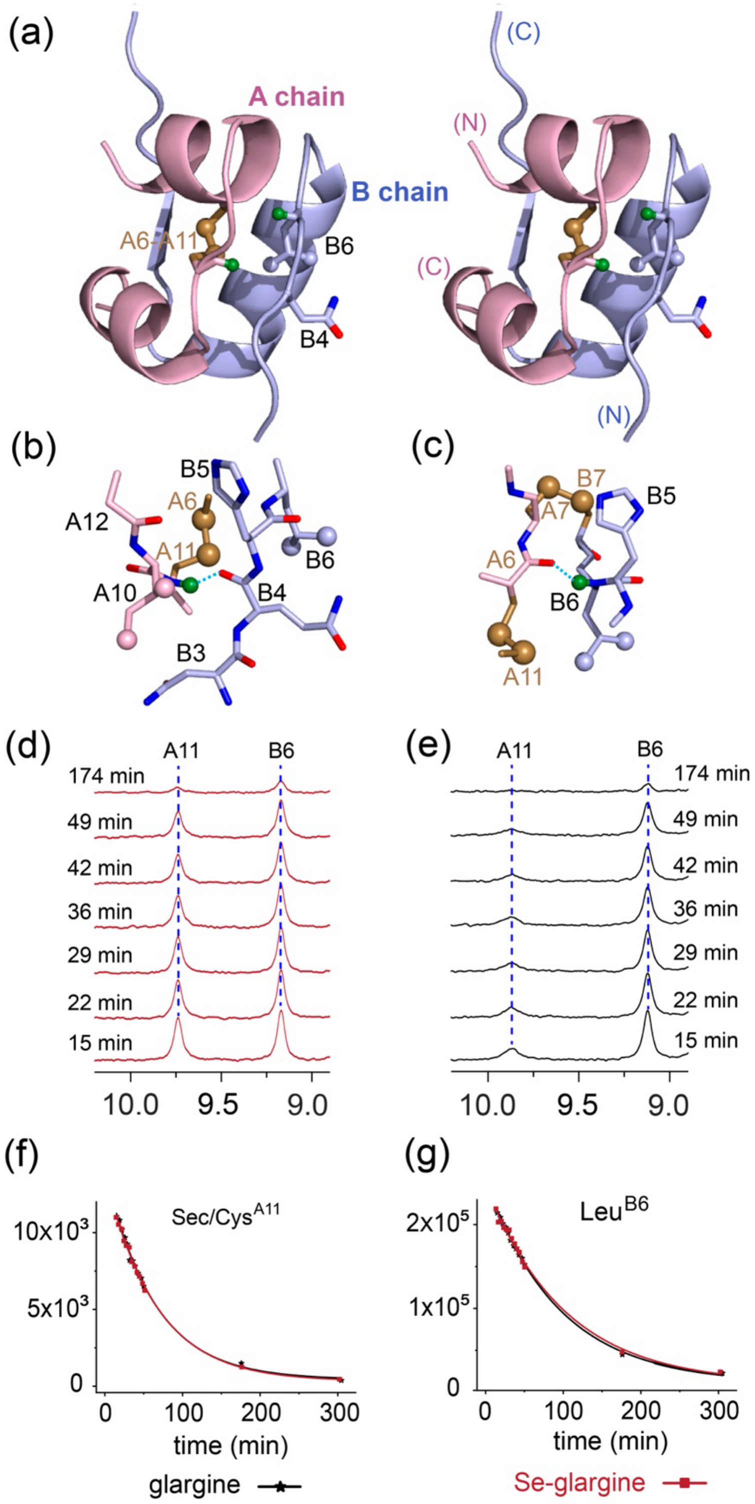

Figure S25. (a) Stereo view of structure representation of A6-A11 bridge-related hydrogen bond (PDB-ID: 4INS). The A chain is in *light pink,* B chain in *light blue* and A6-A11 disulfide bridge in *gold.* The amide protons of A11 and B6 are shown in *green spheres*, the oxygen atoms of B4 and A6 in *red spheres*. Local environment of Sec^{A11}HN-Gln^{B4}CO H-bond (b) and Leu^{B6}HN-Sec^{A6}CO H-bond (c). The color code is same as in panel (a) and dashed lines indicate hydrogen bonds. Successive 1D ^{1}H NMR spectra of A6-A11 diselenide mutant (*panel* d, *maroon*) and glargine (*panel* e, *black*) in far downfield region at the stated time points after dissolving the protein in 100% D_2O in 10% deuterated acetic acid (pH 2.1, direct meter reading) at 25 °C. The 1D data fitting curves of amide-proton ^{1}H-^{2}H exchange for Sec/Cys^{A11} residue (*panel* f) and Leu^{B6} residue (*panel* g) in 10% deuterio-acetic acid at pH 2.1 at 25 °C.

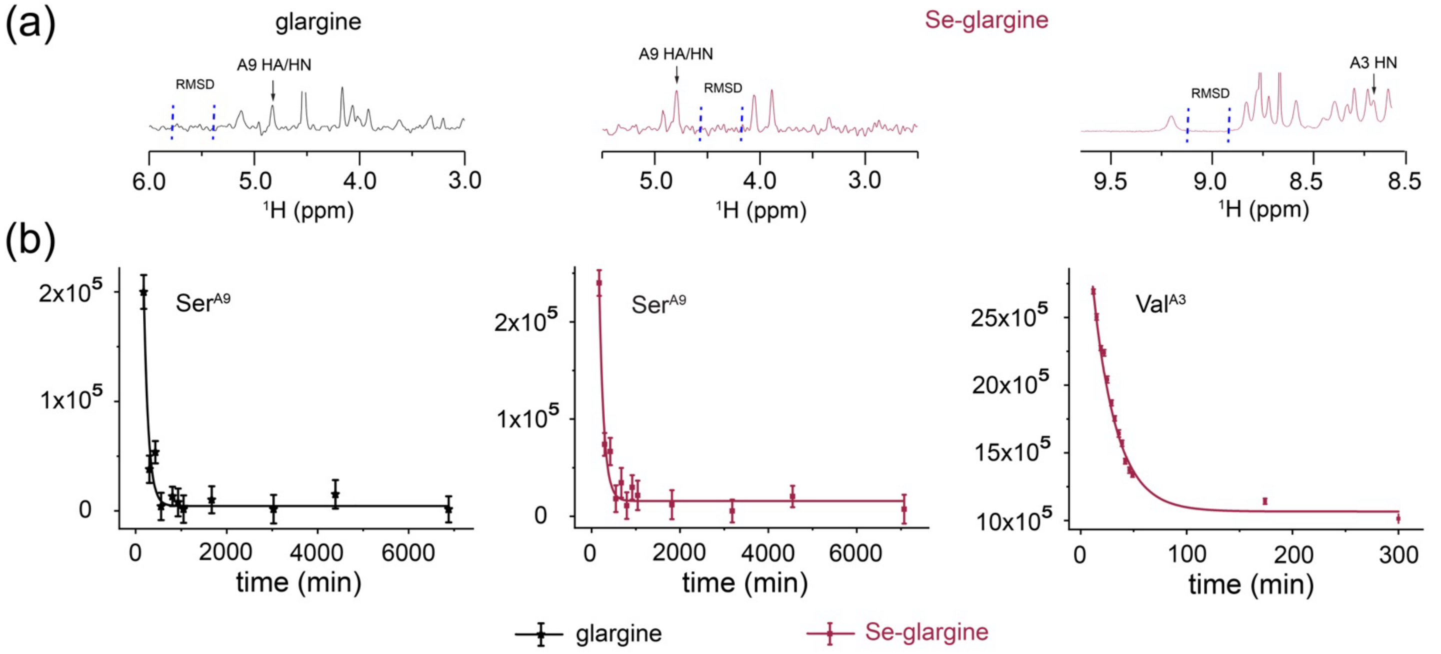

Figure S26. (*a*) The spectral trace of first 2D-TOCSY after adding fresh D_2O for ^1H-^2H exchange in 10% deuterated acetic acid at pH 2.1 at 25 °C: Ser^{A9} H_α/H_N cross-peak of glargine (*left panel, black*) and Se-glargine (*middle panel, maroon*). The *right panel* showed first 1D spectrum of Se-glargine to display amide proton signal of Val^{A3}. The RMSD noise level was measured in the spectral region between *blue* dashed lines. (*B*) The 2D data fitting curves of amide-proton ^1H-^2H exchange for Ser^{A9} in glargine (*left panel, black*) and Se-glargine (*middle panel, maroon*). The *right panel* was 1D data fitting of Val^{A3} in Se-glargine.

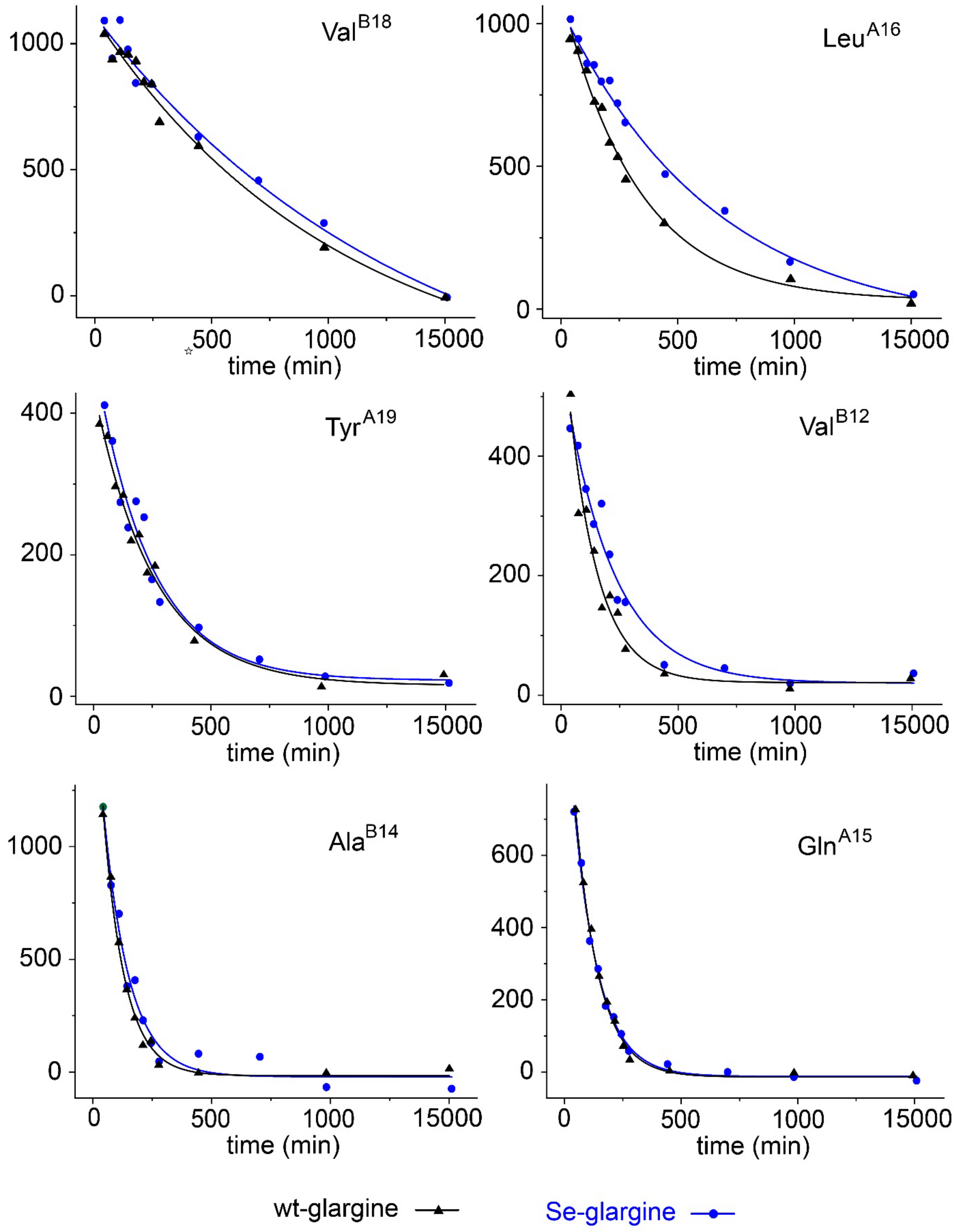

Figure S27. Representative examples of exponential ^{1}H-^{2}H exchange at specific residues associated with subglobal- or global exchange kinetics as defined in the glargine insulin (*black*) and Se-glargine (*blue*). Data were analyzed by 2D amide-proton ^{1}H-^{2}H Exchange spectra in 10-mM deuterated acetic acid (pH 3.0, direct meter reading) at 25 °C.

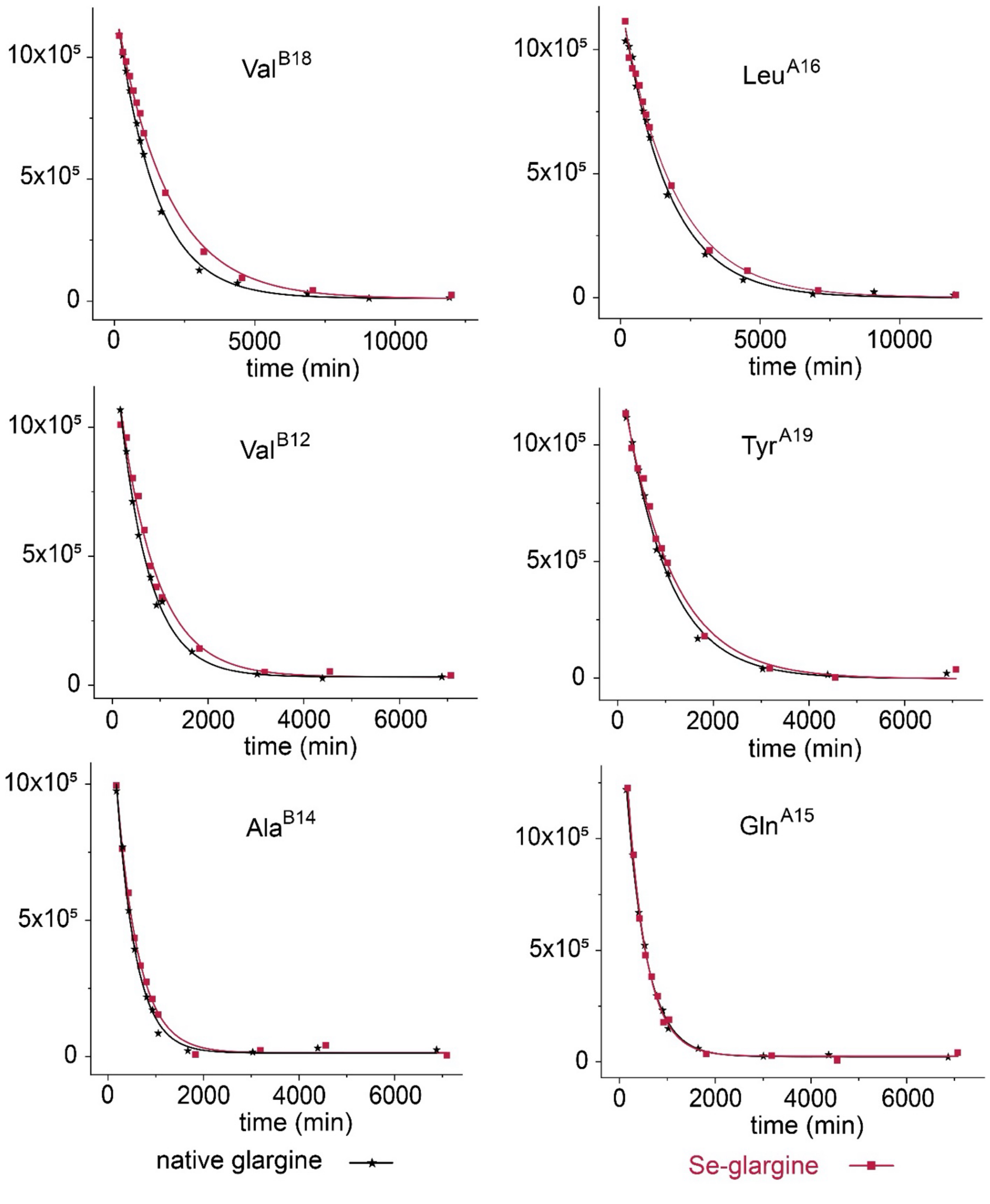

Figure S28. Representative examples of exponential ^{1}H-^{2}H exchange 10% deuterated acetic acid at specific residues associated with subglobal- or global exchange kinetics as defined in the glargine insulin (*black*) and Se-glargine (*maroon*). Analysis of global exchange corroborates with increase in ΔG_u due to selenium incorporation at Cys^{A6} and Cys^{A11} sites.

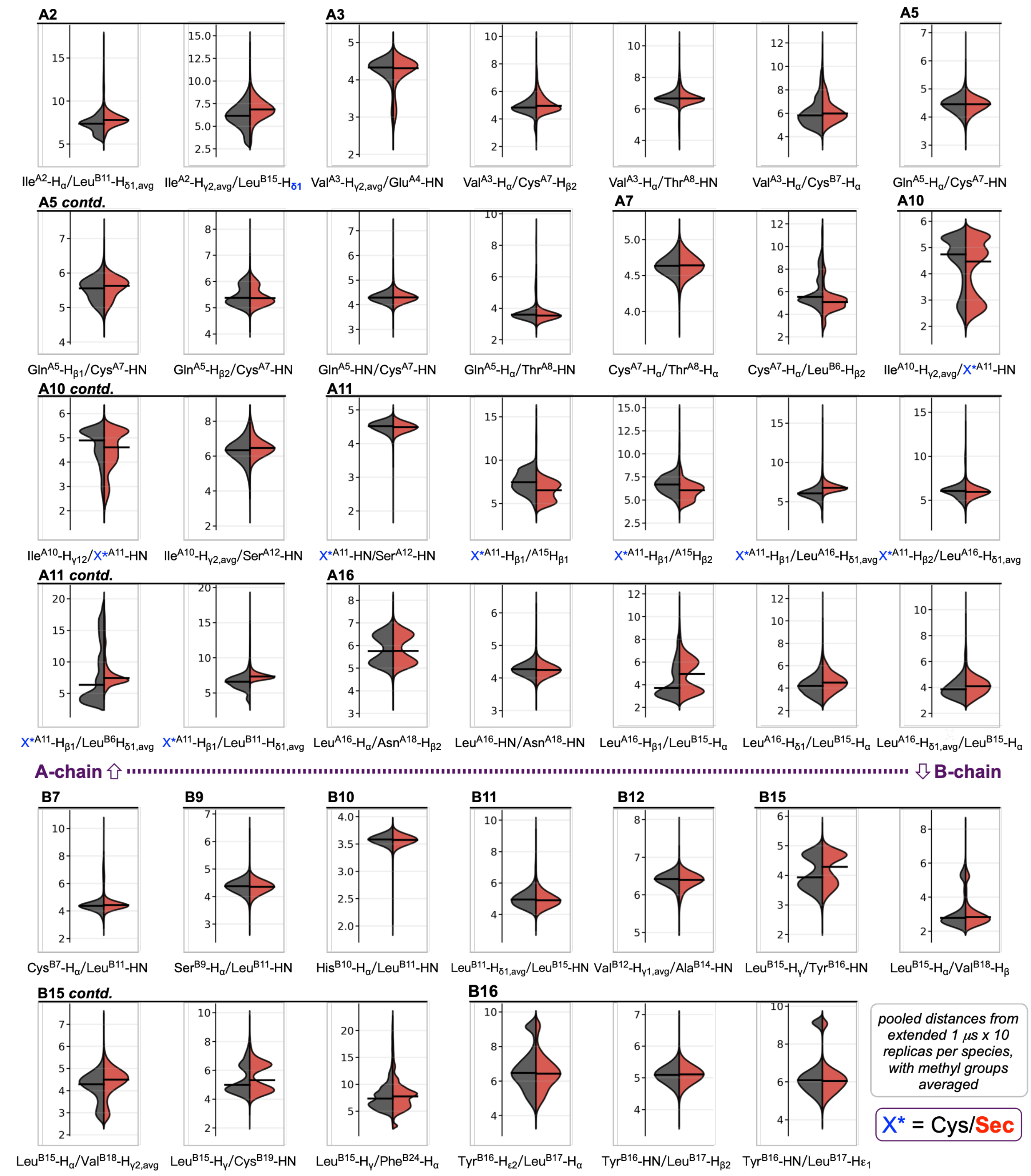

Figure S29. Complementary MD distance analysis for reported NOEs (Table S7). Split-violin plots show pooled NOE-relevant interproton distance distributions for WT glargine and Se-glargine (A6–A11 diselenide) from 10 independent 1-μs MD replicas per system. Distances are reported as NOE-like effective distances, $r_{\text{scaled}} = \langle r^{-6}\rangle^{-1/6}$ thereby weighting shorter distances more strongly. WT is shown on the left half (gray) of each violin and Se-glargine on the right (red). Methyl groups were treated by three-proton averaging prior to distance evaluation. Panels are organized by the residue of the first proton in each pair and separated into A- and B-chain parts. The plots provide NOE-relevant distance *proxies* rather than full back-calculated NOEs.

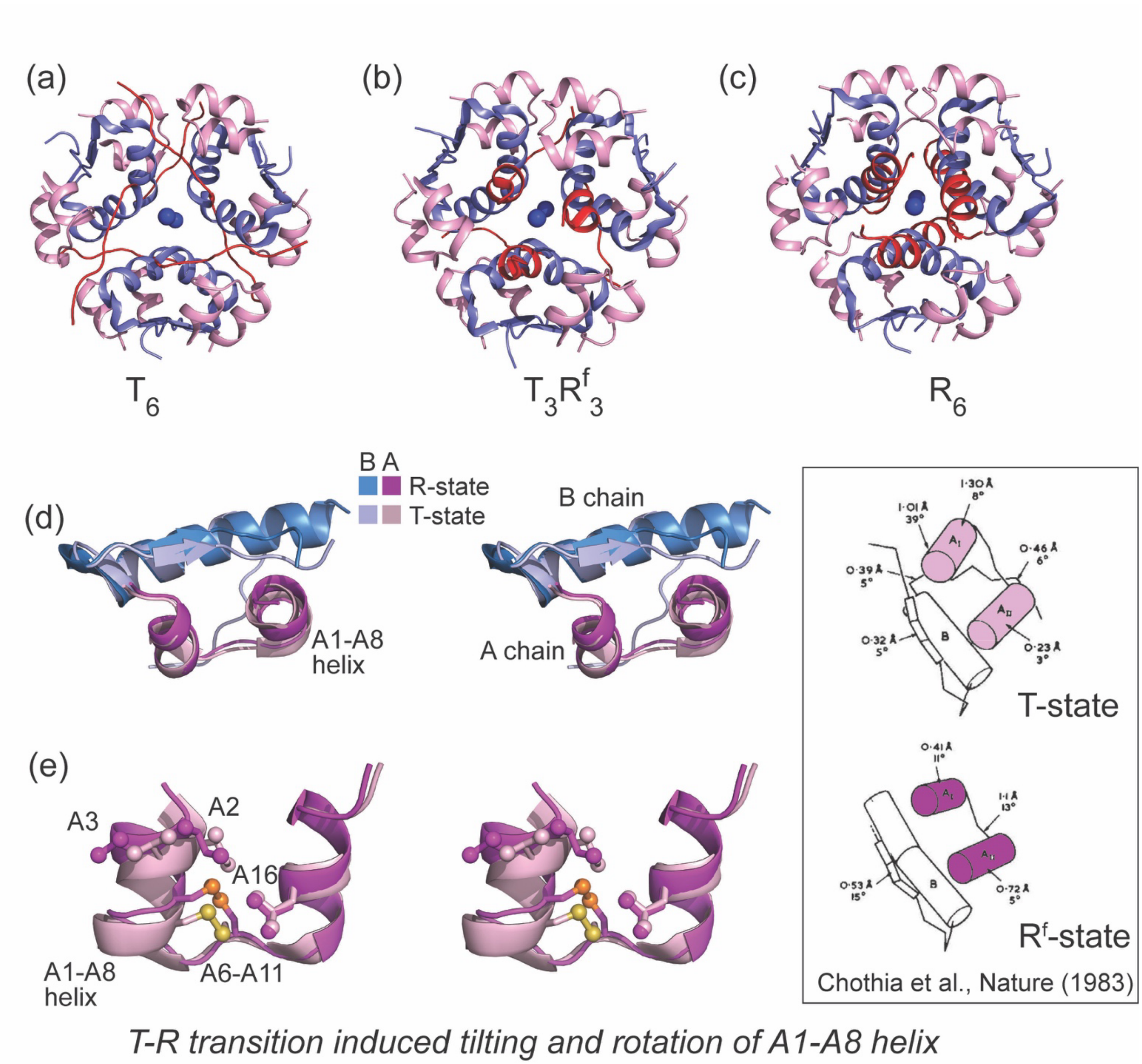

Figure S30. T-R conformational transition. Structures: (a) T_6 hexamer (PDB-ID: 4INS), (b) $T_3R^f_3$ hexamer (PDB-ID: 1TRZ), (c) R_6 hexamer (PDB-ID: 1ZNJ). (d) overlay of T-state and R-state monomer with respect to A12-A20 and B9-B19 helices show tilting and rotation of A1-A8 helix as previously shown by Chothia and colleagues (inset, adapted from reference: (Chothia et al., 1983). (e) close view from the overlay with key residues and A6-A11 diselenide bridge highlighted. Color coding: A chain (pink), B chain (blue), methyl groups and disulfides (gold) are shown as spheres with one third van der Waals radii.

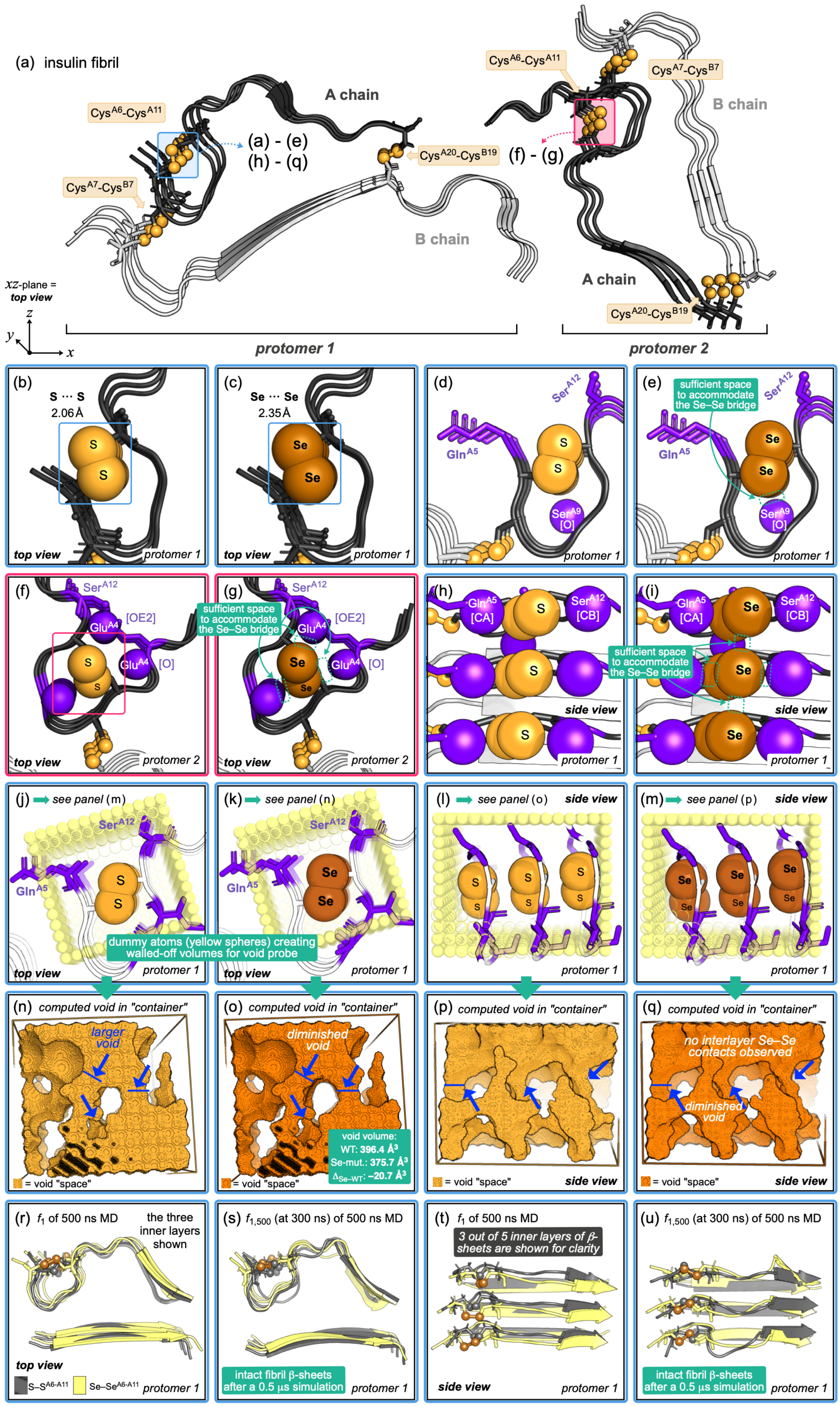

(a) insulin fibril
A chain
B chain
Cys^{A6}-Cys^{A11}
Cys^{A7}-Cys^{B7}
Cys^{A20}-Cys^{B19}
(a) - (e)
(h) - (q)
(f) - (g)
xz-plane = top view
protomer 1
protomer 2
(b) S ··· S 2.06 Å top view protomer 1
(c) Se ··· Se 2.35 Å top view protomer 1
(d) Ser^{A12} Gln^{A5} Ser^{A9} [O] protomer 1
(e) sufficient space to accommodate the Se–Se bridge
(f) Ser^{A12} Glu^{A4} [OE2] Glu^{A4} [O] top view protomer 2
(g) sufficient space to accommodate the Se–Se bridge
(h) Gln^{A5} [CA] Ser^{A12} [CB] side view protomer 1
(i) sufficient space to accommodate the Se–Se bridge side view protomer 1
(j) see panel (m)
(k) see panel (n)
(l) see panel (o) side view
(m) see panel (p) side view
dummy atoms (yellow spheres) creating walled-off volumes for void probe
(n) computed void in "container"
larger void
= void "space"
(o) computed void in "container"
diminished void
void volume:
WT: 396.4 Å^3
Se-mut.: 375.7 Å^3
Δ_{Se-WT}: −20.7 Å^3
(p) computed void in "container" side view
(q) computed void in "container"
no interlayer Se–Se contacts observed
diminished void
side view
(r) f_1 of 500 ns MD
the three inner layers shown
top view
S–S^{A6-A11}
Se–Se^{A6-A11}
(s) $f_{1,500}$ (at 300 ns) of 500 ns MD
intact fibril β-sheets after a 0.5 μs simulation
(t) f_1 of 500 ns MD
3 out of 5 inner layers of β-sheets are shown for clarity
side view
(u) $f_{1,500}$ (at 300 ns) of 500 ns MD
intact fibril β-sheets after a 0.5 μs simulation

Figure S31. Structural accommodation and packing consequences of a modeled A6–A11 diselenide within an insulin fibril scaffold. **(*a*)** cryo-EM insulin fibril model (PDB–ID: 8SBD; Wang *et al.*, 2023) showing two representative protomers and the three native bridges (A6–A11, A7–B7, A20–B19). The A- and B chains are shown as dark gray and white ribbons, respectively, and bridges are shown as spheres. A diselenide analog at A6–A11 was generated by PyMOL replacement and torsion (χ_3) adjustment to increase the chalcogen–chalcogen distance from the native S–S value (~2.06 Å) to a Se–Se value (~2.35 Å), while leaving A7–B7 and A20–B19 unchanged. The coordinate axis indicates the viewing orientation, with the *xz*-plane corresponding to the "top" view. **(*b–e, h–l*)** *Protomer 1* close-ups of the A6–A11 region comparing S–S (*b,d,h*) versus Se–Se (*c,e,i*); neighboring side chains (purple; e.g., Gln^{A5} and Ser^{A9}) indicate sufficient local free volume to accommodate the longer Se–Se bridge without obvious steric clashes in this *ad hoc* model. **(*f–g*)** Equivalent views for *protomer 2* and the adjacent packing environment (purple; e.g., Glu^{A4} and Ser^{A12}), again showing that the Se–Se model can be placed without apparent interprotomer interference. **(*j–m*)** Construction of a walled, rectangular cuboid "container" using dummy atoms (yellow spheres, $r_{vdW,wall}$ = 0.80 Å) around the A6–A11 region and four anchor atoms (Gln5[CB], Ser12[OG], Ile10[CA], and Cys7[CA]) to define the cuboid corners; top views (*j,k*) and side views (*l,m*) are shown for S–S and Se–Se models, respectively. **(*n–q*)** MoloVol void analysis within the same container geometry (probe radius 0.20 Å; grid spacing 0.075 Å; identical settings except chalcogen vdW radii of 1.80 Å for S vs 1.90 Å for Se; Bondi, 1964) reveals a small but reproducible reduction in probe-excluded void space for the Se–Se model (*n,o*; with corresponding side views *p,q*), while preserving interlayer separation and showing no interlayer Se···Se contacts. Blue arrows and guidelines are shown to highlight subtle changes in void volume. **(*r–u*)** MD simulations of the 5-layer fibril model containing the A6–A11 Se–Se substitution (only the three interior layers shown for clarity): overlays of the starting structure (gray = WT; yellow = $Se–Se^{A6–A11}$) and the structure after 0.5 µs from the top- (*r,s*) and side (*t,u*) views show an intact fibril architecture without layer detachment, consistent with only modest local packing adjustments near the A6–A11 site. It is important that only direct comparisons should be made between matched S–S vs Se–Se panel pairs generated under the same view and settings (e.g., *b* vs. *c, d* vs. *e*, *f* vs. *g*, *h* vs. *i*, *j* vs. *k*, *l* vs. *m*, *n* vs. *o*, and *p* vs. *q).*

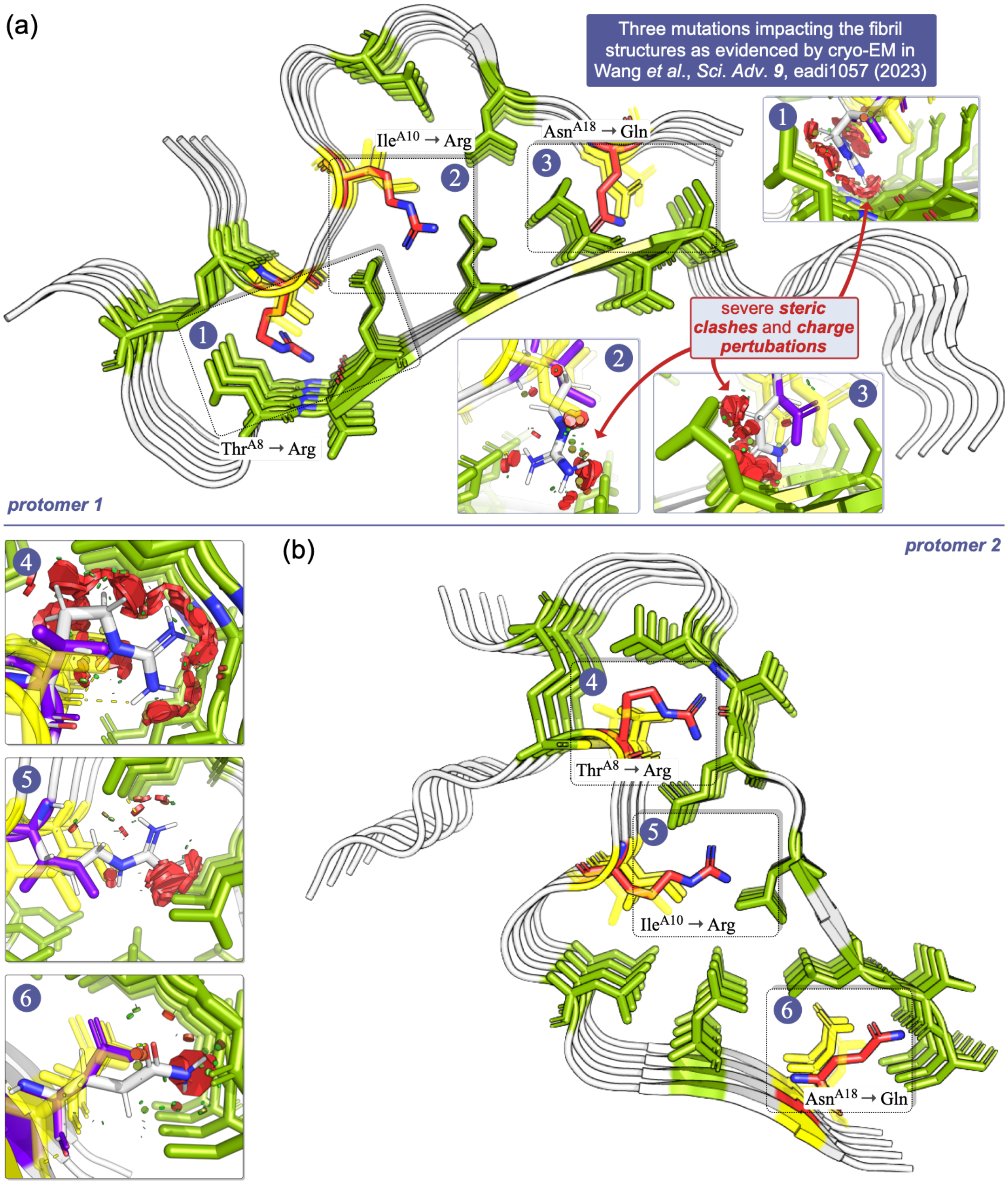

Figure S32. PyMOL mutagenesis-based visualization of severe fibril-disrupting substitutions in the insulin cryo-EM fibril scaffold (PDB–ID: 8SBD; Wang *et al*., 2023), shown to contrast the much subtler S→Se$^{A6–A11}$ substitution. Panels (*a*) and (*b*) show the two protomer contexts within the fibril model, with the three reported substitutions highlighted (labeled **1**–**3**). Numbered zoom panels (**1**–**6**) show local packing around each mutation site in the two protomers. In the inserts, neighboring residues are shown in green; the corresponding WT side chain is shown in yellow in nearby fibrils and magenta which had the substitution); and the modeled mutant rotamer with the lowest PyMOL clash score is shown in red in (*a*) and white in the insert panels. PyMOL clash indicators are displayed as red spheres/cylinders (severe clashes) and yellow cylinders (minor clashes). These substitutions represent substantially larger and/or charge-altering perturbations (two include charge changes), and all three occur at internally packed fibril positions rather than solvent-exposed sites, making local accommodation and reorganization more energetically costly. The visualized clashes are shown for a *single mutation* event in one local filament environment; in the full repeating fibril assembly, analogous steric and electrostatic penalties would be propagated across stacked layers, further reducing feasibility of maintaining the same packing arrangement. This behavior contrasts with the modeled A6–A11 diselenide substitution, which is a comparatively subtle geometric perturbation (primarily a longer Se–Se bond with modest steric expansion) and is therefore considered more compatible with fibril formation, consistent with experimental evidence indicating *increased lag time* rather than complete suppression of fibrillation.

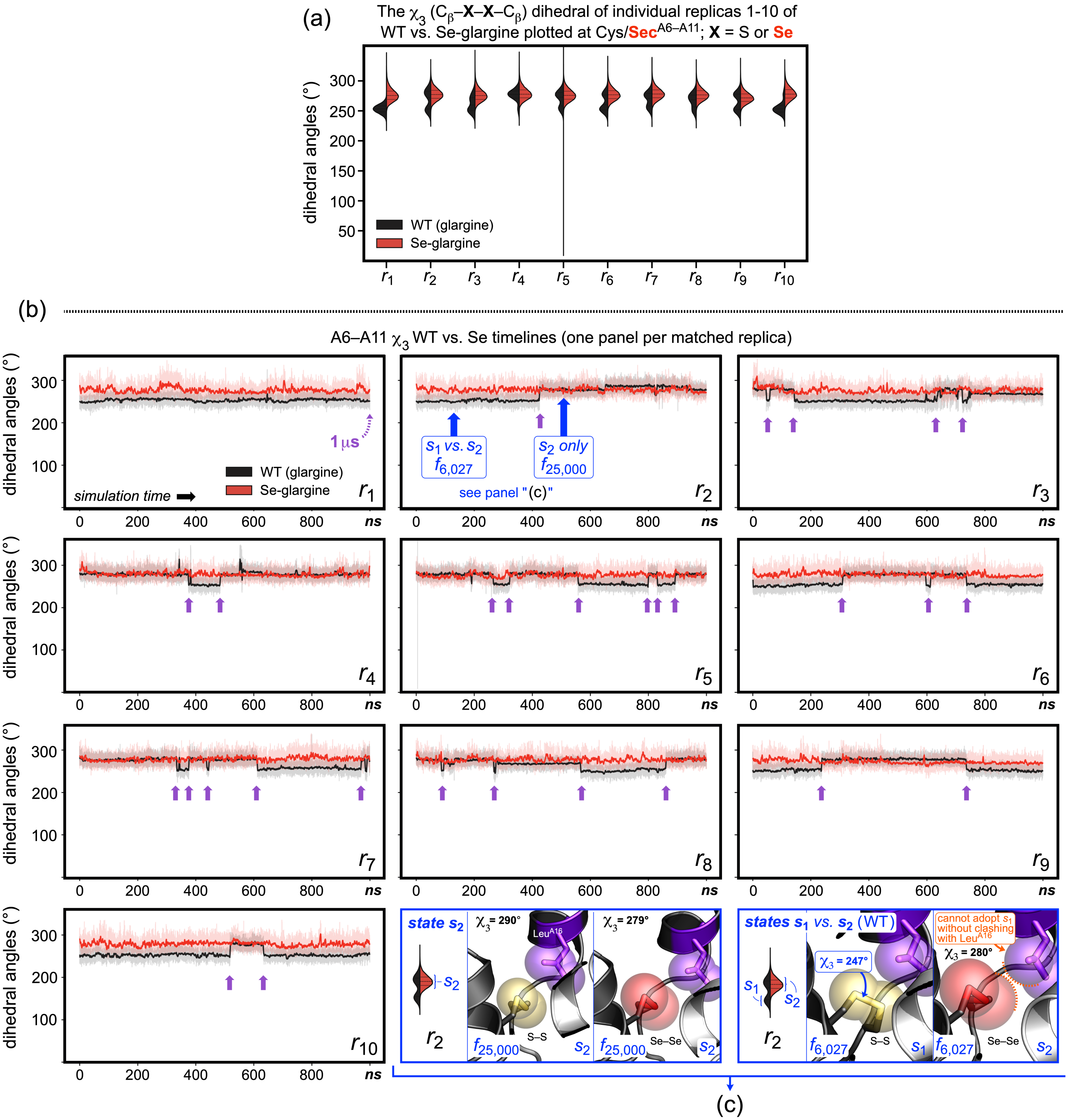

Figure S33. A6–A11 χ_3 torsion in WT glargine versus Se-glargine from 1-μs simulations with expanded replica sampling. The χ_3 dihedral of the A6–A11 bridge, defined as C_β(A6)–**X**(A6)–**X**(A11)–C_β(A11) (**X** = S for glargine; **X** = Se for Se-glargine), is plotted in degrees. Ten independent 1-μs replicas were analyzed for each system (r_1–r_{10}). (a) Split violin plots show the χ_3 distribution for each replica (WT, black; Se-glargine, red). (b) Replica-matched χ_3 timelines show the time evolution of χ_3 over 1 μs; purple arrows (⇑) mark WT transitions between the two major χ_3 substates. (c) Representative structures from replica r_2 define these substates: s_1 ($\chi_3 \approx 247°$), sampled only by WT, and s_2 ($\chi_3 \approx 279$–$290°$), sampled only by WT and Se-glargine. Thus, the WT disulfide can adopt both s_1 and s_2, whereas the Se-glargine diselenide is restricted to s_2, because the s_1 geometry is incompatible with the local packing environment and would clash with Leu^{A16}.

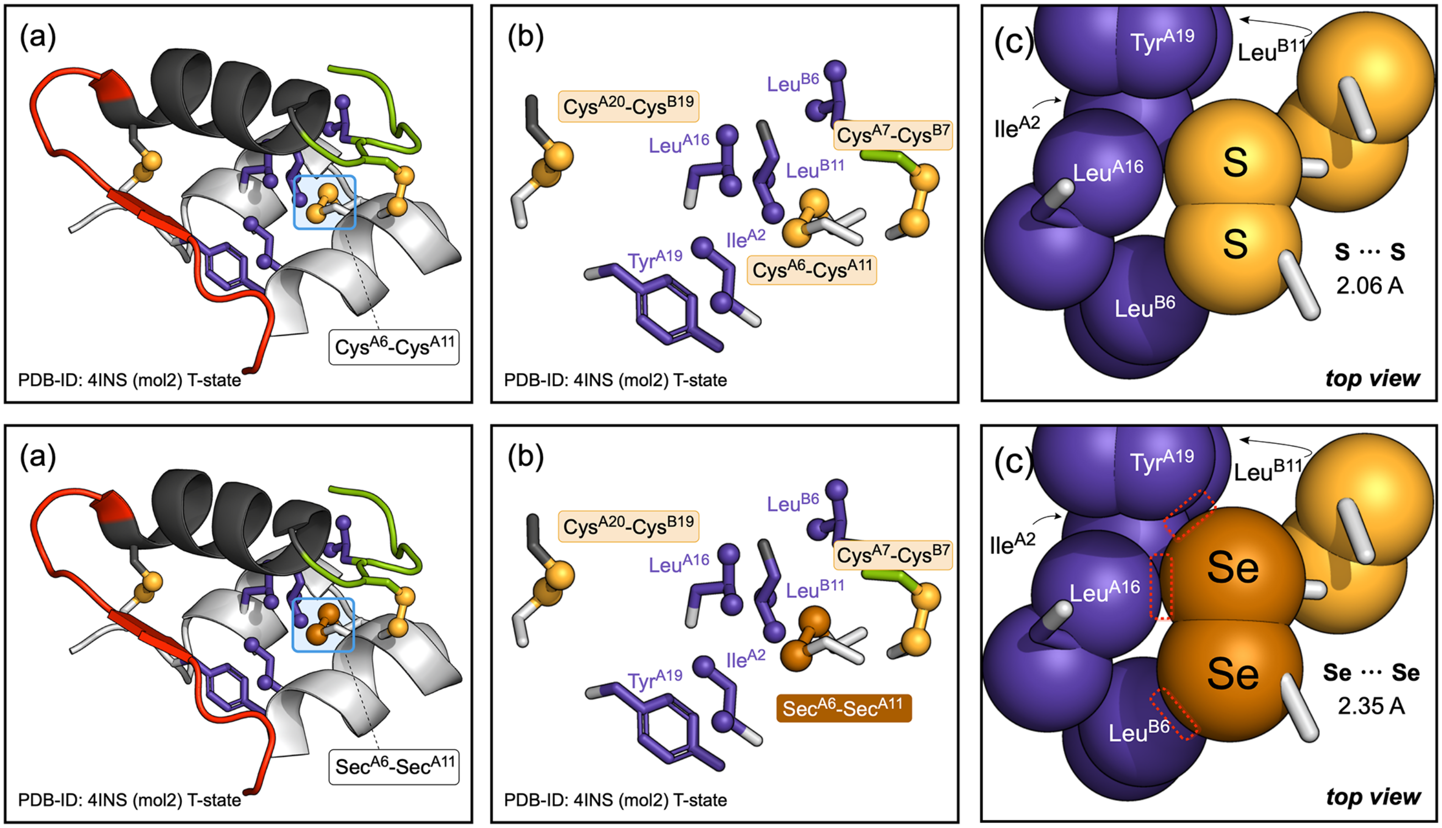

Figure S34. Nearest-neighbor contacts and van der Waals packing at the A6–A11 bridge in 4INS: WT Cys–Cys (S–S, top) versus Sec–Sec (Se–Se, bottom). (a) a ribbon overview with the A chain colored dark gray and the bridges shown as sticks (orange and dark orange for S vs. Se, respectively) with nearby residues shown in purple. (b) heavy-atom nearest neighbors to the bridge (contact cutoff in Pymol, 4.5 Å) shown as sticks and labeled in purple. (c) space-filling models of the local pocket with spheres depict atomic van der Waals radii (S in yellow, Se in orange and at scale, 10% increase). The Se–Se bridge preserves the overall geometry of A6–A11 but increases local steric occupancy, shifting hydrophobic packing among nearby side chains and reducing small voids adjacent to the bridge.

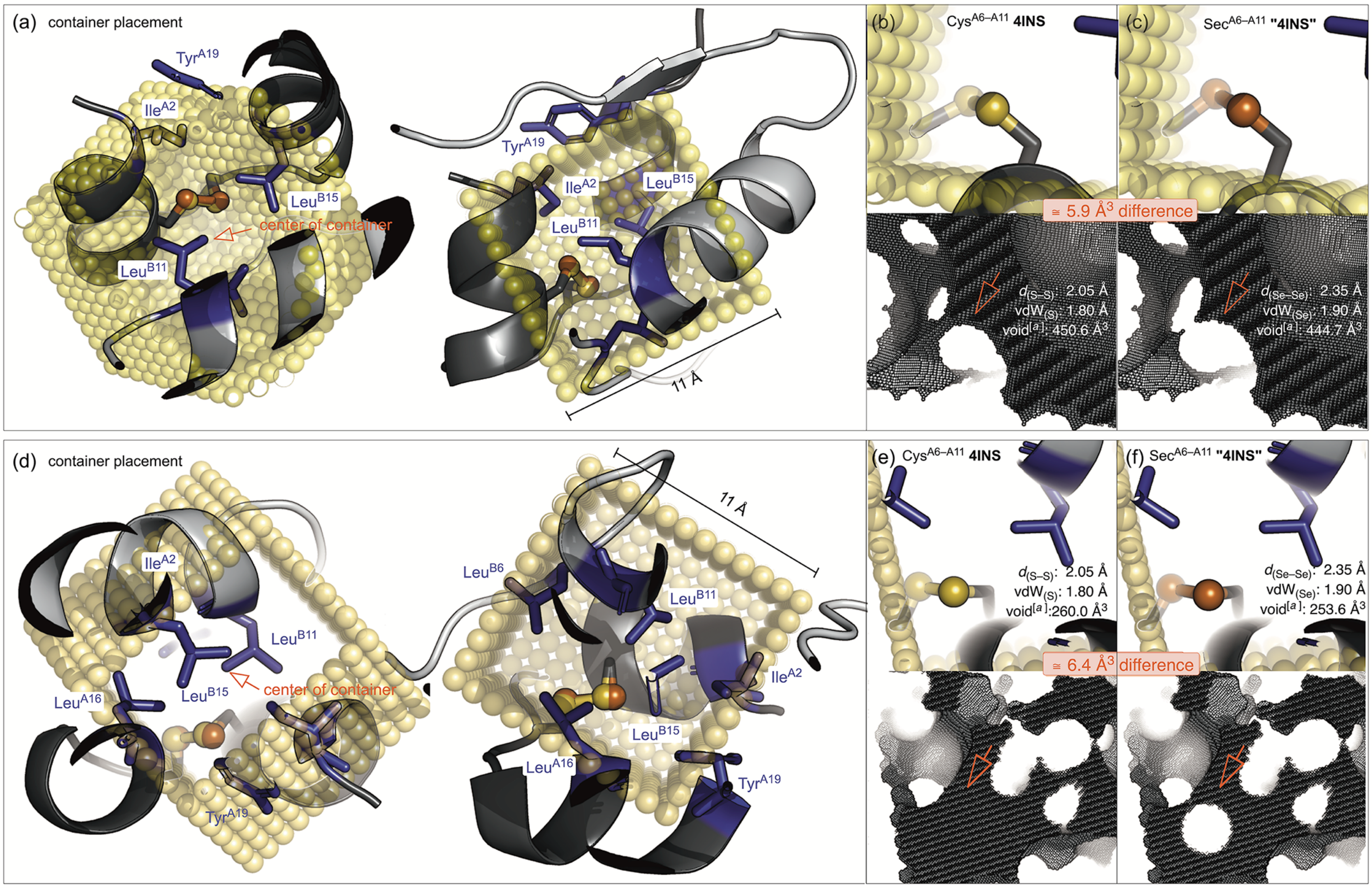

Figure S35. Container-based analysis of local packing around the A6–A11 bridge using MoloVol. (a) Placement of an 11-Å cubic container composed of dummy atoms, centered on the CD1 atom of Leu^{B11} in the crystallographic 4INS scaffold; the same container geometry is used for all subsequent calculations. Panels (b) and (c) show cross-sections of the probe-excluded void within this cube for the $Cys^{A6–A11}$ disulfide (S–S; panel b) and $Sec^{A6–A11}$ diselenide (Se–Se; panel c) variants of 4INS, with the absolute void volumes and their Se–S difference (≈5.9 $Å^3$) indicated. (d) Equivalent container placement for WT glargine after superposition of its A chain onto the A chain of 4INS to ensure that the cube samples the same spatial region in the analog. Panels (e) and (f) show the corresponding analysis for WT glargine (A6–A11 disulfide; panel e) and Se-glargine (A6–A11 diselenide; panel f), again reporting the cavity volumes within the cube and the S to Se difference (≈6.4 $Å^3$).[a] In all cases, void volumes were computed with MoloVol using identical probe radius (0.20 Å), grid spacing (0.075 Å) but with a different van der Waal (vdW) radii (1.80 *vs.* 1.90 for Se), so that the observed differences report changes in local packing around the A6–A11 bridge. Reference [a] (Maglic and Lavendomme, 2022).

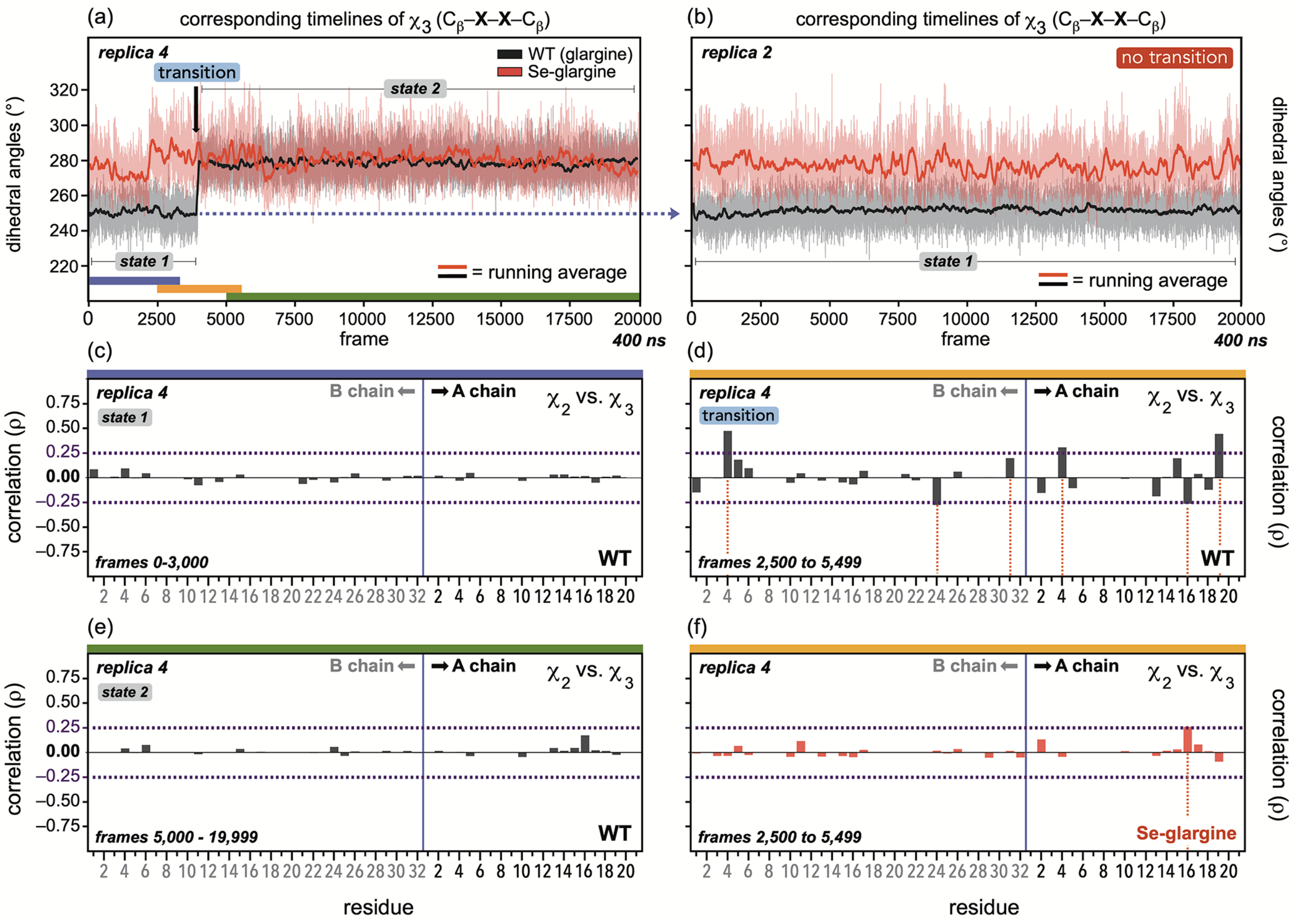

Figure S36. Expanded analysis of the χ_3 dynamics and χ_2 to χ_3 correlations for the A6–A11 bridge in WT glargine and Se-glargine. (a) Timelines of χ_3 (C_β–X–X–C_β; X = S or Se) for the A6–A11 bridge in replica 4 over 400 ns (20,000 frames). Shaded traces show instantaneous dihedral values, and solid curves represent 100-frame running averages (WT glargine in gray, Se-glargine in red). Replica 4 interconverts between two χ_3 basins ("state 1" → "state 2"; colored bars below the axis). (b) Corresponding χ_3 timelines for replica 2, which remains confined to a single basin (state 2) for both WT and Se-glargine and thus serves as a comparison trajectory without a detectable transition. (c) Pearson correlation coefficients (ρ) between χ_2 side-chain dihedrals of each residue and χ_3(A6–A11) for WT glargine, computed for the early portion of replica 4 dominated by state 1 (frames 0–2,999). (e) As in (c), but for the later portion dominated by state 2 (frames 5,000–19,999). (d) χ_2–χ_3 correlation pattern for WT glargine in the transition window of replica 4 (frames 2,500–5,499), highlighting residues with moderate positive correlations (vertical orange dashed lines) primarily in the B chain's detachable tail and near the A6–A11 region. (f) Corresponding χ_2–χ_3 correlations for Se-glargine over the same transition window; correlations are generally attenuated relative to WT, consistent with damping of propagated side-chain rearrangements by the A6–A11 diselenide staple. In panels (c–f) the B chain (B1–B32) and A chain (A1–A21) are separated by a vertical purple line, and horizontal dashed lines mark moderate Pearson's correlations of ρ = ±0.25. See Section S2.3 for additional information on Pearson R correlations.

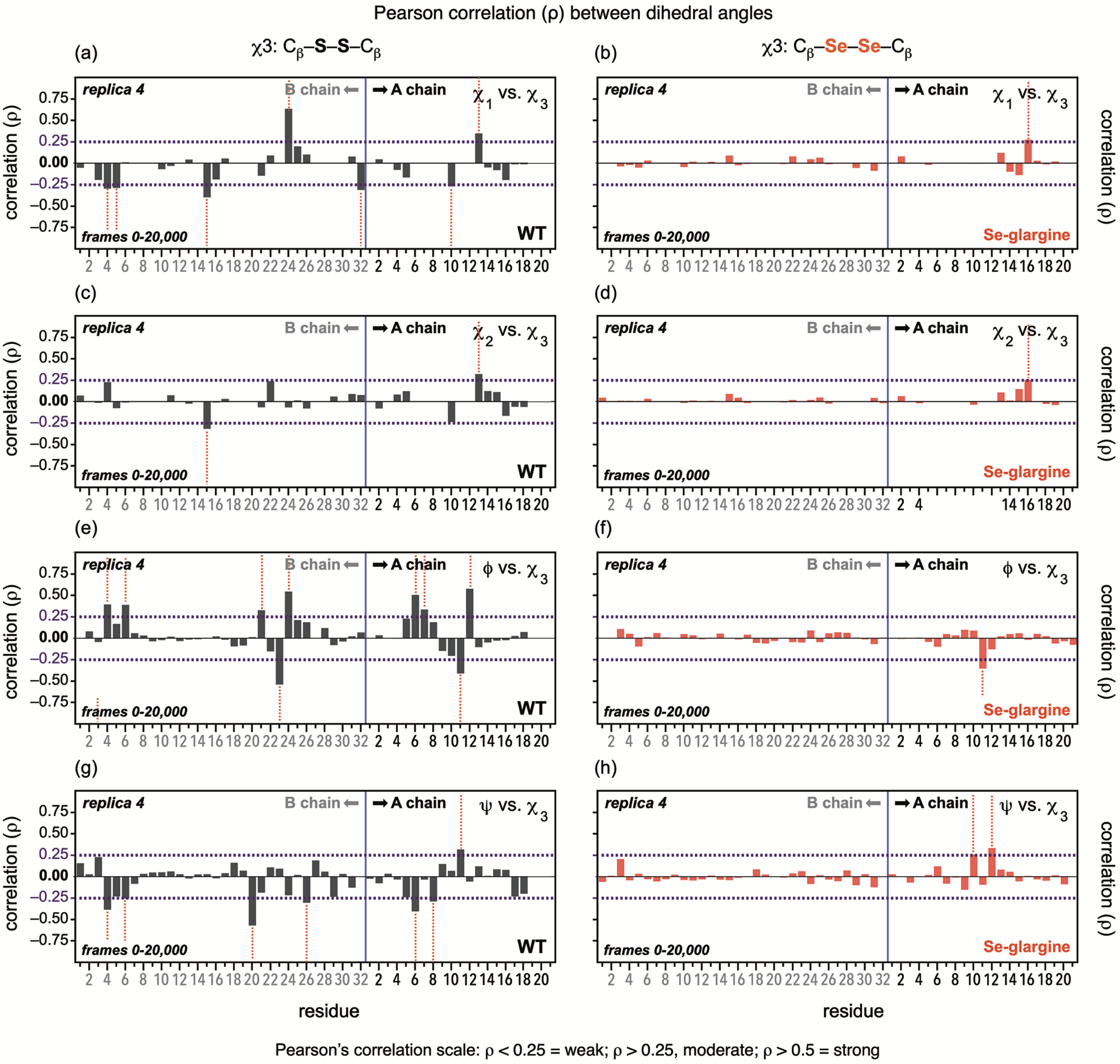

Figure S37. Pearson correlations of A6–A11 χ_3 with backbone and side-chain dihedrals over the full trajectories. Pearson correlation coefficients (ρ) between the χ_3 dihedral of the A6–A11 disulfide/diselenide bridge and χ_1, χ_2, φ, and ψ angles of each residue are shown for replica 4 of WT glargine (left, black bars) and Se-glargine (right, red bars). Rows correspond to χ_1 vs χ_3, χ_2 vs χ_3, φ vs χ_3, and ψ vs χ_3, respectively. The *x*-axis lists A- and B-chain residues; the vertical purple line marks the A/B chain boundary. Vertical orange dashed lines highlight residues in the A-chain helix and selected B-chain positions that show a moderate or larger correlation. Horizontal magenta dashed lines indicate $|\rho| = 0.25$ as a visual guide for modest correlations. Correlations are computed over all 20,000 frames of the trajectories ("all frames"). See Section S2.3 for additional information on Pearson R correlations.

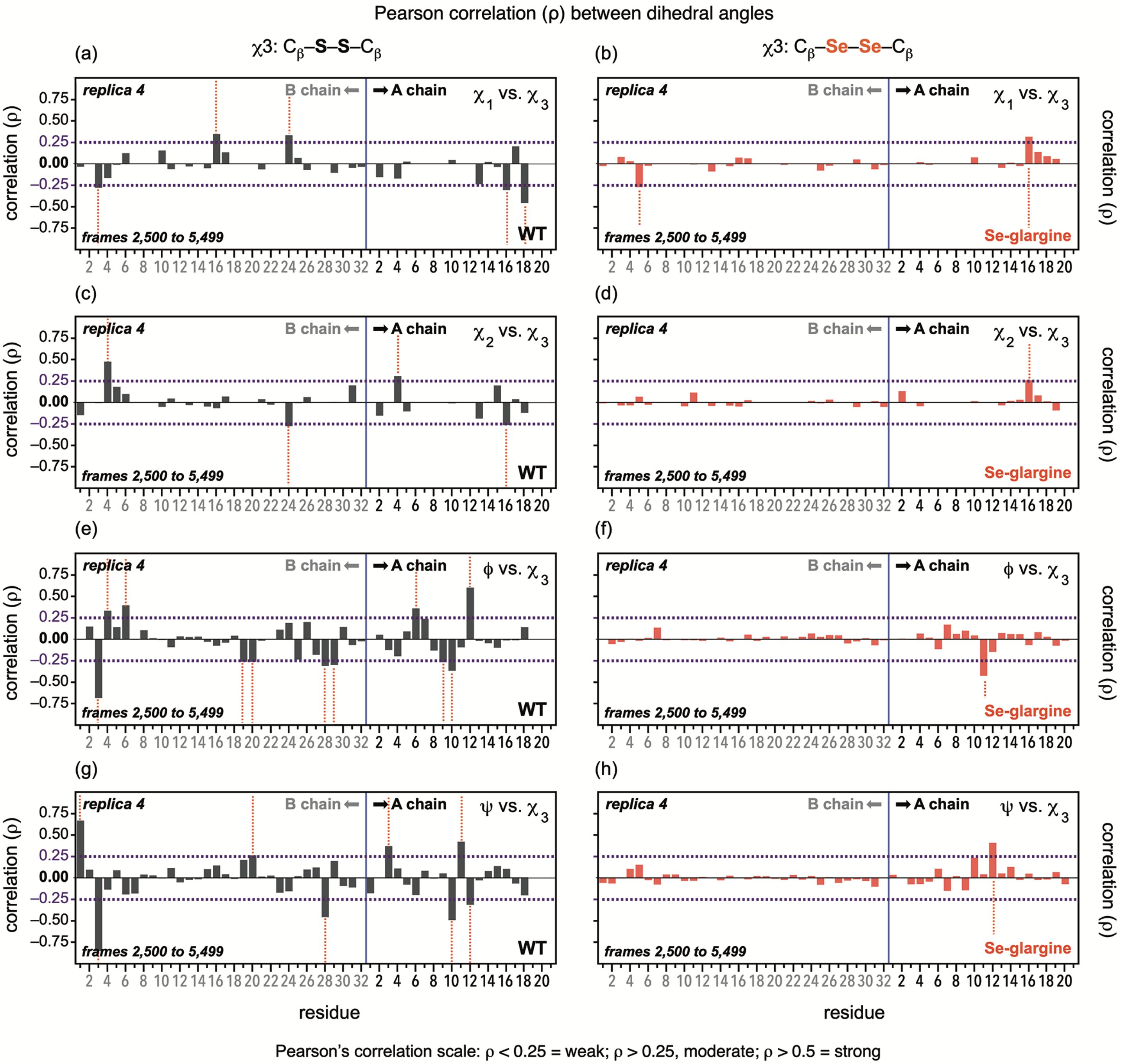

Figure S38. Pearson correlations of A6–A11 χ3 with backbone and side-chain dihedrals during the χ_3 "break" window. As in Figure S37, Pearson correlation coefficients (ρ) between the A6–A11 χ_3 dihedral and χ_1, χ_2, φ, and ψ angles of each residue are shown for replica 4 of WT glargine (left, black bars) and Se-glargine (right, orange bars). Here, correlations are calculated only for frames 2,500–5,499, the window encompassing the χ_3 switching ("break") event in WT ("frames 2,500 to 5,499"). Plot organization, residue labels, and reference lines are identical to the previous figure (purple A/B chain boundary, vertical orange dashed lines highlight residues in the A-chain helix and selected B-chain positions that show a moderate or larger correlation, magenta horizontal dashed lines at |ρ| = 0.25). Within this restricted window WT shows slightly accentuated local correlations near the bridge, whereas Se-glargine—whose χ_3 remains in a single rotamer—continues to exhibit generally weak χ_3 coupling across the scaffold. See Section S2.3 for additional information on Pearson R correlations.

Corresponding timeline plot of the **A6–A11** χ_3 (Cβ–**X**–**X**–Cβ) dihedral of individual replicas (r_1-r_4) with WT vs. Se-glargine at A6-A11; **X** = S or Se

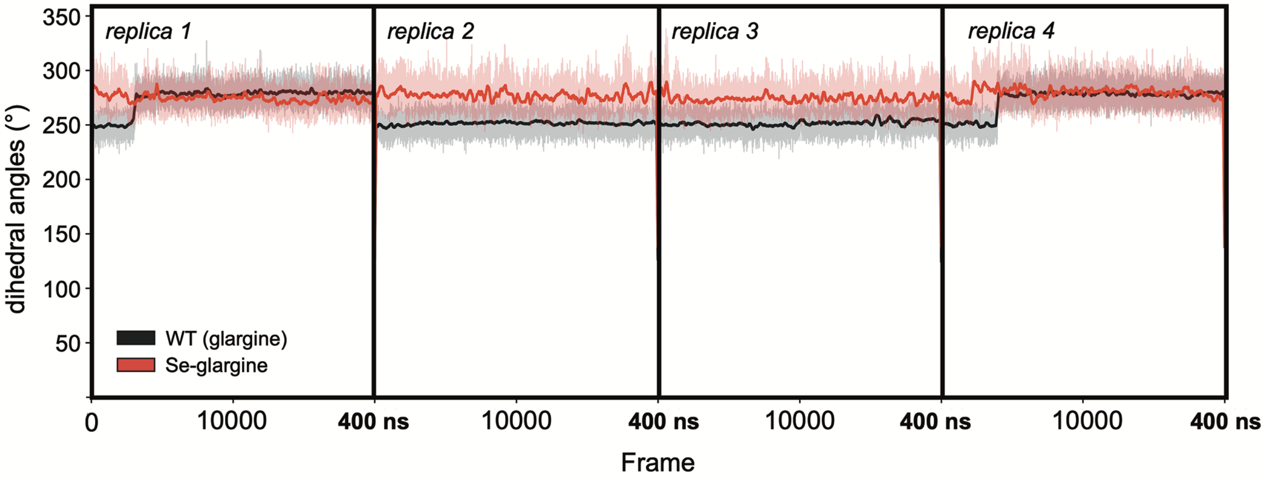

Corresponding timeline plot of the **A7–B7** χ_3 (Cβ–**S**–**S**–Cβ) dihedral of individual replicas (r_1-r_4) with WT vs. Se-glargine at A6-A11

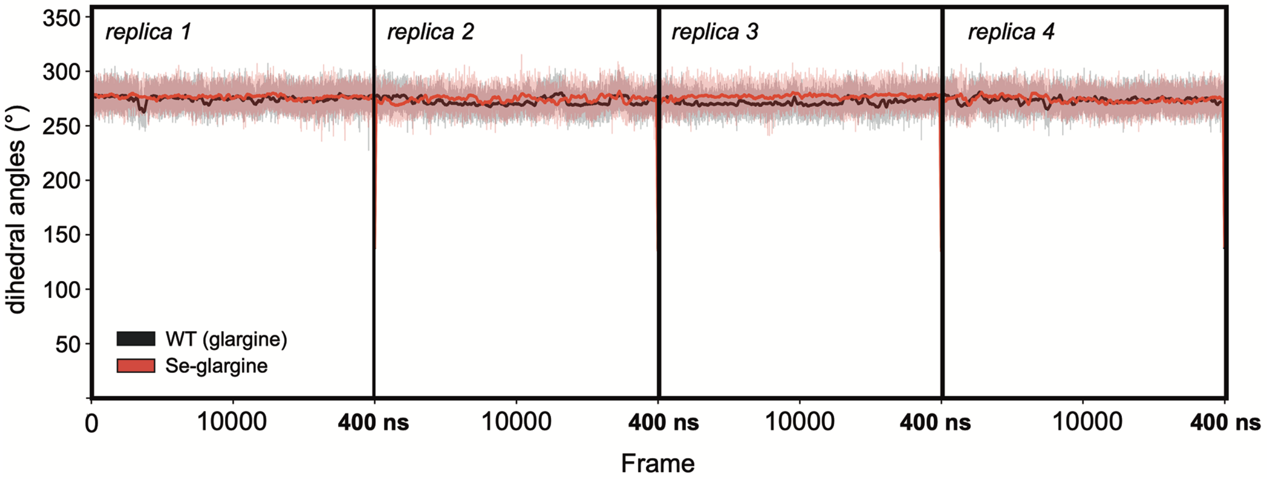

Corresponding timeline plot of the **A20–B19** χ_3 (Cβ–**S**–**S**–Cβ) dihedral of individual replicas (r_1-r_4) with WT vs. Se-glargine at A6-A11

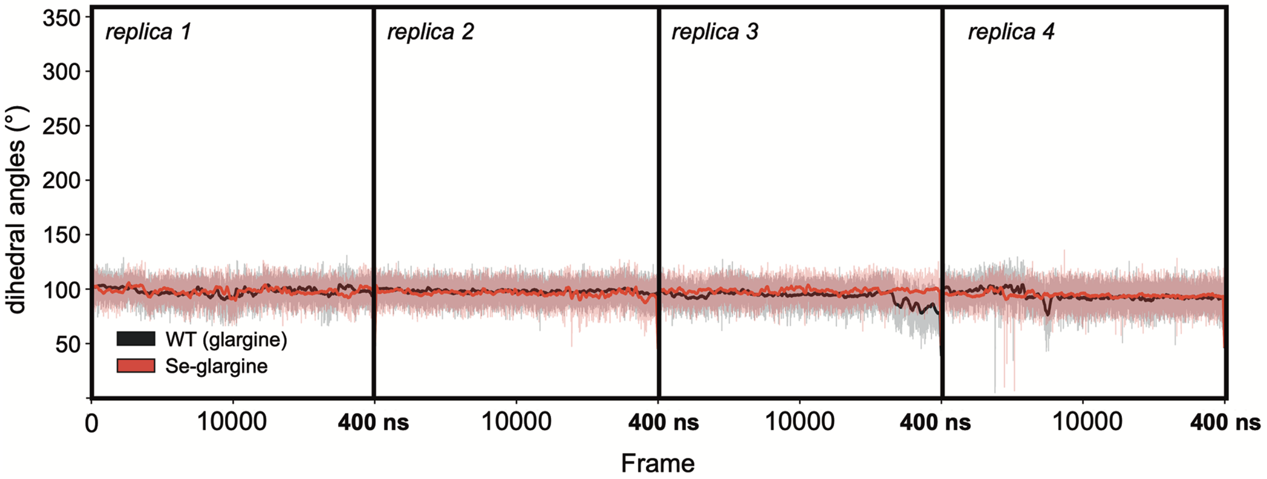

Figure S39. Time-series of side-chain torsions for the A6–A11 bridge comparing WT glargine (black) and Se-glargine (red). The A7-B7 and A20-B19 disulfide bridges were also monitored. Panels show example dihedral angles (χ_3) as labeled in the plots; units °. Four 400-ns replicas per system are concatenated left-to-right; vertical black lines mark replica boundaries. Faint traces are raw frame values; bold curves are running mean averages over 100 frames.

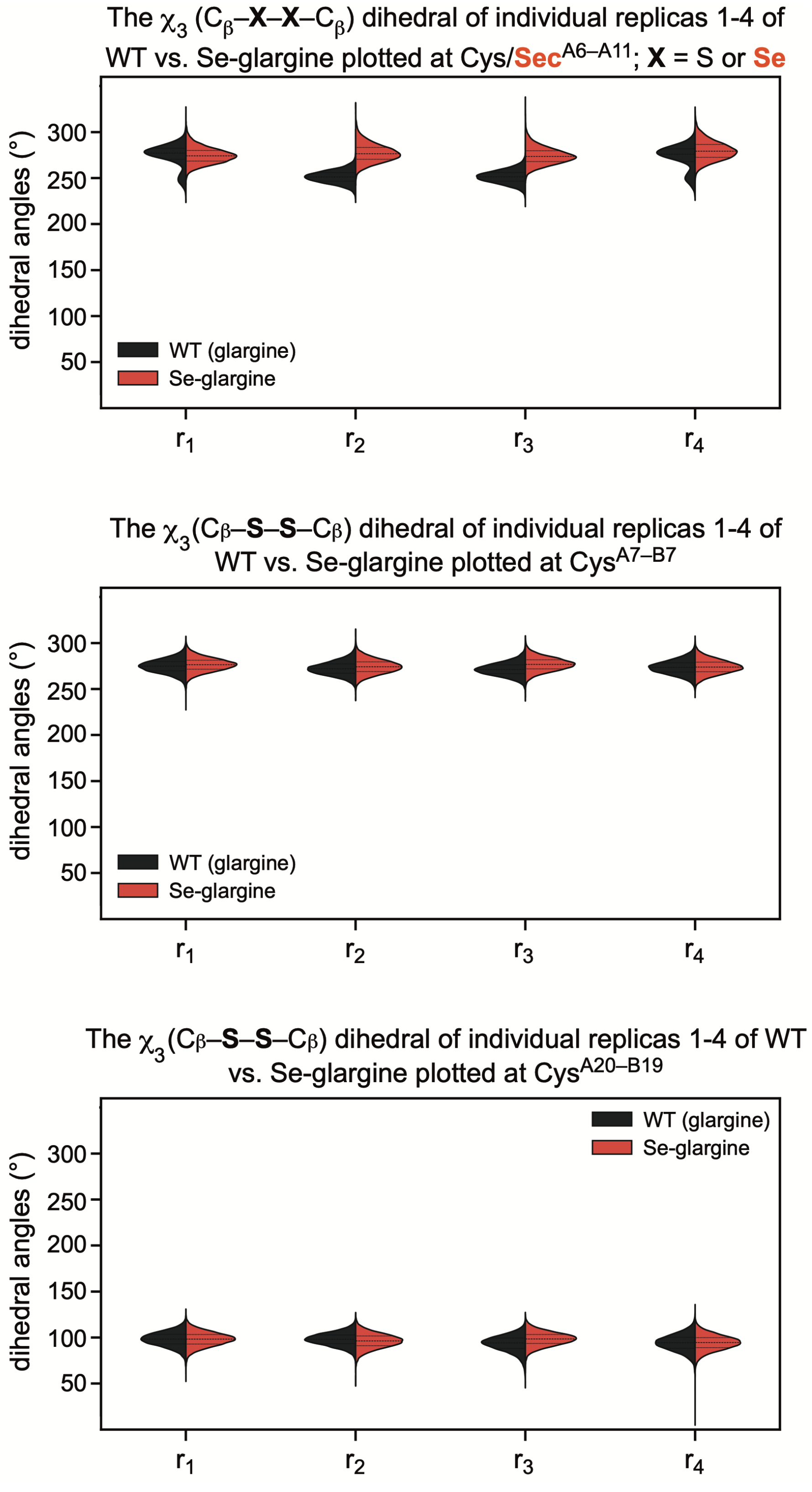

Figure S40. Distributions of disulfide/diselenide torsion angles (χ_3; C_β–X–X–C_β) from MD for glargine (WT, black) and Se-glargine (Sec^{A6}–Sec^{A11}) (red). Each panel shows split-violin plots per replica (r_1–r_4) for one bridge: Cys/$Sec^{A6–A11}$ (X = S in WT, Se in mutant), Cys^{A7}–Cys^{B7}, and Cys^{A20}–Cys^{B19} (X = S in both). Each violin summarizes all frames of the corresponding trajectory; *y*-axis is dihedral angle (degrees). Selenium substitution at A6–A11 shifts the χ_3 distribution relative to WT, while A7–B7 and A20–B19 remain similar across replicas.

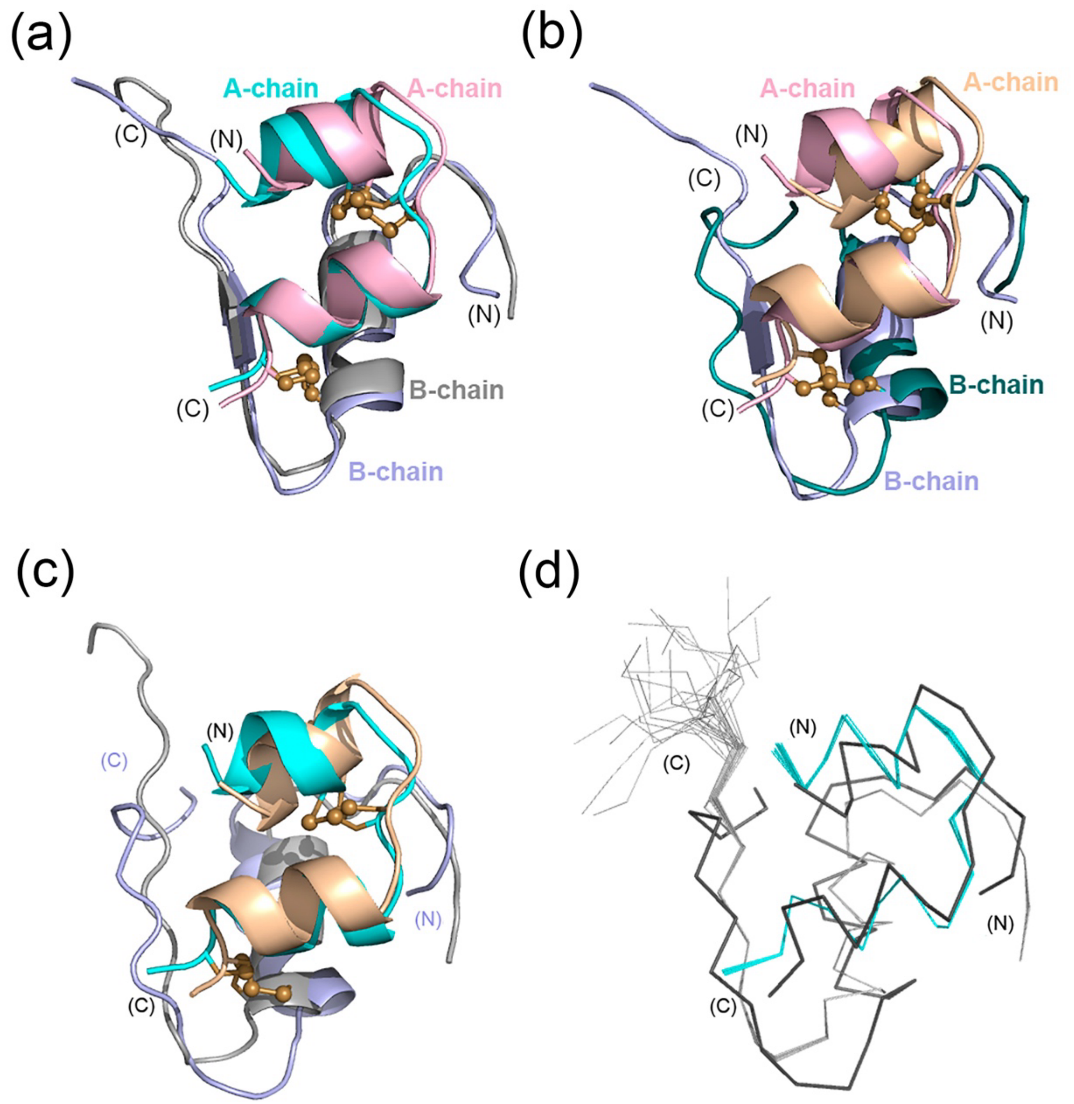

Figure S41. Structural comparisons of native insulin glargine: (a, b) crystal structure (PDB-ID: 4IYD) *versus* respective solution structures in 10% dAA (PDB-ID: 9ZO6 herein; a) or 20% dAA (PDB 6K59; b) as described ((Ratha et al., 2020); and (c, d) alignment of the two solution structures.

(a) Overlay of a representative solution structure in 10% dAA (present work) and a crystallographic protomer, as aligned in the region of A12-A19 and B9-B19. The RMSD is 0.6 Å. In the NMR-derived structure the A chain is shown in cyan, and the B chain in gray. In the crystal structure the A chain is shown in light pink, and the B chain in light blue. In each case disulfide bridges are shown in gold (spheres). (b) Analogous overlay of a representative solution structure in 20% dAA and the crystal structure. The RMSD is 1.8 Å. In the prior NMR-derived structure the A chain is shown in wheat, and the B chain in smudge. The crystal structure is colored as in panel (a).

(c) Overlay of respective solution structures in 10% and 20% dAA. The structures are aligned according to the main-chain atoms of the three helices (residues A2-A8, A13-A19 and B10-B19). In the present structure (10% dAA) the A chain is shown in cyan, and the B chain in gray. In the prior structure (20% dAA) the A chain is shown in wheat, and the B chain in light blue. The sulfur atoms in disulfide bridges are shown in gold (spheres). (d) Overlay of C_α traces of respective NMR-derived structures: thick black line represents the structure determined in 20% dAA (model 1 in PDB-ID: 6K59) and thin lines, the structure presently determined in 10% dAA C_α traces of 20 NMR structures. The color code is otherwise the same as in panel (c)

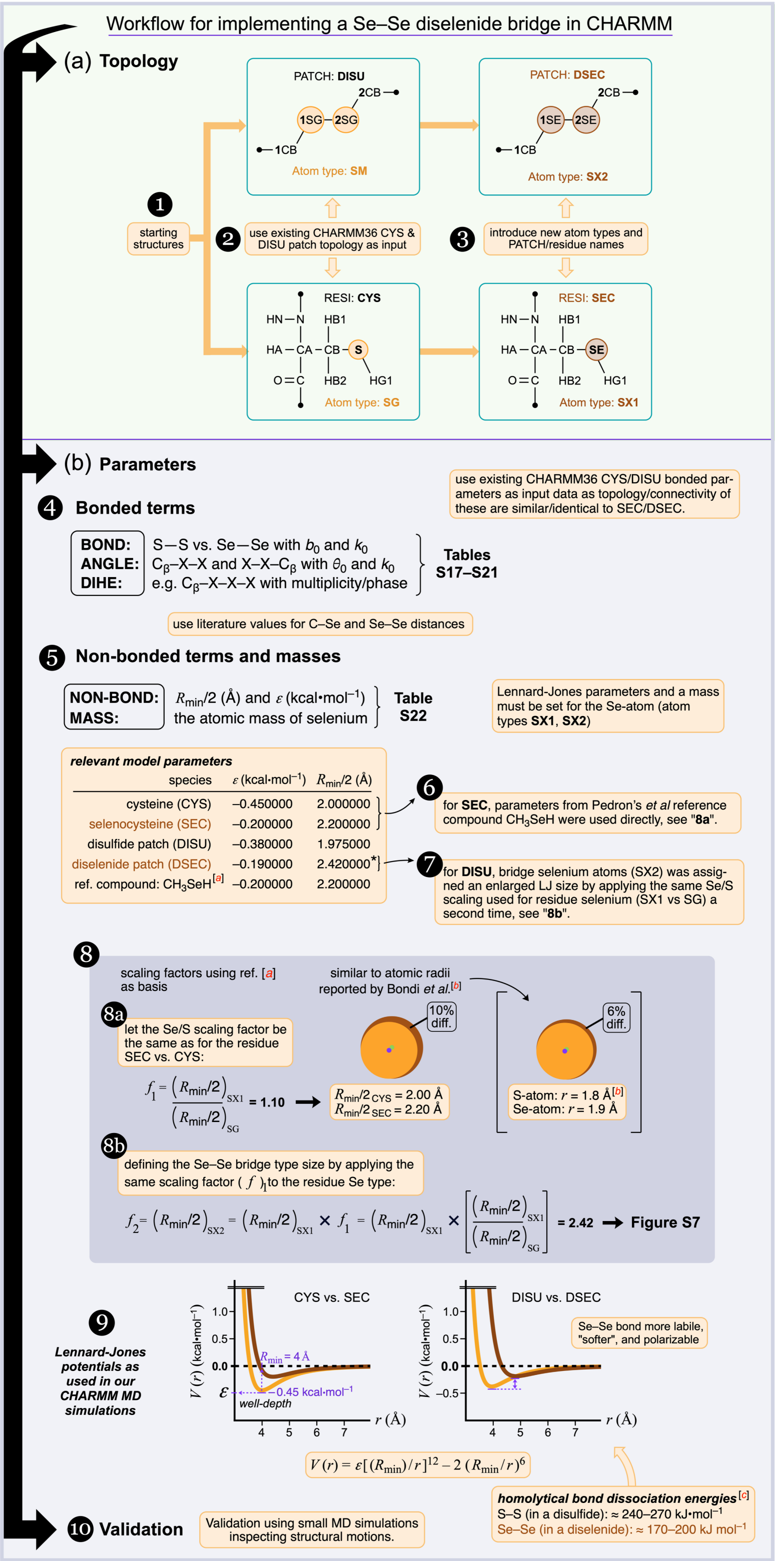

Figure S42. Workflow for implementing a Se–Se diselenide bridge in CHARMM (topology + parameters)

(a) **Topology**. Starting from the standard CHARMM36 disulfide model (**1**), input starting structures; RESI: CYS; PATCH: DISU), we use the existing CHARMM36 CYS residue and DISU patch topology/connectivity as the reference template (**2**). We then introduce the selenium analogs needed to build a diselenide (**3**): (i) a selenocysteine residue template (RESI: SEC) in which the side-chain chalcogen is renamed and retyped from S/SG to Se/SE (atom type SX1), and (ii) a diselenide patch (PATCH: DSEC) that mirrors DISU connectivity but forms an Se–Se bridge (atom type SX2). Separate CYS/SEC residue definitions are required because CHARMM constructs the bridge via a PATCH that removes the chalcogen H-atom (HG1) and connects the two side chains. The used bonded terms are collected in Tables S17–S21 for straightforward porting into a CHARMM-readable parameter file (e.g., *missing_params.prm* that can be easily read by CHARMM).

(b) **Parameters**. Bonded terms for the selenium-containing types (**4**; bonds/angles/dihedrals) are mapped from the corresponding CHARMM36 cysteine/disulfide terms because the topology is the same, and these are collected in Tables S17–S21 for direct porting into a CHARMM-readable parameter file. Non-bonded Lennard–Jones parameters and selenium mass (**5**) are then assigned for the Se atom types SX1 (residue Se) and SX2 (bridge Se) (Table S22). For SEC, non-bonded parameters are taken from a reference selenium compound (e.g., CH_3SeH) and used directly for SX1 (**6**), whereas for the diselenide bridge the LJ size is intentionally enlarged for SX2 to reflect the bulkier bridge environment (**7**). Partial charges were adjusted minimally relative to the sulfur and reference selenol/selenocysteine templates to preserve the overall neutral character of CYS/DISU and SEC/DSEC. Non-bonded sizes were generated using a two-step scaling (**8**): first setting the residue selenium size via scaling relative to cysteine sulfur (f_1, see panel **8a**) and then defining the bridge selenium by applying the same scaling again (f_2, see panel **8b**), yielding 2.42 Å (see also Figure S7). This "extra" scaling is intended to reflect the bulkier and more polarizable bridge environment. The same inset (**8**) also provides the conceptual comparison used for PyMOL visualizations, illustrating the slightly larger van der Waals radius/LJ contact distance for Se vs S and summarizing typical homolytic bond dissociation energy ranges (S–S ≈ 240–270 kJ·mol^{-1}; Se–Se ≈ 170–200 kJ·mol^{-1}; supporting the qualitative expectation that Se–Se is longer/weaker ("softer" or more polarizable) than S–S. Circle sizes illustrate the slightly larger van der Waals radius of selenium relative to sulfur ($r_{(S)}$ ≈ 1.80 Å; $r_{(Se)}$ ≈ 1.90 Å), with corresponding Lennard-Jones contact distances ($R_{min}/2$) of ≈ 2.00 Å for Cys sulfur and ≈ 2.20 Å for Sec selenium. Colored dots mark the centers from which these distances are measured. The LJ potentials used in simulations are illustrated for CYS vs. SEC and DISU vs. DSEC (**9**), and final parameters are validated by short MD tests and inspection of structural stability/motions (**10**). References: [*a*] (Pedron et al., 2023); [*b*] (Bondi, 1964); [*c*] (Kildahl, 1995; Sousa et al., 2019)

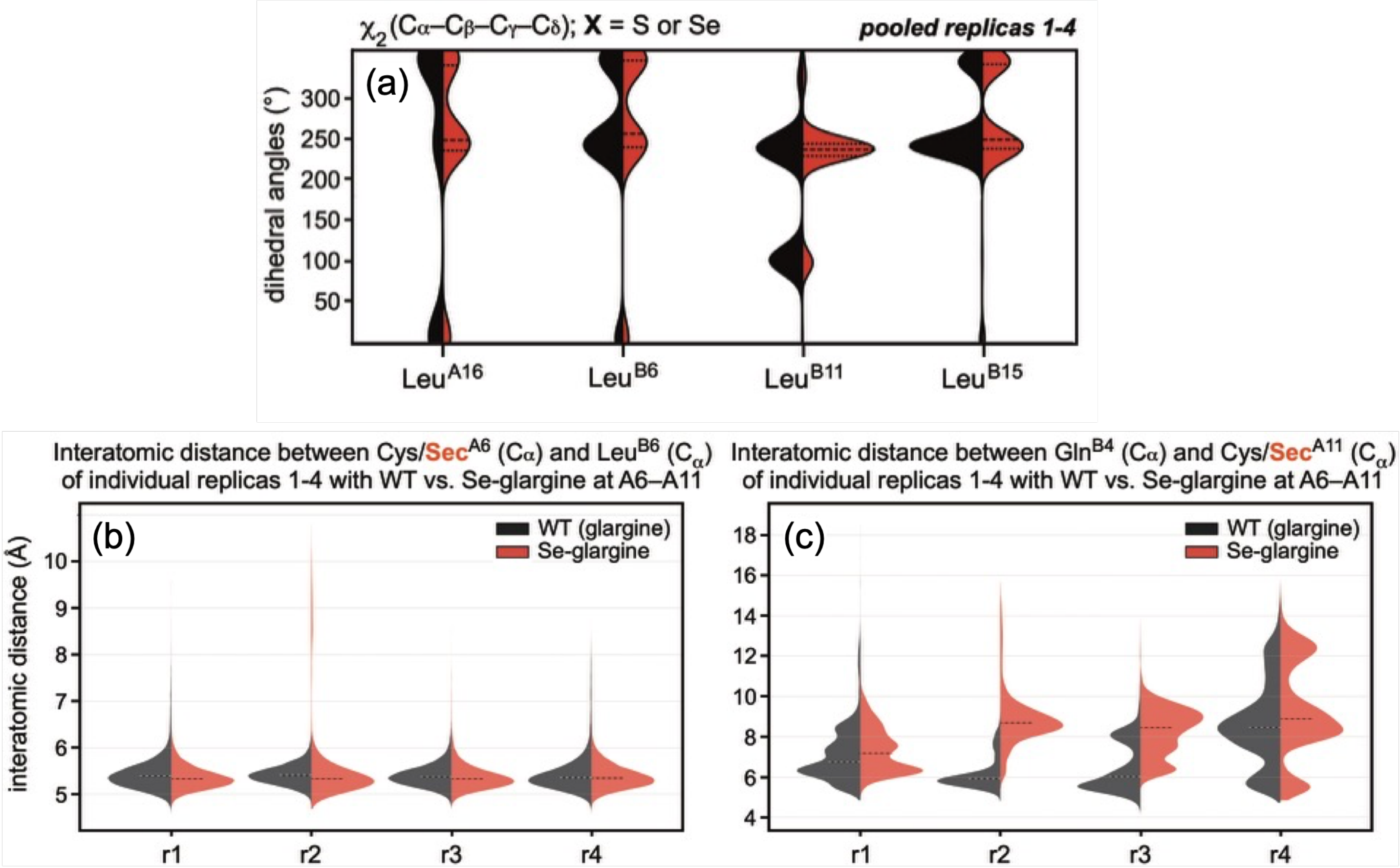

Figure S43. (a) Split violin plots of χ_2 (C_α–C_β–C_γ–C_δ) side-chain dihedral angles for leucine residues surrounding the A6–A11 bridge in WT glargine (black, left half of each violin) and Se-glargine (red, right half), pooled over replicas 1–4. Distributions for Leu^{A16}, Leu^{B6}, Leu^{B11}, and Leu^{B15} report whether rigidification of the A6–A11 diselenide propagates into neighboring hydrophobic side chains; only modest shifts and local narrowing are observed relative to WT. (b) Distributions of interatomic distances from MD simulations of WT glargine (Cys^{A6}–Cys^{A11}, black) and Se-glargine (Sec^{A6}–Sec^{A11}, red). $C_\alpha \cdots C_\alpha$ distance between Cys/Sec^{A6} and Leu^{B6}. (c) $C_\alpha \cdots C_\alpha$ distance between Gln^{B4} and Cys/Sec^{A11}. Each pair of violins corresponds to an individual replica (r_1–r_4). Se-glargine shows a modest compaction and narrowing of the B6–A6 distance distribution, but a broader and more heterogeneous B4–A11 distance distribution, indicating local stabilization near A6 and increased flexibility at the B4–A11 contact.

Table S1. Thermodynamic Stabilities (reproduced from prior publication for convenience of the reader from Weil-Ktorza et al., 2024).

Analog	ΔG_u[a] *kcal/mol*	*m*-value[b] *kcal/(mol•M)*	C_{mid}[c] *M*
human insulin	3.8 ± 0.1	0.76 ± 0.01	5.0 ± 0.1
insulin *glargine*	2.8 ± 0.1	0.57 ± 0.01	4.9 ± 0.1
Se-glargine	3.8 ± 0.1	0.63 ± 0.01	6.0 ± 0.1

[a]Data are from two-state modeling of CD-guanidine titrations performed at 25 °C and pH 4.

[b]The *m*-value (with units kcal/(mol•M) is the slope of unfolding free energy ΔG_U versus molar concentration of denaturant.

[c]C_{mid} is the guanidine denaturant concentration at which 50% of the protein is in the unfolded state.

Table S2. Deconvolution of secondary structures from CD spectra.[a]

analog	Method	α_R	α_D	β_R	β_D	T	U	α-helix (%)	β-sheet (%)	Disordered (%)	Turn (%)
human insulin	SELCON3	0.294	0.213	0.093	0.021	0.137	0.242	50.7	11.4	13.7	24.2
	CONTINLL	0.303	0.254	0.145	0.018	0.107	0.174	55.7	16.3	10.7	17.4
	CDSSTR	0.330	0.252	0.065	0.046	0.154	0.159	58.2	11.1	15.4	15.9
glargine	SELCON3	0.277	0.199	0.094	0.025	0.141	0.255	47.6	11.9	14.1	25.5
	CONTINLL	0.279	0.249	0.169	0.022	0.098	0.183	52.8	19.1	9.8	18.3
	CDSSTR	0.314	0.244	0.085	0.069	0.145	0.144	55.8	15.4	14.5	14.4
Se-glargine	SELCON3	0.267	0.207	0.099	0.034	0.133	0.22	47.4	13.3	13.3	22
	CONTINLL	0.358	0.27	0.136	0.000	0.041	0.197	62.8	13.6	4.1	19.7
	CDSSTR	0.284	0.268	0.055	0.043	0.140	0.210	55.2	9.8	14.0	21

[a]The secondary structures are: α_R, regular helix; α_D, distorted helix; β_R, regular strand; β_D, distorted strand; T, turns; and U, unordered. Protein CD analysis was done using CDPro software package (https://sites.google.com/view/sreerama). Reference dataset used was SP43 (soluble protein 43).

Table S3. Backbone chemical shift perturbation induced by A6-A11 diselenide bridge in Se-glargine. The ^1H_N/$^1H_\alpha$ chemical shift difference bigger than 0.05 ppm and ^{15}N/$^{13}C_\alpha$ chemical shift difference bigger than 0.30 ppm are listed in red. NMR data were acquired in 10% deuterated acetic acid at pH 2.1 and 25 °C.

A domain							
	$^{15}N/^1H_N$ (ppm)[a]		$\Delta_{N/HN}$ (Hz)[b]	$^{13}C\alpha/^1H\alpha$ (ppm)[a]		$\Delta_{C\alpha/H\alpha}$(Hz)[c]	$\Delta_{backbone}$ (Hz)[d]
residue	Se-glargine	glargine		Se-glargine	glargine		
I2	122.25/8.71	121.27/8.56	123.54	64.33/3.782	62.16/3.99	410.65	428.83
V3	122.93/8.16	123.23/8.17	22.013	66.04/3.571	64.92/3.68	212.02	213.16
E4	121.42/8.20	121.52/8.18	17.46	58.70/4.200	57.63/4.27	195.27	196.05
Q5	NA/8.16	NA/8.32	112.05	58.73/4.026	58.77/4.09	48.83	122.23
C6	111.53/8.28	110.95/8.37	73.33	55.91/4.759	54.12/4.97	347.63	355.28
C7	117.87/8.35	118.45/8.28	64.86	57.12/4.877	57.09/4.87	5.67	65.11
T8	115.58/8.41	NA/8.30	77.04	65.32/4.030	65.21/4.04	20.68	79.77
S9	NA/7.28	NA/7.47	133.06	55.82/4.782	55.95/4.82	35.19	137.63
I10	116.74/7.79	117.38/7.88	78.504	59.52/4.518	59.98/4.36	139.61	160.17
C11	126.20/9.75	NA/9.82	44.12	53.29/5.251	53.96/5.00	213.27	217.79
S12	117.188/8.80	117.25/8.75	32.54	56.48/4.58	56.39/4.62	28.12	43.01
L13	121.43/8.69	121.78/8.65	34.66	58.15/3.88	58.36/3.82	55.57	65.49
L16	121.50/8.26	120.05/8.14	131.04	58.46/4.18	58.14/4.20	57.60	143.14
E17	113.39/8.08	113.93/8.11	42.58	57.54/4.26	57.98/4.26	78.82	89.59
Y19	116.65/8.03	117.02/8.00	32.30	59.12/4.47	58.95/4.48	29.94	44.04
C20	115.92/7.42	115.67/7.42	18.04	53.40/5.06	53.21/5.12	53.06	56.04
B domain							
	$^{15}N/^1H_N$ (ppm)		$\Delta_{N/HN}$ (Hz)[b]	$^{13}C\alpha/^1H\alpha$ (ppm)		$\Delta_{C\alpha/H\alpha}$(Hz)[c]	$\Delta_{backbone}$ (Hz)[d]
residue	Se-glargine	glargine		Se-glargine	glargine		
V2	123.99/8.21	124.27/8.12	50.90	61.59/4.13	61.51/4.13	14.86	53.02
Q4	53.78/4.51	54.05/4.54	16.33	53.78/4.51	54.05/4.53	50.75	53.31
L6	127.28/9.17	126.90/9.07	72.36	54.57/4.53	54.74/4.53	29.79	78.25
C7	118.38/8.36	118.95/8.42	59.20	53.51/5.07	53.91/5.04	73.51	94.38
G8	112.26/9.55	NA/9.39	112.75	46.57/3.86	46.59/3.86	6.04	112.91
L11	121.89/7.04	121.40/7.09	50.15	57.61/4.02	57.74/4.05	29.15	58.01
E13	116.70/7.96	116.62/7.91	41.71	58.53/4.14	58.56/4.14	6.37	42.19
A14	121.12/7.76	121.01/7.70	40.59	55.24/4.13	55.18/4.14	14.90	43.24
L17	119.13/7.97	119.16/7.82	99.47	57.62/4.08	57.51/4.10	25.74	102.75
V18	116.50/8.54	117.01/8.53	37.61	64.99/3.91	65.13/3.86	43.41	57.44
F24	116.16/7.71	115.88/7.68	29.16	56.85/4.99	56.77/5.07	51.98	59.60

[a]All chemical shifts were calibrated in parts per million (ppm) relative to 4,4-dimethyl-4-silapentane-1-sulfonic acid (DSS) as an internal standard, which was set to 0 ppm.

[b]Combined $^{15}N/^1H_N$ chemical shift perturbation was calculated according to the equation $\Delta_{H/C} = \sqrt{\delta_{HN}^2 + \delta_N^2}$. δ_{HN} and δ_N are the observed ^1H_N and ^{15}N chemical shift differences between Se-glargine and glargine, respectively.

[c]Combined $^{13}C\alpha/^1H\alpha$ chemical shift perturbation was calculated according to equation $\Delta_{H/C} = \sqrt{\delta_{H\alpha}^2 + \delta_{C\alpha}^2}$. $\delta_{H\alpha}$ and $\delta_{C\alpha}$ are the observed $^1H\alpha$ and $^{13}C\alpha$ chemical shift differences between Se-glargine and glargine, respectively.

[d]Combined backbone chemical shift perturbation was calculated according to the equation $\Delta_{H/C} = \sqrt{\delta_{N/HN}^2 + \delta_{C\alpha/H\alpha}^2}$. $\Delta_{H/HN}$ and $\Delta_{C\alpha/H\alpha}$ are the combined $^{15}N/^1H_N$ and $^{13}C\alpha/^1H\alpha$ chemical shift differences between Se-glargine and glargine, respectively.

Table S4. NOE lists that were only observed in Se-glargine or in glargine (*red*) and that probably resulted in chemical shift degeneracy (*black*).

residue	Se-glargine	Glargine
	A chain	
A2	A2Hα-B11Hδ_1, A2Hγ_2-B27HN, A2Hγ_2-B28Hα	A2Hγ_2-B15Hδ_1
A3	A3Hγ_2-A4HN, A3Hγ_2-B29HN, A3Hα-A7Hβ_2, A3Hα-A8HN, A3Hα--B7Hα, A3Hα-B26Hε_1	
A5	A5Hα-A7HN, A5Hβ_1-A7HN, A5Hβ_2-A7HN, A5HN-A7HN	A5Hα-A8HN
A7	A7Hα-A8Hα, A7HN-A8Hα, A7HN-A8Hγ_2	A7Hα-B6Hβ_2
A10	A10Hγ_{11}-A11HN, A10Hγ_{12}-A11HN, A10Hγ_2-A12HN, A10Hγ_2-A26HN	
A11	A11HN-A12HN, A11Hβ_1-A15Hβ_1, A11Hβ_1-A15Hβ_2, A11HN-A15Hβ_2 A11Hβ_1-A16Hδ_2, A11Hβ_2-A16Hδ_2, A11Hβ_1-B6Hδ_1, A11Hβ_1-B11Hδ_1	
A12	A12HN-A15Hγ_2	
A16	A16Hδ_1-A17HN, A16Hδ_2-A17HN, A16HN-A17HN, A16Hα-A18Hβ_2 A16HN-A18HN, A16HN-A19Hε_2, A16Hβ_1-B15Hα	A16Hδ_1-B15Hα
A17	A17HN-B18Hγ_1	
A20	A20Hα-A21Hα_2, A20Hβ_2-B22Hβ#	
A21	A21Hα_2-B24Hα	A21HN-B22Hβ#
	B chain	
B2	B2Hγ_2-B4Hε_{21}, B2Hγ_2-B4Hε_{22}	
B4		B4HN-B6Hδ_1
B5	B5Hδ_2-B6Hδ_1	
B6	B6Hδ_2-B11Hα	
B7	B7Hα-B11HN	
B9	B9Hα-B11HN	
B10		B10Hα-B11HN
B11	B11Hβ_2-B15Hδ_1, B11Hδ_1-B15HN	
B12	B12Hγ_1-B14HN	B12Hβ-B24Hε_1
B14	B14HN-B15Hγ, B14Hβ-B17Hβ_1	B14HN-B17Hδ_1
B15	B15Hγ-B16HN, B15Hα-B18Hβ, B15Hα-B18Hγ_2, B15Hγ_2-B19HN B15Hγ_2-B24Hα	
B16	B16Hε_2-B17Hα, B16HN-B17Hβ_2	
B17		B17Hα-B20HN
B19	B19HN-B24Hβ_1	
B22	B22Hα-B23Hα_2	
B23	B23HN-B24Hδ_2	
B26	B26HN-B28Hγ_2	

Table S5. Statistics of experimental data and structure calculations of glargine.

Parameter	SA ensemble[a]
RMSD from experimental distance restraints (Å)	
all (1237)	0.036 ± 0.008
intraresidue, i=j (440)	0.039 ± 0.008
sequential, |i-j|=1 (285)	0.036 ± 0.008
medium range, 1<|i-j|<5 (248)	0.034 ± 0.007
long range, |i-j|≥5 (264)	0.033 ± 0.009
Rmsd from idealized covalent geometry	
bonds (Å)	0.0068± 0.0015
angles (°)	0.96 ± 0.22
impropers (°)	2.26 ± 0.51
$E_{L\text{-}J}$ (kcal/mol) [b]	–190.7 ± 42.9
Ramachandran plot [c]	
most favored regions (%)	82.1
additionally and generously allowed regions (%)	17.5
disallowed regions (%)	0.4
Coordinate precision of native insulin-like region [d]	
RMSD of backbone atoms to the mean (Å)	0.34 ± 0.13
RMSD of all heavy atoms to the mean (Å)	0.65 ± 0.15

[a]Mean ± standard error where applicable.
[b]Lennard-Jones potential energy function, calculated with CHARMM19 (Brooks et al., 2009) empirical energy parameters.
[c]Residues 1-21 (A1-A21), 24-49 (B3-B28) for native insulin region. Glycine residues were not included.
[d]Calculated using the Molmol program (Koradi et al., 1996) for residues 1-21 (A1-A21), 24-49 (B3-B28).

Table S6. Statistics of experimental data and structure calculations of Se-glargine.

Parameter	SA ensemble[a]
Rmsd from experimental distance restraints (Å)	
all (1284)	0.035 ± 0.008
intraresidue, i=j (438)	0.038 ± 0.009
sequential, |i-j|=1 (296)	0.033 ± 0.008
medium range, 1<|i-j|<5 (290)	0.036 ± 0.008
long range, |i-j|≥5 (260)	0.031 ± 0.007
Rmsd from idealized covalent geometry	
bonds (Å)	0.0077± 0.0017
angles (°)	1.04 ± 0.23
impropers (°)	2.29 ± 0.51
E_{L-J} (kcal/mol) [b]	–192.1 ± 43.6
Ramachandran plot [c]	
most favored regions (%)	81.8
additionally and generously allowed regions (%)	18.2
disallowed regions (%)	0.0
Coordinate precision of native insulin-like region [d]	
rmsd of backbone atoms to the mean (Å)	0.35 ± 0.11
rmsd of all heavy atoms to the mean (Å)	0.77 ± 0.11

[a]Mean ± standard error where applicable.
[b]Lennard-Jones potential energy function, calculated with CHARMM19 (Brooks et al., 2009) empirical energy parameters.
[c]Residues 1-21 (A1-A21), 24-49 (B3-B28) for native insulin region. Glycine residues were not included.
[d]Calculated using Molmol program (Koradi et al., 1996) for residues 1-21 (A1-A21), 24-49 (B3-B28).

Table S7. NOE cross-checking violations using the NOE distance restraints of Se-glargine to check 20 NMR structures of glargine and that using the NOE distance restraints of glargine to check 20 NMR structures of Se-glargine. The # indicated the chemical shift degeneracy. The number in the brackets was the structure number with NOE violation. The threshold for NOE violation was set up as 0.5 Å.

Se-glargine NOEs for 20 glargine structures	Glargine NOEs for 20 Se-glargine structures
A2Hγ_{12}-B26Hβ# (20), A3HN-A4Hβ# (20), A10HN-A11HN (20), A10Hβ-B5Hε_1 (20), A10Hγ_{11}-B5Hε_1 (20), A10Hγ_{11}-B5Hα (20), A10Hγ_2-B5Hε_1 (20), A10Hδ_1-B5Hα (20), A10Hδ_1-B5Hε_1 (20), A13Hδ_2-A14Hε_1 (20), A13Hα-A16Hβ_1 (20), A13Hα-A16Hδ_2 (20), A13HN-A16Hδ_2 (20), B1Hζ-B6Hγ (20), B9Hα-B10Hδ_2 (20), B9Hβ-B13Hγ# (20), B14Hβ-B17Hδ_1 (20), B25Hβ_1-B27Hγ_2 (20)	A2Hδ_1-A3HN (20), A2Hδ_1-A16Hα (20), A3Hγ_1-A4Hγ_1 (20), A4Hβ_2-A6HN (20), A4Hβ_2-A8Hγ_2 (20), A9Hα-B5Hδ_2 (20), A10Hγ_2-A11Hα (20), A10Hγ_2-A11HN (20), A13Hβ#-A14Hδ_1 (20), A13Hγ-A14Hε_1 (20), A16Hδ_1-A17HN (20), A16Hγ-B11Hδ_1 (20), A16Hβ_2-B14Hβ (20), A18Hβ_1-A19Hε_2 (20),), B4Hβ_1-B6Hγ (20), B5Hδ_2 -B6Hδ_1 (20), B6Hδ_2–B10Hδ_2 (20), B12Hα-B16Hδ_1 (20), B12Hγ_1-B16Hδ_1 (20), B12Hγ_2-B16Hβ# (20), B13HN-B16Hβ# (20), B15Hδ_2–B24Hα (20),
A15HN-A16Hβ_1 (19), B9Hα-B13Hγ# (19)	A10Hγ_2-B5Hδ_2 (19), A11Hβ_2-A15Hγ_1 (19)
	B12Hγ_2-B24Hζ (18),
	A2Hδ_1-A19Hε_2 (17), A13Hδ_1-A13HN (17)
A20Hβ_2-A21HN (16), B13Hβ_2-B16Hε_2 (16)	
A13Hδ_2-A14HN (15)	A15Hγ_1-A15Hε_{22} (15),
A12HN-A14HN (14), B10Hδ_2-B13Hγ# (14)	A2Hα-A4Hβ_2 (14)
A19Hδ_1-B26Hα (13), B2Hγ_2-B4Hγ_2 (13)	A8Hβ_2-A9Hβ_2 (13),), A10Hγ_2-A12HN (13), B4HN-B5Hδ_2 (13),
A5Hγ_1-A6Hα (12)	A6Hα-A9Hβ_1 (12)), B15Hβ_2-B26Hε_1 (12), B27HN-B29HN (12)
A1Hα#-A4HN (11)	
A7Hα-B5Hβ_2 (10), A16Hδ_2-A16HN (10)	B29Hα-B31HN (10)
A5Hγ_1-A19Hε_2 (9), B9HN-B10Hδ_2 (9)	
A15Hγ_1-A16HN (8), B29Hγ_2-B29HN (8)	A11Hβ_1-A15Hγ_2 (8), B4Hβ_1-B5Hδ_2 (8), B19Hα-B22Hδ# (8)
A2Hα-A5Hβ_1 (7), B2HN-B3Hδ_{21} (7)	A11Hβ_2-A16Hδ_1 (7), B19Hα-B22Hε (7)
B21Hγ#-B21HN (6)	
B22Hγ_2-B22HN (5), B29Hγ_1-B29HN (5), A4Hα-A4Hγ_2 (5)	A11Hβ_2-A15Hβ_2 (5), B20HN-B24Hβ_1 (5),
A16Hβ_1-B18Hα (4),	B29Hα-B29Hδ# (4)
B12Hβ-B24Hζ (3), B19Hγ_2-B22Hγ_1 (3), B15Hδ_2-B18Hγ_2 (3), B26Hβ#-B27Hγ_2 (3), B27Hα-B27Hγ_2 (3), B27Hγ_2-B28Hδ# (3), B31Hα-B32Hβ_1 (3)	A3Hγ_1-A4Hα (3), A3Hγ_2-B28Hδ# (3),
A13Hδ_1-A17Hγ_2 (2)	A3Hγ_2-B28Hβ_2 (2), A6Hα-A9Hβ_2 (2)
A5Hα-A8HN (1), A12Hβ_2-A14HN (1), A16Hγ-B18Hγ_2 (1), A17Hα-A19HN (1), A18Hβ_2-A18Hδ_{22} (1), B1Hε_1-B14Hβ (1), B3Hα-B4Hγ_2 (1), B4Hα-B4Hγ_1 (1), B4Hγ_2-B4HN (1), B4Hγ_1-B5HN (1), B4Hγ_2-B6Hδ_1 (1), B16Hβ#-B17Hδ_1 (1), B22Hα-B22Hγ_2 (1), B22Hγ_2-B23HN (1), B26Hε_2-B28Hβ_1 (1)	A2Hγ_{11}-A6Hβ_2 (1), B12Hγ_1-B16Hβ# (1), B12Hγ_2-B24Hδ_1 (1), B15Hδ_2-B24Hδ_1 (1), B15Hδ_1-B26Hε_1 (1)

Table S8. Side-chain chemical shift perturbation greater than 40 Hz induced by A6-A11 diselenide bond. NMR data were acquired in 10% deuterated acetic acid at pH 2.1 and 25 °C.

group	Se-glargine ($^{13}C/^{1}H$, ppm)[a]	glargine ($^{13}C/^{1}H$, ppm)[a]	$\Delta_{C/H}$ (Hz)[b]
A-domain			
I2 δ_1-CH_3	14.43/0.49	14.06/0.62	108.97
I2 $C_{\gamma1}$-$H_{\gamma11}$	28.63/1.00	27.89/1.21	195.87
I2 $C_{\gamma1}$-$H_{\gamma12}$	28.63/0.871	27.89/0.97	146.81
I2 γ_2- CH_3	15.97/0.76	16.73/0.80	137.13
V3 γ_1- CH_3	21.22/0.92	20.98/0.92	41.42
E4 C_β-$H_{\beta1}$	29.58/2.18	29.05/2.13	101.27
E4 C_β-$H_{\beta2}$	29.58/2.12	29.05/2.13	94.54
Q5 C_β-$H_{\beta1}$	29.69/2.09	29.11/2.08	102.17
Q5 C_β-$H_{\beta2}$	29.69/2.15	29.11/2.16	102.17
Q5 C_γ-$H_{\gamma1}$	34.22/2.44	33.80/2.42	75.42
Q5 C_γ-$H_{\gamma2}$	34.22/2.55	33.80/2.51	80.96
Q5 $N_{\varepsilon2}$-$H_{\varepsilon21}$[c]	112.13/7.66	111.53/7.52	101.55
Q5 $N_{\varepsilon2}$-$H_{\varepsilon22}$[c]	112.13/6.98	111.53/6.92	42.02
C6 C_β-$H_{\beta2}$	NA/3.12	NA/2.88	172.98
C7 C_β-$H_{\beta1}$	38.38/3.41	38.69/3.34	73.90
C7 C_β-$H_{\beta2}$	38.38/3.89	38.69/3.82	70.74
T8 C_β-H_β	68.13/4.44	68.44/4.41	59.18
I10 C_β-H_β	39.58/1.51	39.04/1.59	110.13
I10 δ_1-CH_3	12.71/0.50	12.36/0.51	63.06
I10 $C_{\gamma1}$-$H_{\gamma11}$	26.96/1.01	26.96/1.09	51.14
I10 $C_{\gamma1}$-$H_{\gamma12}$	26.96/0.30	26.96/0.42	84.05
C11 C_β-$H_{\beta1}$	NA/3.74	NA/3.24	347.36
L13 C_β-$H_{\beta2}$	41.16/1.36	41.13/1.26	68.19
Q15 C_β-$H_{\beta1}$	29.27/1.99	29.02/2.06	62.46
Q15 C_β-$H_{\beta2}$	29.27/2.45	29.02/2.41	51.74
Q15 $N_{\varepsilon2}$-$H_{\varepsilon21}$[c]	111.86/7.03	111.57/6.97	41.26
L16 C_β-$H_{\beta2}$	41.98/1.81	41.96/1.58	161.11
L16 δ_2-CH_3	26.09/0.81	25.25/0.81	147.97
L16 C_γ-H_γ	26.83/1.70	26.95/1.76	50.014
Y19 C_ε-H_ε	117.70/6.76	117.93/6.78	41.63
B-domain			
L6 δ_1-CH_3	26.046/0.900	26.332/0.924	53.10
C7 C_β-$H_{\beta1}$	47.863/3.248	47.586/3.272	51.60
C7 C_β-$H_{\beta2}$	47.863/2.939	47.586/2.973	54.29
L11 δ_1-CH_3	22.100/0.752	22.337/0.746	41.95
V12 γ_1- CH_3	22.515/0.998	22.229/1.004	50.54
L15 C_β-$H_{\beta1}$	41.023/0.841	40.580/0.747	102.08
L15 C_β-$H_{\beta2}$	41.023/1.310	40.580/1.257	86.40

[a]All chemical shifts were calibrated in parts per million (ppm) relative to 4,4-dimethyl-4-silapentane-1-sulfonic acid (DSS) as an internal standard, which was set to 0 ppm.
[b]Combined $^{13}C/^{1}H$ (or $^{15}N/^{1}H_N$) chemical shift perturbation according to the equation $\Delta_{H/C} = \sqrt{\delta_H^2 + \delta_C^2}$. δ_H and δ_C are the observed ^1H and ^{13}C chemical shift differences between Se-glargine and glargine, respectively.
[c]Side-chain amide chemical shifts of glutamine residue.

Table S9. Monomer-dimer exchange rate of glargine and Se-glargine measured by 2D exchange spectra in 10-mM deuterated acetic acid at pH 3.0 (direct meter reading) and 25 °C.

exchange peak	Exchange rate (ms^{-1})	
	Glargine	Se-glargine
I2MHN-I2DHN	0.058	0.047
V3MHN-V3DHN	0.067	0.043
V3DHN-V3MHN	0.073	0.038
T8MHN-T8DHN	0.088	0.079
T8DHN-T8MHN	0.087	0.071
Y19MHN-Y19DHN	0.120	0.076
Y19DHN-Y19MHN	0.099	0.078
G29MHN-G29DHN	0.119	0.054
S30MHN-S30DHN	0.068	0.033
S30DHN-S30MHN	0.065	0.034
H31MHN-H31DHN	0.075	0.066
V33MHN-V33DHN	0.084	0.065
E34DHN-E34MHN	0.076	0.068
A35MHN-A35DHN	0.084	0.050
A35DHN-A35MHN	0.048	0.048
L36MHN-L36DHN	0.072	0.037
L36DHN-L36MHN	0.076	0.046
Y37MHN-Y37DHN	0.065	0.038
Y37DHN-Y37MHN	0.058	0.045
V39MHN-V39DHN	0.088	0.051
V39DHN-V39MHN	0.103	0.077
C40MHN-C40DHN	0.099	0.068
G41MHN-G41DHN	0.079	0.034
E42MHN-E42DHN	0.067	0.039
E42DHN-E42MHN	0.052	0.038
F45DHN-F45MHN	0.059	0.039
Y47MHN-Y47DHN	0.074	0.049
Y47DHN-Y47MHN	0.092	0.056
T48MHN-T48DHN	0.058	0.039
T51MHN-T51DHN	0.097	0.049
T51DHN-T51MHN	0.091	0.061
R52MHN-R52DHN	0.101	0.046
R52DHN-R52MHN	0.115	0.054
	0.079±0.020	0.052±0.014
global fitting	0.082±0.012	0.057±0.013
lifetime	~12.3±2.6 ms	~17.5±3.8 ms

Table S10. The protein monomer-dimer ratios and dimerization constants of Se-glargine and glargine measured by ^{1}H, ^{13}C-HSQC spectra in different protein concentration. Data were obtained at a ^{1}H frequency of 700 MHz in 10mM deuterated acetic acid (pH 3.0, direct meter reading) at 25 °C.

Glargine		
concentration (μM)	monomer ratio (%)	K_d (μM)
41	40.7	22.9
54	35.7	21.4
88	24.9	14.5
142	19.6	13.6
156	18.6	13.2
273	14.6	13.6
K_d = 16.5(±4.4) μM		
Se-glargine		
concentration (μM)	monomer ratio (%)	K_d (μM)
76	26.8	14.9
60	28.1	13.2
K_d = 14.1(±1.2) μM		

Table S11. Main-chain ^{15}N, ^{1}H$_N$, ^{13}Cα, ^{1}Hα chemical shifts, methyl ^{13}C and ^{1}H chemical shifts[a] and corresponding secondary chemical shifts of Ile^{A2} in Se-glargine and glargine in 10% deuterio-acetic acid at pH 2.1 (direct meter reading) and at 25 °C.

main chain[b]								
Ile^{A2}	^{13}C$_\alpha$	2nd C$_\alpha$	^{1}H$_\alpha$	2nd H$_\alpha$	^{15}N	2nd N	^{1}H$_N$	2nd H$_N$
Se-glargine	64.335	3.235	3.782	-0.388	122.248	1.178	8.710	0.770
Glargine	62.158	1.058	3.992	-0.178	121.271	0.201	8.564	0.624

side chain[c]								
Ile^{A2}	γ2-CH3				δ1-CH3			
	^{13}C	2nd C	^{1}H	2nd H	^{13}C	2nd C	^{1}H	2nd H
Se-glargine	15.966	-1.434	0.759	-0.151	14.425	1.525	0.494	-0.366
Glargine	16.729	-0.67	0.798	-0.112	14.062	1.162	0.620	-0.240

[a]Proton chemical shifts were calibrated in parts per million (ppm) relative to 4,4-dimethyl-4-silapentane-1-sulfonic acid (DSS) as an internal standard, which was set to 0 ppm.

[b]Main chain secondary shift is defined as the differences between observed chemical shift and random-coiled chemical shift (Wang and Jardetzky, 2002).

[b]Side chain secondary shift is defined as the differences between observed chemical shift and random-coiled chemical shift (Wishart et al., 1995).

Table S12. Secondary $^{13}C_{\alpha}$ and $^{1}H_{\alpha}$ chemical shift of Se-glargine and glargine in 10% deuterated acetic acid at pH 2.1 and 25 °C.[a,b]

B domain				
	Se-glargine		Glargine	
residue	2nd C_{α}	2nd H_{α}	2nd C_{α}	2nd H_{α}
F1	0.012	-0.346	-0.008	-0.366
V2	-0.206	-0.009	-0.29	-0.011
N3	0.136	0.138	0.133	0.137
Q4	-2.163	0.249	-1.894	0.275
H5	1.209	-0.07	1.186	-0.041
L6	-0.275	0.179	-0.107	0.184
C7	-4.168	0.627	-3.768	0.597
G8	1.228	0.083	1.248	0.088
S9	2.511	-0.323	2.397	-0.309
H10	1.604	0.044	1.588	0.047
L11	2.76	-0.332	2.886	-0.305
V12	4.776	-0.803	4.818	-0.829
E13	2.139	-0.139	2.175	-0.14
A14	2.567	-0.124	2.507	-0.109
L15	2.935	-0.479	2.764	-0.472
Y16	3.604	-0.279	3.616	-0.248
L17	2.77	-0.273	2.656	-0.25
V18	3.194	-0.226	3.331	-0.28
C19	-3.374	0.344	-3.434	0.357
G20	1.143	0.015	1.141	0.016
E21	1.009	-0.022	0.856	-0.025
R22	1.359	-0.136	1.325	-0.137
G23	-0.726	0.044	-0.714	0.055
F24	-0.087	0.375	-0.173	0.446
F25	-0.091	0.049	-0.106	0.062
Y26	0.281	0.117	0.395	0.134
T27	-2.734	0.321	-2.766	0.343
P28	-0.555	-0.113	-0.549	-0.114
K29	-0.099	0.035	-0.111	0.034
T30	0.035	-0.005	0.04	-0.006
R31	-0.164	0.072	-0.152	0.07
R32	-0.504	0.043	-0.500	0.039

A domain				
	Se-glargine		Glargine	
residue	2nd C_{α}	2nd H_{α}	2nd C_{α}	2nd H_{α}
G1	-2.221	0.118	-2.198	0.079
I2	3.695	-0.348	1.518	-0.138
V3	4.238	-0.569	3.118	-0.458
E4	2.31	-0.08	1.241	-0.006
Q5	2.892	-0.234	2.832	-0.165
C6	-1.77	0.319	-3.564	0.526
C7	-0.561	0.437	-0.589	0.433
T8	3.726	-0.3	3.617	-0.289
S9	-2.527	0.302	-2.401	0.341
I10	-1.119	0.388	-0.657	0.226
C11	-4.39	0.811	-3.716	0.558
S12	-1.871	0.103	-1.962	0.136
L13	3.302	-0.474	3.513	-0.533
Y14	2.269	-0.376	2.418	-0.389
Q15	2.479	-0.255	2.525	-0.246
L16	3.608	-0.166	3.288	-0.149
E17	1.152	-0.025	1.587	-0.017
N18	1.755	-0.082	1.696	-0.072
Y19	1.397	-0.078	1.23	-0.07
C20	-4.277	0.619	-4.466	0.678
G21	-1.471	0.085	-1.46	0.093

[a]All chemical shifts were calibrated in parts per million (ppm) relative to 4,4-dimethyl-4-silapentane-1-sulfonic acid (DSS) as an internal standard, which was set to 0 ppm.
[b]Secondary shift is defined as the differences between observed chemical shift and random coil chemical shift (Wang and Jardetzky, 2002).

Table S13. Backbone $^{1}H_{N}$ and $^{1}H_{\alpha}$ chemical shift of Se-glargine and glargine in 10mM deuterated acetic acid at pH 3.0 (direct meter reading) and 25 °C.[a]

B domain						A domain				
	Se-glargine		Glargine				Se-glargine		Glargine	
residue	$^{1}H_{N}$ (D/M)	$^{1}H_{\alpha}$ (D/M)	$^{1}H_{N}$	$^{1}H_{\alpha}$		residue	$^{1}H_{N}$	$^{1}H_{\alpha}$	$^{1}H_{N}$	$^{1}H_{\alpha}$
F1	/	4.25	/	4.23		G1	/	4.12, 4.03	/	4.11, 4.01
V2	8.16	4.09	8.08	4.08		I2	8.94/8.72	3.81/3.73	8.86/8.63	3.92
N3	8.54	4.70	8.53	4.70		V3	8.55/8.24	3.54/3.23	8.44/8.22	3.60
Q4	8.45/8.48	4.50	8.47/8.58	4.51		E4	8.34/8.31	4.10	8.29/~	4.14
H5	8.73	4.46	8.76	4.41		Q5	8.16	4.05	8.35	4.06
L6	9.19	4.48	9.18	4.51		C6	8.36/8.31	4.89	8.37	4.96
C7	8.29/8.33	5.07	8.38/8.42	5.04		C7	8.24	4.89	8.30/8.22	4.91
G8	9.75/9.65	4.00, 3.90	9.70/9.52	3.94, 3.85		T8	8.57/8.45	3.96	8.52	3.99
S9	8.87/9.26	4.16	8.92/9.19	4.17		S9	6.98/7.15	4.79	7.06	4.81
H10	7.72/8.01	4.34/4.54	8.76	4.35/4.51		I10	7.60/7.79	4.52	7.82	4.38
L11	6.84/7.03	3.93/4.00	6.86/7.08	3.90/4.04		C11	9.74	5.31/5.21	9.88	5.10
V12	6.80/7.10	3.32	6.76/7.07	3.31		S12	8.72	4.57	8.70	4.60
E13	7.65/7.87	3.93/4.11	7.78	4.15		L13	8.63	3.80	8.64	3.73
A14	7.45/7.64	4.16	7.44/7.59	4.14		Y14	7.51	4.16	7.53	4.15
L15	7.69/7.99	3.60/3.78	7.63/7.83	3.61/3.78		Q15	7.54	3.99	7.55	4.01
Y16	7.75/8.17	4.21/4.29	7.74/8.15	4.20		L16	8.21	4.15	8.11	4.18
L17	7.82	4.07	7.81	4.06		E17	7.96/8.02	4.44/4.25	8.06/8.01	4.45/4.26
V18	8.59/8.40	3.85/3.92	8.62/8.42	3.82		N18	7.45	4.50	7.39	4.50
C19	8.62/8.77	4.76	8.60/8.75	4.80		Y19	7.87/8.02	4.49	7.91/8.03	4.49
G20	7.34/7.73	3.95, 3.86	7.42/7.76	3.97, 3.92		C20	7.27/7.35	5.51/5.14	7.29/7.05	5.53/5.18
E21	9.19/8.78	4.17/4.24	9.18/8.83	4.18		G21	8.24	4.05	8.21	4.06
R22	8.00	4.14	8.01	4.18						
G23	7.64/7.17	4.72, 3.78/3.99, 3.76	7.62	4.72, 3.74/4.10, 3.80						
F24	8.87/7.60	5.50/5.06	8.84/7.58	5.53/5.13						
F25	8.54	5.03	8.56	5.03						
Y26	8.51/8.24	4.73	8.50/8.27	4.72						
T27	8.47/7.79	4.57/4.64	8.41/7.76	4.67						
P28	/	4.22	/	4.30						
K29	8.27/8.32	4.51	8.28/8.32	4.50						
T30	8.21/8.08	4.30	8.20/8.13	4.31						
R31	8.45/8.31	4.38	8.44/8.32	4.40						
R32	8.12	4.21	8.14	4.21						

[a]Proton chemical shifts were calibrated in parts per million (ppm) relative to 4,4-dimethyl-4-silapentane-1-sulfonic acid (DSS) as an internal standard, which was set to 0 ppm.

Table S14. $^{13}C_\alpha$, $^{13}C_\beta$ and secondary ^{13}C chemical shift of Se-glargine and glargine in 10mM deuterated acetic acid at pH 3.0 (direct meter reading) and 25 °C.[a]

B domain				
	Se-glargine		Glargine	
residue	$^{13}C_\alpha$/2nd $^{13}C_\alpha$	$^{13}C_\beta$/2nd $^{13}C_\beta$	$^{13}C_\alpha$/2nd $^{13}C_\alpha$	$^{13}C_\beta$/2nd $^{13}C_\beta$
F1	56.97/0.03	39.72/0.29	56.99/0.05	39.77/0.34
V2	61.75/-0.05	33.23/0.55	61.72/-0.08	33.24/0.54
N3	53.18/0.24	38.41/0.19	53.12/0.18	38.28/0.06
Q4	54.38/-1.56	31.25/2.58	53.98/-1.96	31.41/2.74
H5	55.52/-0.26	28.06/-1.56	55.70/-0.08	28.01/-1.61
L6	54.76/-0.09	45.54/3.67	54.78/-0.07	45.80/3.93
C7[b]	53.81/-3.87	48.35/9.97	53.91/-3.77	48.30/9.92
G8	46.72/1.38	/	46.91/1.57	/
S9	61.24/2.89	61.88(62.24)/-2.00(-1.64)	61.08/2.73	62.00/-1.88
H10	57.51/1.94	27.40/-2.22	57.61/1.83	28.06/-1.56
L11	57.45/2.60	39.85/-2.02	57.51/2.66	40.00/-1.87
V12	65.36/4.72	31.37/-1.31	65.86/5.22	31.25/-1.43
E13	58.55/2.16	27.88/-2.14	58.46/2.07	27.70/-2.32
A14	55.26/2.59	18.86(19.02)/-0.17(-0.01)	55.14/2.47	18.97/-0.06
L15	/	40.60/-1.27	57.50/3.00	40.65/-1.22
Y16	60.09/2.37	37.65/-1.06	/	38.02/-0.69
L17	57.71/2.86	42.48/0.61	57.35/2.50	42.35/0.48
V18	65.12/3.32	32.57/-0.11	65.06/3.26	32.66/-0.02
C19[b]	54.00/-3.68	/	54.60/-3.08	36.88/-1.50
G20	46.89/1.55	/	46.50/1.16	/
E21	57.25/0.86	28.15/-1.87	57.17/0.78	/
R22	57.24/1.28	31.30/0.77	57.52/1.56	31.29/0.76
G23	44.94/-0.4	/	45.02/-0.32	/
F24	56.99/0.05	/	57.30/0.36	41.54/2.11
F25	56.33/-0.61	41.25/1.82	55.80/-1.14	41.36/1.93
Y26	56.00/-1.72	41.80/2.37	56.07/-1.65	41.82/2.39
T27	/	70.02/0.27	/	70.00/0.25
P28	/	/	/	/
K29	54.96/-1.44	33.30/0.73	54.87/-1.35	33.25/0.68
T30	61.98/0.39	69.89/0.14	62.03/0.44	69.87/0.12
R31	56.13/0.17	31.01/0.48	56.10/0.14	31.02/0.47
R32	57.04/1.08	31.20/0.67	57.04/1.08	31.22/0.69

A domain				
	Se-glargine		Glargine	
residue	$^{13}C_\alpha$/2nd $^{13}C_\alpha$	$^{13}C_\beta$/2nd $^{13}C_\beta$	$^{13}C_\alpha$/2nd $^{13}C_\alpha$	$^{13}C_\beta$/2nd $^{13}C_\beta$
G1	43.12/-2.22	/	43.30/-2.04	/
I2	62.86/2.22	/	62.25/1.61	/
V3	66.60/5.96	/	66.27/5.63	/
E4	58.79/2.40	/	58.22/1.83	28.86/-1.16
Q5	58.84/2.90	/	58.75/2.81	29.63/0.96
C6	54.56/~	/	54.24/-5.44	/
C7	57.45/-0.23	38.91/0.53	57.34/-0.34	38.51/0.13
T8	65.19/3.60	68.05/-1.70	65.25/3.66	68.18/-1.57
S9	56.85/-1.50	63.91/0.03	56.03/-2.32	63.89/0.01
I10	59.82/-0.82	39.65/1.46	59.91/-0.73	39.59/1.46
C11	53.21/~	/	53.83/-1.74	/
S12	56.61/-1.74	65.71/1.83	56.45/-1.90	65.75/1.87
L13	58.06/3.21	41.21/-0.66	58.36/3.51	41.30/-0.57
Y14	60.19/2.47	37.68/-1.03	60.13/2.41	37.77/-0.94
Q15	58.13/2.19	29.35/0.68	58.01/2.07	29.35/0.68
L16	58.44/3.59	42.16/0.29	57.64/2.79	42.23/0.36
E17	57.54/1.15	28.07/-2.13	57.77/1.38	27.97/- 2.05
N18	54.59/1.65	38.13/-0.09	54.46/1.52	38.35/0.13
Y19	59.04/1.32	37.96/-0.75	58.49/0.77	37.95/-0.74
C20[b]	53.04/-4.64	/	52.50/-5.18	35.67/-2.71
G21	44.64/-0.70	/	44.52/-0.82	/

[a]Proton chemical shifts were calibrated in parts per million (ppm) relative to 4,4-dimethyl-4-silapentane-1-sulfonic acid (DSS) as an internal standard, which was set to 0 ppm.
[b]Unusual secondary ^{13}C chemical shift of cysteine residues may be due to 3D structure packing or aromatic ring effect.

Table S15. HD-exchange results of glargine and Se-glargine in 10 mM dAA at pH 3.0 and 25 °C.

Residue	P. F	ΔG_u	P. F	ΔG_u
	glargine		Se-glargine	
A11HN	19.5±4.5	1.76±0.13	18.9±0.2	1.74±0.01
B6HN	2.9±0.4	0.62±0.09	6.5±0.2	1.10±0.01
A16HA	232.7±11.1	3.23±0.03	313.9±14.1	3.40±0.03
A19HA	257.2±26.7	3.28±0.06	296.8±24.0	3.37±0.05
B18HA[a]	426.9±38.7	3.58±0.05	533.6±24.8	3.72±0.03
A15HA	98.0±3.1	2.71±0.02	105.4±3.6	2.76±0.02
B12HA	69.9±7.5	2.51±0.06	112.8±6.9	2.80±0.04
B14HA	127.7±20.3	2.87±0.09	172.7±12.4	3.05±0.04

[a]ΔG_u at this position indicates that the protein structure should be totally open.

Table S16. HD-exchange results of glargine and Se-glargine in 10% dAA at pH 2.1 and 25 °C.[a]

Residue	K_{obs} (10^{-5})	P. F	ΔG_u	K_{obs} (10^{-5})	P. F.	ΔG_u
		glargine			Se-glargine	
Global effected						
A16 HA[b]	1.01±0.03	127.71±4.36	2.87±0.02	0.83±0.04	155.73±7.04	2.99±0.03
A16HB1	1.03±0.04	124.54±4.57	2.86±0.02	0.89±0.08	145.51±13.23	2.95±0.05
A16HB2	1.04±0.11	123.92±12.84	2.85±0.06	0.88±0.08	146.83±22.29	2.95±0.09
A19 HA	1.85±0.04	99.49±2.34	2.72±0.01	1.64±0.09	111.94±6.21	2.79±0.03
A19HB1	1.85±0.09	99.19±5.07	2.72±0.03	1.98±0.09	93.07±5.68	2.68±0.04
A19HB2	1.73±0.04	106.60±2.34	2.76±0.01	1.79±0.12	104.26±4.52	2.75±0.02
B18HA	1.20±0.05	102.66±3.94	2.74±0.02	0.89±0.02	137.93±2.41	2.92±0.01
B18HB	1.30±0.09	94.83±6.75	2.69±0.04	1.01±0.03	121.84±3.84	2.84±0.02
B18HG1	1.16±0.08	105.90±6.83	2.76±0.04	1.03±0.03	119.41±4.02	2.83±0.02
B18HG2	1.45±0.07	84.87±3.87	2.63±0.03	0.92±0.06	134.32±7.90	2.90±0.03
Sub-global effected						
A14HA	5.49±0.07	32.24±0.43	2.06±0.12	5.62±0.26	31.48±1.45	2.04±0.03
A15HA	3.87±0.09	35.36±0.85	2.11±0.01	3.98±0.13	34.42±1.13	2.09±0.02
A15HB1	4.35±0.27	31.50±1.94	2.04±0.04	4.05±0.09	33.79±0.78	2.08±0.01
A15HB2	4.02±0.07	34.06±0.59	2.09±0.01	3.83±0.04	35.78±0.39	2.12±0.01
A17HA	2.58±0.05	57.29±1.19	2.40±0.01	2.56±0.09	57.70±2.08	2.40±0.02
A17HB	2.68±0.13	55.17±2.67	2.37±0.03	2.99±0.16	49.47±2.59	2.31±0.03
A17HG1	2.41±0.18	61.29±4.50	2.44±0.04	2.09±0.27	70.92±9.15	2.52±0.08
A17HG2	2.72±0.22	54.36±4.33	2.36±0.05	2.24±0.16	66.09±4.86	2.48±0.04
B12HA	2.57±0.08	47.88±1.54	2.29±0.02	2.05±0.07	60.09±2.23	2.42±0.02
B12HB	2.18±0.13	56.31±3.37	2.39±0.03	2.29±0.17	53.67±3.90	2.36±0.04
B12HG	3.10±0.07	39.67±0.89	2.18±0.02	2.03±0.15	60.52±4.52	2.43±0.04
B14HA	4.00±0.13	70.54±2.28	2.52±0.02	3.45±0.09	81.71±2.15	2.61±0.02
B14HB	4.01±0.05	70.28±0.94	2.52±0.01	3.04±0.05	92.72±1.49	2.68±0.01
B15HA	1.76±0.06	96.42±3.34	2.70±0.02	1.55±0.07	109.65±4.79	2.78±0.03
B15HB1	2.49±0.35	68.28±9.73	2.50±0.08	1.64±0.25	103.63±15.91	2.75±0.09
B15HB2	1.67±0.09	101.70±5.71	2.74±0.03	1.44±0.14	117.61±11.42	2.82±0.06
B16HA	2.04±0.13	86.80±5.53	2.64±0.04	1.86±0.04	95.16±2.12	2.70±0.01
B16HB	2.44±0.09	72.56±2.73	2.54±0.02	1.56±0.06	113.31±4.60	2.80±0.03
B17HA	3.60±0.10	32.79±0.88	2.07±0.02	2.51±0.07	47.08±1.31	2.28±0.02
B17HB1	3.53±0.19	33.45±1.78	2.08±0.03	2.62±0.16	44.99±2.67	2.25±0.03
B17HB2	4.03±0.45	29.28±3.26	2.00±0.06	2.31±0.13	51.17±2.81	2.33±0.03
B19HA	4.00±0.20	32.49±1.64	2.06±0.03	3.45±0.10	37.67±1.11	2.15±0.02
B19HB1	4.21±0.18	30.86±1.36	2.03±0.02	3.89±0.30	33.38±2.54	2.08±0.04
B19HB2	4.04±0.13	32.19±1.01	2.05±0.02	3.51±0.06	37.08±0.66	2.14±0.01
Local effected						
A3HN		N/A		45.95±1.43	2.03±0.06	0.42±0.02
A3HA		N/A		48.02±11.36	1.95±0.46	0.39±0.13
A6HN	129.50±8.95	1.22±0.08	0.12±0.04	59.42±2.08	2.66±0.09	0.58±0.02
A7HN	90.38±5.88	2.00±0.13	0.41±0.04	73.03±2.10	2.48±0.07	0.54±0.02
A8HN	85.91±5.31	1.30±0.08	0.16±0.04	64.85±2.10	1.73±0.06	0.32±0.02
A9HA	16.48±1.56	9.10±0.86	1.31±0.06	11.81±2.34	12.70±2.52	1.50±0.12
A10HA	26.51±1.06	7.81±0.31	1.22±0.02	29.97±2.54	6.91±0.58	1.14±0.05
A11HN	24.67±2.01	4.46±0.36	0.88±0.05	25.56±1.73	4.30±0.29	0.86±0.04
A12HA	21.28±2.44	7.85±0.90	1.22±0.07	22.59±3.22	7.39±1.05	1.18±0.08
A13HN	39.00±1.10	3.03±0.08	0.66±0.02	46.22±0.86	2.55±0.05	0.55±0.01
A18HA	8.01±0.25	23.72±0.73	1.87±0.02	8.43±0.31	22.52±0.82	1.84±0.02
A18HB1	8.96±0.46	21.20±1.08	1.81±0.03	8.27±0.32	22.98±0.88	1.86±0.02
A18HB2	7.96±0.42	23.87±1.26	1.88±0.03	7.74±0.37	24.53±1.16	1.89±0.03
A20HA	8.92±0.41	15.35±0.72	1.62±0.03	6.98±1.35	19.63±3.79	1.80±0.11
A20HB	9.13±0.85	15.01±1.39	1.60±0.05	8.62±1.29	15.88±2.38	1.64±0.09
A21HN	100.64±7.90	1.80±0.14	0.35±0.05	59.71±1.58	3.03±0.08	0.66±0.02
B6HN	17.82±5.27	6.73±0.20	1.13±0.02	16.48±2.53	7.28±1.11	1.17±0.09
B6HA	16.58±2.16	7.23±0.94	1.17±0.08	14.80±2.04	8.11±1.11	1.24±0.08
B6HB	12.83±1.00	9.35±0.73	1.32±0.04	11.27±1.99	10.65±1.88	1.40±0.10
B7HN	109.29±6.95	1.10±0.07	0.06±0.02	70.59±1.82	1.70±0.04	0.31±0.02
B11HA	8.70±0.53	13.78±0.83	1.55±0.04	7.35±0.32	16.31±0.71	1.65±0.02
B11HB1	12.69±1.81	9.45±1.34	1.33±0.08	8.81±0.64	13.62±1.90	1.55±0.04
B11HB2	14.32±1.83	8.38±1.07	1.26±0.07	10.40±1.71	11.54±2.23	1.45±0.09
B13HA	7.02±0.24	18.38±0.64	1.72±0.02	4.55±0.17	28.37±1.04	1.98±0.02
B20HA1	49.60±1.65	3.55±0.12	0.75±0.02	30.22±1.54	5.82±0.29	1.04±0.03

[a]In TOCSY spectra, amide protons exhibit NH-αH, NH-βH correlations, and cross peaks with side-chain protons, which enable calculation of multiple separated values for one residue. HN values were obtained from serial 1D experiments. Global, sub-global and local affected amides were defined according to the published P. F. of lispro insulin (KP) (Rege et al., 2020).[b] ΔG_u at this position indicates that the protein structure should be totally open.

The *topology* definitions for the diselenide bridge patch (PRES DSEC) and the selenocysteine residue (RESI SEC) are included in Supplementary **Tables** 17-21. All relevant *parameters* used in the selenium glargine calculations are provided in Supplementary **Tables** 22.

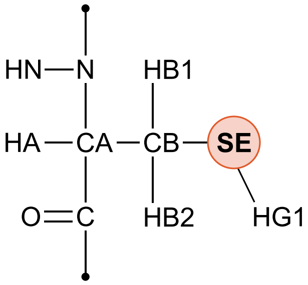

Table S17. Supplementary Table of adjusted and missing *residue topology* and partial charges relevant to the structural characterization of selenocysteine (RESI SEC).

**RESI**	**SEC**	0.00							
**GROUP**	Atom	Type	Charge						
ATOM	N	NH1	–0.47						
ATOM	HN	H	0.31						
ATOM	CA	CT1	0.08						
ATOM	HA	HB1	0.09						
**GROUP**									
ATOM	HB1	HA2	0.10						
ATOM	CB	CT2	–0.13						
ATOM	HB2	HA2	0.10						
ATOM	**SE**	**SX1**	–0.26						
ATOM	HG1	HS	0.18						
**GROUP**									
ATOM	C	C	0.51						
ATOM	O	O	–0.51						
BOND	CB	CA	SE	CB	N	HN	N	CA	
BOND	C	CA	C	+N	CA	HA	CB	HB1	
BOND	CB	HB2	SE	HG1					
DOUBLE	C	O							
IMPR	N	–C	CA	HN	C	CA	+N	O	
CMAP	–C	N	CA	C	N	CA	C	+N	
DONOR	HN	N							
DONOR	HG1	SE							
ACCEPTOR	O	C							
IC	–C	CA	*N	HN	1.3479	123.9300	180.0000	114.7700	0.9982
IC	–C	N	CA	C	1.3479	123.9300	180.0000	105.8900	1.5202
IC	N	CA	C	+N	1.4533	105.8900	180.0000	118.3000	1.3498
IC	+N	CA	*C	O	1.3498	118.3000	180.0000	120.5900	1.2306
IC	CA	C	+N	+CA	1.5202	118.3000	180.0000	124.5000	1.4548
IC	N	C	*CA	CB	1.4533	105.8900	121.7900	111.9800	1.5584
IC	N	C	*CA	HA	1.4533	105.8900	–116.3400	107.7100	1.0837
IC	N	CA	CB	SE	1.4533	111.5600	180.0000	113.8700	1.9610
IC	SE	CA	*CB	HB1	1.9610	113.8700	119.9100	107.2400	1.1134
IC	SE	CA	*CB	HB2	1.9610	113.8700	–125.3200	109.8200	1.1124
IC	CA	CB	SE	HG1	1.5584	113.8700	176.9600	97.1500	1.4780

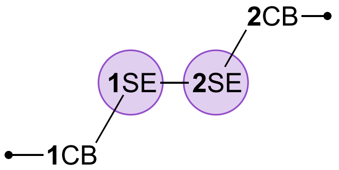

Table S18. Supplementary Table of adjusted and missing *residue topology* and partial charges relevant to the structural characterization of the diselenide bond patch (PRES DSEC).

**PRES**	**DSEC**	–0.42	! Patch for disulfides.[a]						
**GROUP**	Atom	Type	Charge						
ATOM	1CB	CT2	–0.10	! 2SE—2CB					
ATOM	**1SE**	**SX2**	–0.11						
**GROUP**									
ATOM	**2SE**	**SX2**	–0.11	! CB—1SE					
ATOM	2CB	CT2	–0.10						
DELETE	ATOM	1HG1							
DELETE	ATOM	2HG1							
BOND	1SE	2SE							
IC	1CA	1CB	1SE	2SE	0.0000	0.0000	180.0000	0.0000	0.0000
IC	1CB	1SE	2SE	2CB	0.0000	0.0000	90.0000	0.0000	0.0000
IC	1SE	2SE	2CB	2CA	0.0000	0.0000	180.0000	0.0000	0.0000

[a] Patch must be 1-SEC and 2-SEC and follow with AUTOgenerate ANGLEs DIHEdrals command.

Table S19. Supplementary Table of adjusted and missing *bonding* parameters relevant to the structural characterization of selenocysteine residues and diselenide bonds.

! === Selenocysteine ($S_{\beta}EC$) bonds ===				
CT2	SX1	180.500	1.9610	! C_{β}–Se bond (SEC)
SX1	HS	200.000	1.4780	! Se–H bond (SEC side chain)
! === Diselenide (DSEC) bridge bonds ===				
CT2	SX2	180.500	1.9610	! C_{β}–Se bond (DSEC)
SX2	SX2	150.000	2.3500	! Se–Se bond (DSEC)
! === Mixed bond (SEC–DSEC mixed diselenide) ===				
SX1	SX2	150.000	2.3500	! Mixed Se–Se bond (SEC–DSEC)

Table S20. Supplementary Table of adjusted and missing *angular* parameters relevant to the structural characterization of selenocysteine residues and diselenide bonds.

! === Selenocysteine (SEC) Angles ===					
CT2	SX1	HS	50.000	98.0000	! C_β–Se–H
HA2	CT2	SX1	42.500	108.7600	! H_α–C_β–Se
CT2	SX1	SX1	40.000	100.0000	! C_β–Se–(X)
SX1	SX1	CT2	40.000	100.0000	! (X)–Se–C_β
! === Diselenide (DSEC) Angles ==					
CT2	SX2	SX2	40.000	100.0000	! C_β–Se–Se
SX2	SX2	CT2	40.000	100.0000	! Se–Se–C_β
! === SEC–DSEC Mixed Angles ==					
CT2	SX1	SX2	40.000	100.0000	! C_β–Se1–Se2
SX1	SX2	CT2	40.000	100.0000	! Se1–Se2–C_β
! === DSEC Patch-Specific Angles ===					
SX2	CT2	SX2	40.000	100.0000	! Se–C_β–Se (bridging)
SX2	SX2	SX2	40.000	100.0000	! symmetric Se–Se–Se
! === Backbone–Se Angles (required for compatibility) ===					
CT1	CT2	SX2	42.500	110.0000	! C_α–C_β–Se
HA2	CT2	SX2	42.500	108.0000	! H_α–C_β–Se
SX2	CT2	CT1	42.500	110.0000	! reverse
SX2	CT2	HA2	42.500	108.0000	! reverse
! === Additional compatibility angles (backbone–sidechain couplings) =================================					
HB1	CT1	CT2	42.500	110.0000	! H_β–C_α–C_β
NH1	CT1	CT2	42.500	111.0000	! N–C_α–C_β
C	CT1	CT2	42.500	111.0000	! C–C_α–C_β

Table S21. Supplementary Table of adjusted and missing *dihedral* parameters relevant to the structural characterization of selenocysteine residues and diselenide bonds.

! === Selenocysteine (SEC, SX1) Dihedrals ==							
CT1	CT2	SX1	SX1	1.000	3	0.0	! backbone to sidechain: C_α–C_β–Se–H
SX1	SX1	CT2	CT1	1.000	3	0.0	! reversed: H–Se–C_β–C_α
! === Diselenide (DSEC, SX2) Dihedrals ===							
CT2	SX2	SX2	CT2	1.000	3	0.0	! sidechain: C_β–Se–Se–C_β (DSEC diselenide)
CT1	CT2	SX2	SX2	1.000	3	0.0	! backbone to sidechain: C_α–C_β–Se–Se
SX2	SX2	CT2	CT1	1.000	3	0.0	! reversed: Se–Se–C_β–C_α
! === Mixed SEC–DSEC Dihedrals ==							
CT2	SX1	SX2	CT2	1.000	3	0.0	! sidechain: C_β–Se1–Se2–C_β
CT2	SX2	SX1	CT2	1.000	3	0.0	! sidechain: C_β–Se2–Se1–C_β
! === Additional Backbone–Sidechain Coupling Dihedrals (DSEC) ==================================							
HA2	CT2	SX2	SX2	1.000	3	0.0	! H_α–C_β–Se–Se (geometry)
HB1	CT1	CT2	SX2	1.000	3	0.0	! H_β–C_α–C_β–Se
NH1	CT1	CT2	SX2	1.000	3	0.0	! N–C_α–C_β–Se
C	CT1	CT2	SX2	1.000	3	0.0	! C–C_α–C_β–Se

Table S22. Supplementary Table of adjusted and missing *nonbonded* and *mass* parameters relevant to the structural characterization of selenocysteine residues and diselenide bonds.

! === Custom selenium atom types (from selenomethionine/MeSe-benchmarks)[a] =========================				
Atom type	placeholder[b]	ε (kcal·mol^{-1})	$\underline{R}_{min}/2$[c] (Å)	Comment
SX1	0.000000	–0.200000	2.200000	! Se in RESI SEC selenocysteine (C–Se–H/C)
SX2	0.000000	–0.190000	2.420000	! Se in patch DSEC diselenide (C–Se–Se–C)
! === Selenium atom masses placed in the *par_all36m_prot.prm* file ==================================				
MASS	–1	SX1	78.96000	! Se in RESI SEC selenocysteine (C–Se–H/C)
MASS	–1	SX2	78.96000	! Se in patch DSEC diselenide (C–Se–Se–C)

[a] Adopted and scaled using values from selenomethionine/MeSe benchmarks by (Pedron et al., 2023). [b] The second column is an often-ignored placeholder used in historical or in exotic models. [c] Equivalent to the van der Waals radius of the given atom.

Table S23. Supplementary table of measured interatomic ($C_\alpha \cdots C_\alpha$) distances in the conformers (frame #0001) of reported Aaron Dinner's islands named **C0–C9**. Classification is binned into "proximal" and "distant" with value-ranges from 0–7.0 Å and 7.0>, respectively.

conformer #	A6–B6 ($C_\alpha \cdots C_\alpha$)	A6–B6 (classification)	B4–A11 ($C_\alpha \cdots C_\alpha$)	B4–A11 (classification)
**C0**	5.4	proximal	5.7	proximal
**C1**	11.2	distant	19.8	distant
**C2**	5.9	proximal	5.5	proximal
**C3**	11.2	distant	20.3	distant
**C4**	10.2	distant	19.8	distant
**C5**	6.7	proximal	9.9	distant
**C6**	5.6	proximal	5.2	proximal
**C7**	8.3	distant	15.7	distant
**C8**	10.3	distant	13.2	distant
**C9**	5.6	proximal	5.2	proximal

References

Armishaw CJ, Daly NL, Nevin ST, Adams DJ, Craik DJ, Alewood PF. α-Selenoconotoxins, a new class of potent α7 neuronal nicotinic receptor antagonists. J Biol Chem. 2006;281:14136-43.

Armstrong DA, Kaas Q, Rosengren KJ. Prediction of disulfide dihedral angles using chemical shifts. Chem Sci. 2018;9:6548-56. 10.1039/c8sc01423j

Bai Y, Milne JS, Mayne L, Englander SW. Primary structure effects on peptide group hydrogen exchange. Proteins. 1993;17:75-86. 10.1002/prot.340170110

Baker EN, Blundell TL, Cutfield JF, Cutfield SM, Dodson EJ, Dodson GG et al. The Structure of 2Zn Pig Insulin Crystals at 1.5-Å Resolution. Philos Trans R Soc Lond, Ser B: Biol Sci. 1988;319:369-456. https://doi.org/10.1098/rstb.1988.0058

Ben-Nissan G, Sharon M. Capturing protein structural kinetics by mass spectrometry. Chem Soc Rev. 2011;40:3627-37.

Bolli GB, Owens DR. Insulin glargine. The Lancet. 2000;356:443-45. https://doi.org/10.1016/S0140-6736(00)02546-0

Bondi Av. van der Waals volumes and radii. J Phys Chem. 1964;68:441-51.

Brange J. 1987. Galenics of insulin: the physico-chemical and pharmaceutical aspects of insulin and insulin preparations, Springer Berlin Heidelberg, Berlin. https://doi.org/10.1007/978-3-662-02526-0

Brange J, Langkjaer L. Insulin formulation and delivery. Pharm Biotechnol. 1997;10:343-409. 10.1007/0-306-46803-4_13

Brooks BR, Brooks III CL, Mackerell Jr AD, Nilsson L, Petrella RJ, Roux B et al. CHARMM: the biomolecular simulation program. J Comput Chem. 2009;30:1545-614. https://doi.org/10.1002/jcc.21287

Busto-Moner L, Feng C-J, Antoszewski A, Tokmakoff A, Dinner AR. Structural ensemble of the insulin monomer. Biochemistry. 2021;60:3125-36. https://doi.org/10.1021/acs.biochem.1c00583

Chance RE, Frank BH. Research, development, production, and safety of biosynthetic human insulin. Diabetes Care. 1993;16:133-42. https://doi.org/10.2337/diacare.16.3.133

Choi WE, Borchardt D, Kaarsholm NC, Brzovic PS, Dunn MF. Spectroscopic evidence for preexisting T- and R-state insulin hexamer conformations. Proteins: Struct Funct Bioinform. 1996;26:377-90. https://doi.org/10.1002/(SICI)1097-0134(199612)26:4<377::AID-PROT2>3.0.CO;2-9

Chothia C, Lesk AM, Dodson GG, Hodgkin DC. Transmission of conformational change in insulin. Nature. 1983;302:500-05. https://doi.org/10.1038/302500a0

Dawson PE. Native chemical ligation combined with desulfurization and deselenization: a general strategy for chemical protein synthesis. Isr J Chem. 2011;51:862-67.

Dery L, Reddy PS, Dery S, Mousa R, Ktorza O, Talhami A et al. Accessing human selenoproteins through chemical protein synthesis. Chem Sci. 2017;8:1922-26.

Dodson G, Steiner D. The role of assembly in insulin's biosynthesis. Curr Opin Struct Biol. 1998;8:189-94. https://doi.org/10.1016/s0959-440x(98)80037-7

Eriksson AE, Baase WA, Wozniak JA, Matthews BW. A cavity-containing mutant of T4 lysozyme is stabilized by buried benzene. Nature. 1992;355:371-3.

Fobe TL, Kazakov A, Riccardi D. Cys.sqlite: A Structured-Information Approach to the Comprehensive Analysis of Cysteine Disulfide Bonds in the Protein Databank. J Chem Inf Model. 2019;59:931-43. 10.1021/acs.jcim.8b00950

Gowd KH, Yarotskyy V, Elmslie KS, Skalicky JJ, Olivera BM, Bulaj G. Site-specific effects of diselenide bridges on the oxidative folding of a cystine knot peptide, ω-selenoconotoxin GVIA. Biochemistry. 2010;49:2741-52.

Heinemann L, Braune K, Carter A, Zayani A, Krämer LA. Insulin storage: a critical reappraisal. J Diabetes Sci Technol. 2021;15:147-59. https://doi.org/10.1177/1932296819900258

Hondal RJ, Marino SM, Gladyshev VN. Selenocysteine in thiol/disulfide-like exchange reactions. Antioxid Redox Signal. 2013;18:1675-89. https://doi.org/10.1089/ars.2012.5013

Huber R, Criddle R. Comparison of the chemical properties of selenocysteine and selenocystine with their sulfur analogs. Arch Biochem Biophys. 1967;122:164-73. https://doi.org/10.1016/0003-9861(67)90136-1

Jahn TR, Radford SE. The Yin and Yang of protein folding. The FEBS Journal. 2005;272:5962-70. https://doi.org/10.1111/j.1742-4658.2005.05021.x

Jammalamadaka SR, SenGupta A. 2001. Topics in Circular Statistics, WORLD SCIENTIFIC. doi:10.1142/4031

Jarosinski MA, Dhayalan B, Chen YS, Chatterjee D, Varas N, Weiss MA. Structural principles of insulin formulation and analog design: A century of innovation. Mol Metab. 2021;52:101325. 10.1016/j.molmet.2021.101325

Jimenez JL, Nettleton EJ, Bouchard M, Robinson CV, Dobson CM, Saibil HR. The protofilament structure of insulin amyloid fibrils. Proc Natl Acad Sci. 2002;99:9196-201. https://doi.org/10.1073/pnas.142459399

Karplus M. Dynamics of proteins. Adv Biophys. 1984;18:165-90.

Katsoyannis P. Synthesis of insulin. Science. 1966;154:1509-14. https://doi.org/10.1126/science.154.3756.1509

Kildahl NK. Bond energy data summarized. J Chem Educ. 1995;72:423.

Kjeldsen T, Andersen AS, Hubálek F, Johansson E, Kreiner FF, Schluckebier G et al. Molecular engineering of insulin for recombinant expression in yeast. Trends Biotechnol. 2024;42:464-78. https://doi.org/10.1016/j.tibtech.2023.09.012

Koradi R, Billeter M, Wuthrich K. MOLMOL: a program for display and analysis of macromolecular structures. J Mol Graph. 1996;14:51-5, 29-32. https://doi.org/10.1016/0263-7855(96)00009-4

Lenton S, Chaaban H, Khaled M, van de Weert M, Strodel B, Foderà V. Insulin amyloid morphology is encoded in H-bonds and electrostatics interactions ruling protein phase separation. Journal of colloid and interface science. 2025;683:1175-87.

Maglic JB, Lavendomme R. MoloVol: an easy-to-use program for analyzing cavities, volumes and surface areas of chemical structures. J Appl Crystallogr. 2022;55:1033-44. https://doi.org/10.1107/s1600576722004988

Metanis N, Hilvert D. Strategic use of non-native diselenide bridges to steer oxidative protein folding. Angew Chem, Int Ed. 2012;51:5585-88.

Metanis N, Hilvert D. Harnessing selenocysteine reactivity for oxidative protein folding. Chem Sci. 2015;6:322-25. https://doi.org/10.1039/c4sc02379j

Mousa R, Notis Dardashti R, Metanis N. Selenium and selenocysteine in protein chemistry. Angew Chem Int Ed. 2017;56:15818-27. https://doi.org/10.1002/anie.201706876

Mulder FA, Mittermaier A, Hon B, Dahlquist FW, Kay LE. Studying excited states of proteins by NMR spectroscopy. Nat Struct Biol. 2001;8:932-35.

Nielsen L, Frokjaer S, Brange J, Uversky VN, Fink AL. Probing the mechanism of insulin fibril formation with insulin mutants. Biochemistry. 2001;40:8397-409. https://doi.org/10.1021/bi0105983

Palmer III AG. Dynamic properties of proteins from NMR spectroscopy. Curr Opin Biotechnol. 1993;4:385-91.

Pedron FN, Messias A, Zeida A, Roitberg AE, Estrin DA. Novel lennard-jones parameters for cysteine and selenocysteine in the AMBER force field. J Chem Inf Model. 2023;63:595-604. https://doi.org/10.1021/acs.jcim.2c01104

Pegoraro S, Fiori S, Cramer J, Rudolph-Bohner S, Moroder L. The disulfide-coupled folding pathway of apamin as derived from diselenide-quenched analogs and intermediates. Protein Sci. 1999;8:1605-13. 10.1110/ps.8.8.1605

Petznick A. Insulin management of type 2 diabetes mellitus. Am Fam Physician. 2011;84:183-90. https://www.aafp.org/pubs/afp/issues/2011/0715/p183.html

Ratha BN, Kar RK, Bednarikova Z, Gazova Z, Kotler SA, Raha S et al. Molecular details of a salt bridge and its role in insulin fibrillation by NMR and Raman spectroscopic analysis. The Journal of Physical Chemistry B. 2020;124:1125-36. https://doi.org/10.1021/acs.jpcb.9b10349

Rege NK, Liu M, Yang YW, Dhayalan B, Wickramasinghe NP, Chen YS et al. Evolution of insulin at the edge of foldability and its medical implications. Proc Natl Acad Sci. 2020;117:29618-28. https://doi.org/10.1073/pnas.2010908117

Richards FM. The interpretation of protein structures: total volume, group volume distributions and packing density. J Mol Biol. 1974;82:1-14.

Roder H, Wagner G, Wuthrich K. Amide proton exchange in proteins by EX1 kinetics: studies of the basic pancreatic trypsin inhibitor at variable p2H and temperature. Biochemistry. 1985;24:7396-407.

Sosnick TR, Fang X, Shelton VM. Application of circular dichroism to study RNA folding transitions. Methods Enzymol. 2000;317:393-409. https://doi.org/10.1016/s0076-6879(00)17026-0

Sousa SF, Neves RP, Waheed SO, Fernandes PA, Ramos MJ. Structural and mechanistic aspects of SS bonds in the thioredoxin-like family of proteins. Biol Chem. 2019;400:575-87. https://doi.org/10.1515/hsz-2018-0319

Stadtman TC. Selenocysteine. Annu Rev Biochem. 1996;65:83-100.

Suladze S, Sarkar R, Rodina N, Bokvist K, Krewinkel M, Scheps D et al. Atomic resolution structure of full-length human insulin fibrils. Proc Natl Acad Sci. 2024;121:e2401458121. https://doi.org/10.1073/pnas.2401458121

Thim L, Hansen MT, Norris K, Hoegh I, Boel E, Forstrom J et al. Secretion and processing of insulin precursors in yeast. Proc Natl Acad Sci. 1986;83:6766-70. https://doi.org/10.1073/pnas.83.18.6766

Wang L, Hall CE, Uchikawa E, Chen D, Choi E, Zhang X et al. Structural basis of insulin fibrillation. Science Advances. 2023;9:eadi1057. https://doi.org/10.1126/sciadv.adi1057

Wang Y, Jardetzky O. Probability-based protein secondary structure identification using combined NMR chemical-shift data. Protein Sci. 2002;11:852-61. https://doi.org/10.1110/ps.3180102

Waugh DF. The properties of protein fibers produced. Reversibly from soluble protein molecules. Am J Physiol. 1941;133:P484-P85.

Waugh DF. A fibrous modification of insulin. I. The heat precipitate of insulin. J Am Chem Soc. 1946;68:247-50. https://doi.org/10.1021/ja01206a030

Waugh DF, Wilhelmson DF, Commerford SL, Sackler ML. Studies of the nucleation and growth of selected types of insulin fibrils. J Am Chem Soc. 1953;75:2592-600.

Weil-Ktorza O, Dhayalan B, Chen YS, Weiss MA, Metanis N. Se-glargine : chemical synthesis of a basal insulin analogue stabilized by an internal diselenide bridge. ChemBioChem. 2024;25:e202300818. https://doi.org/10.1002/cbic.202300818

Weil-Ktorza O, Rege N, Lansky S, Shalev DE, Shoham G, Weiss MA et al. Substitution of an internal disulfide bridge with a diselenide enhances both foldability and stability of human insulin. Chem Eur J. 2019;25:8513–21. https://doi.org/10.1002/chem.201900892

Weiss MA. The structure and function of insulin: decoding the TR transition. Vitam Horm. 2009;80:33-49. https://doi.org/10.1016/S0083-6729(08)00602-X

Wetzel R. Kinetics and thermodynamics of amyloid fibril assembly. Acc Chem Res. 2006;39:671-79.

Wishart DS, Bigam CG, Holm A, Hodges RS, Sykes BD. ^{1}H, ^{13}C and ^{15}N random coil NMR chemical shifts of the common amino acids. I. Investigations of nearest-neighbor effects. J Biomol NMR. 1995;5:67-81. https://doi.org/10.1007/bf00227471

Zaykov AN, Mayer JP, DiMarchi RD. Pursuit of a perfect insulin. Nat Rev Drug Discov. 2016.
